# Restriction of an intron size *en route* to endothermy

**DOI:** 10.1093/nar/gkab046

**Published:** 2021-02-08

**Authors:** Jana Královičová, Ivana Borovská, Reuben Pengelly, Eunice Lee, Pavel Abaffy, Radek Šindelka, Frank Grutzner, Igor Vořechovský

**Affiliations:** University of Southampton, Faculty of Medicine, HDH, Southampton SO16 6YD, UK; Slovak Academy of Sciences, Centre for Biosciences, 840 05 Bratislava, Slovak Republic; Slovak Academy of Sciences, Centre for Biosciences, 840 05 Bratislava, Slovak Republic; University of Southampton, Faculty of Medicine, HDH, Southampton SO16 6YD, UK; School of Biological Sciences, University of Adelaide, Adelaide 5005, SA, Australia; Czech Academy of Sciences, Institute of Biotechnology, 25250 Vestec, Czech Republic; Czech Academy of Sciences, Institute of Biotechnology, 25250 Vestec, Czech Republic; School of Biological Sciences, University of Adelaide, Adelaide 5005, SA, Australia; University of Southampton, Faculty of Medicine, HDH, Southampton SO16 6YD, UK

## Abstract

Ca^2+^-insensitive and -sensitive E1 subunits of the 2-oxoglutarate dehydrogenase complex (OGDHC) regulate tissue-specific NADH and ATP supply by mutually exclusive *OGDH* exons 4a and 4b. Here we show that their splicing is enforced by distant lariat branch points (dBPs) located near the 5′ splice site of the intervening intron. dBPs restrict the intron length and prevent transposon insertions, which can introduce or eliminate dBP competitors. The size restriction was imposed by a single dominant dBP in anamniotes that expanded into a conserved constellation of four dBP adenines in amniotes. The amniote clusters exhibit taxon-specific usage of individual dBPs, reflecting accessibility of their extended motifs within a stable RNA hairpin rather than U2 snRNA:dBP base-pairing. The dBP expansion took place in early terrestrial species and was followed by a uridine enrichment of large downstream polypyrimidine tracts in mammals. The dBP-protected megatracts permit reciprocal regulation of exon 4a and 4b by uridine-binding proteins, including TIA-1/TIAR and PUF60, which promote U1 and U2 snRNP recruitment to the 5′ splice site and BP, respectively, but do not significantly alter the relative dBP usage. We further show that codons for residues critically contributing to protein binding sites for Ca^2+^ and other divalent metals confer the exon inclusion order that mirrors the Irving-Williams affinity series, linking the evolution of auxiliary splicing motifs in exons to metallome constraints. Finally, we hypothesize that the dBP-driven selection for Ca^2+^-dependent ATP provision by E1 facilitated evolution of endothermy by optimizing the aerobic scope in target tissues.

## INTRODUCTION

Endothermy is the maintenance of an elevated and constant body temperature (*T*_b_) by metabolic means (1–5). Endotherms (largely mammals and birds) have (i) a much higher aerobic activity and (ii) superior thermoregulation as compared to ‘cold-blooded’ animals or ectotherms (1–5). For decades, the two metabolic traits have been at the centre of controversy to explain the acquisition of endothermy (1–4). An early but most widely accepted hypothesis to elucidate selection forces that led to the emergence of ‘warm-blooded’ species, known as the aerobic scope (or capacity) model (5), posited that endothermy evolved mainly through selection for high locomotor activity sustained by improved aerobic metabolism, without primarily selecting for an enhanced thermoregulation or higher *T*_b_. The model has been supported by a functional link between resting and maximal rates of oxygen consumption (RMR and MMR) during vertebrate evolution and by fossil records that were indicative of an increased locomotion and easier ventilation, such as maxilloturbinates (2–4). The hypothesis has gained further support from recent extensive phylogenetic studies (6,7), however, molecular mechanisms that led to selection of the most important metabolic conversion in animal history remain elusive.

The maximum sustainable level of aerobic metabolism and locomotor activity requires a responsive supply of ATP by mitochondria and its conversion to mechanical energy in muscles. One of the most effective signals for sustained aerobic stimulation and ATP production are frequency oscillations of mitochondrial (Ca^2+^_m_) and free cytosolic (Ca^2+^_c_) calcium (8–13). Ca^2+^_m_ levels are elevated by plasma membrane depolarization of muscle fibres (14) and their increase requires intact Ca^2+^ channels (IP3R and RYR1) in the sarcoplasmic reticulum (15). Mitochondrial uptake of IP3R-released Ca^2+^ is essential for sufficient supply of reducing equivalents for oxidative phosphorylation (16). The Ca^2+^_m_ accumulation triggers rapid activation of the mitochondrial metabolic machinery, enhancing oxygen consumption and ATP synthesis (9,15,17). Increased Ca^2+^_m_ levels activate Ca^2+^-dependent dehydrogenases (DHs) in the mitochondrial matrix, either indirectly by dephosphorylation (pyruvate DH) or directly by Ca^2+^-binding (isocitrate DH and 2-oxoglutarate DH) (18–20). These enzymes are key components of the tricarboxylic acid (TCA or Krebs) cycle, which controls NADH- and FADH_2_-dependent oxidative phosphorylation and ATP supply under aerobic conditions.

A critical control point in the TCA cycle is exerted by the conversion of 2-oxoglutarate (2OG) to succinyl-CoA by OGDHC, generating NADH and electrons for the respiratory chain and increasing the cycle flux and the rate of ATP synthesis (9,17,21–23). Of the Ca^2+^-dependent matrix DHs, OGDHC is most sensitive to low ATP/ADP ratios (20). OGDHC belongs to the essential high-flux metabolic backbone that is conserved between *Escherichia coli* and humans (24) but is absent in cyanobacteria where the missing TCA cycle step is compensated by alternative pathways, consistent with their energy produced phototrophically rather than by cellular respiration (25). OGDHC is required for efficient respiration of both animal and plant cells, particularly under conditions of increased energy demand (21–23). Under oxidative stress, OGDHC inhibition limits the amount of NADH available for the respiratory chain (26), in line with its high flux control coefficient and critical contribution to the shared TCA cycle control (21,23 and refs. therein). The systemic importance of the OGDHC branch point in the TCA cycle is supported by its rich allosteric regulation by Ca^2+^ and ADP/ATP, NADH/NAD^+^ and acyl-CoA/CoA ratios at subsaturating concentrations of 2OG (19,20,22,27,28). However, a role of this candidate complex in the acquisition of endothermy has not been studied.

**Figure 1.**
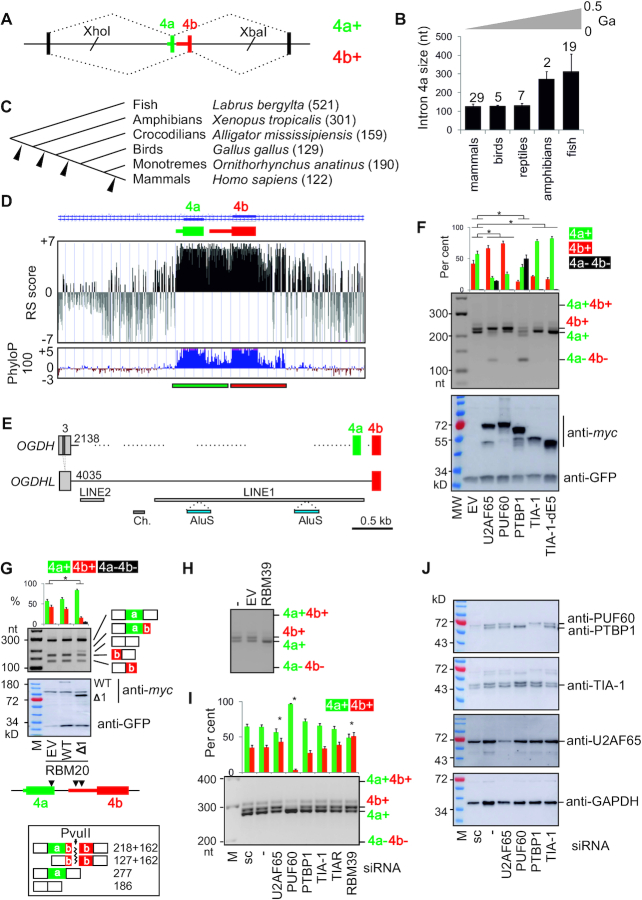
Intron 4a as a regulator of *OGDH* MXEs and target for pyrimidine-binding proteins that control Ca^2+^-sensitive and -insensitive isoforms. (**A**) *OGDH* reporter construct. Introns are shown as horizontal lines, exons as boxes and spliced products (*right*) by dotted lines. MXEs are numbered at the top. Introns are numbered by their genomic location in the main text. The length of MXE PPTs is indicated by coloured horizontal bars. (**B**) Average size of intron 4a in vertebrate classes. Error bars are SDs. The number of species is indicated above each bar. Approximate evolutionary span is at the top (not to scale). Ga, giga-annum. (**C**) Intron 4a size in representative species (in nts). Arrowheads denote deletion events. (**D**) RS (rejected substitutions) scores (65) (*upper panel*) and PhyloP 100 vertebrates conservation (*lower panel*) of the MXE pair. Rectangles at the bottom denote a tentative exon duplication event. (**E**) TEs (horizontal bars) in *OGDH* and *OGDHL* introns 3 and 4a. Their size (in nts) in the reference genome is shown at their 5′ ends. Exons are numbered at the *top*. Dotted lines show regions weakly aligned with *OGDHL*. LINE, long interspersed elements; Ch., DNA transposon Charlie. (**F**) Regulation of *OGDH* MXEs by U-binding proteins. The *OGDH* reporter was cotransfected with expression constructs (*bottom*). *OGDH* mRNA isoforms (*middle panel*) are measured at the top. Error bars are SDs of two independent transfections. Asterisks denote significant *P* values (<0.05; ANOVA, Dunnett's post-hoc tests) for the indicated comparisons. MW, size marker; EV, empty vector. Immunoblot with lysates prepared from transfected cells is in the *lower panel*; antibodies are to the *right*. (**G**) RBM20 is a repressor of *OGDH* exon 4b. Here, RT-PCR products are cut with PvuII, which cleaves exon 4b; digested products and their sizes (in nts) are shown at the *bottom*. Coexpression of the *OGDH* reporter with RBM20 constructs is shown in the middle panel; arrowheads below denote the presence of optimal binding sites of RBM20 (UCUU) (81) located in the 5′ part of megaPPT (segment del2, Figure 2A) and in the 3′ part of exon 4a. *Ogdh* was identified among cardiomyocyte RBM20 HITS-CLIP targets (10 intronic reads) although no *Ogdh* exons were listed among high-confidence RBM20-regulated exons (FDR<0.05) identified by comparing *Rbm20* (−/−) and WT rats (81). (**H**, **I**) Opposite effects of RBM39 overexpression (H) and depletion (I, last lane) (40) on exon 4a/4b ratios. (I) Exon 4a/4b usage in HEK293 cells depleted of Y-binding proteins. sc, a scrambled control. Spliced products are measured at the top; error bars are SDs of two transfections. Asterisks show statistically significant deviations from controls (ANOVA with post-hoc Dunnett's tests). (**J**) Immunoblots for panel (I); antibodies are to the right.

The initial, substrate-specific and irreversible stage of the OGDHC reaction is catalyzed by the E1 subunit (2-oxoglutarate dehydrogenase; OGDH; EC 1.2.4.2). E1 (also known as E1_o_) has the lowest catalytic activity among OGDHC components and is rate-limiting in multiple organisms (22,29,30) whereas E2 and E3 subunits are shared with other enzymatic complexes (31). E1 is encoded by the *OGDH* gene. *OGDH* sustained an ancient exon duplication that gave rise to transcripts with mutually exclusive exons (MXEs) 4a and 4b (Figure 1A), also known as LS1 and S1, respectively (32,33). The exon 4a-encoded E1 isoform (4a+) is Ca^2+^-insensitive, whereas isoform 4b+ evolved a peptide motif (DADLD) that was shown to be essential for Ca^2+^ binding and OGDHC activation (32,33). Isoform 4b+ is prominently expressed in striated muscles but its relative abundance in viscera is lower (32,33). Although this MXE pair acts as a key on/off switch of tissue-specific and activity-dependent energy supply, it is unclear how the inclusion of only one of the two exons in the mRNA is controlled and how the MXE regulation evolved in ecto- and endotherms.

In this study, we first show that splicing of *OGDH* MXEs is enforced by a cluster of distant lariat branch points (dBPs) in the intervening intron. Human dBP adenines are located only 25–41 nucleotides (nt) downstream of the 5′ splice site (5′ss) of exon 4a, below a proposed steric threshold for simultaneous assembly of spliceosomal complexes recruited to 5′ss and BPs (34–36). The separation of dBPs from the 3′ss was present already in anamniotes, precluding expansion of this intron over hundreds of Myrs of vertebrate evolution. Transposed elements (TEs), a key source of intron enlargements in vertebrates (37,38), inserted upstream or downstream of dBPs introduced or eliminated dBPs competitors, impaired the MXE pattern and/or increased unproductive transcripts, thereby limiting the Ca^2+^-dependent OGDHC activation. Following the dBP expansion in early terrestrial species, the intron size was further reduced, which was followed by a uridine enrichment of large polypyrimidine tracts (megaPPTs) downstream of mammalian dBPs. We propose that this enrichment contributed to the observed sensitivity of the MXE pair to RNA-binding proteins (RBPs) with uridine preferences that ensure reciprocal regulation of each exon. The dBP-driven and Ca^2+^-responsive isoform 4b+ led us to investigate the capacity of exonic auxiliary splicing elements to include/exclude binding sites for Ca^2+^ and other common metals in/from coding sequences. We further show that evolution of exonic splicing enhancers and silencers has been shaped by codons that are critical for formation of protein binding sites for metals at the opposite ends of the Irving-Williams affinity order: codons for amino acids required for binding weak metals, such as Ca^2+^, generally increase exon inclusion in mature transcripts whereas codons for amino acids required for strong binders, such as copper, promote exon exclusion. Finally, we hypothesize that the *Ogdh* alternative splicing facilitated evolution of endothermy by maximizing the aerobic scope of striated muscles while protecting other tissues from NADH oversupply.

## MATERIALS AND METHODS

### Splicing reporters and mammalian expression constructs

The human *OGDH* reporter (Figure 1A) was obtained by cloning a 1.9-kb XhoI/XbaI fragment containing exon 4a, intron 4a, exon 4b and flanking intronic sequences into the hybrid pCR3.1 reporter described previously (39). Taxon-specific reporter plasmids were obtained by PCR using primers in Supplementary Table S1 and DNA from the indicated species as a template. Plasmids were mutated by overlap-extension PCR and validated by Sanger sequencing (Eurofins). Mutations and mutagenic primers are shown in Supplementary Table S1. Constructs expressing the indicated *myc*-tagged proteins were prepared with primers shown in Supplementary Table S1 or were described previously (40,41). Their source is also described in the Acknowledgement section.

### Construction of a retroposon library

A population of mammalian interspersed repeats (MIRs) was amplified by PCR using degenerate primers (Supplementary Table S1) that targeted the ends of consensus sequences of MIR subfamilies (42). The amplicons were obtained with a mixture of human DNAs using varying annealing temperatures at 1.5 mM Mg^2+^, were sized between 224 and 268 nts and contained high densities of BP motifs (Supplementary Table S2). The PCR products were separated on 1% agarose gels. The indicated fragments were extracted with the GeneJET Gel Extraction Kit (ThermoFisher), digested and cloned into the unique PstI or EcoRV site introduced upstream or downstream of the dBP cluster, respectively, permitting both sense and antisense MIR orientations. Ligation reactions were introduced into the *E. coli* strain DH5α (Invitrogen) by transformation. Plasmid DNA was extracted with the GeneJET Plasmid Miniprep kit (ThermoFisher) and correctly sized inserts were confirmed by gel electrophoresis following digestions with restriction enzymes. Sanger sequencing of 30 plasmids confirmed 9 (PstI) and 16 (EcoRV) constructs with unique MIR inserts (Supplementary Table S3). Plasmid DNA was individually transfected into human embryonic kidney cells (HEK293) to examine the impact of sense and antisense MIRs on exon 4a/4b and BP usage.

### Splicing assays

Cell lines were grown in DMEM in 12- or 24-well plates, as described in detail (41). Transient (co)transfections were carried out with wild-type (WT) or mutated splicing reporters, *myc*-tagged mammalian expression constructs, pcDNA3.1-GFP as transfection/loading control and jetPRIME (Polyplus) as a transfection reagent. Cells were depleted using small interfering RNAs (siRNAs) shown in Supplementary Table S1 or reported previously (39–41) and harvested 24 or 48 hrs after transfection for RNA and protein lysate preparations. Total RNA was extracted using TRI-reagent (Ambion), treated with DNase I (Promega) and transcribed using the Moloney murine leukaemia virus (MMLV) reverse transcriptase (RT, Promega) and primer d(T)_20_ according to the manufacturers’ recommendations. RT-PCR reactions were performed using minigene- (35E1+PL4 or 35m-amplF) and vector-specific (PL4) primer combinations (Supplementary Table S1). *OGDH* products were also digested with restriction enzymes that cut only one of the two exons. RT-PCR products were separated by gel electrophoresis and their signal intensities were measured as described (43,44).

### Protein purification and mobility shift assays

The WT TIA-1 construct was cloned by inserting BamHI/XhoI amplicons into pET28a, containing His-tags at each end. Recombinant TIA-1 was expressed in BL21 (DE3) pLysS Competent Cells (Promega). The cells were cultivated to OD 0.6 at 37°C and protein expression was induced with 1 mM IPTG at 37°C for 2 h. Bacterial pellets were dissolved in 50 mM Tris–HCl (pH 8.0), 300 mM NaCl, 10% glycerol (w/v) and 3.6 mM β-mercaptoethanol containing cOmplete™, EDTA-free Protease Inhibitor Cocktail (Roche) and were sonicated using SONOPLUS GM Mini20 (Bandelin Electronic). The recombinant protein was purified with Ni^2+^ Sepharose^®^ 6 Fast Flow (GE Healthcare), washed with a buffer containing 50 mM Tris–HCl (pH 8.0), 300 mM NaCl, 10% glycerol (w/v), 3.6 mM β-mercaptoethanol and 20 mM imidazole and eluted using the same buffer upon addition of imidazole to 500 mM. Recombinant WT PUF60 was prepared using a construct cloned by inserting BamHI/XhoI amplicons into pET-28 with a 2xHis-lipoyl-TEV site, as described (45). For long-term storage, both proteins were dialysed against a storage buffer containing a 50 mM potassium phosphate buffer (pH 6.8), 300 mM NaCl, 10% glycerol (w/v) and 3.6 mM β-mercaptoethanol using Slide-A-Lyzer™ G2 dialysis cassettes (ThermoFisher).

For gel shift assays, purified WT proteins were incubated with the indicated oligoribonucleotides (Eurofins) that were end-labeled with [γ-^32^P] ATP and T4 polynucleotide kinase, as described (46). Incubation was performed in a binding buffer (5 mM MgCl_2_, 0.25 μg/μl heparin, 40 mM Tris, pH 8.0, 0.01% Triton and 1 mM DTT) at room temperature for 20 min. RNA-protein complexes were separated on native 6% polyacrylamide gels run in 0.5× TBE at 4°C. Their signal was measured using a Typhoon 9210 PhosphorImager and ImageQuant 8.2 (GE Healthcare). The data were fitted to the Hill equation (*B* = *B*_max_*[*L*^*n*^/(*L^n^* + *K*_d_^*n*^)], where *B*_max_ is a bound fraction of RNA (*B*) at the saturating protein concentration *L* and *n* is the Hill coefficient) to determine dissociation constants (*K*_d_).

### BP mapping

HEK293 cells depleted of the debranching enzyme (DBR1) and control cells were transiently transfected with *OGDH* or control minigenes and harvested after 8–24 h for total RNA extraction. The DBR1 knockdown was achieved with equimolar mixtures of siRNAs shown in Supplementary Table S1 and siRNAs available commercially (Ambion, 16708A). The final concentration of each *DBR1* siRNA was 20 nM. DBR1 converts lariats into linear molecules for degradation by hydrolyzing the 2′-5′ branched bonds (47). Total RNA was extracted using the TRI-reagent and treated with DNase I (Promega). One μg of purified RNA was reverse transcribed with Maxima H Minus RT (ThermoFisher) at 57°C. RT and PCR primers are shown in Supplementary Table S1. Products of the indicated nested reactions amplified 18 cycles at 56°C and 26 cycles at 58°C were subcloned into the pGEM-T-Easy vector (Promega). Plasmid DNA was extracted from randomly selected white *E. coli* colonies using the GeneJET Plasmid Miniprep kit (ThermoFisher). The expected insert size was confirmed using gel electrophoresis following digestion. Insert-containing plasmids were Sanger sequenced (Eurofins) and informative sequences were aligned with Clustal Omega (v.1.2.4).

Human and chicken dBPs were also mapped by RNA sequencing (RNA-seq) of DBR1-deficient HEK293 cells depleted of TIA-1/TIAR or overexpressing PUF60. The cells were transfected with taxon-specific *Ogdh* reporters using jetPRIME (Polyplus). Following total RNA isolation and RT, we employed nested PCRs to obtain lariat products as described above. The products were separated using electrophoresis, extracted using GeneJET Gel Extraction Kit (ThermoFisher) and quantified by NanoDrop. cDNAs were diluted and their concentration and size were confirmed by Agilent 2100 Bioanalyzer (Agilent Technologies). Ampli-seq libraries were prepared using proprietary adapter ligation and amplification (Eurofins Genomics). Ampli-seq was performed using the Illumina NovaSeq 6000 platform in a 150-bp paired end read mode, with a total of 75 716 090 read pairs and a minimum of 5 million reads per sample. Read pre-processing was undertaken using fastp (v.0.20.1) (48); reads were required to have <8 bases with a phred scaled quality of <10. Read pairs were merged into single reads with a minimum overlap of 20 nts and a maximum of 15 mismatches within the overlap. Merged reads shorter than 195 nts were discarded. Reads were aligned to reference sequences (Supplementary Figure S1) using BWA (v.0.7.17) *aln* (49). Alignments allowed a single indel with a gap opening penalty of 11 and extension and mismatch penalties of 4. Aligned reads were quantified with SAMtools (v.1.10). The total number of reads aligned to human and chicken lariat junctions was 32 068 322 and 28 841 414, respectively. Mean read fractions in dBP bins in treated and control cells were compared using unpaired two-tailed *t*-tests with or without the Welch correction. Ampli-seq data were deposited to ArrayExpress (E-MTAB-9412).

### Expression of endogeneous *Ogdh* isoforms

RNA samples from human tissues were purchased from Ambion (The FirstChoice human total RNA survey panel, AM6000, ThermoFisher). Animal tissues were obtained from *Xenopus leavis*, *Coturnix japonica* and *Rattus norvegicus*, as approved by the Animal/Ethics Committees of the Czech Academy of Sciences (approval 66866/2015/MZE-17214), the Slovak Academy of Sciences (Ro-1464/19-221/3) and Veterinary and Food Administration (C.k. Ro 3123/17-221; SK UCH 01017) according to the Directive 2010/63/EU of the European Parliament. Animal handling followed the EU legislation for animal research. Tissue samples from marsupials/monotreme were obtained from *Monodelphis domestica* (opossum), *Tachyglossus aculeatus* (echidna) and *Ornithorhynchus anatinus* (platypus) under the AEEC approval R.CG.07.03, Environment ACT permit LI 2002 270, National Parks and Wildlife Service permit A193, AEC permit S-49-2006 and University of Melbourne permits ID1111998.2 and ID1814535.2. Tissue samples were snap frozen and total RNA was extracted using TRI-reagent (Ambion). RT was with SuperScript IV (Invitrogen) and a mixture of hexamer and oligo-d(T)_20_ for 1 h at 53°C. For PCR amplification, we used taxon-specific RT-PCR primers in Supplementary Table S1.

### RNA structural probing and secondary structure predictions across dBPs

Transcription templates were prepared with and without linker sequences, which allow the RT to become fully processive prior to reaching the region of structural interest and prevent non-templated primer extension products from masking structural information (50). Apart from linkers, PCR primers incorporated a T7 promoter sequence (Supplementary Table S1). Structural probing was carried out using dimethyl sulfate (DMS) (51) and selective 2′-hydroxyl acylation analyzed by primer extension (SHAPE) (50). SHAPE was performed with 2-methylnicotinic acid imidazolide (NAI) essentially as described (52,53). DMS methylates the N1 of adenine and the N3 of cytosine on the Watson–Crick base-pairing face of unstructured regions, whereas SHAPE reagents acylate the 2′-hydroxyl group on the ribose sugar of all four nucleotides, albeit with different efficiencies. DMS and NAI adducts were detected by RT-mediated primer extension that is prevented by modified nucleotides.

Templates were transcribed using MEGAscript™ T7 Transcription Kit (Invitrogen) according manufacturer's recommendations. T7 transcripts were purified using the TRI-reagent and quantified with UV spectroscopy. Their integrity was confirmed on a 8.3 M urea/10% polyacrylamide gels. RNA probes (10 pmol each) were mixed with a SHAPE reaction buffer to a final concentration of 100 mM KCl, 40 mM HEPES (pH 7.5) and 0.5-5 mM MgCl_2_ and incubated at 37°C for 45 min. DMS was added to a 100 mM at 37°C for 5 min. NAI was added to a final concentration of 50 mM or 100 mM at 37°C for 5 min. The reactions were quenched with a freshly prepared dithiothreitol (DTT) at 0.1 M. RNAs were purified and concentrated using the RNA Clean&Concentrator™-5 (Zymo Research) according to the manufacturer's protocol and eluted in RNase-free water. Purified RNAs (6 μl) were mixed with 1 μl of 5 μM Cy5-labeled universal primer (Supplementary Table S1) and heated at 75°C for 3 min, which was followed by adding 2 μl of 5× RT buffer (final concentration of 50 mM Tris–HCl, pH 8.3, 75 mM KCl, 3 mM MgCl_2_, 1 mM DTT). The samples were left at 35°C for 5 min prior to addition of 1 μl (200 U) of SuperScript III (Invitrogen). RT reactions were incubated at 50°C for 15 min, followed by the addition of 0.5 μl of 2 M NaOH and heating at 95°C for 15 min to degrade RNA and denature RT. Each reaction was mixed with an equal volume of stop solution (20 mM Tris, pH 7.5, 20 mM EDTA, Orange G and deionized 95% formamide), heated at 95°C for 5 min, loaded onto 8% gels with 8.3 M urea and size-fractionated at 55 W. Gel images were collected with a Typhoon 9210 PhosphorImager and quantified using ImageQuant 8.2 (GE Healthcare).

Reactivity profiles were generated by subtracting the intensity of normalized modified RNA peaks from intensities of no-reagent control peaks from two replicas, as described for NAI (54) and DMS (51). For SHAPE-guided RNA secondary structure predictions, we employed methods applying pseudo-energies to stacked pairs (55) or all discordant positions (56) as well as a method incorporating dynamic generation of perturbation energies (57), as implemented in RNAstructure (58) and the Vienna RNA Package (59).

### Auxilliary splicing elements and metal binding sites

ESRseq scores of 4096 splicing regulatory hexamers were derived previously by exonic splicing enhancement (ESE) or silencing (ESS) *ex vivo* afforded by random hexamers placed at five different positions into two model exons (60). ESRseq scores provide comprehensive estimates of the ESE and ESS hexamer strength (60). The ESE/ESS ratios were computed for each codon and compared with frequencies of amino acids that contribute critically to protein binding sites for metal ions. We employed residue frequencies derived from fragment transformation methods, which were associated with high (∼95%) accuracy by considering only amino acids within 3.5 Å from the metal ion centre (61). In addition, we obtained residue frequencies from MetalPDB (62) that were based on ∼290 000 metal binding sites from >50 000 macromolecular structures. Codons were ranked by ESE/ESS counts/ratios and ESRseq scores. Apart from ESE/ESS metrics (60), we employed hexamer preference indices previously computed for independent ESE sets (63). The hexamer preference index captures a difference between frequencies of an amino acid in tested and randomized ESE sets normalized by SDs (64). Tested sets were normalized for relative abundances in codon usage after removing stop codons (64). High index values imply a codon enriched in the ESE datasets as compared to its usage in the genome (63).

### Additional bioinformatic analyses and resources

Genomic evolutionary rate profiling RS scores, which provide position-specific estimates of evolutionary constraints using maximum likelihood evolutionary rates, were computed by GERP++, with gaps treated as missing data (65). RS score is the number of substitutions expected under neutrality minus the number of substitutions observed at the indicated intron positions, with positive scores pointing to substitution deficits and evolutionary constraints (65). PhyloP scores for 100 vertebrates species were obtained from UCSC. Gene ontology analysis was carried out with WebGestalt (66) and PANTHER (67). Classification of *OGDH/OGDHL* TEs and MIR subfamilies was confirmed with RepeatMasker (42,68). PU values (probability of unpaired) were computed as described (69). MitoMiner (v.4.0) (70) was interrogated with a set of 219 human genes regulated by PUF60 at the exon level (41). RNA-seq data for cells depleted of PUF60 (41) are available from the ArrayExpress (accession number E-MTAB-6010).

## RESULTS

### Identification of proteins that reciprocally control mutually exclusive *OGDH* splicing

Genomic alignments of *OGDH* orthologues (Supplementary Figure S1) revealed that intron 4a, which separates the MXEs, was shortened from >500 nts in early chordates to a narrow size range of 118–131 nts in birds and mammals (Figure 1B,C). The restricted range first evolved in reptiles and was rigorously maintained in endotherms, except for platypus (Figure 1B, C), despite massive intron expansions in higher vertebrates (37). Genomic evolutionary rate profiling revealed that in mammals intron 4a diverged to a similar extent as flanking exons while Phylop100 confirmed its high conservation in vertebrates (Figure 1D). The upstream intron also remained small, with only ∼5% of TEs; by contrast, the upstream intron in the *OGDH* paralogue *OGDHL* sustained multiple TE insertions, totalling to ∼70% of its length (Figure 1E). The intron size restrictions suggest that the tissue-specific regulation of *OGDH* MXEs requires a tight control by flanking introns.

We set out to explore why *OGDH* intron 4a expansion was prevented throughout vertebrate evolution. The intron has a very long polypyrimidine tract (PPT, Figure 1A and Supplementary Figure S1), a critical 3′ss recognition sequence that interacts with pyrimidine (Y)-binding proteins to facilitate recruitment of the U2 small nuclear ribonucleoprotein (snRNP) to BP (41,71–74). To test their role in exon 4a/4b regulation, we examined splicing of the human *OGDH* reporter in HEK293 cells individually overexpressing a battery of Y-binding proteins, including U2AF65 as a major PPT recognizer (75,76). Most of these proteins were previously shown to recruit components of U1 and U2 snRNPs, which assemble at 5′ss and BPs, respectively, typically in this order (73,77–82). The WT *OGDH* reporter produced a mixture of 4a+ and 4b+ isoforms but no transcripts with (4a+4b+) or without (4a–4b–) both exons (Figure 1F), recapitulating *in vivo* splicing in some viscera (33). Isoform 4b+ was promoted by PUF60 and U2AF65 and was repressed by TIA-1 whereas PTBP1 inhibited inclusion of both MXEs in mature transcripts (Figure 1F). Overexpression of RBM20, which is facilitated by a zinc-finger domain deletion (83), also led to exon repression and skipping, largely at the expense of exon 4b (construct RBM20-Δ1, Figure 1G). RBM39 (CAPERα), a tentative human homolog of *Schizosaccharomyces pombe* rsd1 that bridges U1 and U2 interactions (84), activated isoform 4a+ (Figure 1H). Knockdown of PUF60, RBM39 and both TIA proteins had the opposite effects than their overexpression (Figure 1I, J and further below; (41)). In contrast, both the knockdown and overexpression of U2AF65 promoted exon 4b (Figure 1F, I). U2AF65 overexpression also increased skipping of this MXE pair (Figure 1F), as was observed for cassette exons in other transcripts (Supplementary Figure S2) (85).

Together, these results showed that altering the abundance of U-binding RBPs can reciprocally modulate *OGDH* MXEs, with TIA-1/TIAR and RBM39 activating the 5′ss of intron 4a and PUF60 promoting its 3′ss. This activation is likely to involve their known interactions with U1 and U2 snRNP components, respectively, namely TIA proteins with U1-C (79), RBM39 with U1-A (84) and PUF60 with SF3B1 (74).

### Binding of reciprocal regulators of *OGDH* MXEs to the pre-mRNA

**Figure 2.**
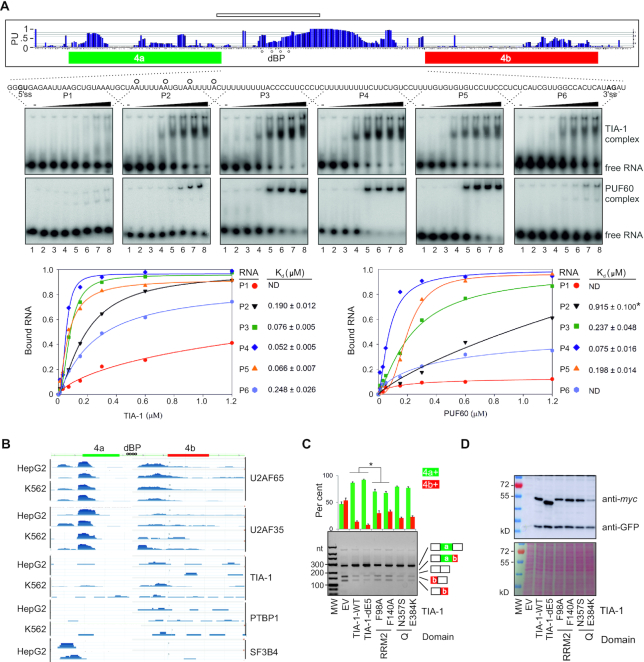
MegaPPT as a binding platform for splicing factors reciprocally activating or inhibiting *OGDH* MXEs. (**A**) Preferential binding of TIA-1 and PUF60 to single-stranded megaPPTs in intron 4a *in vitro*. PU values (probability of unpaired) (69) are shown in the *upper panel*. Higher PU values predict unpaired, more accessible RNA bases (69). Human dBPs identified in Figure 3 are denoted by circles. RNA used for structural probing is denoted by a horizontal rectangle at the *top*. EMSA (*middle panels*) were carried out with 1 nM of oligoribonucleotides P1-P6 and zero, 0.01, 0.03, 0.07, 0.15, 0.3, 0.6 and 1.2 μM of each recombinant protein (lanes 1–8). *Lower panels* show graphical representations of bound fractions. TIA-1 *K*_d_ for probes P1–P6 inversely correlated with their uridine fractions (*r* = –0.85, *P* = 0.03, *F*-test). ND, not determined; *, derived from the linear equation. (**B**) Compilation of a subset of enhanced crosslinking and immunoprecipitation datasets (86) for the indicated cell lines (*left*) and proteins (*right*). (**C**) TIA-1 RRM2 substitutions of residues that contact RNA impair TIA-1-mediated activation of exon 4a. RNA products were treated with PvuII. Restriction fragments are shown in Figure 1G. Asterisk denotes *P* values <0.05 (ANOVA, Dunnett's post-hoc tests). Error bars are SDs of two independent replicates. (**D**) Immunoblots for the transfection experiment shown in panel **C**. Protein lysates (15 μg) visualized by Poncaeau staining (*lower panel*) were incubated with anti-GFP and anti-*myc* antibodies (*upper panel*).

The U-rich sequences in intron 4a showed high PU values (Figure 2A, *upper panel*), which predict single-stranded interactions of splicing regulatory motifs (69). These megaPPTs were preferentially bound by WT TIA-1 and PUF60 in EMSA assays with overlapping oligoribonucleotides spanning intron 4a, as compared with sequences upstream of potential BPs (Figure 2A*, lower panel*). Compilation of enhanced crosslinking and immunoprecipitation datasets (eCLIP) (86) showed that this MXE region bound TIA-1 as well as U2AF and PTBP1 while eCLIP signals across predicted BPs were absent (Figure 2B). Both U2AF65 and U2AF35 bind strongly to the 3′ss of exon 4a, which carries a proposed signature motif (ttncag) for U2AF35b (87), consistent with a strong dependency of exon 4a on both U2AF subunits (41).

TIA-1 binding to U-rich sequences is largely mediated by the second RNA recognition motif (RRM2) while the noncanonical RRM1 and the C-terminal glutamine-rich domain are required for association with the U1 snRNP through U1-C (79,88). Mutations of conserved aromatic residues F98A and F140A in RNP1 and RNP2 motifs of RRM2, which interact with uridines through base stacking interactions (89), reduced the TIA-1-induced promotion of exon 4a in transient cotransfection assays (Figure 2C,D), consistent with their diminished binding to a U_20_ oligo (90). We did not find a significant decrease in exon 4a inclusion with disease-associated TIA-1 substitutions N357S and E384K in the glutamine-rich domain. N357S was reported in late-onset distal myopathy involving gastrocnemius/soleus muscles (91), flexors essential for locomotion and balance. E384K was associated with Welander distal myopathy, which is manifested by weakness and atrophy of distal muscles (92,93). Finally, substitution H169Y in PUF60 RRM1 previously diminished the PUF60-induced increase in exon 4b inclusion (41). Taken together, reciprocal regulation of *OGDH* MXEs by PUF60 and TIA-1 involves their binding to intron 4a megaPPTs by their RRMs.

### Expression of Ca^2+^-sensitive OGDH isoform is enforced by distant lariat branch sites

**Figure 3.**
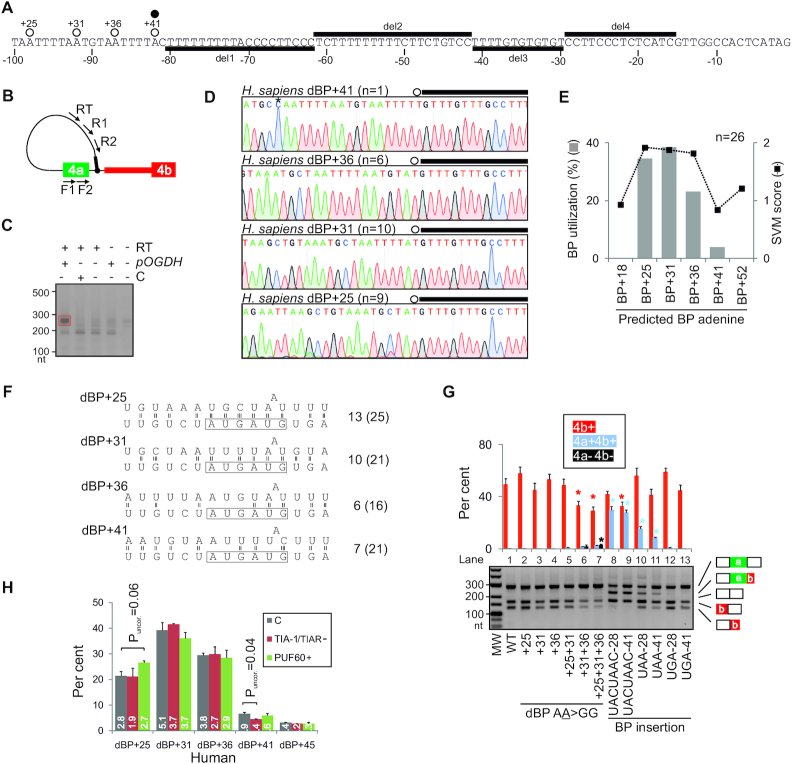
*OGDH* transcripts employ redundant dBP adenines located near the 5′ss of exon 4a. (**A**) The last 100 nts of intron 4a. Newly mapped dBPs (open circles) are denoted by their distance from the 5′ss of intron 4a. A closed circle shows a low-confidence BP reported previously (97). Horizontal bars denote megaPPT deletions (del1–4) introduced in the WT *OGDH* reporter (tested in Supplementary Figure S5). (**B**) BP mapping primers (arrows). Their sequences are in Supplementary Table S1. Black bar denotes the 5′ end of the first minigene intron. (**C**) PCR products from nested reactions. *pOGDH*, the *OGDH* plasmid; RT, reverse transcriptase; C, a control minigene. Red rectangle denotes fragments extracted from the gel for cloning. (**D**) Representative sequence chromatograms illustrating diagnostic A>T mismatches at each lariat junction. The 5′ end of minigene intron is denoted by black rectangles and BPs are circled; *n*, the number of informative clones supporting each dBP; their alignments are shown in Supplementary Figure S3. Asterisk in the dBP+41 panel denotes a PCR-generated T>C mutation. (**E**) dBP usage (*left*) and their SVM scores (*right*). (**F**) Predicted U2 snRNA:dBP base-pairing interactions. Only the most common mode of BP:U2 contacts (193) is shown. The BP-interacting region of U2 snRNA is boxed; number of hydrogen bonds within the U2 box is to the right. The number of hydrogen bonds within the extended region, which may facilitate base shifted registers and less common binding modes (39,193,194), is in parentheses. (**G**) Impact of dBP substitutions (lanes 2–7) and insertions of canonical BPs (lanes 8–13) on splicing. RNA products were digested with PvuII, as shown in Figure 1G. Asterisks show significant changes from the WT. (**H**) dBP usage in cells depleted of TIA proteins or overexpressing PUF60, as determined by RNA-seq. Bar charts represent the mean relative usage (%) of each dBP; error bars are SDs of technical duplicates. C, untreated cells. The number of reads (in millions) supporting each dBP bin is shown in white.

Longer PPTs tend to be associated with BPs that are further upstream of their typical location near 3′ss, known as distant BPs or dBPs (94–96). Transcriptome-wide studies employing exoribonuclease digestions and/or targeted RNA-seq did not report any high-confidence BPs in *OGDH* intron 4a, nor were any intron 4a BPs found by analyzing ∼1.31 trillion reads from 17,164 RNA-seq data sets, possibly because of very poor BP detection rate in such short introns (97,98). To identify the BP(s), we extracted total RNA from HEK293 cells transfected with *OGDH* and control minigenes. RNA samples were reverse transcribed across the lariat junction using an intron-specific RT primer and amplified with nested primers (Figure 3A-C). Sanger sequencing of lariat products revealed four closely spaced BP adenines (A; Supplementary Figure S3A). Three BPs were located 25, 31 and 36 nts downstream of the 5′ss in the optimal UAA motifs and were used at 23–38% (Figure 3D and Supplementary Figure S3A). The fourth BP (41 nts) was supported by a single clone and was in the suboptimal UUA context. This motif slightly reduced splicing of independent introns with solitary UGA or UAA BPs (Supplementary Figure S4), consistent with a low uridine representation at position -1 of established BPs (99). The dBP usage significantly correlated with their positive support vector machine (SVM) scores (*r* = 0.92, *P* = 0.02; Figure 3E), in line with the improved accuracy of BP prediction that takes into account PPTs (96). Assuming canonical base-pairing and bulged A at each dBP, the predicted strength of dBP:U2 snRNA interactions was greater for the two upstream dBPs, which appeared to be preferentially used (Figure 3D–F).

To support the dBP assignments further, we individually mutated each of the three high-usage dBPs to guanine (Figure 3G) since A>G substitutions of solitary BPs caused more severe splicing defects in genetic disease than other substitutions (100). Position –1 relative to each dBP A was mutated to the same residue to prevent the use of adjacent adenines as BPs. Individual UAA>UGG mutations yielded no or only minor alterations in isoform 4a+/4b+ ratios (lanes 2–4), consistent with a compensatory role of the remaining dBPs. However, a double mutation reduced exon 4b inclusion in the mRNA (lanes 6) while the triple mutation diminished it further and generated small amounts of unproductive transcripts 4a+4b+ and 4a–4b– (lane 7).

To test if the usage of individual dBPs in the cluster is influenced by U-binding RBPs that reciprocally regulate exon 4a/4b ratios (Figure 1F–J) and bind the megaPPT (Figure 2A, B), we determined the dBP distribution in HEK293 control cells, cells lacking both TIA proteins and cells overexpressing PUF60. These cells generate almost exclusively isoform 4b+ (Figure 1F and below), thus maximizing the lariat yield. To increase the lariat detection rate further, each group was also depleted of DBR1. The DBR1 depletion alone altered neither the 4a+/4b+ ratio (Supplementary Figure S3B) nor the dBP usage hierarchy (cf. Figure 3E and Supplementary Figure S3C, D). Cells depleted of TIA proteins or overexpressing PUF60 showed a similar dBP distribution as controls (Table 1). To increase the power of this comparison further, we employed RNA-seq of lariat junctions in an independent experiment involving a total of 32 068 322 reads, confirming the same usage of individual dBPs as in controls (Figure 3H) and a significant correlation with SVM-BP scores (*r* = 0.86, *P* = 0.03). The read alignments also showed a putative low-usage dBP uridine downstream of the cluster (termed dBP+45) (Figure 3H), potentially extending the dBP choice further. Finally, BP mapping in control cells, which produce a mixture of 4a+ and 4b+ transcripts (Figure 1F), revealed that the BP used by isoform 4a+ is 27 or 28 nts upstream of the 3′ss of exon 4a (Supplementary Figure S3F).

**Table 1.** Usage of individual dBPs in human intron 4a

*DBR1*-deficient cells	Number of Sanger-sequenced clones	Number of informative clones (%)	dBP+25	dBP+31	dBP+36	dBP+41
Lacking TIA-1 and TIAR	55	39 (71)	14	12	10	3
Overexpressing PUF60	52	43 (83)	18	15	10	0
Controls	57	50 (88)	14	23	12	1
Total	164	132 (80)	46	50	32	4

χ^2^ for 4 × 3 (with dBP+41) and 3 × 3 (without dBP+41) contingency tables was 7.1 (d.f. = 6; *P* = 0.3) and 3.6 (d.f. = 4; *P* = 0.5), respectively. Aligned sequences of informative clones are in Supplementary Figure S3D.

We conclude that the MXE pair in *OGDH* is spliced via canonical (exon 4a) and non-canonical or distant (exon 4b) BPs. All dBPs are located below a previously proposed steric 5′ss-BP threshold of ∼50 nts (34–36). The relative usage of apparently redundant dBPs was insensitive to reciprocal *OGDH* MXE regulators TIA-1/TIAR or PUF60.

### Transposons can both introduce and eliminate efficient dBP competitors

We reasoned that the intron 4a size restriction could be imposed not only by a 5′ss-dBP limit but also by the dBP-3′ss distance. The latter distance is larger (Figure 2A) and perhaps more likely to accommodate random insertions that might introduce new BPs at their typical location 19–37 nts from 3′ss, the home of ∼90% of human BPs (97). To begin to explore competition between dBPs and canonical BPs upon intron 4a expansion, we first introduced three BP motifs at two positions (−28 and −41 nts) upstream of the 3′ss (Figure 3G, lanes 8–13). The first motif was a *Saccharomyces cerevisiae* BP (UACUAAC), which is also a preferred human BP (101), whereas the remaining two had degenerate mammalian BP consensus UAA or UGA (99). The yeast BP generated the highest relative abundance of transcripts 4a+4b+ at each position (lanes 8–9), *ie*. intron 4a splicing. Insertions of UAA, but not UGA, at either position induced intron 4a splicing to a lesser extent (Figure 3G, lanes 8–13). In contrast to *OGDH*, the two mammalian URA motifs were functionally identical in independent introns with solitary BPs (Supplementary Figure S4). These data suggested that the reporter should be informative for examining potential dBP competitors introduced by TEs.

**Figure 4.**
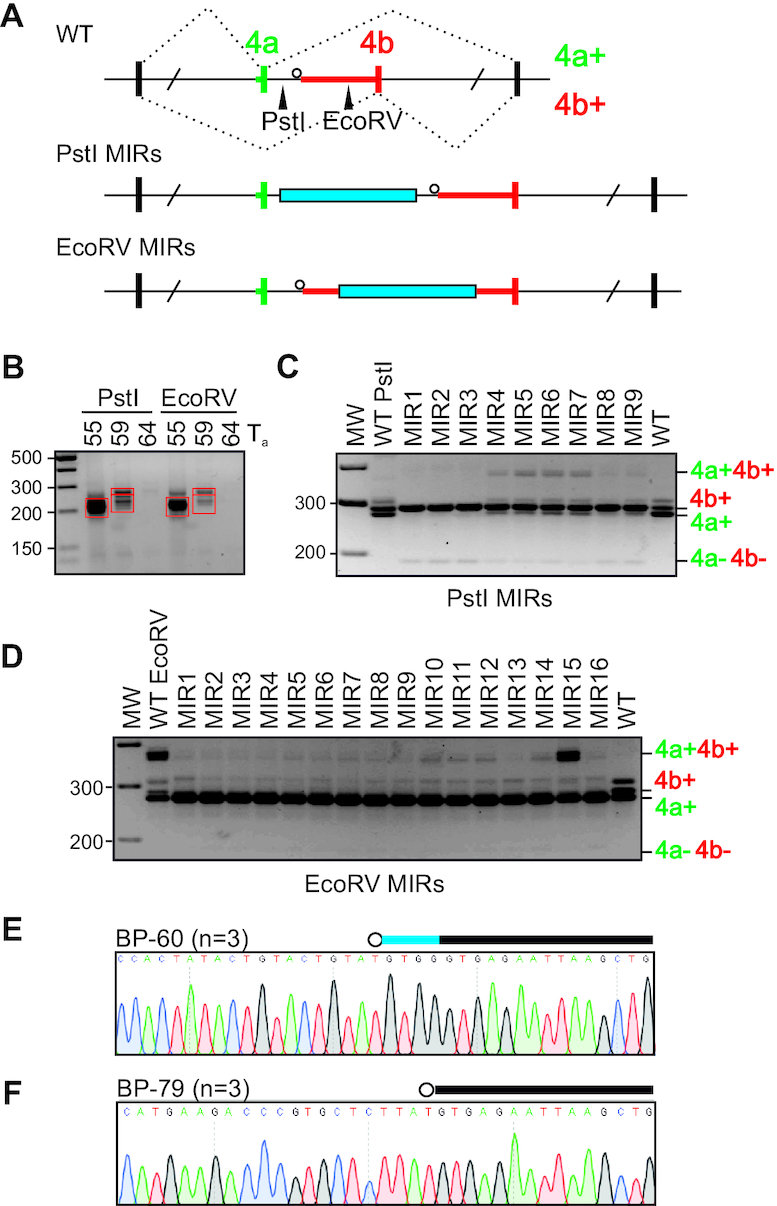
Transposons can both introduce and eliminate dBP competitors. (**A**) Schematics of MIR cloning. MIR insertions (blue bars) were introduced in intron 4a upstream (PstI) or downstream (EcoRV) of the dBP cluster. Restriction sites are denoted by arrowheads in the WT construct. (**B**) Construction of the MIR library. PCR products obtained with the indicated annealing temperatures (*T*_a;_°C) were cut from the gel (red rectangles) and subcloned into PstI or EcoRV sites. (**C, D**) Exon usage of *OGDH* constructs with upstream (**C**) or downstream (**D**) MIR insertions. MIR inserts are characterized in Supplementary Table S3. (**E, F**) MIR15 transcripts employed both a unique BP (**E**) and BP shared with other MIR constructs (**F**). Canonical BPs are denoted by their distances from 3′ss (in nts). Blue horizontal bar denotes the last 4 nts of exon 4a.

Next, we determined the frequency of BP-like URA motifs in short interspersed elements (SINEs). This TE family is overrepresented in mutation-induced cryptic exons that resulted in genetic disease and may supply new BPs (102). The most abundant SINE representatives, *Alu* elements (103), had on average one such motif per ∼30 nts (∼3%) in their consensus sequences and the motif densities were even higher in MIRs (Supplementary Table S2). We then cloned a human MIR library into restriction sites introduced between *OGDH* dBPs and 5′ss or 3′ss and examined splicing of MIR-containing reporters upon transfection into HEK293 cells (Figure 4A-D and Supplementary Table S3). Constructs with MIRs upstream of dBPs invariably diminished exon 4a inclusion. The inhibition was stronger for sense than antisense MIRs; the latter also induced isoform 4a+4b+ (MIR4-7, Figure 4C and Supplementary Table S3). Antisense MIR insertions had more URA motifs in 50-nt segments downstream of the 5′ss than sense insertions (on average, 2.3 versus 0.5), but we were unable to obtain any lariat junctions for PstI clones. The URA motifs may not be recognized as new dBPs because their adjacent downstream sequences lack U-rich PPTs and contain AG dinucleotides (Supplementary Table S3), violating the AG dinucleotide exclusion zone (AGEZ) at 3′ss. New AGs introduced in AGEZs are selected by the splicing machinery as 3′ss if located >∼8–12 nts downstream of BPs (96,97,104,105), however, no cryptic 3′ss were detected.

In contrast to MIR insertions upstream of dBPs, transcripts with MIRs downstream of dBPs repressed exon 4b (Figure 4D). The EcoRV site insertion alone introduced a TGA motif 60 nts from the 3′ss, which was used as the new BP (Figure 4E and Supplementary Table S3). The new BP-60 permitted efficient intron 4a splicing, which was repressed by all MIR insertions, except for MIR15 (Figure 4D). Uniquely, MIR15 maintained uridine at position –2 relative to the BP-60A and contained additional BP motifs that were absent in the remaining MIR constructs (Supplementary Table S3). BP mapping of MIR15 transcripts confirmed the use of BP-60 and revealed utilization of additional BPs upstream, including UAA-79, a motif shared with clones MIR10-16 (Figure 4D, F and Supplementary Table S3). Finally, the use of canonical BP competitors in MIR15 induced an alternative 5′ss activated 4 nts upstream of the authentic 5′ss of intron 4a (Figure 4E). We conclude that TEs can both introduce and eliminate dBP competitors, which can affect splice site choice. TEs inserted upstream or downstream of *OGDH* intron 4a dBPs consistently impaired the MXE pattern, in line with purifying selection for short intron size over hundreds of Myrs of vertebrate evolution (Figure 1B, C). This process can be influenced by DNA variability within a single TE subfamily, TE orientation and distance from splice sites.

### Identification of natural *OGDH* variants that alter MXE ratios

**Figure 5.**
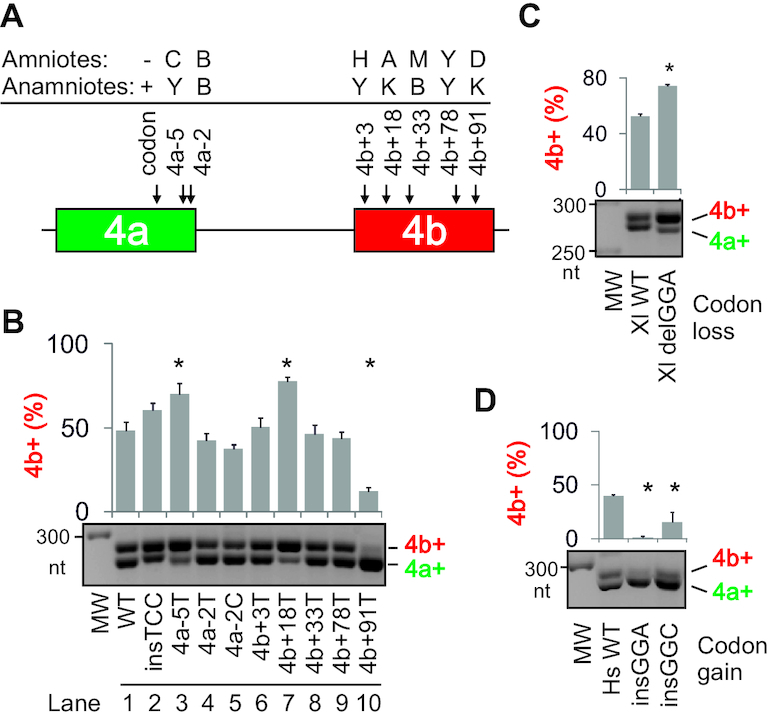
Identification of vertebrate *Ogdh* variants that alter MXE ratios. (**A**) Summary of tested variants. Each polymorphism is represented by a IUPAC code (B = C, G or T; H = A, C or T; K = G or T; M = A or C; D = A, G or T) and is denoted by nt distances from the 5′ss (−) or 3′ss (+) of intron 4a. Supplementary Figure S1 shows each allele in 62 vertebrates. (**B**) The relative abundance of Ca^2+^-sensitive isoforms 4b+ (*upper panel*) following transfections of mutated constructs into HEK293 cells (*lower panel*). Error bars are SDs of two transfection experiments. Asterisks denote significant (*P*< 0.05; one-way ANOVA with Dunnett's post-hoc tests) differences in exon 4b inclusion from the wild-type construct (WT). (C, D) The impact of codon loss (**C**) or gain (**D**) in exon 4a on MXE ratios. (C) Loss of GGA codon from *Xenopus laevis* transcripts promotes isoform 4b+. (D) Codon gain in *Homo sapiens* transcripts promotes isoform 4a+.

The dBP cluster is adjacent to a ∼65-nt megaPPT, which evolved into UC-, UU- and UG-rich segments in mammals (Figure 3A and Supplementary Figure S1). Removal of these segments from human transcripts revealed only minor alterations of exon 4a/4b ratios for outer deletions (Supplementary Figure S5). To identify exon 4a/4b variants that affected the MXE pattern and were selected during evolution, we ‘dehumanized’ phylogenetic positions in our splicing reporter (Figure 5A and Supplementary Figure S1). Transfections of mutated constructs revealed that exon 4a C>T substitution 5 nts upstream of the 5′ss (lane 3) as well as the exon 4b A>T substitution 18 nts downstream of the 3′ss (lane 7), both present in amphibians and a subset of fish, significantly promoted exon 4b (Figure 5B). Substitution A>T 13 nts upstream of the 5′ss of exon 4b, present in fish, amphibians and turtles, diminished exon 4b inclusion (lane 10), indicating that, apart from platypus, the adenine allele has been important for maintaining the Ca^2+^-sensitive isoforms in endotherms. By contrast, individual mutations designed to test apparent relaxation of 5′ss and 3′ss in placentals (Supplementary Table S4) had no or only minor effect (lanes 4–6).

Exon 4a orthologues in amniotes lost a single codon (GRN) from their anamniotic ancestors (Supplementary Figure S1). Removal of the GGA codon from frog transcripts, but not the same codon in the opposite orientation, promoted isoform 4b+ (Figure 5B, lane 2, and 5C). Conversely, the insertion of frog (GGA) or fish (GGC) codons at the same position of the human reporter enhanced isoform 4a+ (Figure 5D).

Together, these results revealed MXE variants that were selected during vertebrate evolution and promoted or repressed Ca^2+^-sensitive *Ogdh* isoforms. Although the megaPPT provides an important platform for fine-regulation of tissue-specific 4a/4b ratios, it is also robust enough to tolerate smaller deletions without diminishing inclusion of either MXE. This finding would explain why such changes contributed most to the shortening of intron 4a in higher vertebrates (Figure 1B,C and Supplementary Figure S1).

### Conserved regulation of vertebrate *Ogdh* MXEs by U-binding proteins

*Ogdh* reporters derived from representative vertebrate taxa produced correctly spliced products in human and rat cells (Supplementary Figure S6A). In these cells, primary transcripts from endotherms failed to generate unproductive isoforms 4a+4b+ or 4a–4b–, unlike those derived from ectotherms (fish and, to a lesser extent, frog). The relative abundance of chicken isoform 4a+ was diminished as compared to other tested species, which was recapitulated by varying the length of the flanking intron and with a pre-mRNA derived from another bird species (Supplementary Figure S6B). Coexpression of human U-binding factors and taxon-specific *Ogdh* reporters in HEK293 cells (Supplementary Figure S6C) showed that PUF60 promoted exon 4b in all tested taxa whereas TIA-1 promoted exon 4a only in amniotes and amphibians, although knockdown of both TIA proteins activated exon 4b in fish-derived transcripts as well (Supplementary Figure S6D, E). Speculatively, this could reflect more diverged TIA-1 RRM domains between fish and humans as compared to PUF60 counterparts (Supplementary Table S5). With chicken *Ogdh*, TIA-1 overexpression as well as PUF60 knockdown were sufficient to activate isoform 4a+ (Supplementary Figure S6B, C and further below). Importantly, examination of nucleotide composition in vertebrate megaPPTs in intron 4a revealed that the U as well as the combined guanine and U content were significantly increased in mammals at the expense of cytosine (Supplementary Figure S7A). The opposite trend was observed for large PPTs downstream of dBPs previously determined in muscle genes encoding tropomyosin and actinin (Supplementary Figure S7B,C), which are regulated by Ca^2+^ (35,36). Taken together, splicing of *Ogdh* MXEs derived from amniotes was more efficient in mammalian cells than those derived from their anamniotic counterparts. The observed transitions in nt composition of vertebrate megaPPTs are likely to alter the repertoire of interacting RBPs.

### Tissue-specific usage of alternative *OGDH* exons

At the gene level, *OGDH* is widely expressed across tissues, with the highest expression in the left heart ventricle and skeletal muscles (Supplementary Figure S8). At the exon level, human exon 4a is preferentially spliced to alternative exon 5 in the brain (Supplementary Figure S9A), as in the mouse (33). However, the association of exons 4a and 5 is not absolute since isoform 4b+5+ was detected in striated muscles (Supplementary Figures S8 and S9A, B). Examination of endogenous transcripts from representative vertebrate species showed that exon 5 inclusion in the mRNA was highest in the brain from amphibians to humans (Supplementary Figure S9B-E). Our attempts to see a brain-specific exon 5 inclusion in zebrafish tissues from independent animals were unsuccessful although we could not exclude target misannotation. Exon 5 splicing in tissues extracted from a monotreme and marsupials was similar to other mammals (Supplementary Figure S9F). Inspection of RNA-seq data from mouse brain cell subpopulations (106) showed that the inclusion of exon 5 in the mRNA was specific to neurons, with trace levels in myelinating oligodendrocytes (MOs), but was absent in other cell types, including astrocytes (Supplementary Figure S10). The relative abundance of exon 4a in neurons was higher than that of exon 4b and still substantive in MOs, but was low in major glia. Transfection of a longer human reporter containing exons 4a, 4b and 5 into HEK293 cells showed correctly spliced isoforms 4a+5+ and 4b+5+; exon 5 inclusion was reduced upon coexpression with U2AF65 and PTBP1 and increased in cells overexpressing TIA-1. This demonstrates responsiveness of exon 5 to MXE regulators (Supplementary Figure S9G and 1F), including endogenous U2AF35 (41). Finally, exon 5-containing isoforms in frog *Ogdhl* transcripts were entirely absent (Supplementary Figure S9E, *lower panel*, and S11), in contrast to *Ogdh*.

We conclude that (i) the dBP-promoted and Ca^2+^-activated isoform 4b+ has been a predominant contributor to the *OGDH* gene-level expression in most vertebrate tissues, particularly in striated muscles; (ii) the Ca^2+^-insensitive isoform 4a+ lacking exon 5 is expressed at low levels, mainly in intestines and testis; (iii) exon 5 is spliced preferentially to exon 4a in neurons of multiple vertebrate species, indicating that neurons contain/lack a conserved brain-specific activator/repressor of transcripts 4a+5+ and that this factor has been active for at least 250 Myr.

### Evolution of the *OGDH* dBP cluster in vertebrates

Although *OGDH* dBPs are conserved in amniotes, their extended motifs are not identical (Supplementary Figure S1). This variability may influence their interactions with *trans*-acting factors, contribute to taxon-specific usage of individual dBPs and affect 4a+/4b+ ratios since substitutions at positions other than 0 or -2 relative to BP A can alter exon inclusion (Supplementary Figure S4) (43,101,107). The consensus motif of dBP+36 remained least variable during evolution but the remaining dBP motifs sustained potentially functional mutations (Supplementary Figure S1). For example, endothermic *Eutheria* gained a 2-nt insertion that optimized the first two positions of BP+25 as compared to *Sauropsida* or *Metatheria*/*Monotremata* (Supplementary Figure S1). In addition, mammals as well as some birds and reptiles carry optimal UAAs at dBP+31, but a subset of sauropsids and platypus evolved a GAA motif instead (Supplementary Figure S1). The GAA motif is highly repressive (Supplementary Figure S4) (43,99,100). The less efficient dBP+31 could be potentially compensated by an optimized sauropsid orthologue of dBP+41 (UUA>UAA, Supplementary Figure S1). Finally, in contrast to amniotes, frogs carry only one UAA motif close to the 5′ss, which might constitute an ancestral dBP.

**Figure 6.**
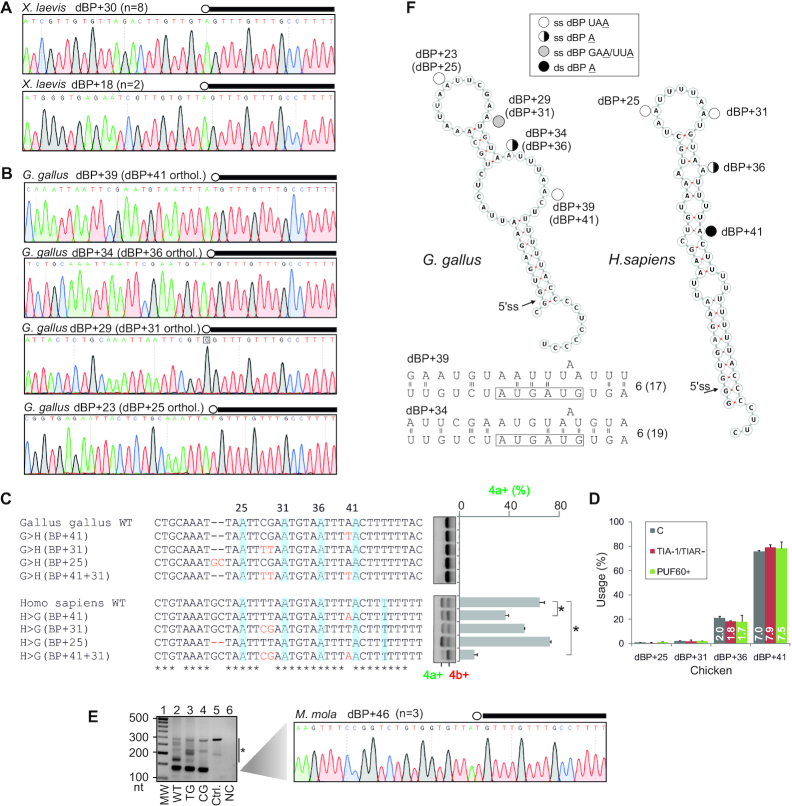
Evolution of the *Ogdh*dBP cluster in vertebrates. (**A, B**) BP mapping in frog (**A**) and chicken (**B**) transcripts. dBPs are circled and denoted by their distance from the 5′ss. The 5′ end of minigene intron is denoted by black rectangles. (**C**) The impact of sauropsid dBP variants on exon 4a/4b usage. Mutations introduced in the WT minigenes are in red. dBPs are shaded. Error bars are SDs of two transfections. Asterisks denote significant differences between means (P<0.05; ANOVA with post-hoc Dunnett's tests). (**D**) Chicken dBP usage in HEK293 cells depleted of TIA-1 or overexpressing PUF60. Bar charts show mean usage of the indicated dBPs; error bars are SDs of two transfection wells. The total number of RNA-seq reads (in millions) is in white. C, untreated cells. (**E**) BP mapping in ocean sunfish (*M. mola*) transcripts. RT-PCR gel (*left panel*) with a 140-nt fragment containing a lariat junction sequenced in the *right panel*. Ctrl., control plasmid, NC, negative RT control. TG and CG denote *M. mola* minigenes with mutations removing the AGEZ spoiler and restoring the long AGEZ (Supplementary Figure S13E). Asterisk denotes RT-PCR artefacts confirmed by sequencing. (**F**) Potential display of dBPs in stable RNA hairpins predicted for chicken (*left*) and human (*right*) transcripts. Putative base-pairing interactions between established chicken dBPs and U2 snRNA are at the bottom; for full legend, see Figure 3F. ss, single-stranded; ds, double-stranded.

To test these assumptions, we used taxon-specific constructs for orthologous BP mapping coupled with mutagenesis of BP motifs. Each construct produced accurately spliced isoform 4b+ in mammalian cells (Supplementary Figure S6A, C). First, *Xenopus laevis* transcripts employed only a single major dBP located 30 nts from the 5′ss of intron 4a and its mutation completely abrogated exon 4b usage (Figure 6A and Supplementary Figure S12), unlike human dBP mutations (Figure 3G). Second, the optimized bird orthologue of dBP+41, which is also present in a subset of reptiles, including alligators (Supplementary Figure S1), dominated the dBP usage while bird orthologues of dBP+31 and dBP+25 were repressed as compared to the human cluster (Figure 6B and Table 2). To test if the bird dBP+41 accounts for the observed exclusive use of exon 4b by exogenous transcripts (Supplementary Figure S6A) and to examine the impact of additional variants in the dBP cluster on splicing, we humanized the dBP cluster in chicken transcripts and ‘chickenized’ the human minigene (Figure 6C). Optimizing dBP+41 and combining the chicken counterparts of dBP+41 and dBP+31 on the human background repressed exon 4a usage. Mutations of bird dBP motifs were not informative (Figure 6C). As with human dBPs, neither conventional Sanger sequencing (Table 2, Supplementary Figure S3E) nor a total of 28 841 414 Ampli-seq reads mapped to chicken transcripts (Figure 6D) showed any deviations of dBP usage upon depletion of both TIA proteins or PUF60 overexpression. Chicken counterparts of putative human dBP+45 were not detected (Figure 6D), despite the presence of a UUA motif just downstream of dBP+41, which is absent in placentals (Supplementary Figure S1).

**Table 2.** Usage of individual dBPs in chicken intron 4a

*DBR1*-deficient cells	Number of Sanger-sequenced clones	Number of informative clones (%)	dBP+23 (dBP+25)	dBP+29 (dBP+31)	dBP+34 (dBP+36)	dBP+39 (dBP+41)
Lacking TIA-1 and TIAR	13	12	0	0	5	7
Overexpressing PUF60	14	14	0	0	2	12
Controls	17	14	1	1	2	10
Total	44	40	1	1	9	29

χ^2^ for 2 × 4 contingency tables comparing the usage of human (Table 1) and chicken dBP orthologues was 37.6 (*P*< 10^−9^) for controls and 95.5 (*P*< 10^−16^) for all clones. Aligned sequences of informative clones are in Supplementary Figure S3E.

Similar to the frog (Figure 6A), BP mapping in transcripts derived from ocean sunfish (*Mola mola*) identified a single dominant dBP (Figure 6E). This dBP was located further away from the 5′ss (46 nts), but still close to the steric threshold (34–36).

Taken together, although *Ogdh* dBP adenines are conserved in amniotes, their relative usage is taxon-specific. The amniote cluster evolved from one dominant dBP in anamniotes by a bidirectional expansion. This process probably conferred a fitness advantage in early terrestrial species by making mobilization of Ca^2+^-mediated ATP supply more fault-tolerant to mutations and, potentially, by expanding regulatory capability of the intron, as proposed for multiple BPs (98). The regulatory capacity of U-binding RBPs predated the dBP expansion, but the expanded cluster was selected prior to the cytosine-to-uridine transition of megaPPTs.

### Unpaired conformation of individual dBPs as a predictor of their usage

Because utilization of splicing recognition motifs can be influenced by RNA folding (108), we explored the structural context of human and chicken dBPs. Secondary structure predictions with overlapping pre-mRNAs containing both MXEs and intron 4a consistently suggested that dBP clusters are located within stable hairpins supported by 9-base pair stems in humans and 8-bp stems in chicken (Figure 6F). Helices containing ≥7 contiguous base pairs are needed for rapid and cooperative annealing of RNA and DNA (109). Under both minimal free energy and centroid folding scenarios, human dBP+25 and dBP+31 are fully exposed in the terminal loop whereas only a bulged A is unpaired at dBP+36; in contrast, dBP+41 as well as position –2 of dBP+36 are base-paired (Figure 6F). In a less stable chicken hairpin, the dBP+41 orthologue is unpaired. In contrast, the number of hydrogen bonds between chicken dBPs and the conserved GUAGUA element of U2 snRNA was similar to that in the human cluster (cf. Figures 3F and 6F), suggesting that canonical dBP:U2 base-pairing cannot *per se* account for distinct usage of individual dBPs in human and chicken clusters.

**Figure 7.**
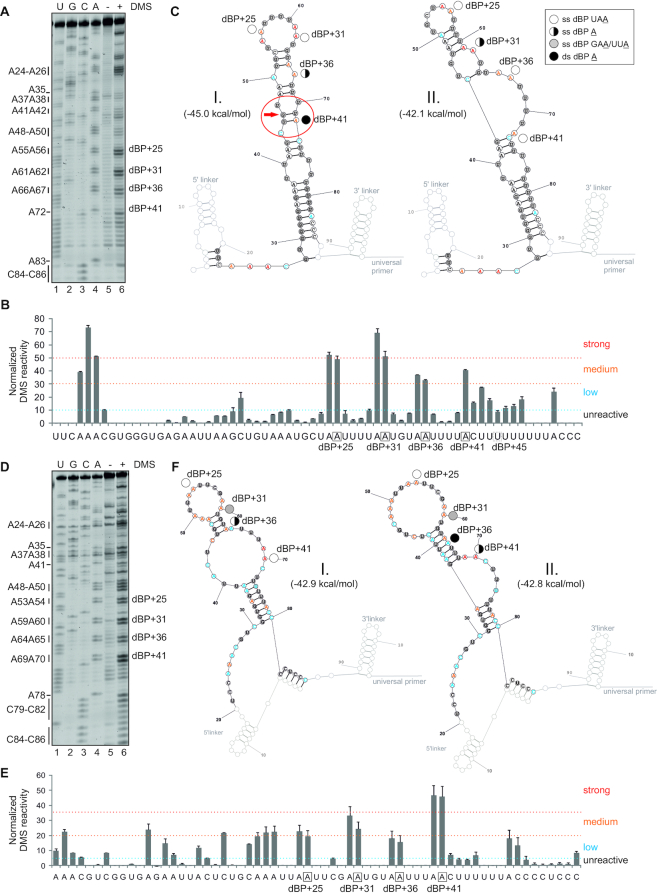
DMS structural probing of human and chicken dBP clusters. (**A–C**) human dBPs; (**D–F**) chicken dBPs. Their usage is shown in Figures 3H and 6D. (**A, D**) Representative gels containing RT products of DMS-treated and untreated RNAs run in parallel with a sequencing ladder. Adenines (*left*) are numbered as in panels **C** and **F**. (**B, E**) Normalized DMS reactivities of duplicate experiments with human (**B**) and chicken (**E**) probes. dBPs are boxed. (**C, F**) Secondary structure predictions showing strong, medium and low DMS reactivities for the most stable (I, *left*) and the second most stable (II, *right*) structures of human (**C**) and chicken (**F**) transcripts. SHAPE-guided structures are in Figure 8. Legend (top right corner of panel **C**) denotes paired and unpaired status of individual dBPs; red arrow shows a wobble pair at the helix end; if disallowed in RNAfold (59), dBP+41 A becomes single-stranded in an asymmetric loop involving nts marked by a red ellipse. Tentative human dBP+45 U (Figure 3H) is single-stranded (panel **C**), but its unused chicken counterpart is double-stranded in the most stable structure (panel **F**).

**Figure 8.**
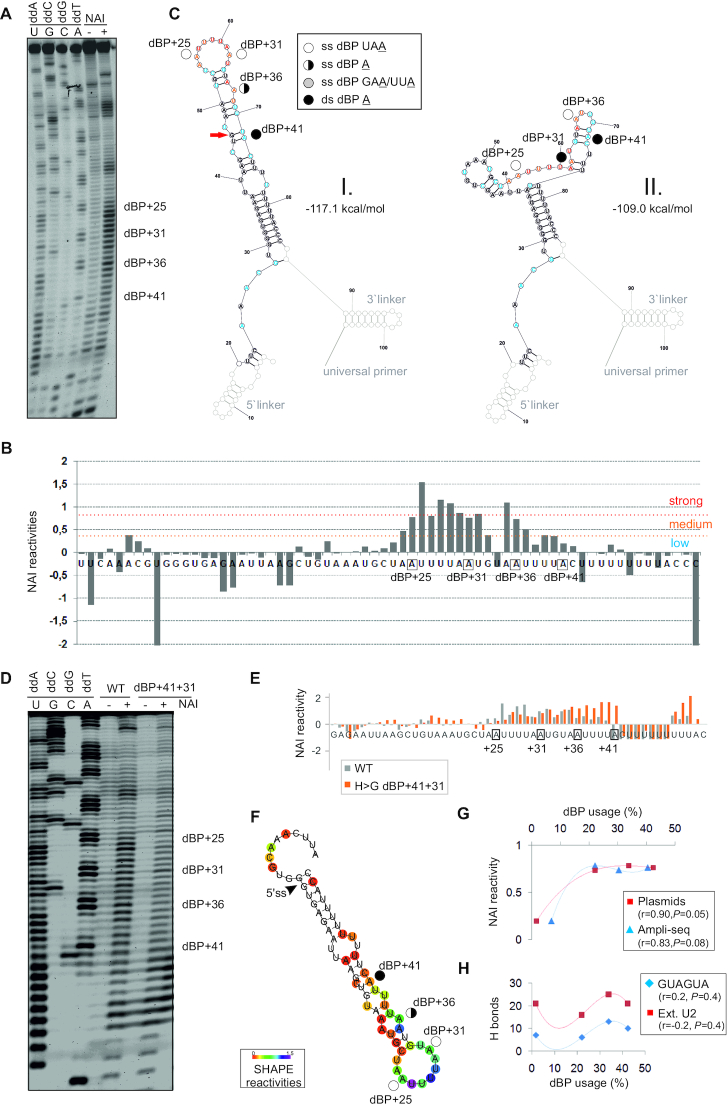
SHAPE with human RNA probes modified by NAI. (**A**) Separated RT products of *in vitro* NAI-treated (+) or untreated (−) reactions with human Cy5-labeled WT RNA probe with linkers. dBP cluster is labeled to the right. Dideoxy sequencing ladder (lanes 1–4) was run in parallel to determine modified positions. (**B**) Normalized NAI reactivities. Nucleotide flexibility is coloured (scale is to the *right*); negative values are shown in full. (**C**) The most stable SHAPE-guided structure (I, *left*) with an alternative stable structure (II, *right*). Predictions were carried out with RNAstructure using reactivity values from panel B. Linkers are greyed. Red arrow shows a wobble pair at the end of helix; if disallowed in RNAfold (59), dBP+41becomes single-stranded. (**D**) Human WT and mutated probes that lack linkers. (**E**) Normalized NAI reactivities for panel D; negative values are cut off at −1. (**F**) Optimal SHAPE-guided secondary structure of the WT RNA probe supported by methods developed by Deigan *et al.* (55) (default slope *m* = 1.9 and intercept *b* = −0.7), Washietl *et al.* (57) (linear mapping and tau/sigma-ratio of 1) and Zarringhalam *et al.* (56) (linear linear log model, slope 1.6, intercept −2.29 and β = 0.8). (**G,H**) Usage of individual dBPs correlates with their NAI reactivities (**G**) rather than predicted U2:BP base-pairing (**H**), shown as the number of hydrogen bonds between dBPs and canonical GUAGUA motif (*blue*) or the extended U2 snRNA region (*red*). r, correlation coefficients and associated *P* values (*F*-tests). For Ampli-seq data, *r* and *P* values were 0.65 and 0.18 (GUAGUA) and 0.26 and 0.4 (extended U2 motif), respectively.

To support these predictions, we carried out structural probing of human and chicken RNAs using DMS and SHAPE. On DMS gels, adenines at dBP+25 and dBP+31 were more reactive than at dBP+41 (Figure 7A, B). The dBP+41 A was predicted to be base-paired in the most stable structure, but was unpaired when disallowing GU wobble pairs at the end of helices (Figure 7C, *left*) or in the second most stable alternative structure (Figure 7C, *right*). By contrast, chicken RNA probes revealed the highest DMS reactivity at dBP+41, consistent with the unpaired conformation both in the most stable and alternative structures (Figure 7D–F). SHAPE with NAI showed elevated NAI reactivities across the human dBP cluster, with a decline toward low-usage dBP+41 (Figure 8A, B). The drop in NAI reactivities at dBP+41was confirmed with the human RNA probe lacking both linkers; in addition, a double mutation that chickenized the human dBP cluster and repressed exon 4a (Figure 6C) reversed the decline (Figure 8D-E). The minimal free energy/centroid folding model (Figure 6F) was supported by SHAPE-guided predictions (Figure 8F), whether considering dynamic perturbation energies (57), pseudo-energies to stacked pairs (55) or discordant positions under the linear log model (56).

Taken together, the species-specific dBP usage statistically correlated with their accessibility in RNA space rather than with the number of hydrogen bonds predicted for canonical base-pairing with U2 snRNA (Figure 8G, H). This finding is consistent with previously reported poor correlation between the BP usage and U2 snRNA complementarity (97,98) and, theoretically, with optional rather than obligatory canonical U2:BP pairing (110). In agreement with complex folding pathways of even small RNAs (52), individual dBPs may adopt alternative conformations that could potentially contribute to dynamic switches in their accessibility during *in vivo* recognition.

### Tracing the origin of *OGDH* dBPs and AGEZ

As compared to tetrapods, established or predicted dBPs in correctly annotated fish intron 4a are on average further away from the 5′ss (Figure 6E, Supplementary Figure S13A), closer to the 5′ss-dBP steric threshold. The average intron 4a size reduced by AGEZ was also larger in anamniotes (or fish) than in amniotes (Supplementary Figure S13B and 1B). Nevertheless, correlation between the AGEZ length and intron 4a size in fish species was still significant (Supplementary Figure S13C, *r* = 0.82 for 19 fish genomes, *P* < 10^−4^), arguing against a possibility that the 5′ss in anamniotes escaped the dBP-mediated repression. This analysis also revealed a single outlier (*Mola mola*) with a significantly reduced AGEZ (Supplementary Figure S13B, C, *left panel*). Sanger sequencing of *M. mola* DNA confirmed that the first downstream AG dinucleotide was in the middle of the megaPPT (Supplementary Figure S13C, *right panel*). Such AGEZ spoilers, both within and outside TEs, may be recognized by unproductive spliceosome assemblies that scan pre-mRNAs downstream of BPs for 3′ss AGs, potentially repressing canonical 3′ss (44,104,105). Consistent with this notion, exogenous transcripts derived from ocean sunfish failed to select this GAG motif as 3′ss and showed the highest relative abundance of 4a−4b− transcripts among tested taxa while maintaining the PUF60/TIA-1 dependencies (Supplementary Figure S13D). However, the high level of skipping of *M. mola* MXEs was not corrected by point mutations that extended the AGEZ to its canonical size of ∼280 nts (Supplementary Figure S13E) while using the same dBP (lanes 2–4, Figure 6E). We found no fish orthologues with YAG motifs downstream of their putative dBPs; these motifs are preferred 3′ss (111). Unlike other fish, *M. mola* contains simple repeats upstream of the AGEZ spoiler (Supplementary Figure S1) that could potentially make the GAG motif less accessible to the scanning process. Also, a subset of fish species contained one or more UAA/UGA motifs ∼150 nts from the 3′ss of intron 4a (Supplementary Figure S1), however, our repeated attempts to determine the BP in cod and zebrafish transcripts were unsuccessful.

Together, the long AGEZs found in most but not all fish genomes support a strong protection of the ancient dBP/megaPPT arrangement, which probably emerged upon duplication of the ancestral exon or early after. The AGEZ reduction by the GAG motif in *M. mola*, the world's largest bony fish, was tolerated, but mutations creating more efficiently recognized 3′ss motifs were probably purged during evolution to preserve the expression of Ca^2+^-responsive isoform 4b+.

### dBP/megaPPT 3′ss organization and MXE-regulated Ca^2+^ signaling

**Figure 9.**
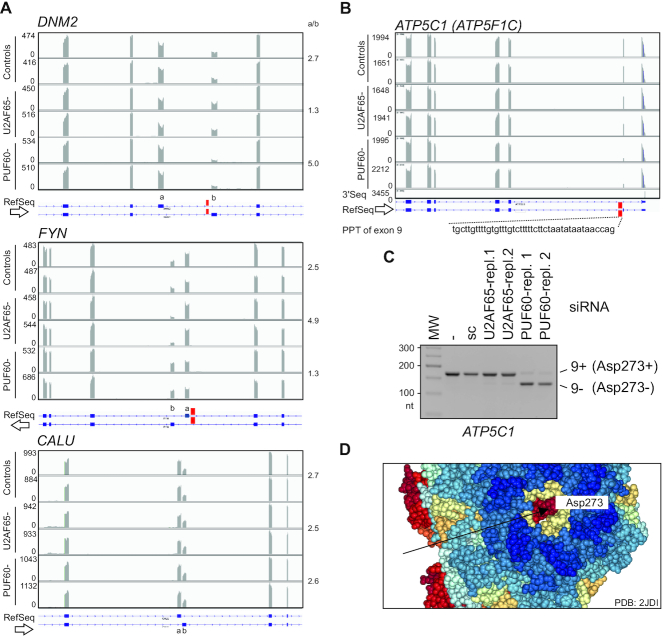
Exon-level co-regulation of OGDH and the γ-subunit of F_1_-ATP synthase. (**A**) UG-rich PPTs precede PUF60-activated MXEs. Genome browser views of RNA-seq of HEK293 cells depleted of PUF60, U2AF65 and untreated controls (in duplicates). UG repeats are denoted by red rectangles in *DNM2* and *FYN* (*top two panels*); they are absent in a 73-nt intron between insensitive *CALU* MXEs (*bottom panel*). Mean MXE ratios (a/b) calculated from Sashimi plots are to the right. Y-axis, read numbers (ArrayExpress accession number E-MTAB-6010). Arrows indicate transcriptional orientation. (**B**) Alternative splicing of PUF60-dependent *ATP5C1* exon 9. Location of UG-rich PPT is denoted by a red rectangle. 3′ end sequencing track (3′Seq, ref. 195) shows a downstream polyadenylation site. (**C**) Validation of PUF60-dependent splicing of exon 9 by RT-PCR; sc, scrambled control (41). (**D**) Spacefill representation of the ground state bovine structure of the F_1_F_o_-ATP synthase at 1.9 Å resolution (196). Arrow denotes the C-terminal amino acid of the γ-subunit encoded by *ATP5C1* isoform 9+, but not by isoform 9-.

How common are the dBP/megaPPTs in protein isoforms regulated by Ca^2+^? Employing MXEs predicted in the human genome (112 and Martin Kollmar, personal communication) and updated overrepresentation tests for biological processes categories (66), we confirmed a significant enrichment for genes involved in muscle development, as reported (112). When considering molecular function categories, the highest enrichment was found for ion channel signalling led by Ca^2+^ (Supplementary Figure S14). We then selected MXEs with the size of intervening introns between 60 and 500 nts where SVM dBP predictions should be more accurate than in longer introns. The selection yielded 41 introns with a distance of <50 nts between the predicted dBPs and the 5′ss, a steric threshold for simultaneous U1 and U2 snRNP recruitment (34–36). Of the 41 introns, 23 contained (U)_4_ runs, 10 had (U)_6_ runs and 4 introns had (U)_>8_ motifs downstream of BPs; all isoforms of the latter group have distinct sensitivities to Ca^2+^ and/or function in mitochondria (Supplementary Table S6). Examination of an independent sample of 97 genes with MXE exons (113) revealed at least 29 genes (30%) with a role in Ca^2+^ signalling, with 21% products localized into mitochondria and 13% into the matrix (Supplementary Table S7). The median intron size of the 29 genes was just 0.5 kb, with at least 10 introns with predicted dBPs. Inspection of their PPTs revealed frequent partitioning into UC, UA or UG-rich segments (for example, in *ACTN2, CACNA1A* or *SCN5A*; Supplementary Table S7). We observed opposite responses of MXEs to U2AF65 and PUF60 knockdowns where PPTs upstream of 3′ss of either MXE contained UG-rich motifs; this regulation was eliminated in their absence (cf. *DNM2* or *FYN* with *CALU*, Figure 9A).

Finally, we asked whether any measurable properties of a Ca^2+^-dependent enzyme could be related to the size of introns that separate MXEs. As shown by McCormack and Denton, Ca^2+^ diminished *K*_m_ values for 2OG of human, rat, pigeon, trout and frog OGDHC isolated from hearts and this reduction was much smaller in trout and frog than in mammals or birds (19). Despite the paucity of species, the *K*_m_ decline inversely correlated with intron 4a size (Supplementary Figure S15).

Together, these data suggest that the dBP/megaPPT organization of MXE 3′ss is associated with gene products regulated by Ca^2+^. Because intracellular Ca^2+^ is a critical ion for muscle contraction (114), this association could account for the observed enrichment of MXEs in genes involved in muscle development. MegaPPTs and their partitioning should increase the capacity to control inclusion levels of MXEs by permitting a wider RBP repertoire to access the pre-mRNA.

### Exon-level regulation by PUF60 and Ca^2+^-mediated ATP supply

Of 219 genes with one or more PUF60-dependent exons (41), 42 (19%) were identified by MitoMiner (v.4.0) (70) as encoding known (*n* = 30) or predicted (*n* = 12) mitochondrial proteins (Supplementary Table S8). For example, *ATP5C1* (also known as *ATP5F1C*), which encodes the γ-subunit of the F_1_F_o_-ATP synthase, contains a PUF60-activated alternative exon 9 (Figure 9B, C). This exon introduces a stop codon in the mRNA; transcripts lacking exon 9 produce a 197-aa isoform 1 expressed in striated muscles, tissues with high and variable energy demand. In contrast, transcripts 9+ generate a 198-aa isoform 2 expressed in liver and other tissues (115 and refs. therein). In cells lacking PUF60, reduced expression of Ca^2+^-sensitive OGDH isoform 4b+ is associated with enhanced expression ATP5C1 isoform 1 (Figure 9B, C). The reciprocal regulation of the two muscle isoforms involves PUF60 binding to *OGDH* intron 4a (Figure 2A) and, most likely, to a conserved U/UG-rich PPT near the 3′ss of *ATP5C1* exon 9 (Figure 9B, C), consistent with its importance for this alternative splicing (115). The γ-subunit forms a central shaft rotor in F_1_ that penetrates the stator cylinder via a coiled coil of N-terminal and C-terminal α-helices (116) and is attached to the c-ring of F_0,_ facilitating rotation within F_1_ subunits α_3_β_3_ (117). The c subunit of F_0_ was proposed to be important for opening of the permeability transition pore complex induced by Ca^2+^_c_ overload (118). The extra aspartate encoded by *ATP5C1* isoform 2 is prominently positioned between the stator and motor in non-muscle tissues (Figure 9D), potentially influencing chemical motor properties, such as rotation, torque or ATP hydrolysis. Because the F_1_F_o_-ATP synthase does not appear to be directly bound by Ca^2+^ (17,119), the rotor may need a distant, exon-level control that links the chemical motor to the Ca^2+^_m_-dependent TCA cycle flux. Indeed, substrate level variations cannot explain flux alterations through the F_1_F_o_-ATPase whereas Ca^2+^_m_ is a major promoter of the enzyme activity (120).

Additional examples of PUF60-dependent alternative splicing in genes involved mitochondrial metabolism are in Supplementary Figure S16A-D. They include *AK2*, which alters the availability of the ATP binding site encoded by the last exon, *AFG3L2*, which mediates degradation of SMDT1/EMRE before its assembly with the mitochondrial Ca^2+^ uniporter (MCU) complex, limiting the availability of SMDT1/EMRE for MCU assembly (121), and *GLS*, which catalyzes the hydrolytic deamidation of glutamine to glutamate (122). Together, these results identify exon-level regulation of distant steps of NADH/ATP supply pathways by PUF60.

### Auxiliary splicing motifs in exons and the Irving-Williams series

Ca^2+^ sensitivity of OGDHC is largely attributable to the DADLD motif encoded by *OGDH* exon 4b (32,33). The motif is in the middle of exon 4b between a conserved 5′ and more diversified 3′ exonic portions (Supplementary Figure S17). Apart from this motif, human and mouse E1 subunits contain a similar site, termed site 2 or ESDLD, which is encoded by *OGDH* exon 6 (32). The Ca^2+^ sensitivity of mutated E1 lacking site 2 showed modest decreases in the *K*_m_ value for 2OG (32). Alignments of pro- and eukaryotic E1 suggested that both the canonical DADLD motif and site 2 evolved around the central aspartate (D) residue present already in *E. coli* (Supplementary Figure S17). The central D also sustained the smallest number of synonymous substitutions during vertebrate evolution as compared to flanking D residues (Supplementary Figure S1).

**Figure 10.**
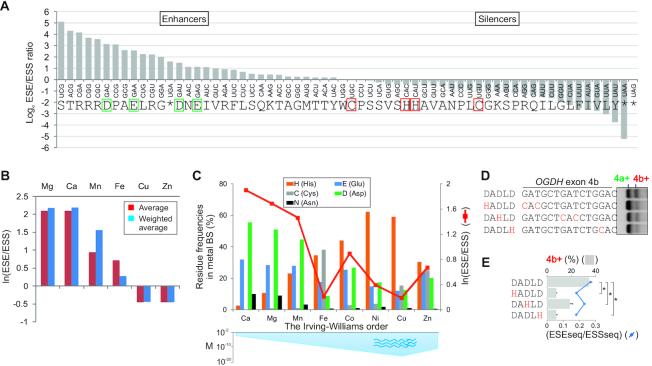
Auxiliary splicing motifs in exons and the Irving-Williams affinity series. (**A**) Sixty-four codons ranked by lo**g**_e_ ESE/ESS ratios. Asterisks denote stop codons. Amino acids preferentially involved in Ca^2+^ or Mg^2+^ binding are boxed in green; residues preferentially involved in Cu^2+^ and Zn^2+^ binding are boxed in red. (**B**) ESE/ESS ratios for codons that encode key residues in protein binding sites for the indicated metals mirror the Irving-Williams affinity order. The weighted average ratios were derived with residue frequencies estimated by fragment transformation methods (61). (**C**) Weighted average log_e_(ESE/ESS) values for the indicated amino acids (*top*) and metal ions (*bottom*). The values were computed for residue frequencies in ∼290 000 metal binding sites from >50 000 pro- and eukaryotic structures (62). The *lower panel* shows estimates of molar concentrations (M) of the free metals in the primordial sea of the Earth (∼4500 Myrs ago), as reported previously (183,197). (D, E) ESE to ESS (Asp to His) reversal of the Ca^2+^-binding DADLD motif in *OGDH* exon 4b. (**D**) Exon 4b inclusion in mature transcripts upon transient transfection of WT and mutated reporters into HEK293 cells. Mutations are in red. (**E**) Measurements of exon 4b inclusion levels for panel D. Error bars represent SDs from two independent transfections. Asterisks denote significant differences for the indicated comparisons (*P*< 0.05, ANOVA with Dunnett's post-hoc tests). The ESEseq/ESSseq ratios were calculated for all hexamers in 25-nt segments around the WT or mutated DADLD motifs using ESEseq and ESSseq scores published previously (60).

Because D and glutamic acid (E) residues have been indispensable for the emergence of Ca^2+^ binding sites (reviewed in 123), we set out to explore the relationship between D and E codons and their splicing outcomes in more detail. We first computed codon counts in a comprehensive set of splicing activating and inhibitory hexamers previously identified by RNA-seq (60). We found that the number of D and E codons in hexamers that activated splicing was ∼7x higher than in inhibitory hexamers (*P* < 10^−6^, binomial test; Table 3). However, D and E residues are also predominant in Mg and Mn binding sites; by contrast, histidine (H) is overrepresented in Cu, Fe and Zn binding sites while cysteine is most enriched in the latter (61). These four residues are most frequently involved in binding metal ions (61,62,124). Unlike D and E codons, H and C codons were enriched in exonic splicing silencers (Figure 10A, Supplementary Figure S18A-B) and their mean ESE/ESS ratios were significantly lower (*P* < 0.005).

**Table 3.** Prevalence of codons critical for the emergence of Ca^2+^ binding sites in splicing regulatory hexamers

Amino acid	E (Glu)	E (Glu)	D (Asp)	D (Asp)	
Codon	GAA	GAG	GAC	GAU	Total
ESEs (*n* = 1182)^a^	146 (0.43)	105 (0.37)	177 (0.49)	113 (0.38)	541
ESSs (*n* = 1090)^a^	10 (−0.37)	32 (−0.30)	7 (−0.34)	23 (−0.41)	72
Fold excess of codon frequencies in ESE over ESS^b^	13.5	3.1	23.3	4.5	

^a^Hexamers and their ESEseq and ESSseq scores were reported previously (60). The scores represent quantitative measures of splicing activities of exon hexamers (60). Mean ESEseq or ESSseq scores of hexamers containing the indicated codons are in parentheses.

^b^Frequencies were computed for 4728 codons in ESEs and 4360 codons in ESSs. Predicted splicing activities of all sense and nonsense codons are shown in Figure 10A and Supplementary Figure S18.

Remarkably, the order of average ESE/ESS ratios computed for residues critical for formation of protein binding sites for common metals (Figure 10B) mirrored the natural order of their affinities (Mg^2+^/Ca^2+^< Mn^2+^< Fe^2+^[<Co^2+^< Ni^2+^] < Cu^2+^/Zn^2+^), known as the Irving-Williams series (125–127). This observation indicates that the weakest binders in the series (Mg^2+^ and Ca^2+^) preferentially interact with residues encoded by splicing-enhancing codons whereas codons for residues interacting with tight binders (Cu^2+^ and Zn^2+^) act as splicing repressors, with moderate binders (Mn^2+^ and Fe^2+^) exhibiting intermediate values of ESE/ESS ratios and splicing activities (Figure 10B and Supplementary Figure S18C, D). The same order was found for hexamer preference indices previously computed for independently derived sets of exonic splicing motifs (Supplementary Figure S19). For some collections, the order of hexamer preference indices for the most competitive metals (Cu^2+^ and Zn^2+^) was flipped (Supplementary Figure S19), as were the stabilities of Cu and Zn complexes in the Irving-Williams order (128). The exact hierarchy of D and E codons (GAC > GAA > GAU > GAG) found for the complete set of 4096 hexamers was not always preserved in independent ESE sets (Supplementary Figure S19), however, these ESE sets were derived by distinct methods and overlap only partially with the comprehensive hexamer set (60,63,129,130).

When extending this comparison to all metal sites in published pro- and eukaryotic macromolecular structures (62), we observed a highly similar gradient of ESE/ESS ratios across the Irving-Williams stability order (Figure 10C), confirming the dominant role of D (enhancing) and H (silencing) codons. This analysis also revealed a less pronounced gradient for codons encoding asparagine, a residue common in the Ca^2+^-binding EF-hands (131).

To support the enhancing and silencing roles of D and H codons experimentally, we replaced each D codon in the DADLD motif of the *OGDH* reporter with a relatively weak silencing codon CAC for H (Figure 10D). As expected, each mutation diminished *OGDH* exon 4b inclusion levels in mature transcripts (Figure 10E), with a remarkably high correlation with average ESEseq/ESSseq scores of WT and mutants (*r* = 0.98, *P* < 0.05). Ultimately, the overall ESE/ESS gradient across the Irving-Williams series mirrored concentrations of these metal ions in the primordial (sulphid) ocean (Figure 10C).

We conclude that mutations creating codons for amino acids required for binding of weak metals in the Irving−Williams order (Ca/Mg) favour exon inclusion whereas mutations generating codons for residues that interact with highly competitive binders (Cu/Zn) generally promote exon skipping (Figure 10A-C). Loss of these codons has the opposite effect (Figure 10D, E). Because exonic auxiliary splicing motifs are highly conserved (132), residues critical for the emergence of Ca^2+^ binding sites must have acted as universal splicing enhancers, promoting exon usage during vertebrate evolution. Thus, the DADLD motif in Ogdh not only binds Ca^2+^, it promotes isoform 4b+ (Figures 10D, E), acting in the same direction as dBPs (Figure 3), allele 4b+91A in endotherms and the exon 4a codon loss in amniotes (Figure 5, Supplementary Figure S1). After exon duplication, this combined action may have been necessary to preserve expression of isoform 4b+ in all tissues (Supplementary Figure S9A), thereby avoiding undesired loss of the allosteric regulation of E1 and facilitating subfunctionalization of the exon pair.

## DISCUSSION

Our results provide new insights into regulation of the inseparable exon tandem that controls OGDHC activation and NADH and ATP supply in response to Ca^2+^_m_. Remarkably, the *OGDH* exon duplication arose about 2,000 Myrs after the earliest living organisms on the Earth had selected the inseparable tandem of ATP and low Ca^2+^_c_ to control cellular signalling, apparently as a compromise between ATP-based energetics and poor Ca-ATP solubility (133). First, we have shown that the MXE usage is tightly controlled by intron 4a sequences spliced via dBPs. The dBPs introduce and protect the megaPPT platform that binds various RBPs to orchestrate activation or repression of flanking splice sites (Figures 1–3), providing a stringent control of each MXE. This highly conserved regulation couples the RBP network to the critical flux control point of the TCA cycle and connects OGDHC to ATP delivery pathways at the exon level, including the rotor of the ATP chemical motor itself (Figure 9B−D). Consistent with purifying selection of intron 4a orthologues over hundreds of Myrs of vertebrate evolution, the intron has been completely immune to any TE or non-TE expansion (Figure 1 and 4). TE insertions can supply or eliminate dBP competitors, impair the cell-specific MXE pattern, inhibit adjacent splice sites or reduce canonical *OGDH* isoforms, and relieve the 5′ss repression by dBPs (Figure 4, Supplementary Table S3). The dBP-driven selection for the short intron was thus likely to facilitate diversification of exon 4a and 4b without losing the Ca^2+^-dependent OGDHC activity. We have also shown that auxiliary splicing motifs in exons display a dichotomy for weak and strong metal binders in the Irving-Williams affinity series (Figures 10, Supplementary Figures S18 and S19). Finally, we hypothesize below that the *OGDH* MXE regulation has served as an ancient selection instrument contributing to the independent emergence of multiple heterothermic and endothermic species in line with the aerobic scope model (5). This process was driven by muscle thermogenesis fuelled by sustained and Ca^2+^-responsive ATP supply during maximum aerobic activity.

### Evolution of MXE-enforcing mechanisms

MXEs represent the rarest type of alternative splicing with only ∼855 predicted pairs in the human genome (112). By offering the cell a choice of employing 2 or more protein isoforms that are very similar in size and function, MXEs permit smaller modifications of protein activity than intron retention, alternative 3′ss or 5′ss, or cassette exons (112,134,135), ensuring the continuity of essential cellular functions after exon duplication, such as Ca^2+^-activated energy supply. MXE genes are also more conserved and more enriched for translated products (112,113). MXE splicing may be enforced by a reading frame incongruity through nonsense-mediated RNA decay (NMD) (112), incompatibility of U2 and U12 spliceosomes (135), RNA secondary structure (39,136,137) or the 5′ss-to-dBP proximity (35). The size restriction of *OGDH* intron 4a (Figures 1 and Supplementary Figure S13), the proximity of dBPs and the 5′ss (Figure 3 and 6) and the impaired MXE usage upon intron expansions by TEs (Figure 4) implicate the latter mechanism. A predicted fraction of human MXE pairs constrained by short 5′ss-BP distances was estimated at ∼9% (112), i.e. perhaps only ∼75 cases.

The combined length of *OGDH* exon 4a and 4b is not a multiple of 3 nts, yet the ‘NMD-immune’ *OGDH* constructs derived from endotherms did not produce out-of-frame transcripts, unlike those derived from ectotherms (Supplementary Figure S6). However, NMD may have contributed to their removal early after the exon duplication when the intron was longer (Figure 1); core NMD components are present already in early eukaryotes (138). If the 5′ss is not repressed by dBPs and intron 4a is spliced, NMD could limit E1 expression and the Ca^2+^-activated ATP supply during sustained muscle activity. Apart from NMD, the observed taxon-specific MXE usage could reflect local conformational flexibilities at the dBP cluster (Figures 3, 6–8). Combination of RNA secondary structure and dBPs was previously implicated in splicing of *Drosophila Dscam* MXEs 17.1 and 17.2 (139). Future studies should address how the relative importance of MXE-enforcing mechanisms changed during evolution.

### Ca^2+^ signalling and the dBPs/megaPPT organization of 3′ss

MXE gene products are overrepresented among proteins involved in Ca^2+^ signalling (Supplementary Figure S14). Because Ca^2+^_c_ is a critical ion in muscle contraction (114), the association could account for the enrichment of MXEs among genes related to striated muscle function (112). The link may be even stronger when considering exon duplications that diverged beyond recognition but their PPTs/AGEZs remained conserved. For example, several *HTR4* exons have similar extended AGEZs and employ multiple dBPs, some in combination with weak canonical BPs (140). HTR4 activation attenuates mitochondrial Ca^2+^ uptake under normoxic and hypoxic conditions and inhibits opening of the mitochondrial permeability transition pore (141).

MegaPPTs are common in MXE genes (Supplementary Table S6,S7). They provide large and accessible platforms for RBPs (Figure 2) that could help co-regulate expression of muscle-specific proteins at the exon level. The presence of U-rich sequences in the vicinity of muscle exons (142,143), evolutionary transitions in PPTs of MXE genes (Supplementary Figure S7), a failure of MXEs lacking PPTs to respond to knockdowns of U-binding proteins (Figure 9A) and reduced binding upon PPT shortening (41,73,144) support this notion. U-binding preferences are very common among RBPs; in solved RRM structures U is the most frequent nt recognized sequence-specifically and was found in all nt-binding pockets (145). TIA-1 and PUF60 EMSA profiles were similar but not identical (Figure 2A), raising a possibility that the proteins compete for RNA binding and activate either MXE indirectly. Notably, TIA-1 bound an unstructured ribonucleotide covering the dBP cluster, but eCLIP signals of TIA-1 and other MXE regulators were absent in this region (Figure 2A, B). The drop of 5′ eCLIP read ends at dBPs is reminiscent of that observed for the Aquarius helicase reads at BPs (146). Aquarius binds single-stranded RNA but not blunt-ended duplexes, displays 3′-to-5′ unwinding activity and contacts U2 SF3a and SF3b proteins within activated spliceosomes (147). The extent to which this pattern reflects crosslinking to lariats in unsynchronized cells or hairpin structures across dBPs (Figures 6–8) remains to be determined.

High-usage *OGDH* dBPs (Figure 3) resemble CUAAC motifs that are enriched downstream of regulated muscle exons as compared to constitutive exons and appeared to be depleted upstream (148,149). The YUAAY motifs are recognized by the STAR family of RBPs, including SF1 (150,151), QKI (152), SAM68 (153) and SLM-2 (154). *Ogdh* was among the most sensitive targets reported in myoblasts depleted of QKI (152), which is expressed in muscles, heart and brain (155). In contrast, the *OGDH* MXEs seemed to be insensitive to SF1 knockdowns in HepG2 or K562 cells (156) and in HEK293 cells (J.K., unpublished data). The colocalization of dBP motifs and targets of the hnRNP K homology/Quaking domains of STAR proteins should be examined in more detail.

### Intron size restriction by dBPs

Intron 4a could not be extended without losing optimal MXE control and was shortened instead during evolution, defying the opposite trend in higher vertebrates (Figures 1,4 and Supplementary Figure S1). The length was reduced by small deletions in both directions from dBPs, consistent with diminishing RS scores from the dBP cluster toward each splice site (Figure 1D), but the 5′ss-dBPs distance remained almost constant in endotherms (Supplementary Figure S13B). Small insertions within the adenine-poor megaPPT would compete less efficiently as BPs than retrotranspositions because short expansions are usually tandem duplications of adjacent sequences (157). Unlike long introns, the size of very short introns was negatively correlated with the intrinsic strength of splice sites, but the correlation was weak (158,159) and the relaxation of *OGDH* intron 4a splice sites in vertebrates had a minimal impact on exon 4a/4b usage (Supplementary Table S4, Supplementary Figure S5). The ESE enrichment in exons flanking short introns was also weak or nonexistent (158), also arguing against a key role in shaping intron 4a size. Thus, the intron 4a size restriction is largely imposed by the dBP/megaPPT organization of 3′ss.

### BP mapping and stability of intron lariats

Global identification of BPs has been hindered by low expression of parent genes, tissue-specific alternative exon usage and by short introns; the latter limited the success rate to ∼20% (98). In addition, detection of multiple adjacent BPs has been hampered by a high mutation rate of RT as the enzyme traverses the 2′-5′ linkage between the 5′ss guanine and BP, introducing small deletions/insertions and substitutions that are diagnostic for BPs (Supplementary Figure S3) (97,99,160,161). For example, only the most downstream dBP of *OGDH* intron 4a (chr7:44 687 173, hg19), but no upstream dBPs, were found among a ‘match-only’ set of 77 668 BPs, but no intron 4a dBPs were reported in the high-confidence set of 59 359 BPs (97) (Figure 3A).

Although BPs can be determined with exogenous taxon-specific pre-mRNAs correctly spliced in mammalian cells (Figures 3, 6 and Supplementary Figure S12), the success rate of our targeted BP mapping remained suboptimal even in cells lacking DBR1. A key obstacle seems to be a wide range of spliced lariat intron stabilities. Lariat introns may be degraded within seconds or days after their excision from newly formed transcripts (162,163). Some stable lariats are selectively exported to the cytoplasm in multiple species and were associated with a 100−500-nt intron size range (162,163), but we found no *Ogdh* introns among these transcripts. In *S. cerevisiae*, lengthening of short BP-3′ss distances was suggested to destabilize intron lariats (164), nevertheless the MIR15 insertion downstream of dBPs did not preclude BP mapping (Figure 4D-F). The distance between the 5′ss of *OGDH* intron 4a and high-usage dBPs is even smaller as compared to other dBP introns in mammals or viruses, including 41 nts in rat *Tpm1* intron 2 (35) or 48 nts in the SV40 t-intron (34). However, its precise role in 5′ss inhibition remains unclear. Increasing this distance by TE insertions did not always derepress the 5′ss (Figure 4C, 2A), but the insertions may displace regulatory RBPs such as TIA-1.

Lariat stability can be affected by the identity of the BP nucleophile, such as cytosine (163), which is a poor substrate for DBR1 (165,166), but we found no cytosine-branched lariats in any *OGDH* orthologues (Figures 3D, H, 6D, E, Supplementary Figure S12). DBR1 depletion altered the relative use of two adjacent TCA and TGA BP motifs in a *GANAB* intron (41), suggesting that the structural context of extended BP motifs can influence DBR1 selectivity. DBR1 provides a positively charged binding surface complementary in shape to that of the BP and flanking nucleotides, which includes a unique lariat recognition loop (167). Processing of circular intronic RNA required GU-rich motifs near 5′ss and C-rich motifs near BPs (168), raising a possibility that their base-pairing may be involved in the escape of circular RNAs from debranching (169). Although a DBR1-mediated lariat accumulation may improve BP detection rates, DBR1 inhibition was reported to induce skipping of exons with weak BPs (170), but it did not alter exon 4a/4b ratios (Supplementary Figure S3B).

### Evolution of the dBP/megaPPT organization in *OGDH*

The dBP/megaPPT arrangement in *OGDH* has been maintained for hundreds of Myrs, defying any intron expansion (Figures 1, 3, 4 and Supplementary Figure S13). In slowly evolving genomes such as platypus or alligator (171), intron 4a remained somewhat longer and failed to accumulate microdeletions (Figure 1C and Supplementary Figure S1). Such megaPPT deletions were not overly detrimental to the inclusion of either exon, neither did they induce unproductive transcripts (Supplementary Figure S5). Interestingly, both alligator and platypus have inefficient dBP+31 orthologues, unlike some other reptiles or birds (Supplementary Figure S1).

The dBP cluster expanded in terrestrial amniotes from a single major dBP in anamniotes and evolved polymorphic extended motifs that control taxon-specific accessibility of dBP adenines (Figures 3, 6-8 and Supplementary Figure S12). The tighter association of dBP usage and NAI reactivity as compared to the strength of canonical U2:dBP base-pairing (Figure 8G, H) suggests that BP accessibility is a more important usage predictor. Formation of competing pre-mRNA structures is an important component of the mechanism that guarantees the inclusion of only one MXE in mRNAs, first proposed for *Drosophila Dscam* (136). Assuming that exon 4a came second (33), exon 4b duplication may not have copied the full dBP hairpin (Figure 1D, 6-8) and instead, it may have created a pair of complementary sequences competing for distinct exons, looping out just one MXE (172). The dBP hairpin (Figures 6–8) is formed as a part of larger, highly dynamic secondary structure assemblies that must include RNA junctions, which would approximate MXE splice sites and regulatory U-rich sequences in space and help coordinate MXE usage. Formation of a three-way junction has recently been implicated in splicing of yeast *SUS1* exon 2 (173). Introns predicted to have more stable RNA structures were on average less likely to be spliced efficiently than less structured introns (174).

About three-quarters of human constitutive introns were proposed to exhibit multiple, tissue-specific BP usage (98). It is tempting to speculate that redundant dBPs might be exploited by tissue-specific regulator(s), such as those that repress *Ogdh* exon 4b in neurons or activate the 5′ss immediately upstream (Supplementary Figure S8-S10). Unlike in frog (Supplementary Figure S12B), human dBPs were compensated by the remaining dBPs in HEK 293 cells (Figure 3G), although this might not necessarily be the case in other cell types. The relative dBP usage was not significantly altered in cells lacking or overexpressing MXE regulators that bind this region (Tables 1, 2; Figures 2A, B, 3G, 6D) and the markedly distinct use of chicken and human dBPs in the cluster was associated with similar splicing patterns of endogenous transcripts in a bird and mammals (Figures 3G, 6D, Supplementary Figure S9). Although the function of individual dBPs remains unclear, the expansion was associated with progressive reduction of unproductive transcripts (Supplementary Figure S6) and expanding repertoires of splicing factors in higher vertebrates. In any case, the expansion ensured both the robustness and the evolvability of Ca^2+^-responsive OGDHC activation in animal tissues, consistent with a complexity-linked degeneracy of the first splicing step.

### Origin of *OGDH* exon duplication and subfunctionalization

Internal gene duplications occur at high frequencies estimated at 0.001−0.013 per gene/Myr, similar to the duplication rate of entire genes (175). Up to 17% of the genes in six genomes carried duplicated intronic and/or exonic regions, with a median size of duplication ∼0.1 kb (175). The ancestral reconstitution of exon 4a/4b duplication was not possible because of highly diverged flanking introns and suboptimal annotations of *OGDH* para-/orthologues in lower vertebrates, but the size of conserved *OGDH* MXE relics in extant species (Figure 1D) is not too far from the median.

Although *OGDH* 4a/4b duplication was proposed to predate the emergence of Ca^2+^ binding site (33), genomic data (176,177) reveal species with orthologous DADLD motifs without exon duplication, including *Lottia gigantea* (mollusc) and *Amphimedon queenslandica* (sponge), probably the oldest surviving metazoan. This suggests that Ca^2+^ sensitivity may have been selected for without duplication and this feature may have been lost in multiple lineages. This scenario is supported by evidence for the independent origin of Ca-binding DxDxDG motifs (178), which are more likely to promote than reduce exon inclusion in mRNAs (Figure 10, Supplementary Figure S18-S19). The first position in the DADLD motif is occupied by alanine in multiple species, including *Callorhinchus milii* and *Capitalliateleta*, and the D>A substitution leads to loss of Ca^2+^ sensitivity (33). Several *Nematoda*, *Rotifera* and *Insecta* orthologues contain the SADLD motif, but how exactly D>S substitutions affect Ca^2+^ affinities is unknown. The same position is occupied by N in *Trichoplax adhaerens*, which lacks muscles but is capable of locomotion, by E in *Helobdella robusta* (leech) and by L in *Daphia pulex* (water flea), however, rapid paralogue divergence and incomplete annotations leave us with uncertainties about their exact identities.

### Metallome and spliceosome cross-talk

Apart from having important roles in recognition of exon boundaries and alternative splicing (60,129,179,180), exonic splicing enhancers/silencers evolved to promote/repress exons that encode binding sites for weak/tight metal binders of the Irving−Williams order (Figures 10, Supplementary Figures S18 and S19). This finding suggests that exonic auxiliary splicing motifs contributed to the extraordinary expansion of eukaryotic protein binding sites for weak metals, especially Ca^2+^. These metals have high coordination numbers and affinity for ligands of low polarizability (181) and are therefore more suited for signalling between organelles/cells and for more complex regulation as opposed to tight binders at the other end of the Irving-Williams order. The dramatic expansion of Ca^2+^ binding sites in larger proteomes was not matched by Zn or Fe binding sites (127) whose key residues are encoded by exon-repressing codons (Figures 10, Supplementary Figure S18-S19). Evolution of Ca^2+^ binding sites was more susceptible to simple substitutions as compared to Zn binding sites, which required more extensive changes of protein structural elements (182). Weak binders (Ca, Mg) also show the highest frequencies of oxygen atoms in metal binding sites, followed by Mn/Fe (61,131), most likely reflecting a switch to oxidative chemistry in early living systems and energy capture from oxygen (183). The increased exon inclusion afforded by codons critical for the OGDH Ca^2+^ binding site (Figure 10D,E) supports the scenario that exon 4a came second (33).

In ∼290 000 sites from >50 000 macromolecular structures (62), H is the most prevalent residue in combined pro- and eukaryotic MetalPDB entries and, together with D, the two residues also dominate the ESE/ESS gradient across the Irving−Williams series (Figure 10C). D and E codons (GAR and GAY, respectively, where R is purine and Y pyrimidine) are also frequent among independently derived exonic splicing enhancers (e.g. (129)). One of the most potent enhancer motif GAA was originally identified in a gene encoding a Ca^2+^ binding peptide and recruited serine/arginine-rich proteins (184). Apart from common metals, the Irving-Williams series includes elements that are much less frequent in mammalian metallomes, such as cobalt. The high ESE/ESS ratio of this outlier could reflect a very limited number of cobalt binding sites in mammals and less accurate residue frequencies (Figure 10C).

Our results suggest that the evolution of exon-level regulation by alternative splicing was constrained by and subservient to properties of biologically available divalent metals, including decreasing ionic radius and increasing electron affinity, polarization and covalence in the Irving-Williams stability order. This would imply that codon preferences for exon inclusion ultimately reflect the availability of free metal ions nost just inside but also outside of the cells (Figure 10C) (183). Restriction or promotion of coding gene segments by tight or weak metals would also influence cross-exon pre-mRNA folding and its evolution. Because the number of vertebrate exons encoding metal binding sites is very high, exonic enhancers and silencers may have played an important role in reducing mismetallation during evolution by promoting weak binders into mature transcripts while excluding metals at the top of the affinity series.

### *OGDH* dBPs/megaPPT *en route* to endothermy

Assuming the validity of aerobic scope model (5), we propose that selection for more efficient and specialized *OGDH* MXE usage facilitated early evolution of endothermy by favouring organisms capable of maximizing sustained NADH and ATP supply in response to Ca^2+^ signals generated by striated muscles. The hypothesis is supported by the (i) crucial position of E1 in Ca^2+^-responsive energy supply pathways (Introduction and Discussion S1); (ii) regulation of E1 by Ca^2+^_m_ in the TCA cycle, ensuring activity-dependent NADH and ATP provision (9,20,33,185); (iii) key role of intron 4a and dBP/megaPPT organization in regulating the balance of Ca^2+^-sensitive and -insensitive *OGDH* isoforms (Figures 1, 2 and 4); (iv) evolution of intron 4a size restriction, dBP/megaPPT organization and OGDHC activity in endotherms, ectotherms and intermediate phenotypes (Figures 1, 3, 6, Supplementary Figure S7, S13, S15); (v) critical contribution of mitochondria in striated muscles and Ca^2+^ signaling to heat generation and heterothermy (186, 187 and Discussion S2); (vi) OGDHC role in hypometabolic states such as torpor and hibernation (Discussion S3); (vii) self-promoting activity of exon 4b coding for the DADLD motif, contributing to the ubiquitous expression of dBP-led isoform 4b+ (Figure 10, Supplementary Figures S8−S10) and (viii) exon 4b repression in non-muscle cells, such as neurons, limiting oversupply and excitoxicity (Discussion S4).

First shown for muscle contraction (114,188), Ca^2+^_c_ is now recognized as a universal regulator in many biological functions, including secretion, vision, division and fertilization (reviewed in 123). These traits have been subject to strong natural selection that favoured organisms moving faster and farther, gathering food more effectively, provisioning more successfully for the developing young, seeing better and growing and reproducing more rapidly, often through selective advantages conferred by one mRNA isoform over another. As the best communicator in the cell and an allosteric metal *par excellence* (189), Ca^2+^_c_ has therefore a potential to unify rival concepts for the evolution of endothermy that have emerged so far (2–4), including a modified aerobic scope (3), parental care (190,191) and body mass (192) (Discussion S5) hypotheses. The extent to which *OGDH* MXEs and other candidate alternatively spliced isoforms (Discussion S6−S8) acted as drivers of the central metabolic conversion in animal history should be evaluated in more detail in future studies.

## DATA AVAILABILITY

dBP RNA-seq data are available from the Array Express (accession number E-MTAB-9412).

## Supplementary Material

gkab046_Supplemental_Files
