# Supplementary material for: Restriction of an intron size en route to endothermy
Source: Nucleic Acids Res. 2021 Feb 8;49(5):2460–87. doi: 10.1093/nar/gkab046 (PMC7969005; doi:10.1093/nar/gkab046)
Supplement: gkab046_Supplemental_Files [file gkab046_supplemental_files.zip › OGDH MXE SUPPLEMENT - REVISED.pdf]

SUPPLEMENTAL INFORMATION

Restriction of an intron size *en route* to endothermy

SUPPLEMENTAL TABLES

Table S1 Synthetic nucleic acids

Oligo(ribo)nucleotide	5'-3' sequence[1,2]
***OGDH* splicing reporter constructs**	
Human-F (*Homo sapiens*)	ATTACTCGAGTTTGGTTTCTGGTGGCATGG
Human-R	CAGGTTAGCAGCACAGTGAC
Opossum-F (*Monodelphis domestica*)	ATTACTCGAGAAGCCTCTGGGTAAAGGT
Opossum-R	ATTATCTAGAAGGTTCTTGGGGAAAGC
Platypus-F (*Ornithorhynchus anatinus)*	ATTACTCGAGTGCAGCTCTAAGGCTTCCTC
Platypus-R	ATTATCTAGAGCCAGGTATCGGTCCAAC
Chicken-F-large *(Gallus gallus)*	ACCACTCGAGAACTGCTGACCCTGAGATTTG
Chicken-F-small	ACCACTCGAGGAGCACTGCTGAGCTCTGTG
Chicken/quail-R	ACCATCTAGACGTCCAGACAGACAGACACAC
Quail-F	ATTACTCGAGCCTCTGCACTCCCTCTGCTA
Python-F (*Python molurus bivittatus)*	ATCACTCGAGAGCAGCTGGACAATGGAAAT
Python-R	ATCATCTAGACTCTTTCCCCACTCCTTCCT
Frog-F (*Xenopus laevis*)	ATTACTCGAGAATTGTGGTTAGTTGAATATTGCTC
Frog-R	ATTATCTAGATAACGCCAAGAGGGATCGT
Zebrafish-F (*Danio rerio*)	ATTACTCGAGCTGAGCCGGGATAAAGACTG
Zebrafish-R	ATTATCTAGACAGGACAGCTCAAGCATCAA
Cod-F (*Gadus morhua*)	ATTACTCGAGTCAAATGAATTTATAGCGTTTTGG
Cod-F	ATTATCTAGATTTTTCCATTCCTTACATTTCCA
Sunfish-F1-large (*Mola mola*)	ACCACTCGAGAAACAGCCTGCCGAAACTTA
Sunfish-F2-small	ACCACTCGAGTTACTGCACACGCTCAGAATG
Sunfish-R	ACCATCTAGACCACCCTTTCAAACAGCAAT
Human OGDH-e5+-F (with exon 5)	ATCATCTAGACACCTGAATCTAGTGCTGAGAAA
Human OGDH- e5+-R (with exon 5)	ATCATCTAGACAAGCCACCATGCAGAG
**Cloning/subcloning of expression plasmids**	
PTB4-Bam	ATTAGGATCCGCCATGGACGGCATTGTCCCA
PTB4-Xho	ATTACTCGAGGATGGTGGACTTGGAGAAGGA
TIA-1-Bam	ATTAGGATCCGCCATGGAGGACGAGATGCC
TIA-1-Xho	ATTACTCGAGCTGGGTTTCATACCCTGCCA
TIAR-Bam	ATTAGGATCCACCATGATGGAAGACGACG
TIAR-Xho	ATTACTCGAGCTGTGTTTGGTAACTTGCC
**TIA-1 RRM2 mutagenesis**	
F98A	CATTTCCATGTCGCTGTTGGTGATCTC
F140A	AAGGGATATGGCGCTGTCTCCTTTTTC
**TIA-1 Q-domain mutagenesis**	
N357S	GGATGGGACCAAGTTATGGAGTGC
E384K	TGGCAGGGTATAAAACCCAGCTC
**Mapping of *H. sapiens* dBPs**	
R1-HS *(U2AF1)*	TCGATCACCTGCCTCACTATT
R2-HS *(U2AF1)*	CACAAATGGAAAATACAACTACGAGA
F1-HS *(OGDH)*	CACAGCTCACTGGATCTGC
F2-HS *(OGDH)*	AGGTCAGGGGTCACCACATT
**Mapping of *G. gallus* dBPs**	
F1-GG	TCTGTATTGTTACTTCCAGGTCAG
F2-GG	ATTGCAAAGCTCGATCCTCTC
R3-GG	TCCTCTCGGCATTAGTTGTGTA

**Mapping of *X. laevis* dBPs**	
F1-XL	CTTCCAGGTGAGGGGTCAT
F2-XL	CCCGCTTGGAATTAGTTCTG
R2-XL	TCACATTGCAAAGCTTGACC
**Mapping of *M. mola* exon 4b BP**	
F1-MM	TTGGATCCTCTGGGAATCAG
F2-MM	TTGATGATGCTCCGGTGAA
R2-MM	ACAAAGTTTCCGGTCTGTGG
***DBR1* depletion**	
DBR1 siRNA-1	[GCAUGCAAGGUGGGAUUAU]TT
DBR1 siRNA-2	[GCAUCAGGCAAAGGAUAAA]TT
**Depletion of TIA proteins**	
TIA-1 siRNA	[AAGCUCUAAUUCUGCAACUCU]TT
TIAR siRNA	[AACCAUGGAAUCAACAAGGAU]TT
**BP mutagenesis**	
OG-BP+25	GCTGTAAATGCTGGTTTTAATGTAAT
OG-BP+31	AATGCTAATTTTGGTGTAATTTTACT
OG-BP+36	CTAATTTTAATGTGGTTTTACTTTTTT
OG-BP+31+36	AATGCTAATTTTGGTGTGGTTTTACT
OG-BP+25+31	AATGCTGGTTTTGGTGTAATTTTACT
OG-ins-BP28F	TGTGTGTCTACTAACCTTCCCTCTCATCGTT
OG-ins-BP28R	GAGGGAAGGTTAGTAGACACACACAAAAGGAC
OG-ins-BP41F	CTTCTGTCCTACTAACTTTTGTGTGTGTCCTTC
OG-ins-BP41R	ACAAAAGTTAGTAGGACAGAAGAAAAAAAAAGA
OG-ins-TAA28	CTTTTGTGTGTGTCTAACTTCCCTCTCAT
OG-ins-TAA41	TTTTTCTTCTGTCCTAATTTTGTGTGTGTCC
OG-ins-TGA28	CTTTTGTGTGTGTCTGACTTCCCTCTCATC
OG-ins-TGA41	TTTTTCTTCTGTCCTGATTTTGTGTGTGTCC
XL-mBP(TAA>TGG)	AGACTTGTTGTGGCGTTCTCTTTTC
**RT-PCR primers for exogenous expression**	
35E1+PL4	CAGGTGCTCTCGGTTGCA
35m-amplF	GCTCGGATCCTACAGAGTCAA
PL4	AGTCGAGGCTGATCAGCGG
**Restriction site insertions for MIR cloning**	
Pst+15	AGAATTAAGCTGCAGTAAATGCTAATT
EcorV+65F	CCCTCTTTTGATATCTTTTTCTTCTGTCCTT
EcorV+65R	AGAAAAAGATATCAAAAGAGGGAAGGGGT
**Amplification of the MIR library**	
MIR-F-PstI	CAGTCCTGCAG(A/G)CAG(C/T)A(C/T)AG(C/T)ATAGTGGTTAAGAGCAC
MIR-R-PstI	CAGTCCTGCAGTAATA(G/A)C(C/T)AACATTTATTGAGCGCTT
MIR-F-EcoRV	CAGTCGATATC(A/G)CAG(C/T)A(C/T)AG(C/T)ATAGTGGTTAAGAGCAC
MIR-R-EcoRV	CAGTCGATATCTAATA(G/A)C(C/T)AACATTTATTGAGCGCTT
**Mapping of MIR BPs**	
BP-MIR15-RT/R1	TACATTAAAATTAGCATTTACAGCTT
BP-MIR15-F1	ATTGAGCGCTTACTGCATTG
BP-MIR15-F2	GCATTGCCATGAATTGCTTT
BP-MIR15-R2	AATTAGCATTTACAGCTTAATTCT
**MegaPPT deletions**	
OG-del1F	TGTAATTTTACTCTTTTTTTTTCTTCTGTCCT
OG-del1R	GAAAAAAAAAGAGTAAAATTACATTAAAATTAGCAT
OG-del2F	TTACCCCTTCCCTTTTGTGTGTGTCCTTCCC
OG-del2R	CACACACAAAAGGGAAGGGGTAAAAAAAAAG
OG-del3F	TTCTTCTGTCCCCTTCCCTCTCATCGTTG
OG-del3R	TGAGAGGGAAGGGGACAGAAGAAAAAAAAAGAGG
OG-del4F	TTTTGTGTGTGTGTTGGCCACTCATAGATACG
OG-del4R	TGAGTGGCCAACACACACACACAAAAGGACAGA
**Exon variants**	
INS-TCC(HS>XL)	TAACTGTTTCTTCTCCAAACGTGGGTGAGAATT
4a-5T(HS>F)	ACTGTTTCTTCAAATGTGGGTGAGAATT
4a-2T (5’SS)	GTTTCTTCAAACGTTGGTGAGAATTAAGC
4a-2C (5’SS)	GTTTCTTCAAACGTCGGTGAGAATTAAGC
4b+3T	GGCCACTCATAGAT(T/C)CGAGGGCACCAT
4b+18T	GAGGGCACCATGT(T/G)GCACAGCTGGACCC
4b+33T	GCACAGCTGGACCC(A/T)CTGGGGATTTTGGAT

4b+78T	TCCGTGCCCGCTGATATTATCTCATCCACA
4b+91T	ACATTATCTCATCCTCAGACAAACTTGGT
H>XL-(insGGA)	CTCCAGTAACTGTTGGATCTTCAAACGTGGGT
H>F-(insGGC)	CTCCAGTAACTGTTGGCTCTTCAAACGTGGGT
XL>H-(delGGA)	CTCCTGTGATAGTCTCTCCAAATATGG
**RT-PCR with endogenous *OGDH* transcripts**	
Human-F (*Homo sapiens*)	GCACAGTCCCTGGTAGAAGC
Human-R	GCAGAGGAAGTGCTGATTCC
Rat-F (*Rattus norwegicus*)	AGCCTAACGTCGACAAGCTC
Rat-R	GGTGGTGGGTAAGTGGAAGA
Opossum-F (*Monodelphis domestica*)	AGTGGCTCCCTGTCATCACT
Opossum-R	AAGTGGAAGGGCAGATTCCT
Echidna-F (*Tachyglossus aculeatus*)	CCGGGTACAGCCTACCAAAG
Echidna-R	CCGTCCCTCCAATGAAAGT
Platypus-F (*Ornithorhinchus anatinus*)	CCGGGTACAGCCTACCAAAG
Platypus-R	CCGTCCCTCCAATGAAAGT
Quail-F (*Coturnix japonica*)	GGGACATCTTCTTTCGCAAC
Quail-R	GTTGTGGGCAAATGGAAGAC
Frog-F (*Xenopus laevis*)	GCAGTCTGTCCACCCTTACC
Frog-R (target shared by *Ogdh* and *Ogdhl*)	AGTGGTCGGGAGATGGAAGA
Zebrafish-F (*Danio rerio*)	GTGGAAGACCATCTGGCAGT
Zebrafish-R	GGCAAGCGGAACACTTTATC
Ogdhl-Xenopus laevis-F	GCAGTCTGTCCACCCTTTCG
**Lengthening of *M. mola* AGEZ**	
molaAG>CG	GTGTTGATGTTGCGCTTTCTTTCTGT
molaAG>TG	GTGTTGATGTTGTGCTTTCTTTCTGT
molaAG>GG	GTGTTGATGTTGGGCTTTCTTTCTGT
**DADLD mutagenesis**	
HADLD	CTGGGGATTTTGCACGCTGATCTGGACT
DAHLD	GGATTTTGGATGCTCACCTGGACTCCTCC
DADLH	GGATGCTGATCTGCACTCCTCCGTGC
**Templates for RNA structural probing**	
T7F-Human	TAATACGACTCACTATAGGGAGAGGGTGAGAATTAAGCTG
R-RT-Human	GAACCGGACCGAAGCCCGGGGTAAAAAAAAAGTAAAATTA
T7F-Chicken	TAATACGACTCACTATAGGGAGACGGTGAGAATTACTCTG
R-RT-Chicken	GAACCGGACCGAAGCCCGGGGAGGGGTAAAAAAGTT
T7F-link-Human	TAATACGACTCACTATAGGGAGAGGCCTTCGGGCCAATTCAAACGTGGGTGAGAA
R-link-Human	GAACCGGACCGAAGCCCGATTTGGATCCGGCGAACCGGATCGAGGGTAAAAAAAAAGTAAAATTACATTA
T7F-link-Chicken	TAATACGACTCACTATAGGGAGAGGCCTTCGGGCCAATCCAAACGTCGGTGAGAA
R-link-Chicken	GAACCGGACCGAAGCCCGATTTGGATCCGGCGAACCGGATCGAGGGAGGGGTAAAAAAGTTAAATTACA
Cy5-labelled universal primer	GAACCGGACCGAAGCCCG

[1]Degenerate primer positions are in parentheses. [2]T7 promoter is highlighted in grey; linker sequences are underlined.

Table S2 The number of BP-like URA motifs in consensus sequences of mammalian interspersed repeats (MIRs)

MIR subfamily[1]	Sense orientation[2]	Antisense orientation[2]
MIR (260)	13 (5.0)	13 (5.0)
MIRc (268)	12 (4.6)	11 (4.2)
MIR3 (224)	10 (3.8)	5 (1.9)

[1]Consensus sequences were retrieved from RepBase (1); their length (in nts) is in parentheses. [2]Densities (%) of URA motifs are in parentheses.

Table S3 Characterization of MIR insertions in *OGDH* reporters

MIR clone	Restr. site	PCR product	Insert size (nt)	MIR orientation	Sequence of transcribed MIRs (5'-3')[1]
1	PstI	T	193	sense	CUGCAGGCAGUACAGUAUAGUGGUUAAGAGCACAAGCUCCCCAGCCUUGCUCUGCACUUGGCAACUCUGCCUGUUUUCUCUCUGUAGAUAAUCAUAUCACCUGUCUUUGUGUACUGCUGUGAGGAUUAAAUAAUCAAUGCAUCUAAAGUUUAAAACAAUACCCGGCUCACAGAAAGCGCUCAAUAAAUGUUAGCUAUUACUGCAG
2	PstI	F	209	sense	CUGCAGGCAGCAUAGUAUAGUGGUUAAGAGCACAGGCUCUCAGCUUUGUCACUUAAUAGCUAUACGACCUAUUUAACCUUUCUGUGACUUAGUUUGCUCAUCUGUAAAGUGGGGCUACUAAUAGUAGCCUCAUAAGAUUGUGAGGACUCAGUGAGGUAAUGCAAGUCAGAAAAGUAUCAGGCACACAGUAAGCGCUCAAUAAAUGUUGGCUAUUACUGCAG
3	PstI	D	209	sense	CUGCAGGCAGCACAGUAUAGUGGUUAAGAGCACAGGCUCUCAGCUUUGUCACUUAAUAGCUAUACGACCUAUUUAACCUUUCUGUGACUUAGUUUGCUUAUCUGUAAAGUGGGGCUACUAAUAGUAGCCUCAUAAGAUUGUGAGGACUCAGUGAGGUAAUGCAAGUCAGAAAAGUAUCAGGCACACAGUAAGCGCUCAAUAAAUGUUAGCUAUUACUGCAG
4	PstI	N	209	antisense	CUGCAGUAAUAGCUAACAUUUAUUGAGCGCUUACUGUGUGCCUGAUACUUUUCUGACUUGCAUUACCUCACUGAGUCCACACAAUCUUAUGAGGCUACUAUUAGUAGCCCCACUUUACAGAUGAGCAAACUAAGUCACAGAAAGGUUAAAUAGGUCGUAUAGCUAUUAAGUGACAAAGCUGAGAGCCUGUGCUCUUAACCACUAUACUAUACUGCCUGCAG
5	PstI	R	209	antisense	CUGCAGUAAUAGCUAACAUUUAUUGAGCGCUUACUGUGUGCCUGAUACUUUUCUGACUUGCAUUACCUCACUGAGUCCUCACAAUCUUAUGAGGCUACUAUUAGUAGCCCCACUUUACAGAUGAGCAAACUAAGUCACAGAAAGGUUAAAUAGGUCGUAUAGCUAUUAAGUGACAAAGCUGAGAGCCUGUGCUCUUAACCACUAUACUGUACUGCCUGCAG
6	PstI	O	209	antisense	CUGCAGUAAUAGCUAACAUUUAUUGAGCGCUUACUGUGUGCCUGAUACUUUUCUGACUUGCAUUACCUCACUGAGUCCUCACAAUCUUAUGAGGCUACUAUUAGUAGCCCCACUUUACAGAUAAGCAAACUAAGUCACAGAAAGGUUAAAUAGGUCGUAUAGCUAUUAAGUGACAAAGCUGAGAGCCUGUGCUCUUAACCACUAUGCUGUACUGCCUGCAG
7	PstI	P	193	antisense	CUGCAGUAAUAACUAACAUUUAUUGAGCGCUUUCUGUGAGCCGGGUAUUGUUUUAAACUUUAGAUGCAUUGAUUAUUUAAUCCUCACAGCAGUACACAAAGACAGGUGAUAUGAUUAUCUACAGAGAGAAAACAGGCAGAGUUGCCAAGUGCAGAGCAAGGCUGGGGAGCUUGUGCUCUUAACCACUAUACUAUACUGCCUGCAG
8	PstI	E	193	antisense	CUGCAGUAAUAGCCAACAUUUAUUGAGCGCUUUCUGUGAGCCGGGUAUUGUUUUAAACUUUAGAUGCAUUGAUUAUUUAAUCCUCACAGCAGUACACAAAGACAGGUGAUAUGAUUAUCUACAGAGAGAANACAGGCAGAGUUGCCAAGUGCAGAGCAAGGCUGGGGAGCUUGUGCUCUUAACCACUAUACUAUGCUGCCUGCAG
9	PstI	A	193	antisense	CUGCAGUAAUAGCUAACAUUUAUUGAGCGCUUUCUGUGAGCCGGGUAUUGUUUUAAACUUUAGAUGCAUCGAUUAUUUAAUCCUCACAGCAGUACACAAAGACAGGUGAUAUGAUUAUCUACAGAGAGAAAACAGGCAGAGUUGCCAAGUGCAGAGCAAGGCUGGGGAGCUUGUGCUCUUAACCACUAUACUGUGCUGCCUGCAG
1	EcoRV	4_E	262	sense	GAUAUCGCAGUACAGCAUAGUGGUUAAGAGCACUAGGCNUUCGGAGUUUUAACUGCCUGUGGUUUAAAUCAGUGCUCUACCACUUACUAGCUAUGUGACCUUGAGCAAGUAUCUUAACUUCUCUGACCUCAGUUUCUUCAUAUAUUAUCCCCCAAAUUGGGGAUAAUAAUAGUAUGCUCUUUGUAGGAUAACUUAAGAUAGCUGCAGAUAAUUAAGGCUUGCAGCUUCGCCUGACAUAUAGUAAGCGCUCAAUAAAUGUUAGCUAUUAGAUAUC
2	EcoRV	3_X	209	sense	GAUAUCGCAGUACAGUAUAGUGGUUAAGAGCACAGGCUCUCAGCUUUGUCACUUAAUAGCUAUACGACCUAUUUAACCUUUCUGUGACUUAGUUUGCUCAUCUGUAAAGUGGGGCUACUAAUAGUAGCCUCAUAAGAUUGUGAGGACUCAGUGAGGUAAUGCAAGUCAGAAAAGUAUCAGGCACACAGUAAGCGCUCAAUAAAUGUUAGCUAUUAGAUAUC
3	EcoRV	3_F	209	sense	GAUAUCGCAGUAUAGUAUAGUGGUUAAGAGCACAGGCUCUCAGCAUUGUCACUUAAUAGCUAUACGACCUAUUUAACCUUUCUGUGACUUAGUUUGCUUAUCUGUAAAGUGGGGCUACUAAUAGUAGCCUCAUAAGAUUGUGAGGACUCAGUGAGGUAAUGCAAGUCAGAAAAGUAUCAGGCACACAGUAAGCGCUCAAUAAAUGUUAGCUAUUAGAUAUC
4	EcoRV	4_I	249	sense	GAUAUCGCAGUAUAGCAUAGUGGUUAAGAGCACAAGCCCAGAAGCAAUUUUGCCAGGGUCAAAUACUGGUUUCACUACUUUCUUGCCACAUGACUCUGGGCAAGAACGUUAUCUUGGCCUUUUUCCAUCCGUAAACAUGAGGAUAAUAAUGGUGCCUACCUCAUGGGACUGCAGUAAGGAUUAAGUGUGGUAACACAUCACAUGAAGUCUUUAAAAUCCAUAACAUACUAAGCGCUCAAUAAAUGUUAGCUAUUAGAUAUC
5	EcoRV	3_K	392	sense	GAUAUCGCAGUACAGUAUAGUGGUUAAGAGCACAAGCUCCCCAGCCUUGCUCUGCACUUGGCAACUCUGCCUGUUUUCUCUCUGUAGAUAAUCAUAUCACCUGUCUUUGUGUACUGCUGUGAGGAUUAAAUAAUCAAUGCAUCUAAAGUUUAAAACAAUACCCGGCUCACAGAAAGCGCUCAAUAAAUGUUGGCUAUUAGAUAUCGCAGCAUAGUAUAGUGGUUAAGAGCACAAGCUCCCCAGCCUUGCUCUGCACUUGGCAACUCUGCCUGUUUUCUCUCUGUAGAUAAUCAUAUCACCUGUCUUUGUGUACUGCUGUGAGGAUUAAAUAAUCAAUGCAUCUAAAGUUUAAAACAAUACCCGGCUCACAGAAAGCGCUCAAUAAAUGUUAGCUAUUAGAUAUC
6	EcoRV	3_A	193	sense	GAUAUCGCAGUAUAGUAUAGUGGUUAAGAGCACUAGCUCCCCAGCCUUGCUCUGCACUUGGCAACUCUGCCUGUUUUCUCUCUGUAGAUAAUCAUAUCACCUGUCUUUGUGUACUGCUGUGAGGAUUAAAUAAUCAAUGCAUCUAAAGUUUAAAACAAUACCCGGCUCACAGAAAGCGCUCAAUAAAUGUUAGCUAUUAGAUAUC
7	EcoRV	3_J	193	sense	GAUAUCGCAGUACAGCAUAGUGGUUAAGAGCACAAGCUCCCCAGCCUUGCUCUGCACUUGGCAACUCUGCCUGUUUUCUCUCUGUAGAUAAUCAUAUCACCUGUCUUUGUGUACUGCUGUGAGGAUUAAAUAAUCAAUGCAUCUAAAGUUUAAAACAAUACCCGGCUCACAGAAAGCGCUCAAUAAAUGUUAGCUAUUAGAUAUC
8	EcoRV	3_D	193	sense	GAUAUCGCAGCAUAGCAUAGUGGUUAAGAGCACAAGCUCCCCAGCCUUGCUCUGCACUUGGCAACUCUGCCUGUUUUCUCUCUGUAGAUAAUCAUAUCACCUGUCUUUGUGUACUGCUGUGAGGAUUAAAUAAUCAAUGCAUCUAAAGUUUAAAACAAUACCCGGCUCACAGAAAGCGCUCAAUAAAUGUUAGCUAUUAGAUAUC
9	EcoRV	H	193	sense	GAUAUCGCAGUAUAGUAUAGUGGUUAAGAGCACAAGCUCCCCAGCCUUGCUCUGCACUUGGCAACUCUGCCUGCUUUCUCUCUGUAGAUAAUCAUAUCACCUGUCUUUGUGUACUGCUGUGAGGAUUAAAUAAUCAAUGCAUCUAAAGUUUAAAACAAUACCCGGCUCACAGAAAGCGCUCAAUAAAUGUUAGCUAUUAGAUAUC
10	EcoRV	4_B	241	antisense	GAUAUCUAAUAGCUAACAUUUAUUGAGCGCUUACCAUGUGCCAGGCACUGAUUAAUAUGUAUUAUUAGCUCAUUUAAUCCUCACAAUAACCCUGUGAAGUAGAAACUAUGUAAGCAGUAAGAAACUGAGGCACCUUGACCAGUAGUGACUUUUGUCCAAGGUAACAUGGCAGCUAAGUGAGAAGCUAGAUAUAAACCCAGGAAAUCUGGCCCUGGAACUUGUGCUCUUAACCACUAUACUGUGCUGCGAUAUC
11	EcoRV	4_C	241	antisense	GAUAUCUAAUAACUAACAUUUAUUGAGCGCUUACCAUGUGCCAGGCACUGAUUAAUAUGUAUUAUUAGCUCAUUUAAUCCUCACAAUAACCCUGUGAAGUAGAAACUAUGUAAGCAGUAAGAAACUGAGGCACCUUGACCAGUAGUGACUUUUGUCCAAGGUAACAUGGCAGCUAAGUGAGAAGCUAGAUAUAAACCCAGGAAAUCUGGCCCUGGAACUUGUGCUCUUAACCACUAUACUAUACUGCGAUAUC
12	EcoRV	3_G	193	antisense	GAUAUCUAAUAGCUAACAUUUAUUGAGCGCUUUCUGUGAGCCGGGUAUUGUUUUAAACUUUAGAUGCAUUGAUUAUUUAAUCCUCACAGCAGUACACAAAGACAGGUGAUAUGAUUAUCUACAGAGAGAAAACAGGCAGAGUUGCCAAGUGCAGAGCAAGGCUGGGGAGCUUGUGCUCUUAACCACUAUACUAUGCUGCGAUAUC
13	EcoRV	4_G	193	antisense	GAUAUCUAAUAACUAACAUUUAUUGAGCGCUUUCUGUGAGCCGGGUAUUGUUUUAAACUUUAGAUGCAUUGAUUAUUUAAUCCUCACAGCAGUACACAAAGACAGGUGAUAUGAUUAUCUACAGAGAGAAAACAGGCAGAGUUGCCAAGUGCAGAGCAAGGCUGGGGAGCUUGUGCUCUUAACCACUAUGCUAUGCUGCGAUAUC
14	EcoRV	I	193	antisense	GAUAUCUAAUAGCUAACAUUUAUUGAGCGCUUUCUGUGAGCCGGGUAUUGUUUUAAACUUUAGAUGCAUUGAUUAUUUAAUCCUCACAGCAGUACACAAAGACAGGUGAUAUGAUUAUCUACAGAGAGAAAACAGGCAGAGUUGCCAAGUGCAGAGCAAGGCUGGGGAGCUUGUGCUCUUAACCACUAUACUAUACUGCGAUAUC
15	EcoRV	4_D	211	antisense	GAUAUCUAAUAGCUAACAUUUAUUGAGCGCUUACUGCAUUGCCAUGAAUUGCUUUACAUGUAUCUCAUAUAAUCCUCUCAGCAAUCCUAUGAAGUUGAUAAUAGAGCCCAUUAAGAAACUUGACCAAAAUCAUCUUGUUAUUAAGUGGUAGAACAAUAUUCAAAUGUAUAUUUGUUUGACAUGAAGACCCGUGCUCUUAACCACUAUACUGUACUGUGAUAUC
16	EcoRV	3_C	209	antisense	GAUAUCUAAUAGCUAACAUUUAUUGAGCGCUUACUGUGUGCCUGAUACUUUUCUGACUUGCAUUACCUCACUGAGUCCUCACAAUCUUAUGAGGCUACUAUUAGUAGCCCCACUUUACAGAUGAGCAAACUAAGUCACAGAAAGGUUAAAUAGGUCGUAUAGCUAUUAAGUGACAAAGCUGAGAGCCUGUGCUCUUAACCACUAUACUGUACUGCGAUAUC

[1] Restriction sites are highlighted in yellow. URA motifs unique to clone 15 are underlined (UGA-181, UAA-178, UAA-165, UAA-135, UGA-100 and UGA-60; each motif is followed by the distance in nts between putative BP-A and the 3'ss). BPs determined in MIR15 transcripts (Figure 4D-F) are in red.

Table S4 Relaxation of *OGDH* intron 4a splice sites in vertebrates had a minimal impact on exon 4a/4b ratios

Species	5'ss of intron 4a[1]	Max. entropy score[2]	Exon 4b inclusion	3'ss of intron 4a[1]	Max. entropy score[2]	Exon 4b inclusion
Placentals	TGGgtgaga	6.04	-	tcatcgttggccactcatagATA	3.36	-
Marsupials	TTGgtgaga	6.29	No change	attgttggcccatttcatagATT	5.11	No change
Birds and reptiles	TCGgtgaga	8.51	Potentially slightly lower	actcctggcccatttcatagATT	6.54	No change

[1]Intron sequences are in lower case, exon sequences are in upper case. [2]Maximum entropy scores (2) were computed for human, wallaby and duck splice sites. Tested mutations are in red. Splicing assays are shown in Figure 5.

Table S5 Amino acid identity between zebrafish and human TIA-1 and PUF60 RRMs

	RRM1	RRM2	RRM3
Human PUF60 *versus* zebrafish PUF60	78/79 (97%)	74/79 (94%)	79/88 (90%)[1]
Human TIA-1 *versus* zebrafish TIA-1	67/77 (87%)	69/79 (87%)	67/73 (92%)

[1]RRM3 is known as the U2AF homology motif or UHM (3).

Table S6 Function of human genes containing MXEs separated by dBP/megaPPT introns[1]

Gene symbol	Length of the intron between MXEs (nts)	SVM dBP score	5'ss to dBP (nts)	Longest PPT U-run 3' of dBP (nts)	Gene product	Function	Human loss-of-function phenotype	Key references
OGDH	122	1.92	25	9	E1 subunit of the 2-oxoglutarate dehydrogenase complex	Ca^{2+}-mediated NADH supply; distinct sensitivity of MXE isoforms to mitochondrial Ca^{2+}	No reliable reports of human mutations; lethal in lower organisms	(4)
KCNMA1	103	-	-	15	K^+ Ca^{2+}-activated channel, subfamily M, α1	Dampens excitatory events that elevate Ca^{2+}_c and/or depolarize the cell membrane; distinct gating characteristics of MXE isoforms; Ca^{2+} sensitivity of splice variants	Dyskinesia, seizures (OMIM # 609446 and 617643)	(5, 6,7)
SNAP25	161	1.74	23	13	Synaptosome associated protein 25	Ca^{2+}-triggered exocytosis and regulation of neurotransmitter release; distinct induction of primed vesicle pool by MXE isoforms	Myasthenia (OMIM # 616330)	(8, 9)
ACAD10	212	1.59	23	20	Acyl-CoA dehydrogenase, 10	Mitochondrial fatty acid metabolism	Not reported[2]	(10)

[1]MegaPPT is defined here by the presence of 8 or more consecutive uridines adjacent to dBPs. This length permits cooperatiave binding of PUF60 and U2AF65 (11) and is close to a limit for in vitro binding of PUF60 (11 pyrimidines) (12). PPTs with longer uridine stretches generally promote the efficiency of 3'ss recognition (13,14); to achieve the same level of splicing, shorter PPT tracts require higher uridine content (15). [2]*Acad10*-deficient mice accumulate excess abdominal adipose tissue, exhibit fasting rhabdomyolysis and have abnormal skeletal muscle mitochondria (16). OMIM #, Online Mendelian Inheritance in Man database ID.

Table S7 MXE responses to PUF60/U2AF65 knockdown in transcripts involved in Ca^{2+} signalling

Human gene symbol	Putative dBPs	PPT in the intron that separates MXEs	~RPKM[4] in HEK293 cells	Effect of PUF60/U2AF65 depletion
ACTN2	+	long, partitioned into UC- and UG-rich segments; UG repeat close to 3'ss	not expressed	-
CACNA1A	+	very long, G(U)n repeat close to 3'ss, $(A)_n$ repeat further upstream	not expressed	-
CACNA1B	+	very long, (C)nU repeats close to 3'ss	not expressed	-
CACNA1C	-	Short	not expressed	-
CACNA1D	-	short, (A)n repeat	not expressed	-
CALU	+?	short and lacks uridines, $(U)_4$ is the longest uridine repeat	1000	no change
DNM2	+	long, partitioned into repeated UC- and UG-rich segments	500	exon b down in PUF60- cells and up in U2AF65-
FYN	-	mid-size; (A)n repeat	500	exon b up in PUF60- cells, down in U2AF65- cells
GNAL[1]	-	NA; ATI	100	first exons sensitive to U2AF65 depletion
GNAS[1]	-	NA; ATI	10000	skipping of a downstream exon in U2AF65- cells
GRIA1	-	short	not expressed	-
GRIA2	-	short	not expressed	-
GRIA3	-	short	not expressed	-
GRIA4	-	short	not expressed	-
IDH3B	-	NA; ATI	500	no change
MASP1[2]	-	NA; APA	not expressed	-
OGDH	+	long, partitioned into U-, C-, UG- and UC-rich segments	200	exon b down in PUF60- cells and up in U2AF65-
OTOF	-	short; MXEs are terminal	not expressed	-
PRKCB	-	NA; ATI	80	no change
PRKG1	-	NA; ATI	not expressed	-
SCN5A	+	long, partitioned into multiple UC- and UG-rich regions	15	not informative, exon b was not used
SLC12A1[3]	+	mid-size	not expressed	-
SLC25A3	-	short	3000	not informative, exon a was not used
SLC25A24	-	NA; ATI	200	no change
SLC8A1	-	mid-size, (U)nG repeats	50	not informative, exon a was not used
SLC8A3	+	mid-size, UC-rich	not expressed	-
SNAP25	+	long, partitioned into (U)nC-rich and GA-rich segments	not expressed	-
TPM2	+	long, partitioned into UC- and C-rich segments	25	exon b down in PUF60- and up in U2AF65-
TPM3	+	long and partitioned	2000	not informative, exon b was not used

[1]Alternative transcription initiation (ATI). [2]Alternative polyadenylation (APA). [3]Three MXEs; dBPs were predicted both for the intron between exon a (5' exon) and b (0.53 kb) and between exon b and c (0.51 kb). [4]RPKM (reads per kilobase of transcript per million mapped reads) data are rounded; full RNA-seq data are available under the ArrayExpress accession number E-MTAB-6010 (17). NA, not applicable.

Table S8 PUF60-regulated exon usage in transcripts that encode mitochondrial proteins

(Microsoft Excel table)

SUPPLEMENTAL FIGURES

Figure S1 Alignment of mutually exclusive *OGDH* exons 4a and 4b and intron 4a in 62 vertebrate species

Intronic sequences are in lower case; exons 4a and 4b are in upper case. dBPs mapped in this study (Figure 3 and 6) are highlighted in green. A tentative dBP+45 is in blue. Conserved adenine residues of amniote dBP clusters are highlighted in yellow. Variants that change their extended BP consensus motifs are in red. Exon variants examined for splicing (Figure 5) are underlined. The AGEZ spoiler in *M. mola* is in magenta. Asterisks denote identical nucleotides. Alignment was carried out with reference sequences (www.ensembl.org) and Clustal Omega (v. 1.2.4.) using exons 4a, introns 4a, exons 4b and 50-nt flanking intronic sequences.

```
human                         gtgttattgcttccagGTCAGGGGTCACCACATTGCAAAACTTGATCCTCTCGGAATTAG
bonobo                        gtgttattgcttccagGTCAGGGGTCACCACATTGCAAAACTTGATCCTCTCGGAATTAG
baboon                        gtgttattgcttccagGTCAGGGGTCACCACATTGCAAAACTTGATCCTCTCGGAATTAG
angola_colobus                gtgttattgcttccagGTCAGGGGTCACCACATTGCAAAACTTGATCCTCTCGGAATTAG
bolivian_squirrel_monkey      gtgttattgcttccagGTCAGGGGTCACCACATTGCAAAACTTGATCCTCTCGGAATTAG
bushbaby                      gtattattgcttccagGTCAGGGGTCACCACATTGCAAAACTTGATCCTCTCGGAATTAG
bamboo_lemur                  gtgttattgcttctagGTCAGGGGTCATCACATTGCAAAACTTGATCCTCTCGGAATTAG
lemur                         gtgttattgcttccagGTCAGGGGTCACCAAATTGCAAAACTTGATCCTCTCGGAATTAG
polar_bear                    gtgttattgcttccagGTCAGGGGTCACCACATTGCAAAACTTGATCCTCTCGGAATTAG
panda                         gtgttattgcttccagGTCAGGGGTCACCACATTGCAAAACTTGATCCTCTCGGAATTAG
horse                         gtattattgcttccagGTCAGGGGTCACCACATTGCAAAACTTGATCCTCTCGGAATTAG
sloth                         gtattgttgcttccagGTCAGGGGTCACCACATTGCAAAACTTGATCCTCTCGGAATTAG
rabbit                        gtgttgtta-ttccagGTCAGGGGTCACCACATTGCAAAACTTGATCCTCTCGGAATTAG
hyrax                         gtattgttgcttccagGTCAGGGGTCACCACATTGCAAAACTTGATCCTCTCGGAATTAG
treeshrew                     gtgttattgcttccagGTCAGGGGTCACCACATTGCAAAGCTTGATCCTCTCGGCATTAG
hedgehog                      gtgttattacttccagGTCAGGGGTCACCACATTGCAAAACTTGATCCTCTCGGAATTAG
microbat                      gtgttattacttccagGTCAGGGGTCACCACATTGCAAAACTTGATCCTCTCGGAATTAG
megabat                       gtgttattgcttccagGTCAGGGGTCACCACATTGCAAAACTTGATCCTCTCGGAATTAG
mouse                         gtgttatta-ttccagGTCAGGGGTCACCACATTGCAAAACTTGATCCTCTCGGAATTAG
kangaroo_rat                  gtgttatta-ttccagGTCAGGGGTCACCACATTGCAAAACTTGATCCTCTCGGAATTAG
blind_mole_rat                gtgttatta-ttccagGTCAGGGGTCACCACATTGCAAAACTTGATCCTCTCGGAATTAG
Damalarand_mole-rat           gtgttgtta-ttccagGTCAGGGGTCACCACATTGCAAAACTTGATCCTCTCGGCATTAG
naked_mole_rat                gtgttgtta-ttccagGTCAGGGGTCACCACATTGCAAAACTTGATCCTCTCGGCATTAG
guinea_pig                    gtgttgtta-ttccagGTCAGGGGTCACCACATTGCAAAGCTTGATCCTCTCGGCATTAG
armadillo                     gtattgttgcttccagGTCAGGGGTCACCACATTGCAAAACTTGATCCTCTCGGCATTAG
Tasm.devil                    gtgttgttgcttctagGTCAGGGGTCATCACATTGCAAAACTTGATCCCCTCGGCATTAG
opossum                       gtattgttgcttccagGTCAGGGGTCACCACATTGCAAAACTTGATCCCCTCGGCATTAG
wallaby                       gtgttgttgcttccagGTCAGGGGTCACCACATTGCAAAACTTGATCCCCTCGGCATTAG
platypus                      atgttgttgctttcagGTCAGGGGTCACCACATTGCAAAGCTCGACCCTCTCGGCATTAG
duck                          gtgttgttgcttccagGTCAGGGGCCATCACATTGCAAAGCTTGATCCTCTCGGCATTAG
zebrafinch                    gtgttgttgcttccagGTCAGGGGTCACCACATTGCAAAGCTTGATCCTCTCGGCATTAG
chicken                       gtattgttacttccagGTCAGGGGCCATCACATTGCAAAGCTCGATCCTCTCGGCATTAG
turkey                        gtattgttgcttccagGTCAGGGGCCATCACATTGCAAAGCTCGATCCTCTCGGCATTAG
quail                         gtgttgttgcttccagGTCAGGGGCCATCACATTGCAAAGCTCGACCCTCTCGGCATTAG
alligator                     atgttgttgcttccagGTCAGGGGTCATCACATTGCAAAACTTGATCCTCTTGGCATTAG
desert_tortoise               gtgttgttgcttccagGTCAGGGGTCACCACATTGCAAAGCTTGATCCTCTCGGCATTAG
turtle_softshell              gtgttgttgcttccagGTCAGGGGTCATCACATTGCAAAGCTTGATCCTCTCGGCATTAG
turtle_painted                gtgttgttgcttccagGTCAGGGGTCACCACATTGCAAAGCTTGATCCTCTCGGCATTAG
tuatara_lizard                atgttgttgcttctagGTCAGGGGTCATCACATTGCAAAGCTTGATCCTCTCGGCATTAG
anole_lizard                  ttgttgttgtttccagGTCAGGGGTCACCACATTGCCAAGCTTGATCCTCTCGGCATTAG
python_Burmese                ttgttgttgtttctagGTCAGGGGTCACCACATTGCCAAGCTTGATCCTCTCGGCATTAG
xenopus_trop.                 ttgtcattgcttccagGTGAGGGGTCATCACATTGCAAAGCTTGACCCGCTTGGAATTAG
xenopus_laevis                ttgtcgttgcttccagGTGAGGGGTCATCACATTGCAAAGCTTGACCCGCTCGGAATTAG
ocean_sunfish                 gtgtgtgtgtgtgtagGTGATGGGGCATCATAATGCCCAGTTGGATCCTCTGGGAATCAG
tilapia                       gtgtgtgtgtgttcagGTGATGGGTCATCACAATGCCCACCTGGATCCACTGGGAATCAG
zebra_mbuna                   gtgtgtgtgtgttcagGTGATGGGTCATCACAATGCCCACCTGGATCCACTGGGAATCAG
stickleback                   gtgtgcgtgcgtgtagGTGATGGGGCATCACAATGCCCAGTTGGATCCTCTGGGAATCAG
climbing_perch                --gtgtgtgtgttcagGTGATGGGGCATCACAATGCCCACCTGGATCCTCTGGGAATCAG
ballan_wrasse                 gtgggtgtgtgtttagGTGATGGGGCATCACAATGCTCAGTTGGATCCTCTGGGAATCAG
swamp_eel                     ctgtgtgtgtgtttagGTGATGGGGCATCACAGTGCCCACTTGGATCCTCTGGGAATCAG
zigzag_eel                    --gtgtgtgtgtttagGTGATGGGGCATCACAATGCCCTGTTGGATCCTCTGCGAATCAG
tetraodon                     gtttgtcagacagcagGGGAGGGGGCATCACAATGCTCGTTTGGATCCTCTGGGAATCAG
tigertail_seahorse            atgtgtgtgtgtgtagGTGATGGGTCATCACAATGCCCACTTGGACCCGCTGGGCATCAG
fugu                          gtttg----tgaacagGTGATGGGGCATCATAATGCTCGTTTGGATCCTCTGGGAATCAG
tongue_sole                   gcgtttctgtgtgtagGTGATGGGGCATCATAATGCCCGTTTGGATCCTCTGGGAATCAG
amazon_molly                  gtgtgtgtgtgttcagGTCCTGGGCCATCACAACGCCCACCTGGACCCTCTGGGGATCAG
shortfin_molly                gtgtgtgtgtgttcagGTCCTGGGCCATCACAACGCCCACCTGGACCCTCTGGGGATCAG
bicolor_damselfish            ---ggtgtatgtttagGTGATGGGCCATCACAATGCCCATCTGGACCCTCTGGGGATCAG
zebrafish                     g---ccttgttttcagGTAATGGGGCATCACAATGCAAACCTTGACCCACTTGGCATCAG
spotted_gar                   a---ccttgtttacagGTGACTGGGCATCACATTGCCAAACTCGATCCTCTGGGGATCAG
channel_catfish               g---ccttgtttccagGTCATGGGGCATCACAATGCCAAGCTCGACCCGCTCGGCATCAG
cod                           attaacaatcacccagGTGATGGGTCACCATAATGCCCACCTGGATCCATTAGGAATCAA
                                           ***     ** ** ** *  **      * ** **  *  * ** *
```

```
human                         TTGTGTAAATTTTGATGATGCTCCAGTAACTGTT---TCTTCAAACGTGGgtgagaatta
bonobo                        TTGTGTAAATTTTGATGATGCTCCAGTAACTGTT---TCTTCAAACGTGGgtgagaatta
baboon                        TTGTGTAAATTTTGATGATGCTCCAGTAACTGTT---TCTTCAAACGTGGgtgagaatta
angola_colobus                TTGTGTAAATTTTGATGATGCTCCAGTAACTGTT---TCTTCAAACGTGGgtgagaatta
bolivian_squirrel_monkey      TTGTGTAAATTTTGATGATGCTCCAGTAACTGTT---TCTTCAAACGTGGgtgagaatta
bushbaby                      TTGTGTAAATTTTGATGATGCTCCAGTAACTGTT---TCTTCAAACGTGGgtgagaatta
bamboo_lemur                  TTGTGTAAATTTTGATGATGCTCCAGTAACTGTT---TCTTCAAACGTGGgtgagaatta
lemur                         TTGTGTAAATTTTGATGATGCTCCAGTAACTGTT---TCTTCAAACGTGGgtgagaatta
polar_bear                    TTGTGTAAATTTTGATGATGCTCCGGTAACTGTT---TCTTCAAACGTGGgtgagaatta
panda                         TTGTGTAAATTTTGATGATGCTCCGGTAACTGTT---TCTTCAAACGTGGgtgagaatta
horse                         TTGTGTAAATTTTGATGATGCTCCAGTAACTGTT---TCTTCAAACGTGGgtgagaatta
sloth                         TTGTGTAAATTTTGATGATGCTCCAGTAACTGTT---TCTTCAAACGTGGgtgagaatta
rabbit                        TTGTGTAAATTTTGATGGTGCTCCGGTAACTGTT---TCTTCAAACGTGGgtgagaatta
hyrax                         TTGTGTAAATTTTGATGATGCTCCAGTAACTGTT---TCTTCAAACGTGGgtgagaatta
treeshrew                     TTGTGTAAATTTTGATGATGCTCCAGTAACTGTC---TCTTCAAACGTGGgtgagaatta
hedgehog                      TTGTGTAAATTTTGATGATGCTCCAGTAACTGTT---TCTTCAAACGTGGgtgagaattg
microbat                      TTGTGTAAATTTTGATGATGCTCCAGTAACTGTT---TCTTCAAACGTGGgtgaggattg
megabat                       TTGTGTAAATTTTGATGGTGCTCCAGTAACTGTT---TCTTCAAACGTGGgtgagaatta
mouse                         TTGTGTAAATTTTGATGATGCTCCGGTAACTGTT---TCTTCAAACGTGGgtgagaatta
kangaroo_rat                  TTGTGTAAATTTTGATGATGCTCCAGTAACTGTT---TCTTCAAACGTGGgtgagaatta
blind_mole_rat                TTGTGTAAATTTTGATGATGCTCCGGTAACTGTT---TCTTCAAACGTGGgtgagaatta
Damalarand_mole-rat           TTGTGTAAATTTTGATGATGCTCCGGTAACTGTT---TCTTCAAACGTGGgtgagaatga
naked_mole_rat                TTGTGTAAATTTTGATGATGCTCCGGTAACTGTT---TCTTCAAACGTGGgtgagaatga
guinea_pig                    TTGTGTAAATTTTGATGATGCTCCGGTAACTGTT---TCTTCAAACGTGGgtgagaatga
armadillo                     TTGTGTAAATTTTGATGATGCTCCGGTAACTGTT---TCTTCAAACGTGGgtgagaattc
Tasm.devil                    TTGTGTAAATTTTGATGATGCACCAGTAACTGTT---TCTTCAAACGTTGgtgagaatta
opossum                       TTGTGTAAATTTTGATGATGCACCAGTAACTGTT---TCTTCAAACGTTGgtgagaatta
wallaby                       TTGTGTAAATTTTGATGATGCACCAGTAACTGTT---TCTTCAAACGTTGgtgagaatta
platypus                      TTGTGTAAATTTTGATGAGGCTCCAATAACTGTT---TCTCCGAACGTCGgtgagaatta
duck                          TTGTGTAAATTTTGATGATGCACCAGTAACTGTT---TCTCCAAACGTCGgtgagaattg
zebrafinch                    TTGTGTAAATTTTGATGATGCGCCAGTAACTGTT---TCTCCAAACGTCGgtgagaatta
chicken                       TTGTGTAAATTTTGATGATGCGCCCGTAACTGTT---TCTCCAAACGTCGgtgagaatta
turkey                        TTGTGTAAATTTTGATGATGCACCTGTAACTGTT---TCTCCAAACGTCGgtgagaatta
quail                         TTGTGTAAATTTTGATGATGCGCCCGTAACTGTT---TCTCCAAACGTCGgtgagaatta
alligator                     CTGTGTAAATTTTGATGATTCGCCAGTAACTGTT---TCTCCAAACGTCGgtgagaattg
desert_tortoise               TTGTGTAAATTTTGATGATGCACCAGTAACTGTT---TCTCCAAACGTCGgtgagaatta
turtle_softshell              TTGTGTAAATTTTGATGATGCACCAGTAACTGTT---TCTCCAAACGTCGgtgagaatta
turtle_painted                TTGTGTAAATTTTGATGATGCACCAGTAACTGTT---TCTCCAAACGTCGgtgagaatta
tuatara_lizard                TTGTGTAAATTTTGATGATGCACCAGTAACAGTT---TCTCCAAACGTCGgtgagaataa
anole_lizard                  TTGTGTAAATTTTGATGATTCAGCAGTTTCTGAT---TCTCCAAACGTCGgtgagaatta
python_Burmese                TTGTGTAAATTTTGATGATTCGACAGTAACTGAT---TCTCCAAACGTTGgtgagaatta
xenopus_trop.                 TTCTGTTAATTTTGATGGGGCTCCGGTGATAGTCGGATCTCCAAATATGGgtgagaatca
xenopus_laevis                TTCTGTTAATTTTGATGGGGCTCCTGTGATAGTCGGATCTCCAAATATGGgtgagaatcg
ocean_sunfish                 CTGTGTGAATTTTGATGATGCTCCGGTGAATCCCGGCTTCCAGGACGTCGgtgagaacac
tilapia                       CTGTGTGAATTTTGATGATGCTCCGGTGAATGCCGGCTTCCAGGATGTCGgtgagagcgc
zebra_mbuna                   CTGTGTGAATTTTGATGACGCTCCGGTGAATGCCGGCTTCCAGGATGTTGgtgagagcgc
stickleback                   CTGTGTGAATTTTGATGATGCTCCGGTGAATTCCGGCTTCCAGGATGTCGgtgaga-caa
climbing_perch                CTGTGTGAATTTTGATGATGCTCCGGTCAATACCGGCTTCCAGGATGTTGgtgagaacac
ballan_wrasse                 CTGTGTGAATTTTGATGATGCTCCGGTCAATAGCGGCTTCCAGGACGTCGgtgaga-caa
swamp_eel                     CTGTGTGAATTTTGATGATGCGCCGGTCAGTACCGGCTTCCACGACGTCGgtgagaacgg
zigzag_eel                    CTGTGTGAATTTTGATGATGCTCCGGTCAATGCCGGCTTCCAGGACGTCGgtgagaacag
tetraodon                     CTGTGTGAATTTTGATGATGCTCCAGTCAGTACTGGCTTCCAGGACGTTGgtgag--aag
tigertail_seahorse            CTGTGTGAATTTTGATGACGCTCCCGTCAATAGCGGCTTCCAAGATGTTGgtgagcaa--
fugu                          CTGTGTGAATTTTGATGATGCTCCAGTTAATACTGGCTTCCAGGATGTTGgtgagg-aag
tongue_sole                   CTGTGTGAATTTTGATGATGCTCCAGTCAATACTGGTTTCCAGGATGTTGgtgagaacga
amazon_molly                  CTGTGTGAATTTTGACGACGCTCCGGTCAGCGCCGGCTTCCAGGACGTCGgtgagac---
shortfin_molly                CTGTGTGAATTTTGACGACGCTCCGGTCAGCGCCGGCTTCCAGGACGTCGgtgagac---
bicolor_damselfish            CTGTGTGAATTTTGATGATGCTCCGGTCAATACCGGCTTCCAGGACGTTGgtgagaactt
zebrafish                     TTGTGTAAATTTTGACGAGGCCCCAGTAGCTACTGGGTTCCAGCATGTTGgtgagaaatg
spotted_gar                   TTGTGTAAATTTTGACGATGCCCCAGTAACAGTCGGGTTCCCGAATGTTGgtgagaaata
channel_catfish               TTGTGTAAATTTTGACGAAGCCCCAGTAGCTACTGGTTACCAGCATGTTGgtgagaaatg
cod                           CTCTGTGAATTTTGAGGATGTCCCCGCCTGTTCAGAGATCATGGACGTGGgtgagagccc
                               * *** ******** *      *                    *  * ******
```

```
human                        a---g-ct------gtaaatgctaattttaa-----------------------------
bonobo                       a---g-ct------gtaaatgctaattttaa-----------------------------
baboon                       a---g-ct------gtaaatgctaattttaa-----------------------------
angola_colobus               a---g-ct------gtaaatgctaattttaa-----------------------------
bolivian_squirrel_monkey     a---g-ct------gcaaatgctcattttaa-----------------------------
bushbaby                     a---t-ct------gtaaatactaattttaa-----------------------------
bamboo_lemur                 a---t-ct------gtaaatgctaattttaa-----------------------------
lemur                        a---t-ct------gtaaatgctaattttaa-----------------------------
polar_bear                   a---t-ct------gtaaacgctaattttaa-----------------------------
panda                        a---t-ct------gtaaacgctaattttaa-----------------------------
horse                        a---t-ct------gtaaacagtaattttaa-----------------------------
sloth                        a---t-ct------gaaaacagtaattttaa-----------------------------
rabbit                       a---c-ct------gtaaacactaattttaa-----------------------------
hyrax                        a---t-ct------gcaaatgctaattttaa-----------------------------
treeshrew                    a---t---------gcaaatgctcattttaa-----------------------------
hedgehog                     a---t-ct------gtaaatgataattttaa-----------------------------
microbat                     a---t-ct------gtaaatactaattttaa-----------------------------
megabat                      a---t-ct------gtaaacgctaattttaa-----------------------------
mouse                        a---t-at------gtaaatgctaattttaa-----------------------------
kangaroo_rat                 a---t-ct------gtaaatgctaattttaa-----------------------------
blind_mole_rat               a---t-at------gtaaatgctaattttaa-----------------------------
Damalarand_mole-rat          a---t-ct------gtaaatgctaattttaa-----------------------------
naked_mole_rat               a---t-ct------gtaaatgctaattttaa-----------------------------
guinea_pig                   a---t-ct------gtaaatgctaattttaa-----------------------------
armadillo                    a---t-ct------gaaaacgctaattttaa-----------------------------
Tasm.devil                   c---t-ct------gcaaa--ctaattttaa-----------------------------
opossum                      c---t-ct------gcaaa--ctaattttaa-----------------------------
wallaby                      c---t-ct------gcaaa--ctaattttaa-----------------------------
platypus                     g---t-ct------gcaaa--ttcattggaa-----------------------------
duck                         c---t-ct------gcaaa--ttaattctaa-----------------------------
zebrafinch                   c---t-ct------gcaaa--ttaattctaa-----------------------------
chicken                      c---t-ct------gcaaa--ttaattcgaa-----------------------------
turkey                       c---t-ct------gcaaa--ttaattcgaa-----------------------------
quail                        c---t-ct------gcaaa--ttaatttgaa-----------------------------
alligator                    a---t-ct------gccaat-ttaatttgaa-----------------------------
desert_tortoise              c---t-ct------gcaaa--ttaattagaa-----------------------------
turtle_softshell             c---t-ct------gcaaa--ttaattcgaa-----------------------------
turtle_painted               c---t-ct------gcaaa--ttaattcgaa-----------------------------
tuatara_lizard               c---t-ct------gcaaa--ttaattctaa-----------------------------
anole_lizard                 c---t-ct------gcaaa--ttaattctaa-----------------------------
python_Burmese               t---t-ct------gcaag--ttaattttaa-----------------------------
xenopus_trop.                t---t-tt------gtcaga-cttgttgtaa--ctttttctctttttcctgtgctttttc
xenopus_laevis               t---t-gt------gttaga-cttgttgtaa--cgttctc----ttttctgtgctttttc
ocean_sunfish                c---a-------------gagctaaaacaa----agtttc----------------cggt
tilapia                      c---g-------------caactaaaccaccaa------a----------------caga
zebra_mbuna                  c---a-------------caactaaaccaccaa------a----------------caga
stickleback                  c---a-------------ggactgagaagact----attg----------------tggc
climbing_perch               c---a-------------gaactaaaaagcaaactgcttc----------------cagt
ballan_wrasse                c---a-------------gagctgaaaaacaaactgcttc----------------cagt
swamp_eel                    c---a-------------gagctaacaaa----ct----g----------------cttc
zigzag_eel                   c---a-------------gagctaacaaacaaacaaactg----------------cttc
tetraodon                    c---a-------------gcagtagaaa-----ccagttg----------------gagt
tigertail_seahorse           a---a-------------gacggaaaa------gtgctcc----------------gtgc
fugu                         c---a-------------gcagtaggaa-----ccagttt----------------gagt
tongue_sole                  c---a-------------aacttacaaa------taatcc----------------aggc
amazon_molly                 ----------------------gccagaacagaac---ca----------------g-aa
shortfin_molly               ----------------------gccagaacagaac---ca----------------g-aa
bicolor_damselfish           g---a-------------cgacgaaacaacaaacc---ga----------------caga
zebrafish                    ttttctt----tcccccacagctcaacagacc-gccat--------taat------gagc
spotted_gar                  ttcct-ct------gcagctgctctgcgcaaa-cgaacgat---------------gaac
channel_catfish              ttttttctgtcgcctccacagctctgcagaac-atcat--------taat------gagc
cod                          t---c----------------------------------c----------------catg
```

```
human                        --------tgtaattt-tactt-------t---tt----ttttac----ccctt------
bonobo                       --------tgtaattt-tactt-------t---t-----ttttac----ccctt------
baboon                       --------tgtaattt-tactttttt---t---tt----ttttac----ccctt------
angola_colobus               --------tgtaactt-tactttttttt-t---tt----ttttac----ccctt------
bolivian_squirrel_monkey     --------tgtaattt-tacttt------t---tt----ttttac----ccctt------
bushbaby                     --------tgtaattt-tgcttttt----t---tt----ttttac----ccctt------
bamboo_lemur                 --------tgtaattt-tactttt-----t---tt----ttttac----ccctt------
lemur                        --------tgtaattt-tactttttt---t---tt----ttttac----ccctt------
polar_bear                   --------tgtaattt-tactttt---------------ttttac----ccctt------
panda                        --------tgtaattt-tactttt---------------ttttac----ccctt------
horse                        --------tgtaattt-tactttt---------------ttttac----ccctt------
sloth                        --------tgtaattt-tactttttttt-atcc-c-----------------tt------
rabbit                       --------tgtaattt-tacttt-----------t----ttttta----ccctt------
hyrax                        --------tgtaattt-taccttttttt-accc-c-----------------tt------
treeshrew                    --------tgtgattt-tacttt-----------t----ttttac----cccct------
hedgehog                     --------tgtaattt-tactttt----------t----ttttac----ccctt------
microbat                     --------tgtaattt-tactttt---------------ttttac----ccctt------
megabat                      --------tgtaattt-tactttt---------------ttttac----ccctt------
mouse                        --------tgtaattt-tacttt----------tt----ttttgc----ccctt------
kangaroo_rat                 --------tgtaattt-tactt------------t----ttttac----ccctt------
blind_mole_rat               --------tgtaattt-ttactt----------tt----ttttgc----ccctt------
Damalarand_mole-rat          --------tgtaattt-tgctttttttt-tttttt----ttttta----ccctt------
naked_mole_rat               --------tgtaattt-tgcttttttt------tt----ttttta----ccctt------
guinea_pig                   --------tgtaattt-tgcttttttt------tt----ttttta----ccctt------
armadillo                    --------tgtgattt-tactttttttt-accc-c-----------------tt------
Tasm.devil                   --------tgtaattt-tacttttttta-cccctt-----------------cc------
opossum                      --------tgtaattt-tacttttttta-ccccct------------------t------
wallaby                      --------tgtaattt-tacttttttta-cccccc-----------------tt------
platypus                     --------tgtaattttcctttttttta-cccctc----cccccg----cctttctctc
duck                         --------tgtaattt-aactttt-tta-cccc-------------------tc------
zebrafinch                   --------tgtaattt-aactttt-tta-cccc-------------------tc------
chicken                      --------tgtaattt-aactttt-tta-cccc-------------------tc------
turkey                       --------tgtaattt-aactttt-tta-cccc-------------------tc------
quail                        --------tgtaattt-aactttt-tta-cccc-------------------tc------
alligator                    --------tgtaactt-aacttttt-ta-cccctc----cc-ctc----cctttccca--
desert_tortoise              --------tgtaattt-aacttttttta-tccc-------------------tt------
turtle_softshell             --------tgtaattt-aacttttttta-tccc-------------------ta------
turtle_painted               --------tgtaattt-aacttttttta-tccc-------------------tt------
tuatara_lizard               --------tgtaattt-taacttttata-cccc-------------------tt------
anole_lizard                 --------tgtaatttttaacttttata-cccctt----tccttt----ctgttcccg--
python_Burmese               --------tataatgtttaacttttata-cccctt----tccttc----ccccccctt--
xenopus_trop.                ctc-ttcttgttatctttacctttctgt-gtcctc---------------ttcacccaac
xenopus_laevis               ctc-ctcctgttgtctttg--------t-gccc-c---------------tttacct---
ocean_sunfish                ct--gtggtgttaatgttgctctctctc--tctctctctctctctctctctctctctc--
tilapia                      cc--atgatgttaactttgcttttc-cc--tcccacccgccctcctccacccccacc---
zebra_mbuna                  ct--gtgatgttaactttgcttttc-cc--tcccacccgccctcccccctctctcct---
stickleback                  tt--gtggtgctaatacttctctctctc--tctccccaccctcctcctctcctc-----
climbing_perch               ct--gtggtgttaatgttgcttttc-cc--acccaccctctcttcttctctccttc----
ballan_wrasse                ct--gtggtgttaatgttgcttttc-cc--tcccaccctctctctctctctctctctc
swamp_eel                    ct--gtggtgttaatgctgcttttc-cc--tcccaccctccctcttcttctcct---ctc
zigzag_eel                   ct--gtaatgttaatgtcgcttttc-cc--ccccgccctctctttt-ctcttctt-----
tetraodon                    gt--gtgtcgctaacaccatttctcccc----cctgt---gcccc-----------tctc
tigertail_seahorse           tc--gttgtgttaatgtcgcttctc-cg--tcccacccgctctcctcctcctcctc----
fugu                         gt--gtgt---taacatcatttctccac----cccccctccccccctcc----cccctctc
tongue_sole                  ct--gtggtgttaatgttgctcttcctccctcccacccctctatctttc--ctctcctctc
amazon_molly                 ct--ctggtgttaacgttgctcttc--c--tcccacccatcctcctcct------c----
shortfin_molly               ct--ctggtgttaacgttgctcttc--c--tcccacccatcctcctcct------c----
bicolor_damselfish           ct--gtggtgttaatgctgcttctc-cc--tcccaccc-tcctcctcctcttcctc----
zebrafish                    ctgttttgtgtgattgttgctttccccc-tt------------------c--cctttatc
spotted_gar                  ctg-tttgtgttaattgtgctttttccc-ccctt----ccttcc----tccttcctt--
channel_catfish              ctgttttgtgttattgtcgctcccccccc-accccacccaacctccccttcccccctctatc
cod                          ac--cccgtgttaatactgcttctc-cc--tcccaccctctcttgtcctctctttctccc
```

```
human                          ---c--cctcttttt---------------t----------ttt---cttctgt------
bonobo                         ---c--cctcttt-----------------t----------ttt---cttctgt------
baboon                         ---c--cctctttt----------------t----------ttt---cttctgt------
angola_colobus                 ---c--cctctttt----------------t----------ttt---cttctgt------
bolivian_squirrel_monkey       ---c--cctctttt----------------t----------ttt---ctcctgt------
bushbaby                       ---c--cctctttt----------------t----------ttt---ctcctgt------
bamboo_lemur                   ---c--cctctttt----------------t----------ttt---ctcctgt------
lemur                          ---c--cctctttt----------------t----------ttt---ctcctgt------
polar_bear                     ---c--cctcttttt---------------t-----------tt---ctcctgt------
panda                          ---c--cctcttttt---------------t-----------tt---ctcctgt------
horse                          ---c--cctcttttt---------------t-----------tt---ctcctgt------
sloth                          ---c--cctcttttt---------------ttt--tctcc-t----------gt------
rabbit                         ---c--cctcttt-----------------t----------ttt---ctcctgt------
hyrax                          ---c--cctcttttt---------------ttt--tctcc-t----------gt------
treeshrew                      ---c--cctctttt----------------t----------ttt---ctcctgt------
hedgehog                       ---c--cctcttttt---------------t-----------tt---ctcctgt------
microbat                       ---c--cctcttttt---------------t----------ttc---ctcctgt------
megabat                        ---c--cctcttttt---------------t-----------tt---ctcctgt------
mouse                          ---c--cctctttt----------------t----------ttt---ctcctgt------
kangaroo_rat                   ---c--cctcttttt---------------t----------ttt---ctcctgt------
blind_mole_rat                 ---c--cctctttt----------------t----------ttt---ctcctgt------
Damalarand_mole-rat            ---c--cctctttt----------------t----------ttt---ctcctgt------
naked_mole_rat                 ---c--cctctttt----------------t----------ttt---ctcctgt------
guinea_pig                     ---c--cctctttt----------------t----------ttt---ctcctgt------
armadillo                      ---c--cctcttttt---------------t-----ttcc-t----------gt------
Tasm.devil                     ---c--tcttttttt---------------ttt--tctct-t----------gt------
opossum                        ---c--cctcttttt---------------ttt--tctct-t----------gt------
wallaby                        ---c--cctcttttt---------------ttt--tctct-t----------gt------
platypus                       tgtc--tgtcttttt---------------ttttttct----tc---attttgt------
duck                           ---c--cctcttttg---------------ttt-ttctcccttt---ctcctgt------
zebrafinch                     ---c--cctcttttg---------------ttt-ttctc--------------t------
chicken                        ---c--cctcttttg---------------ttt-ttctccctct---cccctct------
turkey                         ---c--cctcttttg---------------ttt-ttctccctct---cccctct------
quail                          ---c--cctcttttt---------------gtt-t---------------ctct------
alligator                      --cc--cctcttttt---------------ttt-g------ttt---ctcctgt------
desert_tortoise                ---c--cctttcctt---------------tt---------ccc---cccccct------
turtle_softshell               ---c--cctcttttt---------------ttt-ttt--ctttt---cctttct------
turtle_painted                 ---c--cctttcctt---------------ct---------ccc---cccc---------
tuatara_lizard                 ---t--ccttttttc---------------tt---------tct---cccctat------
anole_lizard                   ------------------------------------------ct---cccccat------
python_Burmese                 --cc-------------------------------------ccc---cccattt------
xenopus_trop.                  tgtc--cctttctgt--------------gctttgccacctttccatcctttct------
xenopus_laevis                 ------------------------------cttcttcctctttccttcctttgt------
ocean_sunfish                  ------tctctctctctctctctctctctct---------------------ctcctc
tilapia                        --------------------------------------cc---cccttct----------
zebra_mbuna                    --------------------------------------cc---tcctcct----------
stickleback                    ----------------------------------------------------ctctggtt
climbing_perch                 -------ctctttttgtc------aa-atctttct----c---ttttcctttctttggtc
ballan_wrasse                  tctctcgctctctctctctctctctccccctctctcctcc---tcttcctctctttggtc
swamp_eel                      cttc---ctcctcttgtc-----caa------------tg---ccatcctccctttggtt
zigzag_eel                     -------------ctgtc---------------------c---ttcctctctctttggtc
tetraodon                      c------------t---------------ctcctccctcc---tcactct----------
tigertail_seahorse             -------ctcctctt--------------ct---t---cc---tcctcct---cttggtt
fugu                           t------------ctcc------------ctccctccacc---tcactct----------
tongue_sole                    ctctgctctcctct-------------------------g---ctctcctctctgtgt--
amazon_molly                   -------c----------------------------------------------------
shortfin_molly                 -------c----------------------------------------------------
bicolor_damselfish             -------ctcttcctc-----------ctcttcctcctct---tcctcctct---tcc--
zebrafish                      tgtc--cctctttct--------------ctcacttcctcttac----------------
spotted_gar                    ---------c--------------------ctttatctc--------cttctgt------
channel_catfish                tctc--tctctctct--------------ctc--tccatctctc---cctctctctct--
cod                            ----------ctgttgtgttcctccatctctccctcctgt---t---------cctgggt
```

```
human                          --------------------cc---------------------tt---------------
bonobo                         --------------------cc---------------------tt---------------
baboon                         --------------------cc---------------------tt---------------
angola_colobus                 --------------------cc---------------------tt---------------
bolivian_squirrel_monkey       --------------------cc---------------------tt---------------
bushbaby                       --------------------cc---------------------tt---------------
bamboo_lemur                   --------------------cc---------------------tt---------------
lemur                          --------------------cc---------------------tt---------------
polar_bear                     --------------------cc---------------------tt---------------
panda                          --------------------cc---------------------tt---------------
horse                          --------------------cc---------------------tt---------------
sloth                          --------------------cc---------------------t----------------
rabbit                         --------------------cc---------------------tt---------------
hyrax                          --------------------cc---------------------tt---------------
treeshrew                      --------------------cc---------------------tc---------------
hedgehog                       --------------------cc---------------------tt---------------
microbat                       --------------------cc---------------------tt---------------
megabat                        --------------------cc---------------------tc---------------
mouse                          --------------------cc---------------------tt---------------
kangaroo_rat                   --------------------cc---------------------tt---------------
blind_mole_rat                 --------------------cc---------------------tt---------------
Damalarand_mole-rat            --------------------cc---------------------tt---------------
naked_mole_rat                 --------------------cc---------------------tt---------------
guinea_pig                     --------------------cc---------------------tt---------------
armadillo                      --------------------cc---------------------tc---------------
Tasm.devil                     --------------------cc---------------------tt---------------
opossum                        --------------------cc---------------------tt---------------
wallaby                        --------------------cc---------------------tt---------------
platypus                       --------------------ca------------------c--tt---------------
duck                           --------------------cc---------------------ct---------------
zebrafinch                     --------------------cc---------------------ct---------------
chicken                        --------------------cc---------------------ct---------------
turkey                         --------------------cc---------------------ct---------------
quail                          --------------------cc---------------------ct---------------
alligator                      --------------------cc------------------c--tt---------------
desert_tortoise                --------------------cc---------------------ct---------------
turtle_softshell               --------------------cc---------------------ct---------------
turtle_painted                 --------------------cc---------------------cc---------------
tuatara_lizard                 --------------------cc------------------c--tt---------------
anole_lizard                   --------------------cc------------------c--tc---------------
python_Burmese                 --------------------cc------------------c--tc---------------
xenopus_trop.                  --------------------gc------------------c--tc---------------
xenopus_laevis                 --------------------gc------------------t--ac---------------
ocean_sunfish                  ttcccctctacgctcaccctgctgttgtccttctgtctcttcctttttttcc-----tctc
tilapia                        --ctctaacgctt----cctgtt-------------------------------------
zebra_mbuna                    --ctctaacactt----cctgct-------------------------------------
stickleback                    tcctc-tct-----------------------------------------------cctc
climbing_perch                 t---tttttaca-cc--cccgctgttgtccaacactttacctcttta-------------
ballan_wrasse                  gtcttttattcttttaccctgctgttgttggatttctgccttctttcctct---gttctc
swamp_eel                      ttctttaaaac--tcaccctgctgttatccatcactctcctcctttccttccaatttttt
zigzag_eel                     t---tttatact-ccgccctgctcctgtccaacaccgtcctccttt--------------
tetraodon                      ------------------------------------------------------------
tigertail_seahorse             gtgtttgct---------------------------------------------------
fugu                           ------------------------------------------------------------
tongue_sole                    ------aa-----tcatcttcctctggtccccgcccctc---attt-----------tt-
amazon_molly                   ------------------------------------------------------------
shortfin_molly                 ------------------------------------------------------------
bicolor_damselfish             ----------------tcctcct------------cctcct---------------cctc
zebrafish                      ---------------------c------------------c-cgt---------------
spotted_gar                    --------------------tg------------------c--ct---------------
channel_catfish                ------------------ctct------------------c-ctt---------------
cod                            ttcttatctttt--t---tccgtgtggtccacttgcgt----------------------
```

```
human                         --ttgtgt----------------------------------------------------
bonobo                        --ttgtgt----------------------------------------------------
baboon                        --ttgtgt----------------------------------------------------
angola_colobus                --ttgtgt----------------------------------------------------
bolivian_squirrel_monkey      --ttgtgt----------------------------------------------------
bushbaby                      --ttgtgt----------------------------------------------------
bamboo_lemur                  --ttgtgt----------------------------------------------------
lemur                         --ttgtgt----------------------------------------------------
polar_bear                    --ttgtgt----------------------------------------------------
panda                         --ttgtgt----------------------------------------------------
horse                         --ttgtgt----------------------------------------------------
sloth                         --ctgtgt----------------------------------------------------
rabbit                        --ttgtgt----------------------------------------------------
hyrax                         --ttgtgt----------------------------------------------------
treeshrew                     --tcgtgt----------------------------------------------------
hedgehog                      --ttgtgt----------------------------------------------------
microbat                      --ttgtgt----------------------------------------------------
megabat                       --ttgtgt----------------------------------------------------
mouse                         --ttgtgt----------------------------------------------------
kangaroo_rat                  --ttgtgt----------------------------------------------------
blind_mole_rat                --ttgtgt----------------------------------------------------
Damalarand_mole-rat           --ttgtgt----------------------------------------------------
naked_mole_rat                --ttgtgt----------------------------------------------------
guinea_pig                    --ttgtgt----------------------------------------------------
armadillo                     --ctgtgt----------------------------------------------------
Tasm.devil                    --ttgtat----------------------------------------------------
opossum                       --ttgtat----------------------------------------------------
wallaby                       --ttgtatt---------------------------------------------------
platypus                      --ccttgcc----cccc-----------------c----c-----------cccccatcc
duck                          --c-ttggt---------------------------------------------------
zebrafinch                    --t-ttggt---------------------------------------------------
chicken                       --c-ctggt---------------------------------------------------
turkey                        --c-ctgga---------------------------------------------------
quail                         --c-ctggt---------------------------------------------------
alligator                     --t--tggt---------------------------------------------------
desert_tortoise               --ttttggt---------------------------------------------------
turtle_softshell              --gtgtggt---------------------------------------------------
turtle_painted                --ttttggt---------------------------------------------------
tuatara_lizard                --ctttgct---------------------------------------------------
anole_lizard                  --tttcggt---------------------------------------------------
python_Burmese                --ttttggt---------------------------------------------------
xenopus_trop.                 --ttctcct----cccct--------------gcc----c-----------cccccaacc
xenopus_laevis                --ttctccc----a-cct--------------gc-----a-----------cctccaacc
ocean_sunfish                 gtgtgtgtgt----------gttgatgttgagcttt-ctttctgt------cgttcctcc
tilapia                       -----tct-tctaactt---ttc---------------------------------aacg
zebra_mbuna                   -----ttt-cctaactt---ttc---------------------------------accg
stickleback                   atccgtgtgtctaatcttttttg--tggtga-----------------------------
climbing_perch                --acttcttt----------ccg---------------tttcaact--------------
ballan_wrasse                 gttttctgtcaaaccctgtttctatatgattcccttctctctattcttctccttcacgt
swamp_eel                     gtttttttgccaaatcctgtttcgatgctgatacttttgccc---------tcttcattt
zigzag_eel                    --ccttctgtcaaatcctgttcg-atgttgg-------tctttaat--------------
tetraodon                     --atgtgtgc----------ttccattt--------------------------------
tigertail_seahorse            --tggtgtgc----------tgccgtgt----------tctc--------tcctcca---
fugu                          ---tgtgtgc----------ttctgtgt--------------------------------
tongue_sole                   --gctcctgcctcgctcatgtccgacgctcct----------------------gcctcc
amazon_molly                  ------------------------------------------------------------
shortfin_molly                ------------------------------------------------------------
bicolor_damselfish            gtt--tctcc----------tctgatgt--------------------------------
zebrafish                     --ttttcaa----cccct--------------c------c-----------tctacaccc
spotted_gar                   --ttttttt----cccc-----------------a----c-----------actacatat
channel_catfish               --ttatctg-----tcct--------------cctt--tc-----------ttttcttac
cod                           --------------------gtttatattcacccgcttcccc----cccctccctcctgc
```

```
human                        -------------gt--------------gt-------------------------cctt
bonobo                       -------------gt--------------gt-------------------------cctt
baboon                       -------------gt--------------gt-------------------------cctt
angola_colobus               -------------gt--------------gt-------------------------cctt
bolivian_squirrel_monkey     -------------gt--------------gt-------------------------cctt
bushbaby                     -------------gt--------------gt-------------------------cctt
bamboo_lemur                 -------------gt--------------gt-------------------------cctt
lemur                        -------------gt--------------gt-------------------------ccat
polar_bear                   -------------gt--------------gt-------------------------cc--
panda                        -------------gt--------------gt-------------------------cc--
horse                        -------------gt--------------gt-------------------------cctt
sloth                        -------------gc--------------gt-------------------------cct-
rabbit                       -------------gt--------------gt-------------------------cctt
hyrax                        -------------gt--------------gt-------------------------cct-
treeshrew                    -------------gt--------------gt-------------------------cctc
hedgehog                     -------------gt--------------gt-------------------------cctt
microbat                     -------------gt--------------gt-------------------------ccct
megabat                      -------------gt--------------gt-------------------------cctt
mouse                        -------------gt--------------gt-------------------------cctt
kangaroo_rat                 -------------gt--------------gt-------------------------cctt
blind_mole_rat               -------------gt--------------gt-------------------------cctt
Damalarand_mole-rat          -------------gt--------------gt-------------------------cctt
naked_mole_rat               -------------gt--------------gt-------------------------cctt
guinea_pig                   -------------gt--------------gt-------------------------cctt
armadillo                    -------------gc--------------at-------------------------cct-
Tasm.devil                   -------------gt--------------gt-------------------------cct-
opossum                      -------------at--------------gt-------------------------cct-
wallaby                      -------------gt--------------gt-------------------------cct-
platypus                     t-c---------ggt--------------gt-------------------------acgt
duck                         -------------at--------------ct-------------------------gttc
zebrafinch                   -------------tc--------------ct-------------------------gttc
chicken                      -------------ac--------------ct-------------------------gttc
turkey                       -------------ac--------------ct-------------------------gttc
quail                        -------------ac--------------ct-------------------------gtcc
alligator                    -------------ct--------------cc-------------------------actc
desert_tortoise              -------------at--------------cc-------------------------act-
turtle_softshell             -------------gt--------------cc-------------------------act-
turtle_painted               -------------at--------------cc-------------------------act-
tuatara_lizard               -------------at--------------ct-------------------------act-
anole_lizard                 -------------gt--------------cc-------------------------actt
python_Burmese               -------------tt--------------cc-------------------------acta
xenopus_trop.                tgc---------cttcccctgtatctga-ctgca----------------------actc
xenopus_laevis               tgc---------cg------ccatctga-ctgct----------------------acgc
ocean_sunfish                t-tttcttttatcctttactttcc-taatcctccaccttatccatgcttgtcct------
tilapia                      tttctccc----------gtttct-gatttcttccctccatc-ctgcttctcatctgctt
zebra_mbuna                  tttctccc----------gtttct-gatttcttccgtccatc-ctgcttctcatctgctc
stickleback                  tattc---acctgttttacttca-caaacct-----tccatc-ctgcttctctgct----
climbing_perch               ---ttttctgttcctttactttct-aaatctttcactccgtt-gtgcttctcgtccacca
ballan_wrasse                tatctctc-tttcttttacttcctccaaattttcactccatc-ttgcttctcatccactc
swamp_eel                    tctctatctctccttatacttttt-aaaccctttcctccatc-ttgcttctcatccactc
zigzag_eel                   ---ctctccttccttatggtttct-gaatctcccactccatc-ttgcttctcatccactc
tetraodon                    ----------ctggttt---------aat-------tccactcttcctc-----------
tigertail_seahorse           -------------------------caacctgtcg-------tttgcttttccc--act-
fugu                         ---------cctgcttt---------att-------cccactcttggtc-----------
tongue_sole                  tttgtt--tttttttttttttttttaaat-------cccccccttgcttatcttgcgtcc
amazon_molly                 -------------tctaaccctct--aaca-----ctcccc-----------ctctgttg
shortfin_molly               -------------tctaaccctct--aaca-----ctcccc-----------ctctgttg
bicolor_damselfish           ------------ttttaactctct--aaac-----ctccatc-ctgcttctcatctgctg
zebrafish                    g--------------acacttcatctacgatcaa----------------------actt
spotted_gar                  t-t---------ttt--------------ctcc-----------------------aatt
channel_catfish              c--------------acctttcatca---ctcgc----------------------actt
cod                          -gtttgccttttctttaaccttct-aaacctctcacacctcccc-------caaccacta
```

```
human                        -c----------------------------------------------------------
bonobo                       -c----------------------------------------------------------
baboon                       -c----------------------------------------------------------
angola_colobus               -c----------------------------------------------------------
bolivian_squirrel_monkey     -c----------------------------------------------------------
bushbaby                     -c----------------------------------------------------------
bamboo_lemur                 -c----------------------------------------------------------
lemur                        -c----------------------------------------------------------
polar_bear                   ------------------------------------------------------------
panda                        ------------------------------------------------------------
horse                        -c----------------------------------------------------------
sloth                        -t----------------------------------------------------------
rabbit                       -c----------------------------------------------------------
hyrax                        -t----------------------------------------------------------
treeshrew                    -c----------------------------------------------------------
hedgehog                     -c----------------------------------------------------------
microbat                     -c----------------------------------------------------------
megabat                      -c----------------------------------------------------------
mouse                        -c----------------------------------------------------------
kangaroo_rat                 -c----------------------------------------------------------
blind_mole_rat               -c----------------------------------------------------------
Damalarand_mole-rat          -c----------------------------------------------------------
naked_mole_rat               -c----------------------------------------------------------
guinea_pig                   -c----------------------------------------------------------
armadillo                    -t----------------------------------------------------------
Tasm.devil                   -t----------------------------------------------------------
opossum                      -t----------------------------------------------------------
wallaby                      -t----------------------------------------------------------
platypus                     gtc-gt-----ccg-----tccgtccc---------c---------ccctc---------
duck                         -t----------------------------------------------------------
zebrafinch                   -t----------------------------------------------------------
chicken                      -t----------------------------------------------------------
turkey                       -t----------------------------------------------------------
quail                        -t----------------------------------------------------------
alligator                    -tc--------ctc-----tcctctcc---------t---------ccttt---------
desert_tortoise              -t----------------------------------------------------------
turtle_softshell             -t----------------------------------------------------------
turtle_painted               -t----------------------------------------------------------
tuatara_lizard               -t----------------------------------------------------------
anole_lizard                 -t----------------------------------------------------------
python_Burmese               -t----------------------------------------------------------
xenopus_trop.                cca-ctccatgctt-----atctgcac---------ctgctcccaatcatc-ttcctact
xenopus_laevis               ctc-ctccatgcat-----ttctc----------------------ttctc-ttcctact
ocean_sunfish                ---------------tcc----------gaccgtttgctctctacatcttgtttcctc--
tilapia                      cgctcttcatcc-------------tctgtctgttatttggttgcatctc-----ctc--
zebra_mbuna                  cgctcttcatcc-------------tctgtctgttatttggttgcatctc-----ctc--
stickleback                  ----------------------------------tgtttggttccttct------ctc--
climbing_perch               tcctc-----ccacatc-----tgtatttct--------------------gttattc--
ballan_wrasse                gcctctcaacgtcctctcgcctccccttatcggctgttttgttgcgtcttgtttcctc--
swamp_eel                    gg-tctccacgtcttcccacc----------ttttatttctttgcacattgtttcctc--
zigzag_eel                   tgctctccatccacatc-----ctctctttccattgttacgttgcatcttggttcctc--
tetraodon                    --------------------------c----tgcctgtttgctgcatcttgc--------
tigertail_seahorse           ---------------------------------------------------cctcgtg--
fugu                         --------------------------t----tggctgtttgctgcatcttctttcctg--
tongue_sole                  gcctctccatactttccg----------------------------------gtcctc--
amazon_molly                 -----------------t----gactctt-------------------------cat---
shortfin_molly               -----------------t----gactctt-------------------------cat---
bicolor_damselfish           -----------------cacctcactctttccgttatttcgttgcacctcctctcatc--
zebrafish                    cca--------------caaca------------------tctcaaccttc-tttctgcc
spotted_gar                  acc-ct----cccc-----ttctcccc---------c---------ccctc---------
channel_catfish              ccatcttcaccctctcccatcgtgggt---------gctctcgcaat---c-tttgcccc
cod                          ---tctccc-----------ctccctccctctcttctttctcccc-------tccctc--
```

```
human                         ------------------------------------------------------------
bonobo                        ------------------------------------------------------------
baboon                        ------------------------------------------------------------
angola_colobus                ------------------------------------------------------------
bolivian_squirrel_monkey      ------------------------------------------------------------
bushbaby                      ------------------------------------------------------------
bamboo_lemur                  ------------------------------------------------------------
lemur                         ------------------------------------------------------------
polar_bear                    ------------------------------------------------------------
panda                         ------------------------------------------------------------
horse                         ------------------------------------------------------------
sloth                         ------------------------------------------------------------
rabbit                        ------------------------------------------------------------
hyrax                         ------------------------------------------------------------
treeshrew                     ------------------------------------------------------------
hedgehog                      ------------------------------------------------------------
microbat                      ------------------------------------------------------------
megabat                       ------------------------------------------------------------
mouse                         ------------------------------------------------------------
kangaroo_rat                  ------------------------------------------------------------
blind_mole_rat                ------------------------------------------------------------
Damalarand_mole-rat           ------------------------------------------------------------
naked_mole_rat                ------------------------------------------------------------
guinea_pig                    ------------------------------------------------------------
armadillo                     ------------------------------------------------------------
Tasm.devil                    ------------------------------------------------------------
opossum                       ------------------------------------------------------------
wallaby                       ------------------------------------------------------------
platypus                      ------------------------------------------------------------
duck                          ------------------------------------------------------------
zebrafinch                    ------------------------------------------------------------
chicken                       ------------------------------------------------------------
turkey                        ------------------------------------------------------------
quail                         ------------------------------------------------------------
alligator                     ------------------------------------------------------------
desert_tortoise               ------------------------------------------------------------
turtle_softshell              ------------------------------------------------------------
turtle_painted                ------------------------------------------------------------
tuatara_lizard                ------------------------------------------------------------
anole_lizard                  ------------------------------------------------------------
python_Burmese                ------------------------------------------------------------
xenopus_trop.                 gt--tcctact-----------------------g-------------------------
xenopus_laevis                gt----------------------------------------------------------
ocean_sunfish                 -ctcttcc-tccctt------ca-tttgctccattccctcctcttcc-------------
tilapia                       -c---tcc-----ttct-------------tcatc----------------tcaccctgt
zebra_mbuna                   -ctcctcc-----tcct-------------tcatc----------------tcaccctgt
stickleback                   -ttccttcacctctttc----------atctcactccatcca-------cttcaccttct
climbing_perch                ----ctc-actcc-------------------------------tctct---------tt
ballan_wrasse                 -gtcctc-attccttctccttcg-tttcgttcgttcgctcctcctcctcgtccaacttct
swamp_eel                     -ctcgtccatgccttct-----------tttcactcccacctcctcttcgtttc------
zigzag_eel                    ----ccc-accccttcctccttg-------tcatttcctcctcctcttc-ttcaccgtct
tetraodon                     ------------------------------------------------------------
tigertail_seahorse            -ctcgtccatgtgttt--cctcacttggcttcac--------------------------
fugu                          -cttttt----tcttc------------tctcatc-------------------------
tongue_sole                   -gtcctg----ccttat-------tttgttgcact-----------------------g-
amazon_molly                  ----------------------------------------------------c-------
shortfin_molly                ----------------------------------------------------c-------
bicolor_damselfish            -----------t---------------------------------------cc-------
zebrafish                     cc--ttctacatctttt---------tcctgtggg-------------------------
spotted_gar                   ------------------------------------------------------------
channel_catfish               cattttttaaatttttt---------ttt-ttatt-------------------------
cod                           -cttctctct-tctttt---------------------tcctcctctctggttatcggac
```

```
human                        ----------cctc--tcatcg-----------t------tg--------------g-c-
bonobo                       ----------cctc--tcatcg-----------t------tg--------------g-c-
baboon                       ----------cctc--tcatcg-----------t------tg---------------cc-
angola_colobus               ----------cctc--tcatcg-----------t------tg---------------cc-
bolivian_squirrel_monkey     ----------cctc--tcatcg-----------t------tg--------------gcc-
bushbaby                     ----------cctc--tcatcg-----------t------tg--------------gtc-
bamboo_lemur                 ----------cctc--tcatcg-----------t------tg--------------gcc-
lemur                        ----------cctc--tcatcg-----------t------tg--------------gcc-
polar_bear                   -----------ccct-ccctcg-----------c------tg--------------gcc-
panda                        -----------ccct-ccctcg-----------c------tg--------------gcc-
horse                        ----------cctct-ccatcg-----------c------tg--------------gcc-
sloth                        ----------ccctgtccatcg-----------t------tg--------------gcc-
rabbit                       ----------cctc--tcatcg-----------c------tg--------------gcc-
hyrax                        ----------ccctctccattg-----------t------tg--------------gcc-
treeshrew                    ----------cttc--acctcg------------------tg--------------gcc-
hedgehog                     ----------cctcccccaccg-----------t------tg--------------gcc-
microbat                     ----------cctcc-ccatca-----------c------tg--------------gcc-
megabat                      ----------cctct-ccatcg-----------c------tg--------------gcc-
mouse                        ----------cctc--tcatcg-----------t------tg--------------gcc-
kangaroo_rat                 ----------cctc--tcattg-----------t------tg--------------gtc-
blind_mole_rat               ----------cctc--tcattg-----------t------tg--------------gcc-
Damalarand_mole-rat          ----------cctc--tcatcg-----------t------tg--------------gcc-
naked_mole_rat               ----------cctc--tcatcg-----------t------tg--------------gcc-
guinea_pig                   ----------cctc--tcatcg-----------t------tg--------------gcc-
armadillo                    ----------ccccgtccactg-----------t------tg--------------gccc
Tasm.devil                   ----------tcctttccattg-----------t------tg--------------gcc-
opossum                      ----------tcctttccattg-----------t------tg--------------gcc-
wallaby                      ----------tcctttccattc-----------t------tg--------------gcc-
platypus                     ---------cccgcctccactc-----------c------cg--------------gc--
duck                         ----------cc-tgtccactc-----------c------tg--------------gcc-
zebrafinch                   ----------cc-tttccattc-----------c------tg--------------atg-
chicken                      ----------cc-cctccattc-----------c------cg--------------gcc-
turkey                       ----------ccccctccattc-----------c------cg--------------gcc-
quail                        ----------cc-cctccattc-----------c------cg--------------gcc-
alligator                    ---------cctccctccattc-----------c------cg--------------gct-
desert_tortoise              ----------tcctttccattc-----------c------tg--------------gcc-
turtle_softshell             ----------tcctttccactc-----------c------tg--------------gcc-
turtle_painted               ----------tcctttccattc-----------c------tg--------------gcc-
tuatara_lizard               ----------tcctttctactc-----------c------tg--------------act-
anole_lizard                 -----------ccttttcatcc-----------c------tg--------------gcc-
python_Burmese               -----------ccttttcattt-----------c------tg--------------gcc-
xenopus_trop.                ---------tctatttcaaccc-----------ctc-ccatgtcctg-tccttggaacct
xenopus_laevis               ---------tctacttcaaccc-----------ctc-ccatgtcctg-tccttggaacct
ocean_sunfish                ----------------------tcctcctcctcct--cctcctcctatccctgggtgggc
tilapia                      c----------ct--------------caccctcc--tctccccctg-ccctgggtgggc
zebra_mbuna                  t----------ct--------------cacccccc--cct--tcctg-ccctgggtgggc
stickleback                  ccttcat----ctcatcc---------cttcctct--cctcactccc-tctcgggtgggt
climbing_perch               cctt----------------------------cct--cttcctcccg-tcctgggtgggc
ballan_wrasse                cctcacttcttcacctccatcctcctcctcctctt--cttcctcctg-tcccgggtgggc
swamp_eel                    ---------gattcccccacactcctccatccctt--tctcctcctg-tcccgggtgggc
zigzag_eel                   ccttcgtt-ttctcgtca---------ccccccct--ccctctcctg-ccctgggtgggc
tetraodon                    ---------------------ctcctcctcctcct--cctcctctgcctcctggctgggt
tigertail_seahorse           ----------tgtcctccatcctcctgttcctcct--ccttctcctc-tcccgggtgggc
fugu                         ---------------tgcttcctcctcctcctcct--cctcctcctcttcctgggtcggt
tongue_sole                  ---------------tgc------ttcctcttcct--cctcctcctt-ttctgggtgggc
amazon_molly                 -----------ac---------tcttcctcctcct--cct-gtcctg-tcctgggtgggt
shortfin_molly               -----------ac---------tcttcctcctcct--cct-gtcctg-tcctgggtgggt
bicolor_damselfish           -----------accctccaccctccatctcctcct--cctcctcctg-tcctgggtgggt
zebrafish                    ---------tggttttgcgtttg---------gctc-c--ctttttg-tggcgggacctc
spotted_gar                  ---------tttatttttattt-----------t------tg--ttt-ctctccgaaccc
channel_catfish              ---------ttgtttt--atttt---------gttc-c--ccctccc-ccgtcggctgca
cod                          tcttcctcttgttctttcac--tctgcctccccatccacacttcctgtccccgggtgc-g
```

```
human                         ---------------cac--tcatagATACGAGGGCACCATGTAGCACAGCTGGACCCCC
bonobo                        ---------------cac--tcatagATACGAGGGCACCATGTAGCACAGCTGGACCCCC
baboon                        ---------------cac--tcatagATACGAGGGCACCATGTAGCACAGCTGGACCCCC
angola_colobus                ---------------cac--tcatagATACGAGGGCACCATGTAGCACAGCTGGACCCCC
bolivian_squirrel_monkey      ---------------cac--tcatagATACGAGGGCACCATGTAGCACAGCTGGACCCCC
bushbaby                      ---------------cac--tcatagATACGAGGGCACCATGTAGCACAGCTGGACCCCC
bamboo_lemur                  ---------------cac--tcatagATACGAGGGCACCATGTAGCACAGCTGGACCCCC
lemur                         ---------------cac--tcatagATACGAGGGCATCATGTAGCACAGCTGGACCCCC
polar_bear                    ---------------cgt--ccgtagATACGAGGGCACCATGTAGCGCAGCTGGACCCCC
panda                         ---------------cgt--ccgtagATACGAGGGCACCATGTAGCGCAGCTGGACCCCC
horse                         ---------------cat--tcgtagATACGAGGGCACCATGTAGCACAGCTGGACCCCC
sloth                         ---------------cat-ttcatagATACGAGGGCACCATGTAGCACAGCTGGACCCCC
rabbit                        ---------------c----tcgtagATCCGAGGGCACCATGTAGCACAGCTGGACCCCC
hyrax                         ---------------cat--ccatagATACGAGGGCACCATGTAGCACAGCTGGACCCCC
treeshrew                     ---------------cac--tcgtagATACGCGGGCACCATGTAGCGCAGCTGGACCCCC
hedgehog                      ---------------cat--tcgtagATACGAGGGCACCATGTAGCACAGCTGGACCCCC
microbat                      ---------------ctt--tcatagATACGAGGGCACCATGTAGCACAGCTGGACCCCC
megabat                       ---------------ctt--tcgtagATACGAGGGCACCATGTAGCACAGCTGGACCCCC
mouse                         ---------------cac--tcatagATACGAGGGCACCATGTAGCACAGCTGGACCCCC
kangaroo_rat                  ---------------cac--tcatagATACGAGGGCACCATGTAGCACAGCTGGACCCCC
blind_mole_rat                ---------------cac--tcatagATACGAGGGCACCATGTAGCACAGCTGGACCCCC
Damalarand_mole-rat           ---------------cac--tcatagATACGAGGGCACCATGTAGCACAGCTGGACCCCC
naked_mole_rat                ---------------cac--tcatagATACGAGGGCACCATGTAGCACAGCTGGACCCCC
guinea_pig                    ---------------cac--tcatagATACGAGGGCACCATGTAGCACAGCTGGACCCCC
armadillo                     ---------------cat-cttgtagATACGAGGGCACCATGTAGCACAGCTGGACCCCC
Tasm.devil                    ---------------cat-ttcatagATTCGAGGGCACCATGTAGCACAGCTGGATCCCC
opossum                       ---------------cat-ttcatagATTCGAGGGCACCATGTAGCACAGCTGGATCCCC
wallaby                       ---------------cat-ttcatagATTCGAGGGCACCATGTAGCACAGCTGGATCCCC
platypus                      ---------------cac-cccatagATCCGAGGGCACCATGTGGCCCAGCTGGACCCCC
duck                          ---------------cat-ttcatagATTCGAGGGCATCATGTAGCACAACTCGACCCAC
zebrafinch                    ---------------cat-ttcatagATTCGAGGGCATCATGTAGCACAACTCGATCCAC
chicken                       ---------------cat-ttcatagATTCGAGGGCACCATGTAGCACAGCTCGACCCCC
turkey                        ---------------cat-ttcatagATTCGAGGGCACCATGTAGCACAACTCGACCCCC
quail                         ---------------cat-ctcatagATTCGAGGGCACCATGTAGCACAACTCGACCCCC
alligator                     ---------------cat-ttcatagATTCGAGGGCACCATGTAGCACAGCTCGACCCAC
desert_tortoise               ---------------cat-ttcatagATTCGAGGGCATCATGTAGCACAGCTCGACCCCC
turtle_softshell              ---------------cat-ttcatagATTCGAGGGCATCATGTAGCACAGCTCGACCCAC
turtle_painted                ---------------cat-ttcatagATTCGAGGGCATCATGTAGCACAGCTCGACCCAC
tuatara_lizard                ---------------cat-ttcatagATTCGAGGGCATCATGTAGCACAGCTCGACCCAC
anole_lizard                  ---------------cat-ttcatagATTCGAGGGCATCATGTAGCACAGCTCGACCCAC
python_Burmese                ---------------cat-ttcatagATTCGAGGGCATCATGTAGCCCAGCTCGACCCAT
xenopus_trop.                 ------------tgaatt-catatagATCCGTGGGCACCATGTTGCTCAGCTTGACCCTC
xenopus_laevis                ------------tcaatt-tctatagATCCGTGGGCACCATGTTGCTCAGCTTGACCCAC
ocean_sunfish                 tccatactgttgtcgctc-cctgtagATCCGAGGGCACCACGTGGCCCAGTTGGACCCCC
tilapia                       tctgtgttgttgtcactt-cctgtagATCCGAGGGCACCACGTGGCCCAGTTGGACCCCC
zebra_mbuna                   tctgtgctgttgtcactt-cctgtagATCCGAGGGCACCACGTGGCCCAGTTGGACCCCC
stickleback                   t--------------ccc-cctctagATCCGGGGGCATCACGTGGCTCAGTTGGACCCCC
climbing_perch                tctgtactgttgtcactc-cttgtagATCCGAGGGCACCACGTGGCCCAGTTGGACCCTC
ballan_wrasse                 tctgtgctgttgtcaccc-cctgtagATCCGGGGGCACCATGTGGCCCAGTTGGACCCCC
swamp_eel                     tctgcattgttgtcactc-cctttagATCCGAGGGCACCATGTGGCACAGTTGGACCCTC
zigzag_eel                    tctgtactgttgtcactc-cctgtagATCCGAGGGCACCATGTGGCCCAGTTGGACCCTC
tetraodon                     tc-----tgttctgactt-tctgtagATCCGAGGGCACCAGGTGGCCCAGTTGGACCCTC
tigertail_seahorse            tc-----tgtcctcgttcccctgtagATTCGCGGTCACCAGGTGGCCCAATTGGACCCGC
fugu                          tc-----tgttctcactt-cttgtagATCCGAGGGCACCAGGTGGCCCAGTTGGACCCTC
tongue_sole                   tctctacttttgtcactcccctgtagATCCGAGGTCACCAAGTGGCCCAGTTGGACCCCC
amazon_molly                  tctgtgtc------gccc-tctgtagATCCGGGGGCACCACGTGGCCCAGCTGGACCCGC
shortfin_molly                tctgtgtc------gccc-tctgtagATCCGGGGGCACCACGTGGCCCAGCTGGACCCGC
bicolor_damselfish            tctgttg---------tc-cctgtagATCCGAGGGCACCATGTGGCCCAGTTGGACCCTC
zebrafish                     ------------ttgtct-cctgtagATACGTGGTCACCATGTGGCTCAGCTGGACCCTC
spotted_gar                   ------------cgtgtt-cctgtagATTCGCGGGCACCATGTTGCTCAGCTGGACCCCC
channel_catfish               ------------tcgtct-cctgtagATCCGGGGTCACCATGTCGCTCAGCTGGACCCTC
cod                           tctgtgctgttgtcactt-cctgcagATCCGAGGGCACCATGTGGCCCAGTTGGACCCTC
                                                      **** ** ** ** ** ** ** **  * ** **
```

```
human                         TGGGGATTTTGGATGCTGATCTGGACTCCTCCGTGCCCGCTGACATTATCTCATCCACAGACAAACTTGgtgagg
bonobo                        TGGGGATTTTGGATGCTGATCTGGACTCCTCCGTGCCCGCTGACATTATCTCATCCACAGACAAACTTGgtgagg
baboon                        TGGGGATTTTGGATGCTGATCTGGACTCCTCCGTGCCCGCTGACATTATCTCATCCACAGACAAACTTGGtgagg
angola_colobus                TGGGGATTTTGGATGCTGATCTGGACTCCTCCGTGCCCGCTGACATTATCTCATCCACAGACAAACTTGgtgagg
bolivian_squirrel_monkey      TGGGGATTTTGGATGCTGATCTGGACTCCTCCGTGCCCGCTGACATTATCTCATCCACAGACAAACTTGgtgagg
bushbaby                      TGGGGATTTTGGATGCTGATCTGGACTCCTCCGTGCCCGCTGACATTATCTCATCCACAGACAAACTTGgtgagg
bamboo_lemur                  TGGGGATTTTGGATGCTGATCTGGACTCCTCCGTGCCCGCTGACATTATCTCATCCACAGACAAACTTGgtgagg
lemur                         TGGGGATTTTGGATGCTGATCTGGACTCCTCCGTGCCCGCTGACATTATCTCATCCACAGACAAACTTGgtgagg
polar_bear                    TGGGGATTTTGGACGCCGATCTGGACTCGTCCGTGCCCGCTGACATTATCTCCTCCACAGACAAACTTGgtgagg
panda                         TGGGGATTTTGGACGCCGATCTGGACTCGTCCGTGCCCGCTGACATTATCTCCTCCACAGACAAACTTGgtgagg
horse                         TGGGAATTTTGGATGCTGATCTGGACTCCTCCGTGCCCGCTGATATTATCTCATCCACAGACAAACTTGgtgagg
sloth                         TGGGAATTTTGGATGCTGATCTGGACTCCTCCGTGCCAGCTGATATTATCTCATCCACAGACAAACTTGgtgagg
rabbit                        TGGGGATTTTGGACGCTGATCTGGACTCCTCCGTGCCCGCTGACATTATCTCATCCACAGACAAACTTGgtgagg
hyrax                         TGGGAATTTTGGATGCCGATCTGGACTCCTCCGTGCCAGCTGACATTATCTCATCCACAGACAAACTTGgtgagg
treeshrew                     TGGGGATCTTGGATGCTGATCTGGACTCCTCCGTGCCCGCTGACATTATCTCATCCACAGACAAACTTGgtgagg
hedgehog                      TGGGAATTTTGGATGCTGATCTGGACTCCTCCGTGCCCGCTGACATTATCTCATCCACAGACAAACTTGgtgagg
microbat                      TGGGAATTTTGGATGCCGATCTGGACTCCTCCGTGCCCGCTGACATTGTCTCATCCACAGATAAACTTGgtgagg
megabat                       TGGGAATTTTGGATGCCGATCTGGACTCCTCCGTGCCCGCCGACATTATCTCCTCCACAGACAAACTTGGtgagg
mouse                         TGGGGATTTTGGATGCTGATCTGGACTCCTCCGTGCCCGCTGACATTATCTCATCCACAGACAAACTTGgtgagg
kangaroo_rat                  TGGGGATTTTGGATGCTGATCTGGACTCCTCCGTGCCCGCTGACATTATCTCATCCACAGACAAACTTGgtgagg
blind_mole_rat                TGGGGATTTTGGATGCTGATCTGGACTCCTCCGTGCCCGCTGACATTATCTCATCCACAGACAAACTTGgtgagg
Damalarand_mole-rat           TGGGGATTTTGGATGCTGATCTGGACTCCTCCGTGCCCGCTGACATTATCTCATCCACAGACAAACTTGGtgagg
naked_mole_rat                TGGGGATTTTGGATGCTGATCTGGACTCCTCCGTGCCCGCTGACATTATCTCATCCACAGACAAACTTGgtgagg
guinea_pig                    TGGGGATTTTGGATGCTGATCTGGATTCCTCCGTGCCCGCTGACATTATCTCATCCACAGACAAACTTGgtgagg
armadillo                     TGGGAATTCTGGATGCTGATCTGGACTCCTCCGTGCCAGCTGATATTATCTCATCCACAGACAAACTTGgtgagg
Tasm.devil                    TGGGAATTTTGGATGCTGATCTGGACTCCTCCGTTCCAGCTGATATTATCTCATCCACAGACAAACTTGgtgagg
opossum                       TGGGAATTTTGGATGCTGATCTGGACTCCTCCGTTCCAGCTGATATTATCTCATCCACAGACAAACTTGgtgagg
wallaby                       TGGGAATTTTGGATGCTGATCTGGACTCCTCCGTTCCAGCTGATATTATCTCATCCACAGACAAACTTGgtgagg
platypus                      TGGGGATCCTGGACGCCGATCTGGACTCGTCCGTCCCAGCAGACATAATCACATCCTCAGACCGACTGGgtgagg
duck                          TGGGCATCTTGGATGCGGACCTGGACTCGTCCGTTCCGGCCGATATTATCTCATCCACAGACAAACTGGgtgagg
zebrafinch                    TGGGCATCTTGGATGCAGATCTGGACTCCTCCCTTCCTGCCGATATTATCACATCCACAGACAAACTGGGtgagg
chicken                       TGGGCATCCTGGATGCGGATCTGGACTCATCCGTTCCAGCCGATATTATCACATCCACAGACAAACTGGgtgagg
turkey                        TGGGCATCCTGGATGCGGATCTGGACTCATCCGTTCCAGCCGATATTATCACATCCACAGACAAACTGGgtgagg
quail                         TGGGCATCCTGGATGCGGATCTGGACTCATCCGTTCCAGCCGATATTATCACATCCACAGACAAACTGGgtgagg
alligator                     TGGGAATCTTGGATGCTGATTTGGACTCGTCCGTTCCAGCTGATATAATCACATCCGCTGACAAACTGGgtgagg
desert_tortoise               TGGGAATCTTGGATGCAGATCTGGACTCGTGCGTTCCAGCTGATATTATCACATCCTCAGACAAACTGGgtgagg
turtle_softshell              TGGGGATCTTGGATGCAGATCTGGACTCGTGCGTGCCAACTGATATTATCACGTCCTCAGACAAACTGGgtgagg
turtle_painted                TGGGAATCTTGGATGCAGATCTGGACTCGTGCGTTCCAGCTGATATTATCACATCCTCAGACAAACTGGgtgagg
tuatara_lizard                TGGGAATCTTGGATGCAGATCTGGACTCGTCCATTCCAACAGATATTATCTCATCCACAGACAAACTGGgtgagg
anole_lizard                  TGGGCATCTTGGATGCTGATCTGGACTCTTCCGTTCCAGCTGATATTATCACTTCCACAGACAAACTGGgtgagg
python_Burmese                TGGGCATCTTGGATGCTGATCTGGACTCTTCCATTCCAGCCGATATTATCAGTTCCGCAGACAAACTGGgtgagg
xenopus_trop.                 TTGGCATTCTGGATGCAGATCTGGACTCCTGTGTGCCAGCAGATATTGTCACTTCCTCAGACAAACTCGGtaaag
xenopus_laevis                TTGGCATTCTGGATGCAGATCTGGACTCGTGCGTGCCAGCAGATATTGTCACGTCCTCAGACAAACTCGgtgagg
ocean_sunfish                 TCGGCATCATGGACGCTGATCTGGACTCGTGTGTTCCCACTGACATAATCACCTCCTCCGACAAGCTGGgtgagn
tilapia                       TCGGCATCATGGACGCCGATCTGGACTCATGTGTCCCCACTGACATTATCACGTCCTCCGACAAGCTGGgtgagg
zebra_mbuna                   TCGGCATCATGGACGCCGATCTGGATTCGTGTGTCCCCACTGACATTATCACGTCCTCCGACAAGCTGGgtgagg
stickleback                   TCGGCATCATGGACGCCGACTTGGACTCGTGTGTCCCCACCGACATAATCACCTCCTCCGACAAGCTGGgtgagg
climbing_perch                TCGGCATCATGGACGCCGATCTGGACTCATGTGTCCCCACTGACATTATCACCTCCTCTGACAAGCTGGgtgagg
ballan_wrasse                 TCGGCATCATGGACGCTGACTTGGACTCGTGTGTCCCCACTGACATTATCACCTCCTCCGACAAGCTGGGtgagg
swamp_eel                     TCGGCATCATGGACGCCGATCTGGACTCTTGTGTCCCCACTGACATTATCACCTCCGATGACAAACTGGgtgagg
zigzag_eel                    TCGGCATCATGGACGCTGATCTGGACTCGTGTGTCCCCACTGACATTATCACCTCCTCCGACAAACTGGgtgagg
tetraodon                     TGGGCATCATGGATGCTGATCTGGACTCCTGTGTTCCGACTGACATCATCACCTCGTCAGACAAGCTGGgtgagc
tigertail_seahorse            TTGGCATCATGGACGCTGATCTGGACGCGTGCGTGCCCACTGACATTATCACGTCCTCGGACAAGCTGGgtgagg
fugu                          TGGGCATCATGGATGCTGATCTGGACTCGTGTGTTCCGACTGACATTATAACCTCCTCAGACAAGCTGGgtgagc
tongue_sole                   TAGGCATCATGGACGCCGATCTGGATTCTTGTGTTCCCACTGACATAATCACTTCCTCCGACAAGCTGGgtgagg
amazon_molly                  TGGGCATCATGGACGCCGATCTGGACTCGTGCGTTCCCACTGACATTATCACATCGTCCGACAAGCTGGgtgagg
shortfin_molly                TGGGCATCATGGACGCCGATCTGGACTCGTGCGTTCCCACTGACATTATCACATCGTCCGACAAGCTGGgtgagg
bicolor_damselfish            TCGGCATCATGGACGCCGATCTGGACTCGTGCGTCCCCACTGACATTATCACGTCCTCCGACAAGCTGGGtgagg
zebrafish                     TTGGGATCATGGATGCTGATCTGGATTCATGCGTTCCCACCGACATTATTACATCATCGGATAAACTCGgtgagg
spotted_gar                   TCGGCATCATGGACGCAGATCTGGACTCTTGCGTCCCGACGGATATAATCACGTCCTCGGATAAACTCGgtgagg
channel_catfish               TGGGGATCATGGATGCCGATCTGGACTCGTGCGTCCCCACCGACATTATTACGTCTTCGGATAAACTCGgtgagg
cod                           TGGGCATCATGGACGCTGATCTGGACTCGCATGTTCCTACTGACATTATCACTTCGTCGGACAAGCTGGgtgagc
                              * ** **  **** ** **  ****  *     * **  * ** **  *    **    **    ** *** *
```

Figure S2 Example of exon skipping in HEK293 cells overexpressing U2AF65

10+, 10-, RNA products with and without *HGD* exon 10. *HGD* minigene assay was described previously (18). EV, empty vector.

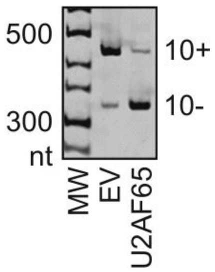

Figure S3 Usage of individual *OGDH* dBPs and MXE regulatory proteins

A, Alignment of 26 informative sequences across lariat junctions in control cells. Intron 4a reference sequence shown in at the top, dBP adenines are in green. Mutations around lariat junctions introduced by RT or PCR are highlighted in yellow. The 5’ss GT dinucleotide is in red. **B,** *DBR1* knockdown does not alter exon 4a/4b ratios. **C,** Immunoblots (upper panels) from HEK293 cells depleted of DBR1 and TIA proteins or overexpressing PUF60. Lower panels show PCR products containing lariat junctions (red rectangles). **D,** Usage of individual human dBPs in HEK293 cells lacking or overexpressing *OGDH* MXE regulators. Clones are sorted by dBPs; their numbers are summarized in Table 1. **E,** Chicken dBP usage in HEK293 cells lacking or overexpressing *OGDH* MXE regulators. Clone numbers are shown in Table 2. **F,** Mapping of *OGDH* exon 4a BP. BP is denoted by a circle. The 5’ end of the reporter intron is shown as a black rectangle. Location of exon 4a BP corresponds to a pile up of SF3B4 eCLIP reads (Figure 2B).

A

```
                      +25   +31  +36 +41
Clone     gctgtaaatgctaattttaatgtaatttac
-----------------------------------------------------------------------
4-5/1     GCTGTAAATGCTAATTTTAATGTAT-----GTTT-TTTGCCTTTTTTT-CATGTAAATTAT
4-5/2     GCTGTAAATGCTAATTTTTAT---------GTTTGTTTGCCTTTTTTT-CATGTAAATTAT
4-5/3     GCTGTAAATGCTAATTTTTAT---------GTTTGTTTGCCTTTTTTT-CATGTAAATTAT
4-5/4     GCTGTAAATGCTAT----------------GTTTGTTTGCCTTTTTTT-CATGTAAATTAT
4-5/5     GCTGTAAATGCTAATTTTAATGTA------GTTTGTTTGCCTTTTTTT-CATGTAAATTAT
4-5/6     GCTGTAAATGCTAATTTTAATGTTT-----GTTTGTTTGCCTTTTTTTTCATGTAAATTAT
BP1       GCTGTAAATGCTAATTTTAT----------GTTTGTTTGCCTTTTTTT-CATGTAAATTAT
BP2       GCTGTAAATGCTAATTTTTA----------GTTTGTTTGCCTTTTTTT-CATGTAAATTAT
BP3       GCTGTAAATGCTAATTTTT-----------GTTTGTTTGCCTTTTTTT-CATGTAAATTAT
BP4       GCTGTAAATGCCAATTTTAATGTAATTTTTGTTTGTTTGCCTTTTTTT-CATGTAAATTAT
BP5       GCTGTAAATGCTAATTTTAATGTAT-----GTTTGTTTGCCTTTTTTT-CATGTAAATTAT
BP6       GCTGTAAATGCTAATTTTAT----------GTTTGTTTGCCTTTTTTT-CATGTAAATTAT
BP7       GCTGTAAATGCT------------------GTTTGTTTGCCTTTTTTT-CATGTAAATTAT
BP9       GCTGTAAATGCTAATTTTAATGTAT-----GTTTGTTTGCCTTTTTTT-CATGTAAATTAT
BP10      GCTGTAAATGCTTT----------------GTTTGTTTGCCTTTTTTT-CATGTAAATTAT
BP11      GCTGTAAATGCTAATTTTAATGTAT-----GTTTGTTTGCCTTTTTTT-CATGTAAATTAT
BP12      GCTGTAAATGCTAT----------------GTTTGTTTGCCTTTTTTT-CATGTAAATTAT
BP13      GCTGTAAATGCTAT----------------GTTTGTTTGCC-TTTTTT-CATGTAAATTAT
BP14      GCTGTAAATGCTTT----------------GTTTGTTTGCCTTTTTTT-CATGTAAATTAT
BP15      GCTGTAAATGCTAATTTTAT----------GTTTGTTTGCCTTTTTTT-CATGTAAATTAT
BP16      GCTGTAAATGCT------------------GTTTGTTTGCCTTTTTTT-CATGTAAATTAT
BP17      GCTGTAAATGCTAATTTTAT----------GTTTGTTTGCCTTTTTTT-CATGTAAATTAT
BP20      GCTGTAAATGCTTT----------------GTTTGTTTGCCTTTTTTT-CATGTAAATTAT
BP22      GCTGTAAATGCTAATTTTAT----------GTTTGTTTGCCTTTTTTT-CATGTAAATTAT
BP23      GCTGTAAATGCTAATTTTTAT---------GTTTGTTTGCCTTTTTTT-CATGTAAATTAT
BP24      GCTGTAAATGCTTT----------------GTTTGTTTGCCTTTTTTT-CATGTAAATTAT
```

B

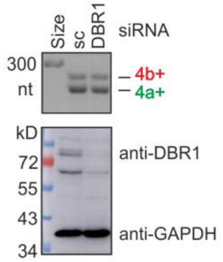

C

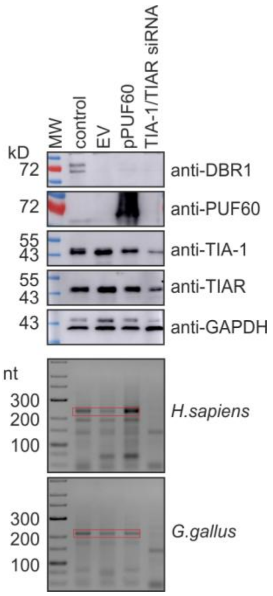

D

Control cells (human transcripts)

```
                                               BP+25 BP+31 BP+36 BP+41
Clone           gagaattaagctgtaaatgctaattttaatgtaatttta
----------------------------------------------------------------------------
H2_EV_N         GAGAATTAAGCTGTAAATGCT------------------GTTTGTTTGCCTTGTTTTCATG
con4            GAGAATTAAGCTGTAAATGCT------------------GTTTGTTTGCCTTTTTTTCATG
con2            GAGAATTAAGCTGTAAATGCT------------------GTTTGTTTGCCTTTTTTTCATG
con20           GAGAATTAAGCTGTAAATGCT------------------GTTTGTTTGCCTTTTTTTCATG
con9            GAGAATTAAGCTGTAAATGCT------------------GTTTGTTTGCCTTTTTTTCATG
con22           GAGAATTAAGCTGTAAATGCTT-----------------GTTTGTTTGCCTTTTTTTCATG
H2_EV_S         GAGAATTAAGCTGTAAATGTTT-----------------GTTTGTTTGCCTTTTTTTCATG
con30           GAGAATTAAGCTGTAAATGCTAT----------------GTTTGTTTGCC-TTTTTTCATG
H-F2-4          GAGAATTAAGCTGTAAATGCTAT----------------GTTTGTTTGCCTTTTTTTCATG
H-F2-6          GAGAATTAAGCTGTAAATGCTAT----------------GTTTGTTTGCCTTTTTTTCATG
con13           GAGAATTAAGCTGTAAATGCTAT----------------GTTTGTTTGCCTTTTTTTCATG
con36           GAGAATTAAGCTGTAAATGCTAT----------------GTTTGTTTGCCTTTTTTTCATG
con29           GAGAATTAAGCTGTAAATGCTTT----------------GTTTGTTTGCCTTTTTTTCATG
H2_EV_U         GAGAATTAAGCTGTAAATGCTAT----------------GTTTGTTTGCCTTTTTTTCATG
con7            GAGAATTAAGCTGTAAATGCTAATTTTA-----------GTTTGTTTGCCTTTTTTTCATG
con14           GAGAATTAAGCTGTAAATGCTAATTTTT-----------GTTTGTTTGCCTTTTTTTCATG
con27           GAGAATTAAGCTGTAAATGCTAATTTTT-----------GTTTGTTTGCCTTTTTTTCATG
con31           GAGAATTAAGCTGTAAATGCTAATTTTT-----------GTTTGTTTGCCTTTTTTTCATG
con8            GAGAATTAAGCTGTAAATGCTGATTGCTT----------GTTTGTTTGCCTTTTTTTCATG
con33           GAGAATTAAGCTGAAAATGCTAATTTTAT----------GTTTGTTTGCCTTTTTTTCATG
con12           GAGAATTAAGCTGTAAATGCTAATTTTAC----------GTTTGTTTGCCTTTTTTTCATG
con3            GAGAATTAAGCTGTAAATGCTAATTTTAT----------GTTTGTTTGCCTTTTTTTCATG
H-F2-7          GAGAATTAAGCTGTAAATGCTAATTTTTT----------GTTTGTTTGCCTTTTTTTCATG
con18           GAGAATTAAGCTGTAAATGCTAATTTTTT----------GTTTGTTTGCCTTTTTTTCATG
H-F2-3          GAGAATTAAGCTGTAAATGCAAATTTTAT----------GTTTGTTTGCCTTTTTTTCATG
con16           GAGAATTAAGCTGTAAATGCTAATTTTAT----------GTTTGTTTGCCTTTTTTTCATG
con17           GAGAATTAAGCTGTAAATGCTAATTTTAT----------GTTTGTTTGCCTTTTTTTCATG
con26           GAGAATTAAGCTGTAAATGCTAATTTAATG---------GTTTGTTTGCCTTTTTTTCATG
con28           GAGAATTAAGCTGTAAATGCTAATTTAATG---------GTTTGTTTGCCTTTTTTTCATG
H2_EV_T         GAGAATTAAGCTGTAGATGCTAATTTAT-----------GTTTGTTTGCCATTTTTTCATG
H2_EV_P         GAGAATTAAGCTGTAAATGCTAATTTTTA----------GTTTGTTTGCCTTTTTTTCATG
H2_EV_E         GAGAATTAAGCTGTAAATGCTAATTTTAT----------GTTTGTTTGACTTTTTTTCATG
H2_EV_B         GAGAATTAAGCTGTAAATGCTAATTTTAT----------GTTTGTATGCCTTTTTTTCATG
H2_EV_C         GAGAATTAAGCTGTAAATGCTAATTTTAT----------GTTTGTTTGCCTTTTTTTCATG
H2_EV_D         GAGAATTAAGCTGTAAATGCTAATTTTAT----------GTTTGTTTGCCTTTTTTTCATG
H2_EV_K         GAGAATTAAGCTGTAAATGCTAATTTTAT----------GTTTGTTTGCCTTTTTTTCATG
H2_EV_R         GAGAATTAAGCTGTAAATGCTAATTTTAT----------GTTTGTTTGCCTTTTTTTCATG
H-F2-2          GAGAATTAAGCTGTAAATGCTAATTTTAATGTT------GTTTGTTTGCCTTTTTTTCATG
H-F2-5          GAGAATTAAGCTGTAAATGCTAATTTTAATGTA------GTTTGTTTGCCTTTTTTTCATG
con1            GAGAATTAAGCTGTAAATGCTAATTTTAATGTA------GTTTGTTTGCCTTTTTTTCATG
con32           GAGAATTAAGCTGTAAATGCTAATTTTATCCAG------GTTTGTTTGCCTTTTTTTCATG
H2_EV_O         GAGAATTAAGCTGTAAATGCTAATTTTAATGTA------GTTTGTTCGCCTTTTTTTCATG
H2_EV_I         GAGAATTAAGCTGTAAATGCTAATTTTAATATA------GTTTGTTTGCCTTTTTTTCATG
H2_EV_A         GAGAATTAAGCTGTAAATGCTAATTTTAATGTA------GTTTGTTTGCCTTTTTTTCATG
H2_EV_L         GAGAATTAAGCTGTAAATGCTAATTTTAATGTA------GTTTGTTTGCCTTTTTTTCATG
H2_EV_M         GAGAATTAAGCTGTAAATGCTAATTTTAATGTA------GTTTGTTTGCCTTTTTTTCATG
H-F2-1          GAGAATTAAGCTGTAAATGCTAATTTTAATGTTT-----GTTTGTTTGCCTTTTTTTCATG
con10           GAGAATTAAGCTGTAAATGCTAATTTTAATGTAT-----GTTTGTTTGCCTTTTTTTCATG
con35           GAGAATTAAGCTGTAAATGCTAATTTTAATGTAT-----GTTTGTTTGCCTTTTTTTCATG `
H2_EV_G         GAGAATTAAGCTGTAAATGCTAATTTTGATGTAATTTTAGTTTGTTTGCCTTTTTTTCATG
```

Cells overexpressing PUF60 (human transcripts)

```
                               BP+25 BP+31 BP+36 BP+41
Clone         gagaattaagctgtaaatgctaattttaatgtaatttta
---------------------------------------------------------------------------
H2_PUF_M      GAGAATTAAGCTGTAAATGC-------------------GTTTGTTTGCCTTTTTTTCATG
PUF-BP7       GAGAATTAAGCTGTAAATGCT------------------GTTTGTTTGCCTTTTTTTCATG
PUF-BP20      GAGAATTAAGCTGTAAATGCT------------------GTTTGTTTGCCTTTTTTTCATG
PUF-BP21      GAGAATTAAGCTGTAAATGCT------------------GTTTGTTTGCCTTTTTTTCATG
PUF-BP27      GAGAATTAAGCTGTAAATGCT------------------GTTTGTTTGCCTTTTTTTCATG
PUF-BP14      GAGAATTAAGCTGTAAATGCTA-----------------GTTTGTTTGCCTTTTTTTCATG
PUF-BP15      GAGAATTAAGCTGTAAATGCTA-----------------GTTTGTTTGCCTTTTTTTCATG
PUF-BP32      GAGAATTAAGCTGCTAATTTTAT----------------GGTTGTTTGCCTTTTTTTCATG
PUF-BP18      GAGAATTAAGCTGTAAATGCTTT----------------GTTTGTTTGCCTTTTTTTCATG
H-P-3         GAGAATTAAGCTGTAAATGCTAT----------------GTTTGTTTGCCTTTTTTTCATG
PUF-BP13      GAGAATTAAGCTGTAAATGCTAT----------------GTTTGTTTGCCTTTTTTTCATG
PUF-BP16      GAGAATTAAGCTGTAAATGCTAT----------------GTTTGTTTGCCTTTTTTTCATG
PUF-BP31      GAGAATTAAGCTGTAAATGCTAT----------------GTTTGTTTGCCTTTTTTTCATG
PUF-BP34      GAGAATTAAGCTGTAAATGCTAT----------------GTTTGTTTGCCTTTTTTTCATG
PUF-BP35      GAGAATTAAGCTGTAAATGCTAT----------------GTTTGTTTGCCTTTTTTTCATG
H2_PUF_B      GAGAATTAAGCTGTAAATGCTAT----------------GTTTGTTTGCCTTTTTTTCATG
H2_PUF_S      GAGAATTAAGCTGTAAATGCTAC----------------GTTTGTTTACCTTTTTTTCATG
H2_PUF_C      GAGAATTAAGCTGTAAATGCTTT----------------GTTCGTTTGCCTTTTTTTCATG
PUF-BP24      GAGAATTAAGCTGTAAATGCTAATTTT------------GTTTGTTTGCCTTTTTTTCATG
PUF-BP41      GAGAATTAAGCTGTAAATGCTAATTTTAT----------GTTTGTTTGCCTTTTTTTCATG
PUF-BP42      GAGAATTAAGCTGTAAATGCTAATTTTAT----------GGTTGTTTGCCTTTTTTTCATG
H-P-1         GAGAATTAAGCTGTAAATGCTAATTTTAT----------GTTTGTTTGCCTTTTTTTCATG
PUF-BP8       GAGAATTAAGCTGTAAATGCTAATTTTAT----------GTTTGTTTGCCTTTTTTTCATG
PUF-BP9       GAGAATTAAGCTGTAAATGCTAATTTTAT----------GTTTGTTTGCCTTTTTTTCATG
PUF-BP12      GAGAATTAAGCTGTAAATGCTAATTTTAT----------GTTTGTTTGCCTTTTTTTCATG
PUF-BP23      GAGAATTAAGCTGTAAATGCTAATTTTAT----------GTTTGTTTGCCTTTTTTTCATG
H2_PUF_P      GAGAATTAAGCT-TAAGTGCTAATTTTAT----------GTTTGTTTGCCTTTTTTTCATG
H2_PUF_L      GAGAATTAAGCTGTAAATGCTAATTTTAT----------GTTTGTTTGCCTTTTTTTCATG
H2_PUF_O      GAGAACTAAGCTGTAAATGCTAATTTTAT----------GTTTGTTTGCCTTTTTTTCATG
H2_PUF_H      GAGAATTAAGCTGTAAATGCTAATTTTAT----------GTTTGTTTGCCTTTTTTTCATG
H2_PUF_R      GAGAATTAAGCTGTAAATGCTAATTTTAT----------GTTTGTTTGCCTTTTTTTCATG
H2_PUF_I      GAGAATTAAGCTGTAAATGCTAATTTGTTT---------GTTTGTTTGCCTTTTTTTCATG
PUF-BP33      GAGAATTAAGCTGTAAATGCTAATTTTATCCAG------GTTTGTTTGCCTTTTTTTCATG
H-P-6         GAGAATTAAGCTGTAAATGCTAATTTTAATGTT------GTTTGTTTGCCTTTTTTTCATG
PUF-BP11      GAGAATTAAGCTGTAAATGCTAATTTTAATGTT------GTTTGTTTGCCTTTTTTTCATG
PUF-BP22      GAGAATTAAGCTGTAAATGCTAATTTTAATGTT------GTTTGTTTGCCTTTTTTTCATG
PUF-BP28      GAGAATTAAGCTGTAAATGCTAATTTTAATGTA------GTTTGTTTGCCTTTTTTTCATG
PUF-BP36      GAGAATTAAGCTGTAAATGCTAATTTTAATGTA------GTTTGTTTGCCTTTTTTTCATG
PUF-BP37      GAGAATTAAGCTGTAAATGCTAATTTTAATGTA------GTTTGTTTGCCTTTTTTTCATG
PUF-BP19      GAGAATTAAGCTGTAAATGCTAATTTTAATGTAT-----GTTTGTTTGCCTTTTTTTCATG
PUF-BP26      GAGAATTAAGCTGTAAATGCTAATTTTAATGTTG-----GTTTGTTTGCCTTTTTTTCATG
H2_PUF_N      GAGAATTAAGCTGTAAATGCTAATTTTAATGTTT-----GTTTGTTTGCCTTTTTTTCATG
H2_PUF_F      GAGAATTAAGCTGTAAATGCTAATTTTAATGTAT-----GTTTGTTTGCCTTTTTTTCATG
```

Cells lacking TIA-1 and TIAR (human transcripts)

```
                               BP+25 BP+31 BP+36 BP+41
Clone       gagaattaagctgtaaatgctaattttaatgtaatttta
-------------------------------------------------------------------------
TIA-34      GAGAATTAAGCTGTAAATGCT------------------GTTTGTTTGCCTTTTTTTCATG
TIA-45      GAGAATTAAGCTGTAAATGCT------------------GTTTGTTTGCCTTTTTTTCATG
TIA-35      GAGAATTAAGCTGTAAATGCTA-----------------GTTTGTTTGCCTTTTTTTCGTG
TIA-12      GAGAATTAAGCTGTAAATGCTA-----------------GTTTGTTTGCCTTTTTTTCATG
TIA-18      GAGAATTAAGCTGTAAATGCTA-----------------GTTTGTTTGCCTTTTTTTCATG
H2_TIA_C    GAGAATTAAGCTGTAAATGCTT-----------------GTTTGTTTGCCTTTTTTTCATG
TIA-30      GAGAATTAAGCTGTAAATGCTTT----------------GTTTGTTTGCGTTTTTTTCTTG
TIA-37      GAGAATTAAGCTGTAAATGCTTT----------------GTTTGTTTGCCTTTTTTTCATG
TIA-22      GAGAATTAAGCTGTAAATGCTAT----------------GTTTGTTTGCCTTTTTTTCATG
TIA-11      GAGAATTAAGCTGTAAATGCTAA----------------GTTTGTTTGCCTTTTTTTCATG
H-T-6       GAGAATCAAGCTGTAAATGCTAT----------------GTTTGTTTGCCTTTTTTTCATG
H-T-7       GAGAATTAAGCTGTAAATGCTAT----------------GTTTGTTTGCCTTTTTTTCATG
TIA-36      GAGAATTAAGCTGTAAATGCTAT----------------GTTTGTTTGCCTTTTTTTCATG
TIA-42      GAGAATTAAGCTGTAAATGCTAT----------------GTTTGTTTGCCTTTTTTTCATG
TIA-25      GAGAATTAAGCTGTAAATGCTAATTTTA-----------GTTTGTTTGCCTTTTTTTCATG
H2_TIA_E    GAGAATTAAGCTGTAAATGCTAATTTTT-----------GTTTGTTTGCCTTTTTTTCATG
H2_TIA_B    GAGAATTAAGCTGTAAATGCTAATTTTAT----------GTTTGTTTGCCTTTTTTTCATG
H2_TIA_O    GAGAATTAAGCTGTAAATGCTAATTTTAT----------GTTTGTTTGCCTTTTTTTCATG
H2_TIA_S    GAGAATTAAGCTGTAAATGCTAATTTTAT----------GTTTGTTTGCCTTTTTTTCATG
H2_TIA_U    GAGAATTAAGCTGTAAATGCTAATTTTAT----------GTTTGTTTGCCTTTTTTTCATG
TIA-44      GAGAATTAAGCTGTAAATGCTAATTTTAT----------GTTTGTTTGCCTTTTTTTCATG
H-T-4       GAGAATTAAGCTGTAAATGCTAATTTTAT----------GTTTGTTTGCCTTTTTTTCATG
TIA-24      GAGAATTAAGCTGTAAATGCTAATTTTAT----------GTTTGTTTGCCTTTTTTTCATG
TIA-38      GAGAATTAAGCTGTAAATGCTAATTTTAT----------GTTTGTTTGCCTTTTTTTCATG
TIA-39      GAGAATTAAGCTGTAAATGCTAATTTTAT----------GTTTGTTTGCCTTTTTTTCATG
TIA-32      GAGAATTAAGCTGTAAATGCTAATTTTTT----------GTTTGTTTGCCTTTTTTTCATG
TIA-8       GAGAATTAAGCTGTAAATGCTAATTTTAATGTA------GTTTGTTTGCCTTTTTTTCATG
TIA-28      GAGAATTAAGCTGTAAATGCTAATTTTAATGTA------GTTTGTTTGCCTTTTTTTCATG
H2_TIA_G    GAGAATTAAGCTGTAAATGCTAATTTTAATGTA------GTTTGTTTGCCTTTTTTTCATG
H2_TIA_H    GAGAATTAAGCTGTAAATGCTAATTTTAATGTA------GTTTGTTTGCCTTTTTTTCATG
H2_TIA_P    GAGAATTAAGCTGTAAATGCTAATTTTAATGTA------GTTTGTTTGCCTTTTTTTCATG
TIA-15      GAGAATTAAGCTGTAAATGCTAATTTTAATGTAT-----GTTTGTTTGCCTTTTTTTCATG
TIA-23      GAGAATTAAGCTGTAAATGCTAATTTTAATGTAT-----GTTTGTTTGCCTTTTTTTCATG
TIA-26      GAGAATTAAGCTGTAAATGCTAATTTTAATGTAT-----GTTTGTTTGCCTTTTTTTCATG
TIA-43      GAGAATTAAGCTGTAAATGCTAATTTTAATGTAT-----GTTTGTTTGCCTTTTTTTCATG
H2_TIA_F    GAGAATTAAGCTGTAAATGCTAATTTTAATGTAT-----GTTTGTTTGCCTTTTTTTCATG
H-T-3       GAGAATTAAGCTGTAAATGCTAATTTTAATGTAATTTT-GTTTGTTTGCCTTTTTTTCATG
TIA-13      GAGAATTAAGCTGTAAATGCTAATTTTAATGTAATTTT-GTTTGTTTGCCTTTTTTTCATG
H2_TIA_N    GAGAATTAAGCTGTAAATGCTAATTTTAATGTAATTTTTGTTTGTTTGCCTTTTTTTCATG
```

E

```
Control cells          (chicken transcripts)

                                BP+25 BP+31 BP+36 BP+41
Clone        gtgagaattactctgcaaattaattcgaatgtaatttaa
-------------------------------------------------------------------
K-EV-U       GTGAGAATTACTCTGCAAATTAT----------------GTTTGTTTGCCTTTTT
K-EV-L       GTGAGAATTACTCTGCAAATTAATTCGTG----------GTTTGTTTGCCTTTTT
K-EV-D       GTGAGAATTACTCTGCAAATTAATTCGAATGTAT-----GTTTGTTTGCCTTTTT
K-EV-R       GTGAGAATTACTCTGCAAATTAATTCGAATGTAT-----GTTTGTTTGCCTTTTT
K-EV-E       GTGAGAATTACTCTGCAAATTAATTCGAATGTAATTTG-GTTGGTTGGCCTTTTT
K-EV-M       GTGAGAATTACTCTGCAAATTAATTCGAATGTAATTTT-GTTTGTTTGCCTTTTT
K-EV-N       GTGAGAATTACTCTGCAAATTAATTCGAATGTAATTTA-GTTTGTTTGCCTTTTT
K-EV-H       GTGAGAATTACTCTGCAAATTAATTCGAATGTAATTTACGTTTGTTTGCCTTTTT
K-EV-P       GTGAGAATTACTCTGCAAATTAATTCGAATGTAATTTTTGTTTGTTTGCCTTTTT
K-EV-B       GTGAGAATTACTCTGCAAATTAATTCGAATGTAATTTATGTTTGTTTGCCTTTTT
K-EV-C       GTGAGAATTACTCTGCAAATTAATTCGAATGTAATTTATGTTTGTTTGCCTTTTT
K-EV-I       GTGAGAATTACTCTGCAAATTAATTCGAATGTAATTTATGTTTGTTTGCCTTTTT
K-EV-O       GTGAGAATTACTCTGCAAATTAATTCGAATGTAATTTATGTTTGTTTGCCTTTTT
K-EV-T       GTGAGAATTACTCTGCAAATTAATTCGAATGTAATTTTTGTTTGTTTGCCTTTTT

Cells overexpressing PUF60         (chicken transcripts)

                                BP+25 BP+31 BP+36 BP+41
Clone         gtgagaattactctgcaaattaattcgaatgtaatttaa
--------------------------------------------------------------------
K-PUF-F       GTGAGAATTACTCTGCAAATTAATTCGAATGTAT-----GTTTGTTTGCCTTTTT
K-PUF-O       GTGAGAATTACTCTGCAAATTAATTCGAATGTAT-----GTTTGTTTGCCTTTTT
K-PUF-G       GTGAGAATTACTCTGCAAATTAATTCGAATGTAATTT--GTTTGTTTGCCTTTTT
K-PUF-L       GTGAGAATTACTCTGCAAATTAATTCGAATGTAATTTT-GTTTGTTTGCCTTTTT
K-PUF-C       GTGAGAATTACTCTGCAAATTAATTCGAATGTAATTTT-GTTTGTTTGCCTTTTT
K-PUF-K       GTGAGAATTACTCTGCAAATTAATTCGAATGTAATTTA-GTTTGTTTGCCTTTTT
K-PUF-D       GTGAGAATTACTCTGCAAATTAATTCGAATGTAATTTTAGTTTGTTTGCCTTTTT
K-PUF-E       GTGAGAATTACTCTGCAAATTAATTCGAATGTAATTTATGTTTGTTTGCCTTTTT
K-PUF-J       GTGAGAATTACTCTGCAAATTAATTCAAATGTAATTTTTGTTTGTTTGCCTTTTT
K-PUF-B       GTGAGAATTACTCTGCAAATTAATTCGAATGTAATTTATGTTTGTTTGCCTTTTT
K-PUF-A       GTGAGAATTACTCTGCAAATTAATTCGAATGTAATTTATGTTTGTTTGCCTTTTT
K-PUF-H       GTGAGAATTACTCTGCAAATTAATTCGAATGTAATTTATGTTTGTTTGCCTTTTT
K-PUF-I       GTGAGAATTACTCTGCAAATTAATTCGAATGTAATTTATGTTTGTTTGCCTTTTT
K-PUF-M       GTGAGAATTACTCTGCAAATTAATTCGAATGTAATTTATGTTTGTTTGCCTTTTT

Cells lacking TIA-1 and TIAR       (chicken transcripts)

                                BP+25 BP+31 BP+36 BP+41
Clone         gtgagaattactctgcaaattaattcgaatgtaatttaa
----------------------------------------------------------------
K-TIA-C       GTGAGGATTACTCTGCAAATTAATTCGAATGTA------GTTTGTTTGCCT
K-TIA-K       GTGAGAATTACTCTGCAAATTAATTCGAATGTA------GTTTGTTTGCCT
K-TIA-O       GTGAGAATTACTCTGCAAATTAATTCGAATGTA------GTTTGTTTGCCT
K-TIA-E       GTGAGAATTACTCTGCAAATTAATTCGAATGTAT-----GTTTGTTTGCCT
K-TIA-L       GTGAGAATTACTCTGCAAATTAATTCGAATGTAA-----GTTTGTGTGCGT
K-TIA-J       GTGAGAGTTACTCTGCAAATTAATTCGAATGTAATTTT-GTTTGTTTGCCT
K-TIA-A       GTGAGAATTACTCTGCAAATCAATTCGAATGTAATTTT-GTTTGTTTGCCT
K-TIA-B       GTGAGAATTACTCTGCAAATTAATTCGAATGTAATTTT-GTTTGTTTGCCT
K-TIA-D       GTGAGAATTACTCTGCAAATTAATTCGAATGTAATTTT-GTTTGTTTGCCT
K-TIA-G       GTGAGAATTACTCTGCAAATTAATTCGAATGTAATTTATGTTTGTTTGCCT
K-TIA-H       GTGAGAATTACTCTGCAAATTAATTCGAATGTAATTTATGTTTGTTTGCCT
K-TIA-M       GTGAGAATTACTCTGCAAATTAATTCGAATGTAATTTATGTTTGTTTGCCT
```

F

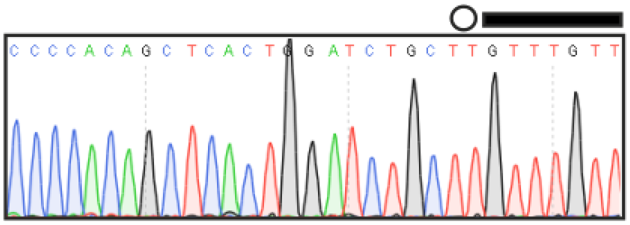

Figure S4 Solitary BPs: splicing activities of systematically mutated BP consensus motifs in *TSC2* intron 38 and in *F9* intron 1

The WT reporter construct (19) was mutated at the indicated positions of extended BP motif (denoted by a rectangle in the middle panel; BP adenine is at position 0) and transfected into HEK293 cells. Total RNA was extracted 24 hours later, treated with DNase, reversed transcribed with oligo-d(T) primers, amplified with vector-specific primers and separated by electrophoresis (upper panel). RNA products (schematically shown to the left) are denoted by boxes (exons) and lines (introns). The relative abundance of spliced products measured from two independent transfections is shown in the lower panel. US, unspliced products; IR, intron retention; CS, canonical splicing; ES, exon skipping. Error bars, SDs. Mutations with significant impact on splicing are in red. Putative silencing and enhancing mutations are in pink and blue, respectively. The first exon of the *F9* minigene employs a cryptic 5’ splice site, as described (19).

TSC2

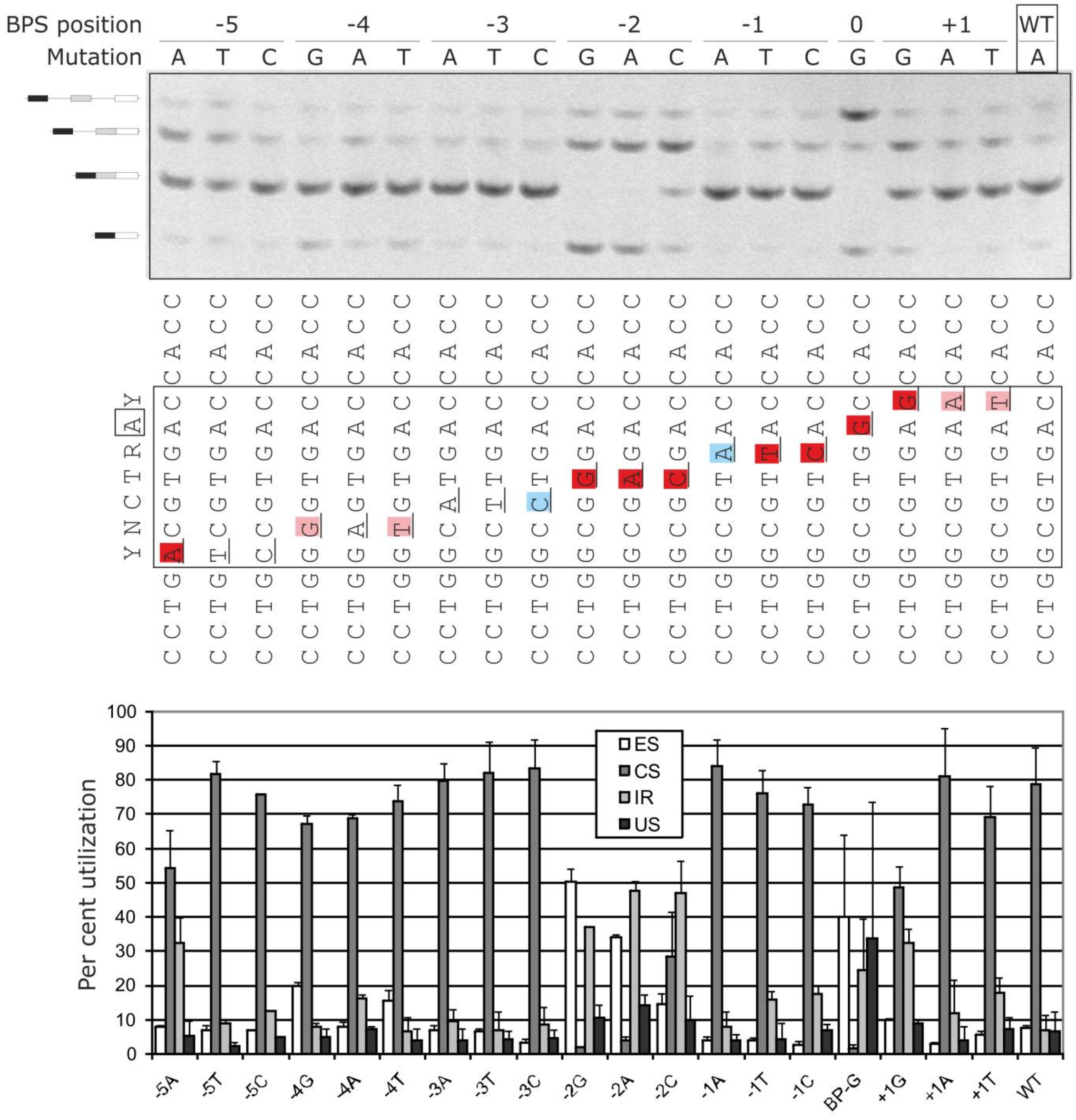

F9

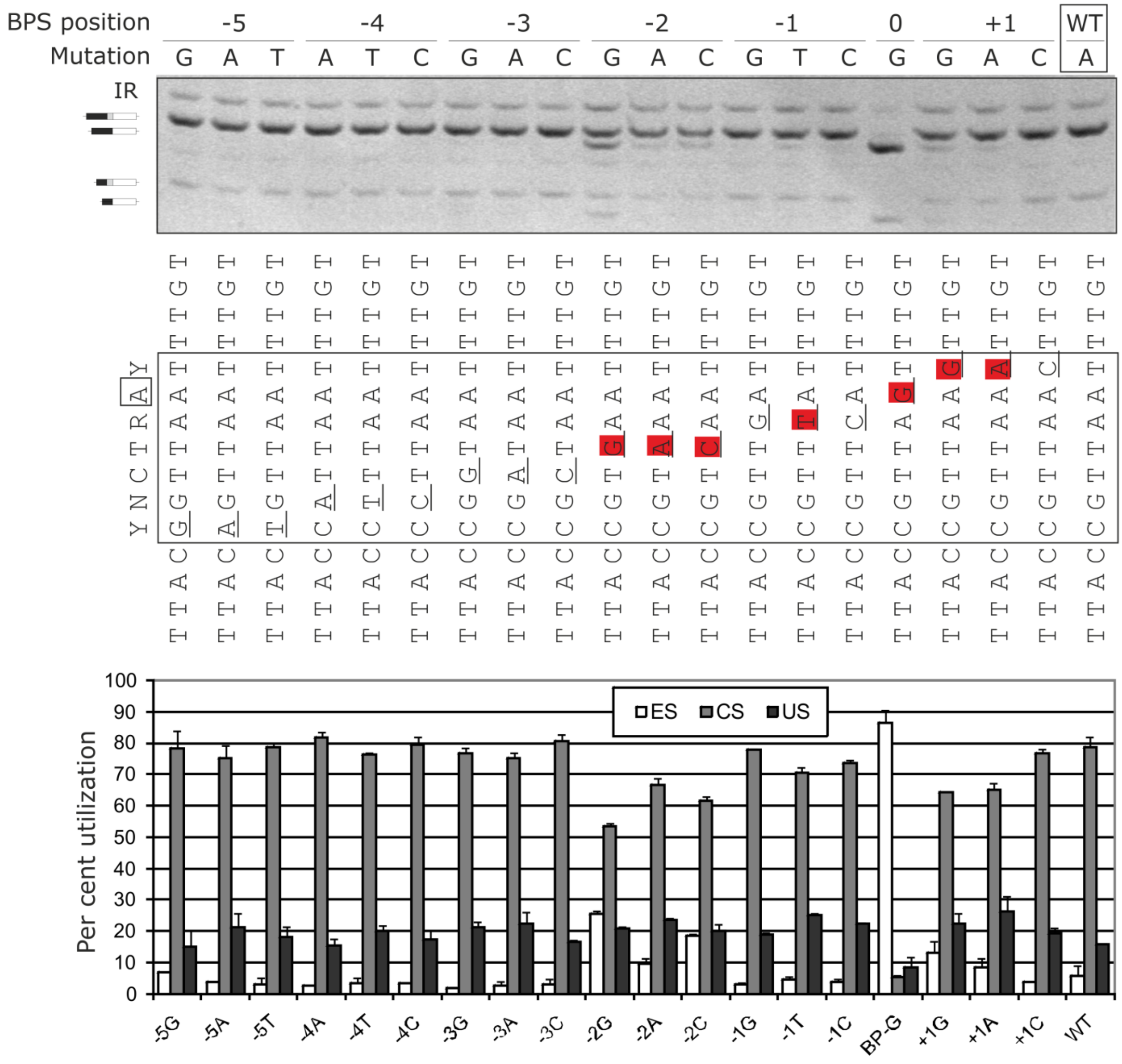
BPS position
-5
-4
-3
-2
-1
0
+1
WT
Mutation
G A T A T C G A C G A C G T C G G A C A
IR
Y N C T R A Y
Per cent utilization
ES CS US
-5G -5A -5T -4A -4T -4C -3G -3A -3C -2G -2A -2C -1G -1T -1C BP-G +1G +1A +1C WT

Figure S5 MegaPPT as a robust substructured platform for regulating *OGDH* MXEs

Upper panel: Exon 4b usage upon deletions (del1-4, Figure 3A) of megaPPT subregions. Columns are means of 2 transfections; error bars are SDs. Asterisks denote significant changes in isoform 4b+ usage (P<0.05, one-way ANOVA with post-hoc Dunnett's tests). *Middle panel:* PCR products digested with PvuII, which cuts only exon 4b. Spliced products are shown schematically to the right and in Figure 1G. *Lower panel:* Uncut products separated by an extended electrophoresis run.

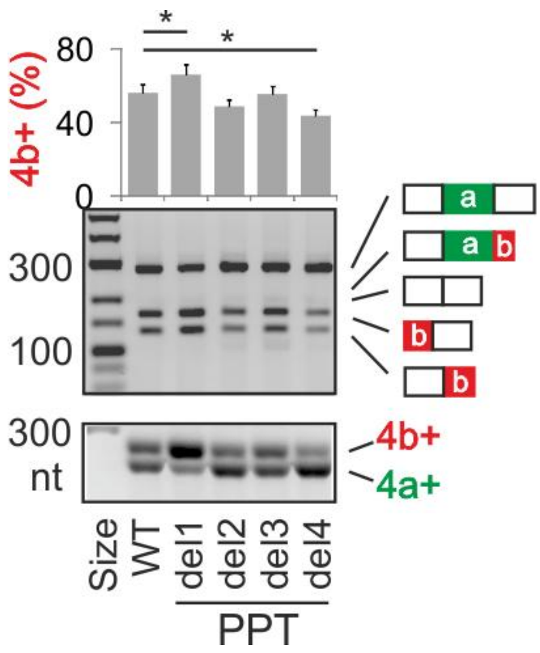

Figure S6 Conserved regulation of *OGDH* MXEs by U-binding proteins in vertebrates

A, Splicing of exogenous vertebrate transcripts derived from *Homo sapiens (Hs)*, *Monodelphis domestica (Md)*, *Gallus gallus (Gg)*, *Xenopus laevis (Xl)*, *Gadus morhua (Gm)* and *Danio rerio (Dr)* in two cell lines. Spliced products are shown to the right; asterisk denotes minor products or heteroduplexes. **B,** A lack of isoform 4a+ in exogenous bird *Ogdh* mRNAs is insensitive to deletions in the upstream intron. Bird isoform 4a+ was found in viscera (Figure S9D) and was reported in chicken lymphocytes (NM_001031382.1) (20). **C,** Coexpression of species-specific reporters and splicing factors that regulate exon 4a/4b ratios. A corresponding immunoblot is bottom right. **D,** Conserved TIA-1/TIAR regulation of exons 4a and 4b in transcripts derived from the indicated species. **E,** Immunoblots from depleted cells for panel **D.**

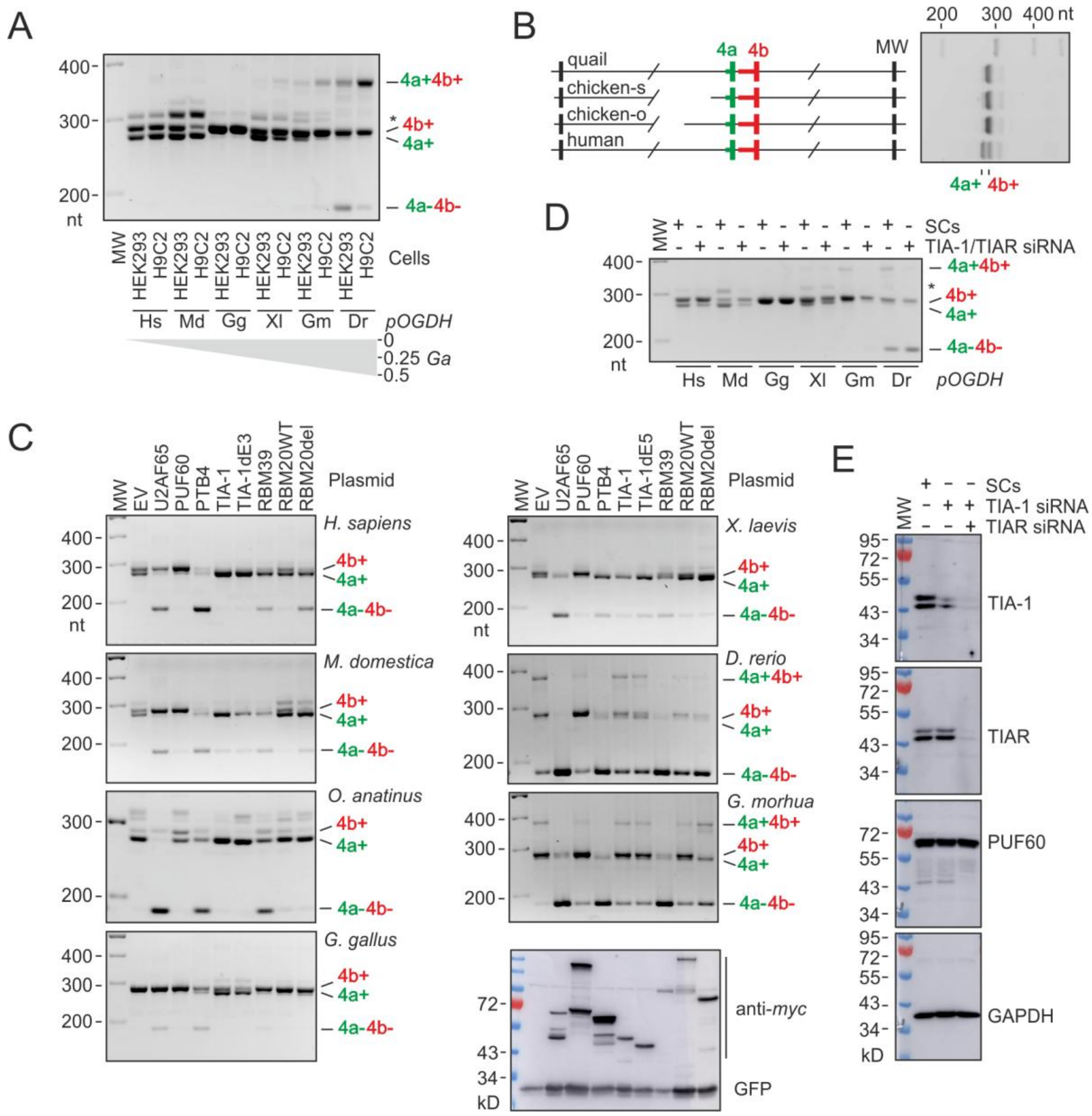

Figure S7 Evolutionary transitions between cytosine- and uridine-rich megaPPTs in introns with dBPs

Uridine (red lines) and cytosine (black lines) fractions (y-axis) were computed for sequences between the most 3’ dBP and position -3 relative to the 3’ss. Their mean length in the indicated orthologues is at the top right corner. *OGDH* dBPs are mapped in Figures 3 and 6. Rat *Actn1* and *Tpm1* dBPs were reported previously (21,22). For *KCNMA1*, we selected a putative dBP that was most conserved, as none was predicted by SVM-BP (10). MegaPPTs are located between *OGDH* exons 4a and 4b **(A)**, *ACTN1* (actinin) exons 2a and 2b **(B)**, *TPM1* (tropomyosin) exons 2a and 2b **(C)** and *KCNMA1* (Ca^{2+}-activated potassium channel) exons 9a and 9b **(D)**.

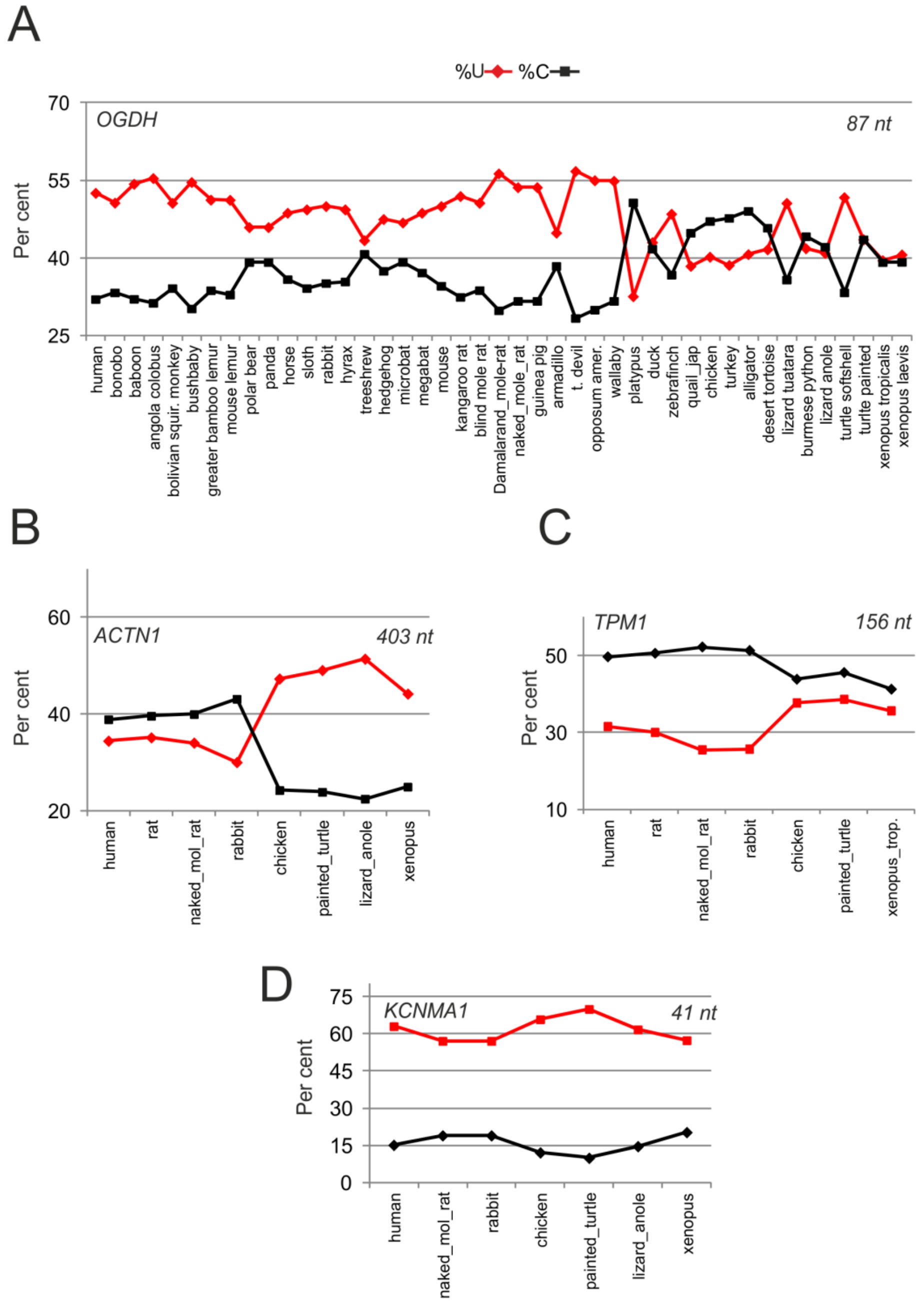

Figure S8 Gene- and exon-level expression of *OGDH* across human tissues

Gene-level expression is based on the GENCODE 19 (http://www.gencodegenes.org/releases/19.html) annotation collapsed to a single transcript model (*upper panel*). Exons associated with transcripts annotated as “retained_intron” and “read_through” were excluded. RPKM and TPM values were produced with RNA-SeQC v1.1.8; the filters were applied using the “-strictMode” flag in RNA-SeQC. Reads overlapping introns were not counted and the TPM values were not corrected for covariates**.** *Exon-level* expression is quantified for exon read counts; if a read overlapped multiple exons, a fractional value equal to the portion of the read contained within that exon was allotted. *Transcript-level* expression (*lower panels*) were calculated using RSEM v1.2.22. Data were compiled from ref. (23).

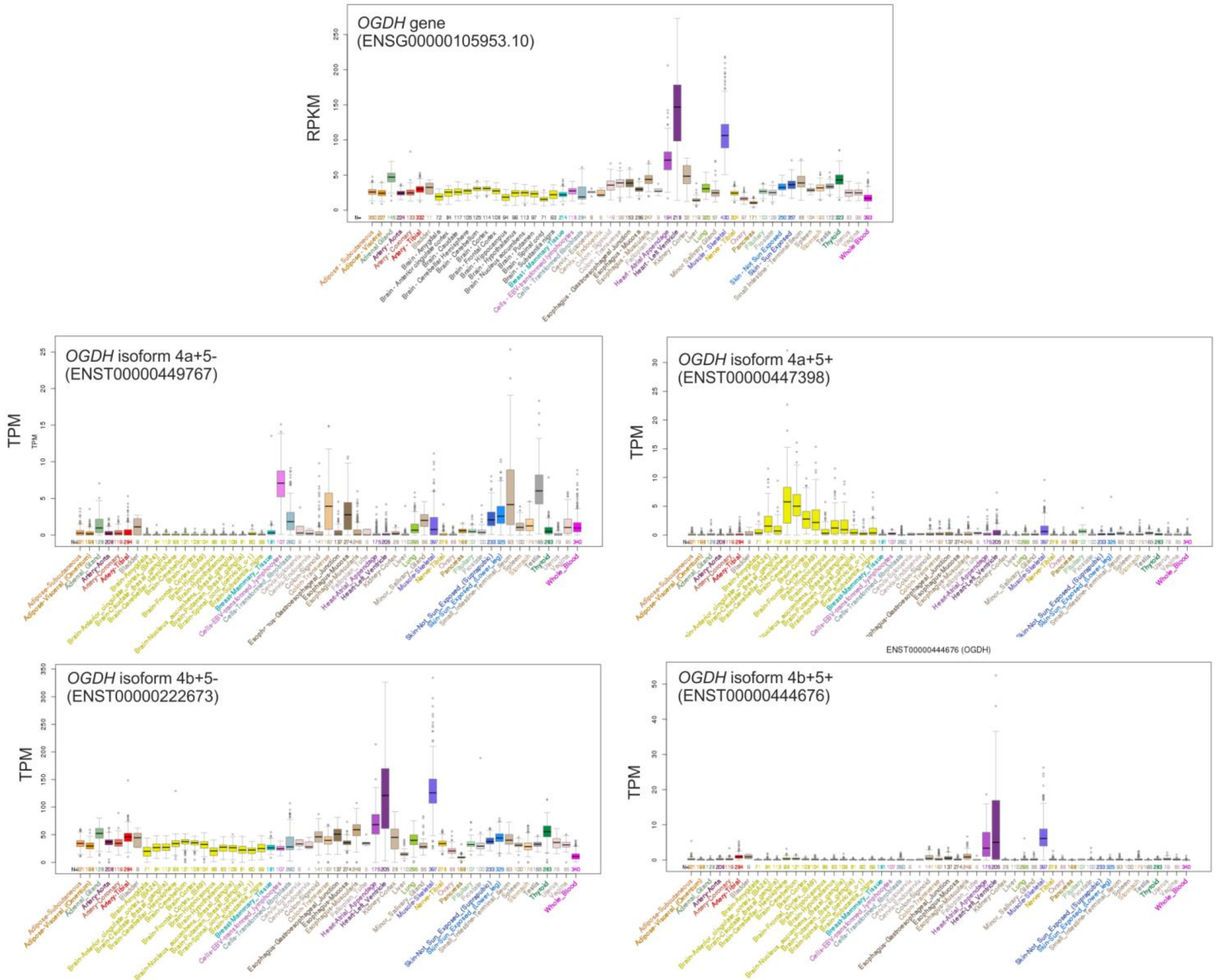

Figure S9 Endogenous expression of E1 transcripts during vertebrate evolution

A, *OGDH* (*upper panel*) and *OGDHL* (*lower panel*) mRNAs in human tissues. **B-F,** Comparison of endogenous *Ogdh* expression in striated muscles, brain and viscera in the indicated vertebrate classes. Restriction sites and the size of digestion products are schematically shown for each species at the top. **B,** human; **C,** rat; **D,** quail; **E,** frog; **F,** opossum, echidna and platypus. Asterisks denote heteroduplexes. The alignment of *Ogdhl* and *Ogdh* in *X. laevis* is in Figure S11. **G,** Inclusion of *OGDH* exon 5 is regulated by U-binding proteins that control MXEs.

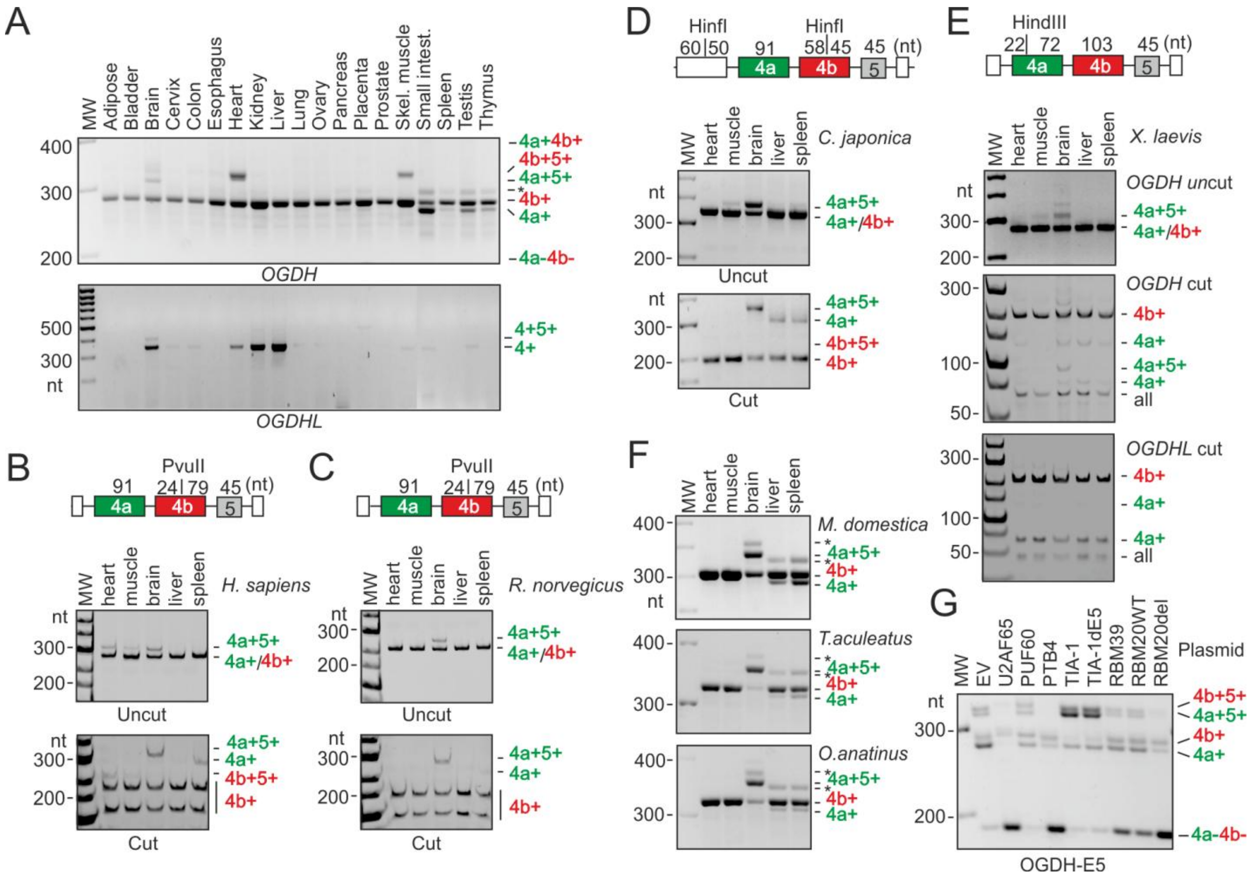

Figure S10 ***Ogdh* isoforms in the mouse brain**

Exon usage was analyzed by next-generation sequencing of RNA samples extracted from mouse brain cell populations (24) (http://jiaqianwulab.org/braincell/RNASeq.html). MO, myelinating oligodendrocytes; NFO, newly formed oligodendrocytes; OPC, oligodendrocyte precursor cells. Exons are numbered at the bottom.

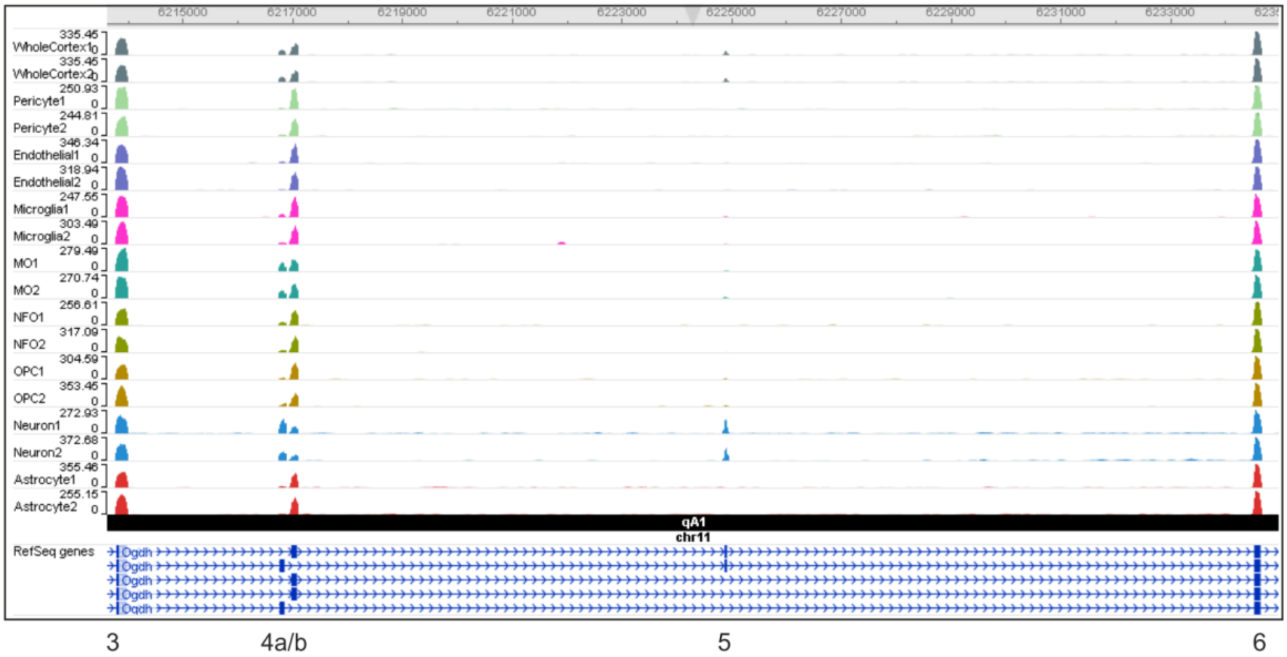

Figure S11 Alignment of *Xenopus laevis Ogdh* and *Ogdhl* intron 4a

Exons 4a and 4b are in green and red. HindIII sites are double underlined.

```
Ogdh        tgcttccagGTGAGGGGTCATCACATTGCAAAGCTTGACCCGCTCGGAATTAGTTCTGTT
Ogdhl       tgcttccagGTGAGGGGTCATCACATTGCAAAGCTTGACCCGCTGGGAATTAGTTCTGTT
            ******************************************** ***************

Ogdh        AATTTTGATGGGGCTCCTGTGATAGTCGGATCTCCAAATATGGgtgagaatcgttgtgtt
Ogdhl       AATTTTGATGGGGCTCCTGTGATAGTCGGATCTCCAAATGTTGgtgagaatccttgtgtt
            *************************************** * ********** *******

Ogdh        agacttgttgtaacgttctcttttctgtgctttttc----ctcctcctgttgtct-----
Ogdhl       agacctgttgtaactttttttcttttctgtgctttttttcctcctcctgttacctgtctt
            **** ********* ** * * ** * **   ***     ***********  **     

Ogdh        -ttgtgcccctttacctcttcttcctct---------------------------ttcct
Ogdhl       tgtgcccccctttaccacttcatccaatcgcccccttctgtgctttgccctccctttctt
              **  ********** **** ***  *                           *** *

Ogdh        tcctttgtgctacttctcc-cacctgcacctccaacctgccgccatctgactgctacg--
Ogdhl       tcctttgtaccacttctcttccccttcccctccaacctgctgccatctgtatgttactgc
            ******** * *******  * *** * ************ ********  ** ***   

Ogdh        ------------------------cctcctccatgcatttctcttctcttcctactgttc
Ogdhl       accttctcctccatgctttactgcacctgctcccaattctctctcctcgtcctactgttc
                                     *     *     * ***** *** ***********

Ogdh        tacttcaacccctcccatgtcctgtccttggaaccttcaatttctatagATCCGTGGGCA
Ogdhl       ttctgcaacccctcccttgtcctgtccttggatccctgaattcctatagATCCGTGGGCA
            * ** *********** *************** ** * **** *****************

Ogdh        CCATGTTGCTCAGCTTGACCCACTTGGCATTCTGGATGCAGATCTGGACTCGTGCGTGCC
Ogdhl       CCATGTTGCTCAACTTGATCCACTTGGCATTCTGGATGCAGATCTGGACTCGTGCGTGCC
            ************ ***** *****************************************

Ogdh        AGCAGATATTGTCACGTCCTCAGACAAACTCGgtgagggct
Ogdhl       AGCAGATATTATCACTTCCTCAGACAAACTCGgtgagggct
            ********** **** *************************
```

Figure S12 BP mapping in *Xenopus laevis Ogdh* intron 4a

A, Alignment of 10 clones with lariat junctions. dBP adenines are highlighted in green. **B,** Mutation of the dominant exon 4b dBP (dBP+30) eliminates exon 4b usage. **C,** Sequencing chromatograms of informative clones. Vertical arrowheads denote the 5’ss of intron 4a. Circles represent lariat junctions and horizontal bars denote the 5’ end of the first minigene intron. The mutation pattern is consistent with a typical variability across lariat junctions introduced largely by RT (25-27).

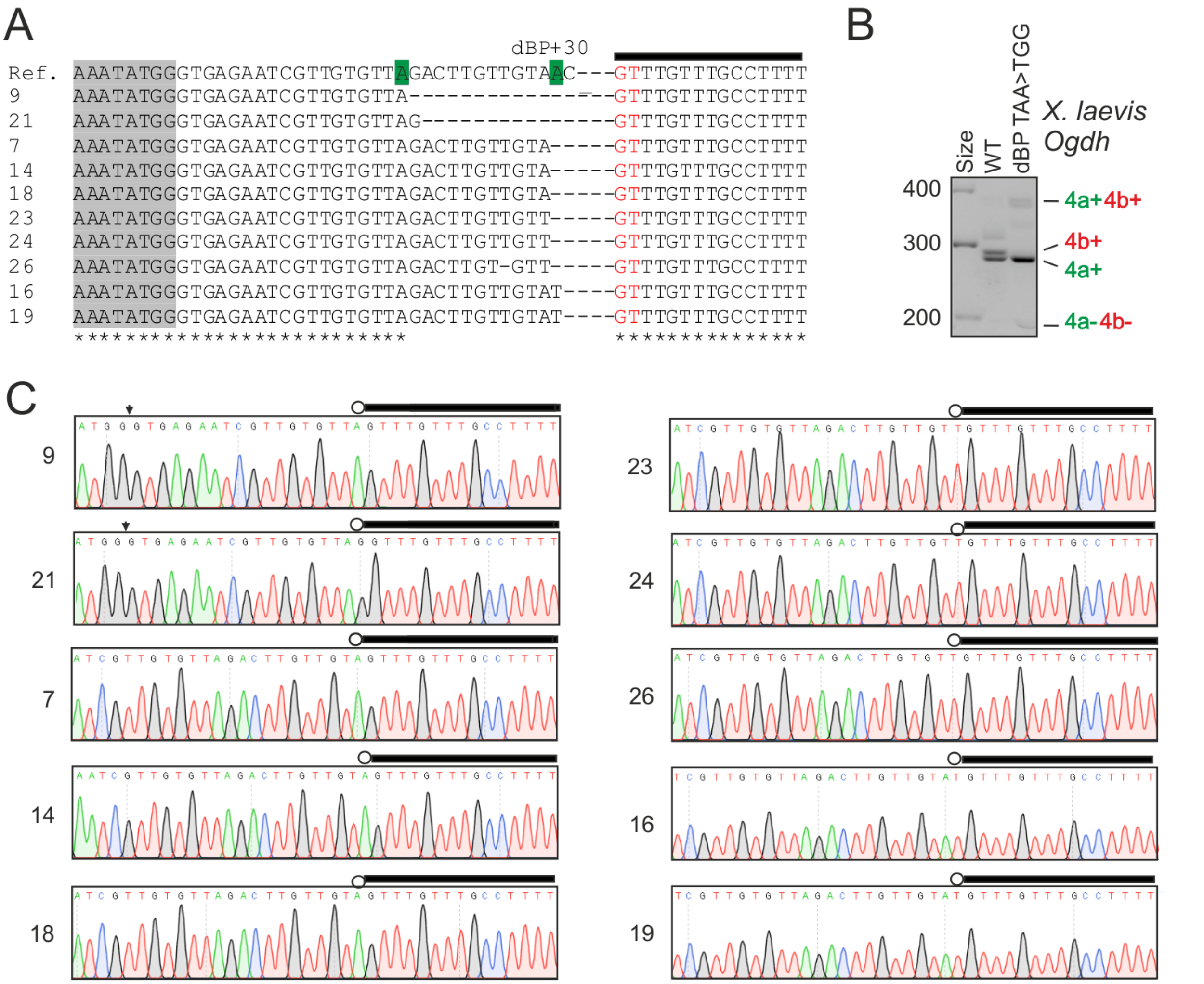

Figure S13 Tracing the origin of *OGDH* dBPs and AGEZ

A, Mean (±SD) distances (in nts) between the 5'ss of intron 4a and adenine in the first URA motif downstream. Asterisk denotes a significant difference between tetrapod and fish. **B,** Bidirectional shortening of *OGHD* intron 4a *en route* to terrestrial life**.** Average delta values (intron size reduced by AGEZ, in nts) in the inset highlight the shift to a stricter 5'ss-dBP threshold in amniotes. Error bars are SDs. **C,** High positive correlation between vertebrate *Ogdh* intron 4a size and their AGEZ length (r=0.96, $P<10^{-12}$). The outlier is *M. mola*, the largest bony fish. The violation of *M. mola* intron 4a AGEZ by an AG dinucleotide (boxed) is shown to the right. Mutations that extend the AGEZ to its canonical size in the *M. mola* reporter (bottom) are tested in panel **E**. **D,** Splicing pattern of taxon-specific constructs in cells lacking two *OGDH* MXE regulators. *Ogdh* reporters derived from the indicated species and their mutated versions were transfected into HEK293 cells depleted of U-binding RBPs shown at the top. RNA products are shown to the right. MW, size markers; sc, control cells transfected with scrambled siRNAs (17). *M. mola* transcrips lacking both MXEs are indicated by a red arrow. Immunoblots are bottom right. **E,** Removal of the *M. mola* AGEZ spoiler by point mutations failed to correct exon skipping observed for the WT (*P*>0.1, ANOVA with Dunnett's t-tests). Error bars are SDs of duplicate transfections.

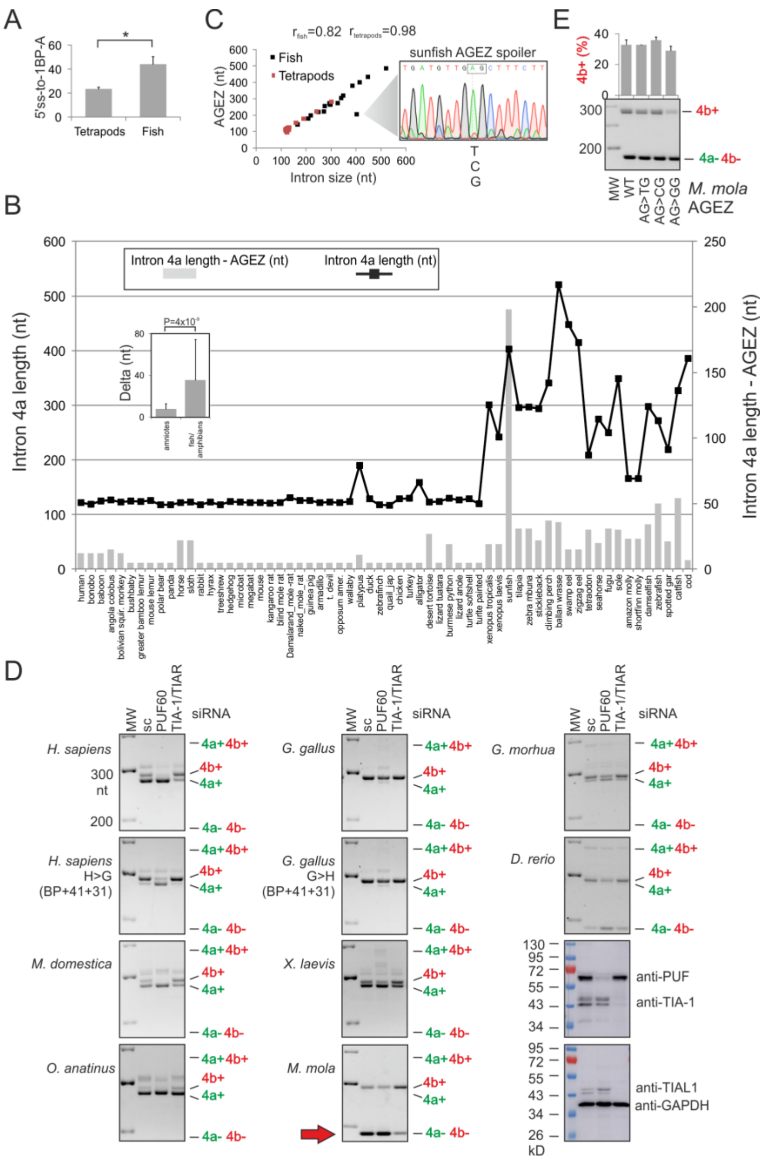

Figure S14 MXE genes preferentially function in calcium and sodium channel signalling

Enrichment of human MXE genes was analyzed using the overrepresentation method and molecular function categories of updated WebGestalt ((28); accessed on 28 October 2020), with 16,671 annotated IDs to functional categories as the reference, 550 unambiguously mapped EntrezID entries, and the entire human genome annotation as a background. Molecular function categories with the corrected false discovery rate (FDR) higher than 0.05 are not shown.

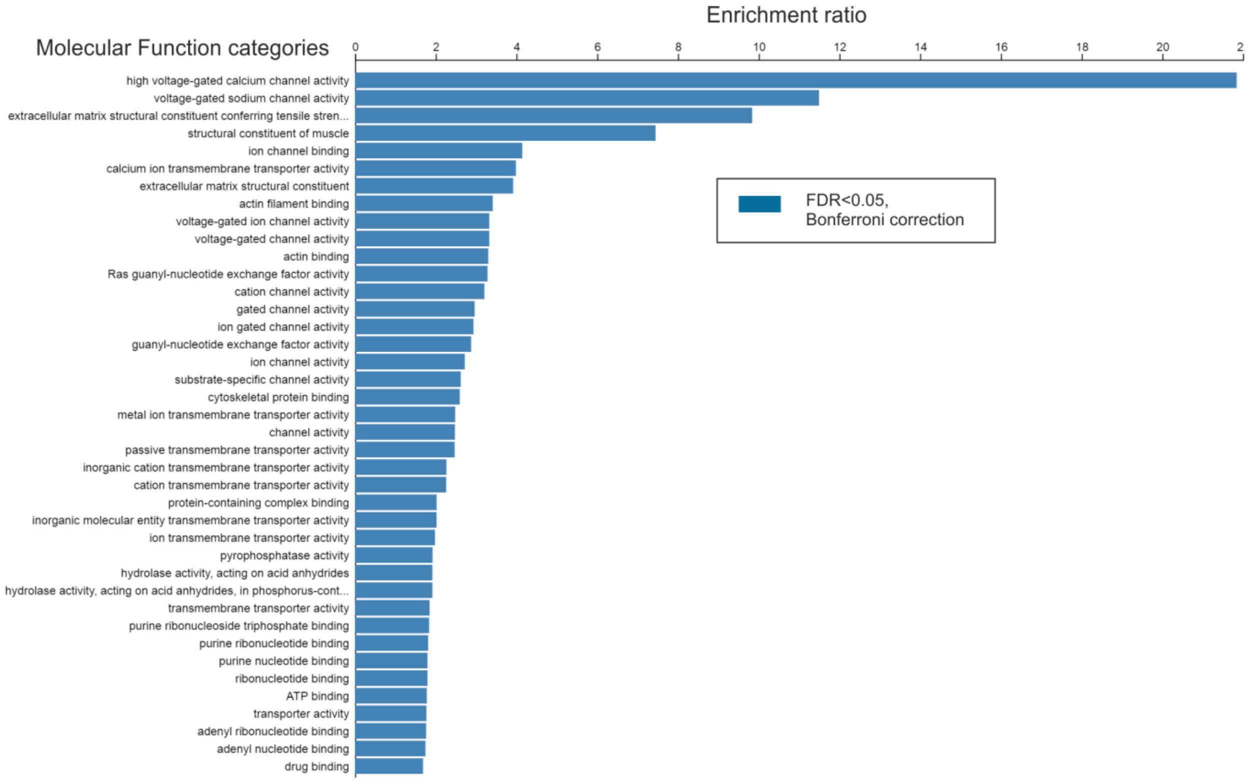

Figure S15 Ca^{2+}-induced OGDHC activation: endotherm/ectotherm split and *OGDH* intron 4a size

K_m values for 2OG in heart mitochondria were taken from reference (29). Y-axis shows K_m ratios at <1 nM and ~30 μM Ca^{2+}. Endotherms (human, rat and pigeon) are in red, ectotherms (frog and fish) are in blue.

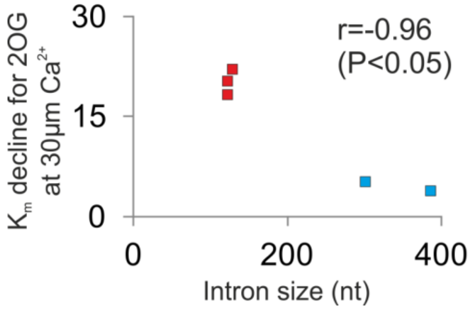

Figure S16 Additional examples of PUF60-regulated exons in genes involved in mitochondrial Ca^{2+} signalling/ATP synthesis

A, PUF60-depletion induced retention of the last *AK2* intron. This intron contains an alternative polyadenylation site (APA; vertical red triangle). The use of APA site would alter the availability of the ATP binding site at Q214 encoded by the last exon. **B,** Spacefill representation of the human AK2 structure. Arrow denotes the exposed residue that binds ATP. **C,** Downregulation of *AFG3L2* exons 1-4 in cells lacking PUF60. AFG3L2 mediates degradation of SMDT1/EMRE before its assembly with the mitochondrial Ca^{2+} uniporter (MCU) complex, limiting the availability of SMDT1/EMRE for MCU and promoting efficient assembly of gatekeeper subunits with MCU (30). Downregulation of *AFG3L2* exons 1-4 in cells lacking PUF60 would reduce the availability of AFG3L2 isoforms with the transit peptide (encoded by exon 1) and propeptide (encoded by exon 2). PUF60 depletion would therefore reduce not only the OGDH sensitivity to Ca^{2+} and but also canonical *AFG3L2* transcripts that encode proteins capable of reaching mitochondria. Mitochondria in cultured AFG3L2-deficient Purkynje cells are inefficient in buffering and shaping Ca^{2+} peaks; spinocerebellar ataxia due to *AFG3L2* mutations can be rescued by reduced Ca^{2+}_c concentrations (31). Neurons lacking AFG3L2 and the essential MCU subunit EMRE are also vulnerable to mitochondrial Ca^{2+} overload (30). Loss of MICU1 and mitochondrial Ca^{2+} overload specifically affects Purkynje cells and leads to ataxia (32). **D,** APA of *GLS* transcripts. Proximal APA site was promoted in cells lacking PUF60 while distal site was promoted in cells lacking U2AF65 (see also Figure 9). Red rectangles show differentially used exons. *GLS* is alternatively spliced and polyadenylated, giving rise to long (669 aa, KGA) and short (598 aa, GAC) isoforms ((33) and refs. therein). The GAC isoform is abundant in the heart whereas isoform KGA is expressed in the brain, mainly in neurons and less in astrocytes (33). The GAC isoform lacks ankyrin repeats and the KEN box and its dependence on inorganic phosphate (P_i) may not be identical to that of KGA (33). GLS catalyzes the hydrolytic deamidation of glutamine to glutamate and contributes to the production of the most

important neurotransmitter in the brain (33). Synaptic vesicles are, however, capable of synthesizing glutamate from 2OG (34,35), although the exclusive supply to glutamate through this pathway might deplete the TCA cycle of key intermediates (36).

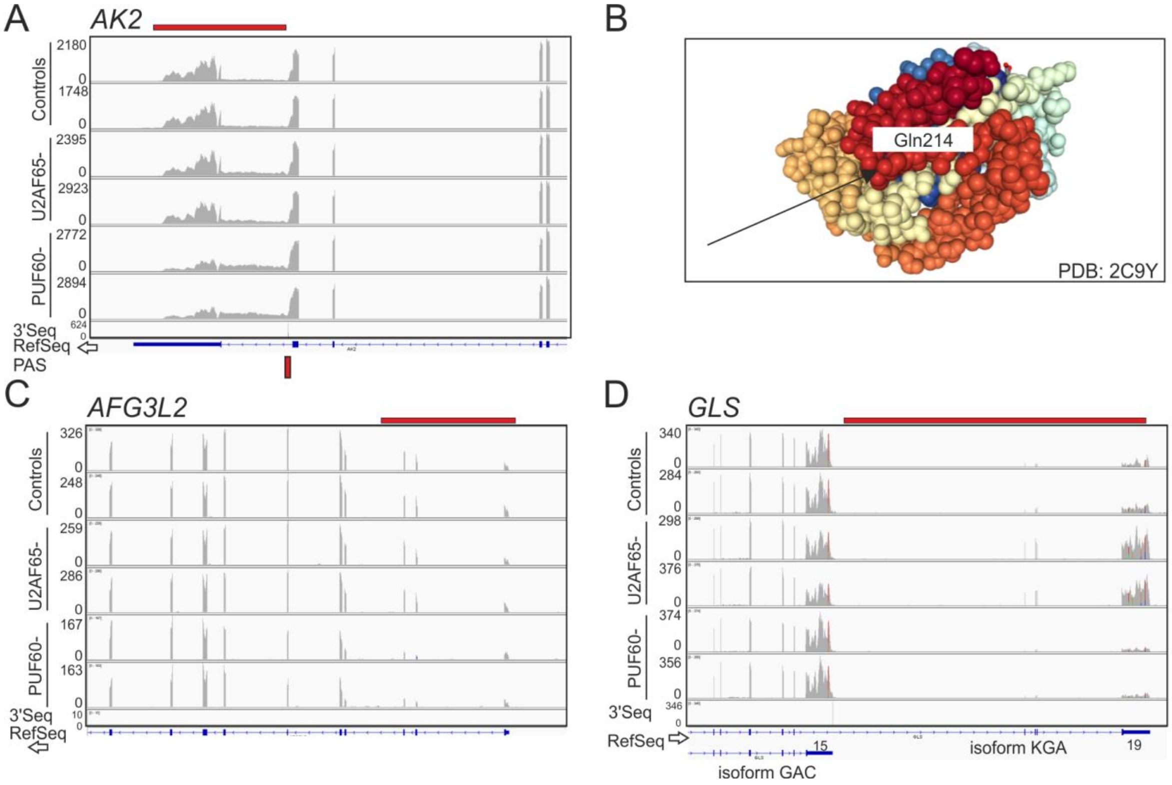

Figure S17 Evolution of the DADLD motif and site 2 in Ogdh

A, Partial alignment of prokaryotic and eukaryotic E1 proteins. Residues encoded by mammalian exon 4 variants are underlined; residues encoded by exon 6 are double-underlined. Putative Ca^{2+} -binding motif at site 2 (4) is highlighted in yellow. In some photosynthetic eukaryotes, such as green algae, site 2 contains the DADLD motif (in red). Full alignment of E1 proteins was carried out using Clustal-Omega (v. 1.2.4) with default options. Residues encoded by neuron-specific exon 5 are not shown. **B,** Alignment of peptides encoded by human *OGDH* exon 4a and 4b (highlighted in grey).

A

```
                                                            Site 2
Escherichia              NLDPLGLWQQ-D--------KVADLDPSFHDLTEADFQETFNVGSF------ASGKETMK  152
Pelagibacterium          NLDPLGLENR-E--------EAPELDPAAYGFSEADYTREIFIDNY-------LGLEFAT  182
Starkeya                 KLDPLGLEP-ER--------SAPELDPASYGFREADLDRPIFIDHV-------LGLEFAT  186
Chelativorans            DLDPLGLAKPME--------DYNELSPEAYGFTEADFDRPIFIDNV-------LGLETAT  184
Labrenzia                DLDPLQLATPG---------DHEELHPSSYGFTEADWDRSIFIDHV-------LGLEYAT  182
Medicago                 KLDPLNLEARQ---------IPDDLDPALYGFSEADLDREFFLGVWRMAGFLSENRPVQT  189
Glycine                  KLDPLNLEPRQ---------ISEDLDPALYGFTEADLDREFFLGVWRMAGFLSENRPVQT  195
Brachypodium             KLDPLGLEERP---------VPDVLDPAFYGFSEDDLDREFFLGVWKMAGFLSENRPVQT  181
Zea                      KLDPLGLEERP---------VPDVLDPGFYGFSEADLDREFFLGVWMMAGFLSENRPVQT  190
Oryza                    KLDPLALEERP---------IPDVLDPAFYGFSEADLDREFFLGVWRMAGFLSENRPVQT  181
Arabidopsis              KLDPLGLEKRE---------IPEDLTPGLYGFTEADLDREFFLGVWRMSGFLSENRPVQT  190
Solanum                  KLDPLDLEERD---------IPDVLDPVSYGFTEADLDREFFLGVWRMAGFLSENRPVQT  187
Vitis  (grapevines)      KLDPLGLEERE---------IPDDLDPALYGFTEADLDREFFLGVWRMAGFLSENRPVQT  190
Prunus                   KLDPLGLEERE---------IPDDLDPALYGFTEADLDREFFLGVWRMAGFLSENRPVQT  187
Ricinus (castoroilplant) KLDPLGLEERE---------IPEDLDPALYGFAEADLDREFFLGVWRMSGFLSENRPVQT  187
Populus (poplar)         KLDPLGLEERE---------IPDDLDPALYGFTDADLDREFFLGVWRMAGFLSENRPVQT  187
Volvox                   DLDPLGISGHA---------HPPELDPSFWGFKETDLDREFYIGNWNQAGFLAEGRPMRT  195
Chlamydomonas            DLDPLRISGHT---------HPPELDPAFWGFKDTDLDREFFVGNWNQSGFLAEGRPTRT  191
Ostreococcus             RLDPLGLDKRE---------GIIYLEPALYGFSEDDMDREFFIGTWKMQGFLSEDRPVQS  242
Micromonas (green algae) KLDPLGLDVRD---------VPVELDPALYGFTDADLDREFFLGSWRMKGFLSEDNPVQT  231
Schizosaccharomyces      KLDPLGINVNH-----N---RPSELTLEHYGFTESDLNRTIHLGPGILPNFREAGRKTMT  195
Aspergillus              KIDPLGIRGEAEAFGYS---KPKELELDHYGFTERDLDEEFDLGPGILPRFATEGRKKMS  223
Saccharomyces            HIDPLGISFGSN-KNNP---VPPELTLDYYGFSKHDLDKEINLGPGILPRFARDGKSKMS  196
Candida                  KIDPLGISFGDN-T--T---VPKELTLDYYGFTEQDLAKEITLGPGILPRFAQGGKKSMT  185
Drosophila               HLDPLEINTPELPGNSS-----TKSIYANFSFGEQDMDRQFKLPST---TFIGGDEASLP  197
Felis (isoform 4b)       QLDPLGILDADLDSFVPSDLITTIDKLAFYDLREADLDKEFQLPTT---TFIGGSEHTLS  185
Mus (isoform 4b)         QLDPLGILDADLDSSVPADIISSTDKLGFYGLHESDLDKVFHLPTT---TFIGGQEPALP  202
Homo (isoform 4a)        KLDPLGISCVNFDDA----PVTVSSNVGFYGLDESDLDKVFHLPTT---TFIGGQESALP  198
                          :*** :                     .: . *  . : :
```

B

```
4a      RAYQVRGHHIAKLDPLGISCVNFDDA----PVTVSSNVGFYGL
4b      RAYQIRGHHVAQLDPLGILDADLDSSVPADIISSTDKLGFYGL
        ****:****:*:******  .::*.:     :: :.::*****
```

Figure S18 Exon enhancing and silencing activity of 64 codons: dichotomy of codons involved in Ca^{2+}/Mg^{2+} binding versus Cu^{2+}/Zn^{2+} binding

A,B, Codon frequencies in high-confidence ESEs **(A)** and ESSs **(B)**. The ESE and ESS hexamers were reported previously (37). Residues preferentially involved in binding weak Ca^{2+} and Mg^{2+} are boxed in green; residues preferentially involved in binding of competitive Cu^{2+} and Zn^{2+} are boxed in red. Asterisks denote stop codons. The UAG codon has the highest silencer activity and is completely absent in the comprehensive set of ESE hexamers (37). **C,D,** Average splicing activities for codons involved in metal binding sites for Mg^{2+}, Ca^{2+} and Mn^{2+} **(C)** and Fe^{2+}, Cu^{2+} and Zn^{2+} **(D)**. Amino acids are shown at the bottom; their frequencies in metal binding sites were compiled using estimates based on fragment transformation methods (38).

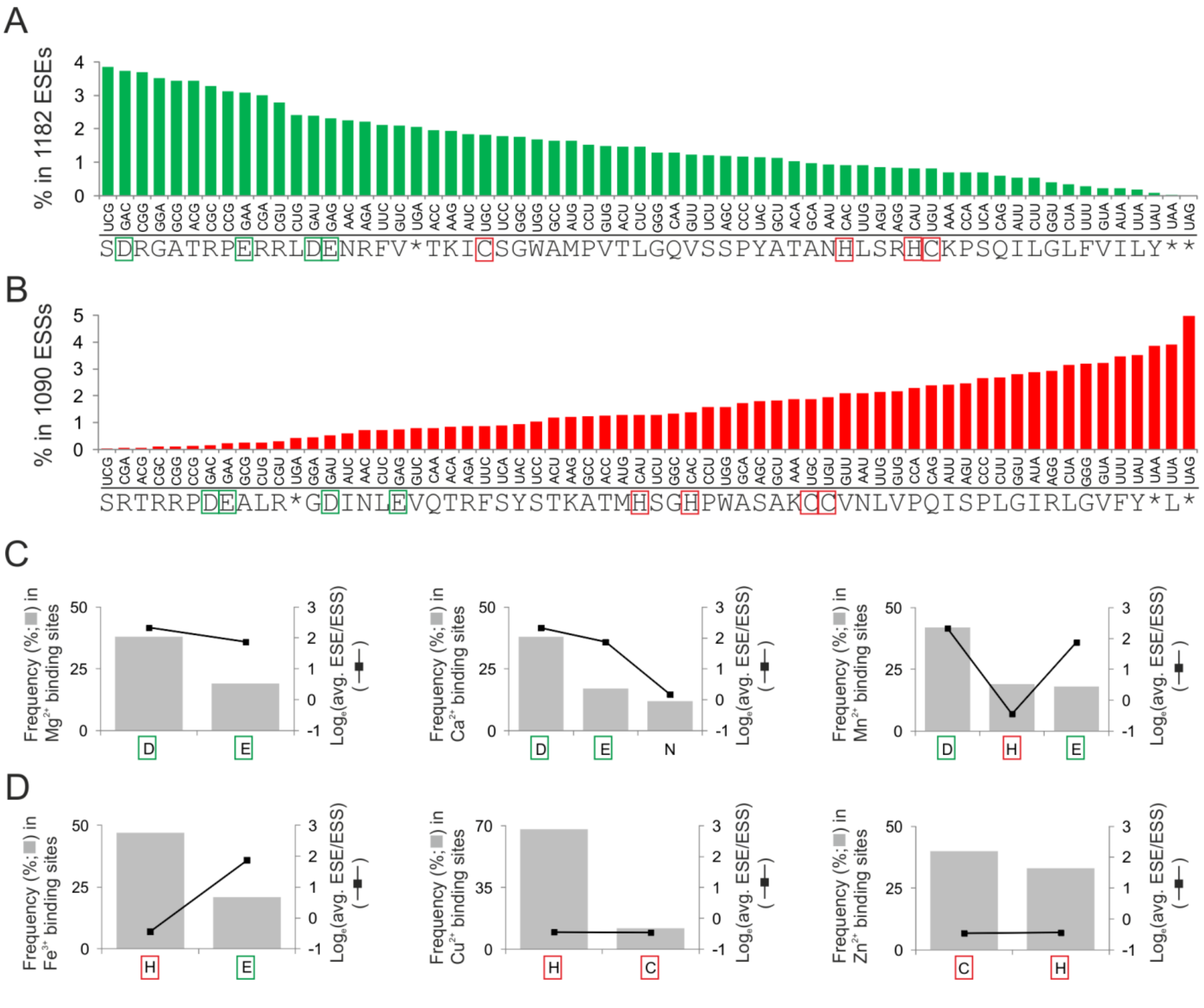

Figure S19 Hexamer preference indices (HPIs) of ESE sets and residues most frequent in metal binding sites

Designation of ESE sets is as described by Cáceres et al. (39), including RESCUE ESE (40,41), PESE (42), a mixture of ESE and ESS (ESR; (43)), Ke-ESE (37) or their subset (Ke_ESE), or various intersects (INT sets). **A**, Codons for key residues in calcium/magnesium binding sites. **B**, Codons for key residues in copper/zinc protein binding sites. **C**, The Irving-Williams order (top) and HPI (39) gradient for the ESE sets shown at the bottom. Weighted average HPIs were normalized for amino acid frequencies in protein binding sites for the indicated metals, employing only residues within 3.5Å of the metal ion centre (38).

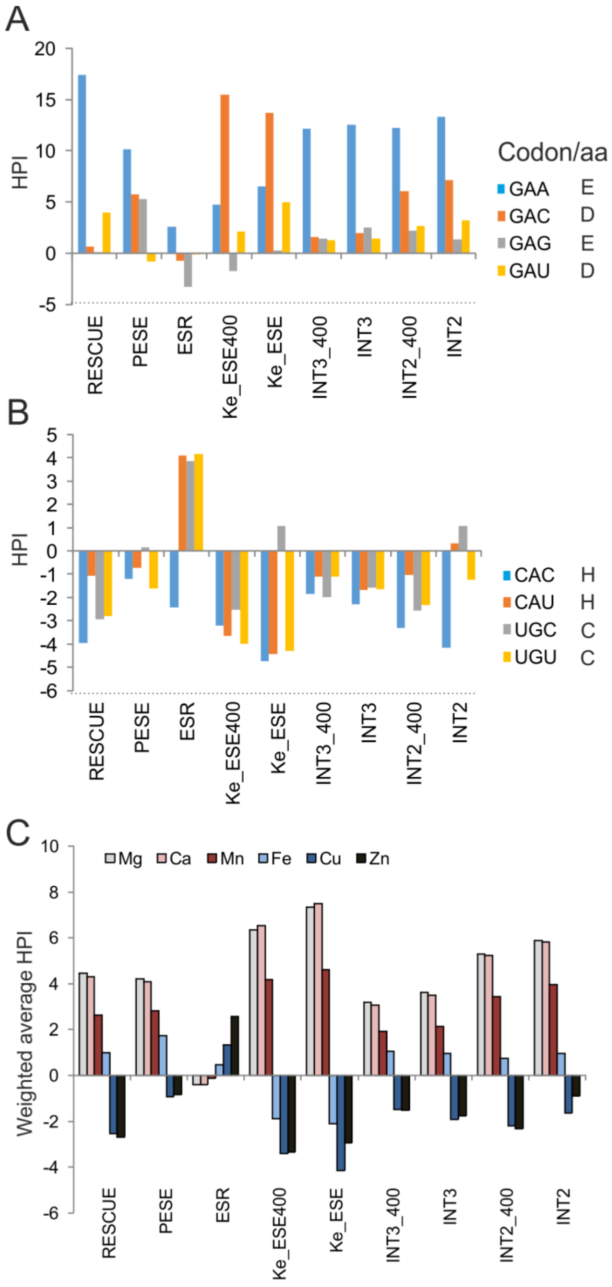

SUPPLEMENTAL DISCUSSION

Evolution of endothermy and *Ogdh* MXEs: a hypothesis

Discussion S1 *Ogdh* MXEs and the energy supply face of becoming warmblooded

Ca^{2+}_m levels are important regulators of the TCA cycle. They can exceed 10 µM during cell activation, stimulating matrix DHs, increasing the cycle flux and ATP production and the release of ATP to the cytosol. The increase of ATP supply depends on the amplitude of Ca^{2+}_m rise and the availability of oxidative substrates ((44,45) and refs. therein). OGDHC plays a key role in Ca^{2+}-activated delivery of NADH and ATP, limiting reducing equivalents, electron transport and ATP synthesis (46-50). OGDH activities provide good estimates of the maximum flux through the TCA cycle in vivo (51) and may define the maximal respiratory capacity of mitochondria (48,49,52). OGDHC may have a greater effect on the respiration rate than other TCA enzymes. For example, OGDH and isocitrate DH were estimated to control 70% and 23%, respectively, of the respiratory flux upon Ca^{2+} stimulation (53). Moreover, catalytic OGDH reactions are accompanied by a large change in free energy and, among Ca^{2+}-sensitive matrix enzymes, OGDH has the smallest K_m values in the presence of ADP ((44,54) and refs. therein). OGDHC deficiency or inhibition leads to a significant reduction in respiration rate that may be more dramatic than inhibition of mitochondrial isoforms of other TCA enzymes, including aconitase (*ACO1*), malate DH, fumarase, citrate synthase and the NAD+-dependent isocitrate DH (50,52). Transgenic studies in plants showed that the TCA cycle control points were distributed among malate DH, OGDH (flux control efficient 0.79), ACO1, fumarase, and succinate DH (52). Besides ACO1, systematic inactivation of TCA cycle genes in yeast coupled with transcript analysis revealed altered expression of the largest number of genes for *Ogdh* mutants (55). The expression of aerobic genes, predominantly of the mitochondrial respiratory complexes, was diminished in *Ogdh*-mutated strains and to a lesser extent in *ACO1* mutants, but not in other mutants of TCA cycle enzymes, while the expression of anaerobic and hypoxic genes was elevated (55).

Simulation studies using a human heart model of mitochondrial metabolism suggested that the effect of OGDHC inactivation on the energy status is manifested only under conditions of increased energy demand, and not at rest (56). Mice lacking the mitochondrial Ca^{2+} uniporter (MCU) cannot perform strenuous work as a result of diminished Ca^{2+} uptake in skeletal muscle mitochondria (57). Addition of Ca^{2+} over the physiological range increased mitochondrial respiration in the wild-type mice, but no Ca^{2+}-dependent stimulation was observed in homozygous mutant mice while the basal metabolism seemed unaffected, although Ca^{2+}_m was not completely absent (57). The large change in ATP requirements during striated muscle activity renders Ca^{2+} transfer to mitochondria a key signalling step (58). At rest, a low MCU activity was enough to sustain basal ATP levels and heart rate, but under stress conditions the MCU-dependent Ca^{2+}_m uptake and ATP generation became crucial (59).

Activation of Ogdhc by Ca^{2+} can be, to a large extent, explained by the DADLD motif encoded by exon 4b (4,60). This motif as well as closely linked site 2 probably evolved on multiple occassions during evolution (Figure S17A). Expression of this motif has been promoted by the capacity of codons encoding critical residues in Ca^{2+}-binding sites to enhance exon inclusion levels in mature transcripts (Figure 10, S18-S19), by a loss of the enhancing codon in competing exon 4a during evolution (Figure 5) and by the unusual arrangement of the 3’ss, with dBPs located near the opposite intron end (Figure 1-4). The DADLD motif evolved in ectotherms (4), but ectotherms have longer intron 4a (Figure 1) and their splicing pattern is not fully develolped (Figure S6). Non-redundant dBPs in anamniotes evolved into redundant and more robust dBP clusters, followed by enrichment of megaPPTs for uridines that bind abundant splicing factors (Figure 1,2). This intron organization was completely immune to enlargements, protecting it from transposon expansions for hundreds of Myrs (Figures 1-4), in line with the importance of the MXE regulation in cell energy supply.

Taken together, *Ogdh* MXEs provide a critical switch for Ca^{2+}-dependent NADH and ATP supply. Fine-tuning of their alternative splicing may result in a strong selective advantage at the cellular level, as well as at the level of the organism and the species. Optimized regulation of their tissue-specific

inclusion in mRNAs at multiple levels should ensure responsive and sustained activity of striated muscles, major contributors to thermogenesis (Discussion S2), as well as maximum locomotory endurance during vertebrate evolution, a central tenet of the aerobic scope model of endothermy.

Discussion S2 OGDHC in Ca^{2+}-dependent muscle thermogenesis

Cell motility was intrinsically coupled to evolution of muscles (61), which evolved muscle-specific alternative splicing pathways in vertebrates (62,63) (Table S6,S7). Apart from locomotory function, striated muscles have played a key role in both activity-dependent and -independent heat production and have the largest potential to generate body heat ((64) and refs. therein). Although they may not generate most body heat at rest in some species, increased muscle activity can produce ~40x as much heat as the rest of the body, including visceral organs. For example, the contractile apparatus and ion pumps together account for ~75% of ATP consumption by cardiac myocytes ((65) and refs. therein).

Striated muscles have also the highest densities of organelles that generate most of the heat. Mitochondrial as well as respiratory chain complex densities are much higher in striated muscles than in the brain or viscera and muscle mitochondria have a very distinct morphology, including cristae organization, matrix density and subcellular distribution (66). In endotherms, mitochondria physiologically operate at temperatures as high as ~50 ºC, as measured by a fluorescent dye that accumulates in active organelles and may bind matrix components (67). The high temperature fell upon addition of respiratory inhibitors and rose upon pyruvate addition, although Ca^{2+}_m levels were not measured (67). Human respiratory enzymes also operate at higher temperatures and their thermofilic counterparts and matrix DHs can withstand even hotter temperatures without loss of activity (67-69). Thus, muscle mitochondria have been major heat suppliers to the cells.

Skeletal muscles of many partial or regional endotherms evolved into ‘heater organs’ which employ energetically costly Ca^{2+} cycling to/from sarcoplasmic reticula (70). One of the best studied heater organs are slow-twitch muscles of some fish predators, such as tunas or billfishes (70). These organs have high oxidation rates and aerobic capacity and are densely packed with sarcoplasmic reticula and mitochondria. The two organelles are intimately juxtaposed in the cell (58). The heater organs lack myofibrillar structures typical of force-producing (slow-twitc) muscles, reflecting high NADH/ATP demand and selection for rapid Ca^{2+} release and reuptake (70). The slow-twitch muscles have on average higher stamina, smaller glucogen stores and lower glycolytic activity, higher oxidative phosphorylation and reliance on oxygen, smaller fibre diameter, higher capillary density, slower build up of lactate, and a higher myoglobin concentration and oxygen storage capacity than their fast-twitch counterparts ((64,71) and refs. therein). A prominent example of a largely slow-twitch muscle is soleus, which can contain up to 100% of slow myofibres, is critical for walking and running, evolved from a common limb flexor present already in early tetrapods, including walking fish *Ambystoma mexicanum,* and may have originated from fish pelvic appendages (72). In lizards, stance-phase muscles such as gastrocnemius or soleus are better predictors of maximum performance than swing phase muscles (73). Disuse of skeletal muscles in non-hibernating mammals leads to slow-to-fast fibre type transitions (74). In contrast, muscles in hibernators (Discussion S3) undergo fast-to-slow transitions (74) and are capable of a remarkable preservation of skeletal muscle mass, which could contribute to their resistance to atrophy ((75) and refs. therein). Slow-twich muscle fibre formation is driven by PGC-1α (76). PGC1 proteins are master metabolic regulators that may also function as RBPs since they contain a C-terminal RMM and a serine/arginine-rich domain along with N-terminal transcriptional activation domain (77). Thus, under the aerobic scope model of endothermy, slow-twitch muscles are prime candidates for selection processes acting on MMR.

Contraction-based (‘shivering’) thermogenesis is an ancient mechanism for generating heat, well documented in invertebrates (78) and long predating the *Ogdh* exon duplication. With a significant increase of skeletal muscle mass in vertebrates, striated muscles have become the primary thermogenic organ (79). At some point in evolution, however, futile Ca^{2+} cycling through sarcoplasmic reticulum Ca^{2+} ATPases (SERCAs) and ryanodine receptor channels became a key mechanism for a dominant form of heat production during prolonged cold adaptation, replacing shivering thermogenesis with a ‘non-

shivering' counterpart (64). This process had to rely on a Ca^{2+}-dependent ATP supply by mitochondria to match the demand of ATPase activities, particularly SERCAs, which are more important for establishing non-shivering thermogenesis than myosine ATPase or Na+/K+ ATPase (79). SERCA2 (ATP2A2) is differentially expressed in oxidative and glycolytic chicken myofibers (80). *D. rerio* with high levels of sustained locomotor performance had elevated levels of SERCA1, RYR and PGC-1 mRNAs than the fish with low sustained swimming speeds (81). The importance of Ca^{2+}-mediated pathways in both resting and activity-induced heat production is probably best illustrated by a high fraction of the muscle energetic turnover attributable to Ca^{2+} cycling in multiple species and close parallels between 'tuna burn' and malignant hyperthermia in mammals ((70) and refs. therein). Malignant hyperthermia is caused by mutations in *RYR1*, ryanodine receptor tightly regulated by ATP binding in a Ca^{2+}-dependent manner (82,83). The significance of futile cycles in T_b control is highlighted by a loss of *Drosophila THADA* (THyroid ADenoma Associated human homologue), one of the most strongly selected gene. The *THADA* knockout leads to elevated activity of SERCA, diminished heat production and cold sensitivity (84). THADA binds SERCA and uncouples its ATP hydrolysis from Ca^{2+} pumping (84).

Many defects of Ca^{2+}_m pathways and ATP synthesis generate phenotypes primarily involving striated muscles. For example, MCU silencing in limb muscles leads to muscle atrophy, suggesting that the MCU is required for muscle size control (85). Loss-of-function mutations in the *MICU1* gene (mitochondrial calcium uptake 1) cause proximal myopathy and extrapyrmidal movement disorders (86). Mutations in the *OGDH* MXE regulator TIA-1 (Figure 1F) were associated with myopathy (87,88) and may affect steady-state TIA-1 expression or protein properties other than RNA binding (Figure 2D).

Muscular activity in endotherms, but not in vertebrates with cartilaginous skeletons, leads to abrupt hypercalcaemia (89). During maximal contraction, ATP consumption in skeletal muscles can increase over 100-fold (90). Muscle ATP stores are insufficient to meet this demand, necessitating a rapid and sustained supply of new molecules. Mitochondrial Ca^{2+} uptake is fast enough to support step changes in muscle workload: in isolated heart mitochondria, the uptake occurs within a 100 ms time-resolution limit, leading to activation of NADH production and Ca^{2+}-dependent DHs within 200 ms and oxidative phosphorylation within 270 ms, although the off-kinetics is much slower (91). Beat-to-beat changes in Ca^{2+}_m in cardiomyocytes can translate time-dependently into steady-state alterations in ATP (92,93). Even a single muscle twitch could be associated with measurable changes in Ca^{2+}_m in living motor fibers milliseconds later (94). Although electrical conduction via the mitochondrial reticulum as opposed to ATP or oxygen diffusion is a more effective way to quickly and uniformly distribute energy in muscle cells, metabolite-facilitated diffusion pathways become significant during maximum endurance (95). Mitochondrial ATP production is kinetically more responsive to changes in Ca^{2+} than ADP or P_i (91). Elevation of Ca^{2+}_m stimulates oxidative phosphorylation more strongly than substrate oxidation to achieve homeostasis of mitochondrial membrane potential (96). Although the exon-level regulation of OGDH activation by RBPs (Figure 1) is slower than the time-scale of Ca^{2+} transients, the drop in ATP synthesis was considerably delayed after the transient Ca^{2+}_m signal had returned to basal levels (45).

The MXE organization of *OGDH* exons 4a/4b resembles that found in other muscle-related genes, including tropomyosin (*TPM1*) (21), α-actinin (*ACTN1*) (22,97), troponin T (*TNNT*) (98) and myosin light chains (*MYL1* and *MYL3*) (99). Actinin MXEs that encode isoforms with differential Ca^{2+} sensitivity employ dBPs of the smooth muscle-specific exon (22). *ACTN3* is only expressed in glycolytic myofibers (100) and its null allele has been associated with increased aerobic capacity and endurance and higher citric synthase activities ((101,102) and refs. therein). The null allele leads to increased SERCA1 expression and Ca^{2+} leak from the sarcoplasmic reticulum (101,102). The futile cycle involving an increased reuptake of Ca^{2+} would generate heat by non-shivering muscle thermogenesis and provide an explanation for the evolutionary advantage of carrying the null allele in cold climates (101,102). Actinin and tropomyosin are also important in slow-to-fast muscle fibre transitions in hypometabolic states ((103) and refs. therein; Discussion S3).

To what extent were tissues other than skeletal muscles important for the acquisition of endothermy? Although cardiovascular characteristics of animals have been studied extensively, improved delivery of oxygenated blood is unlikely to be selected for in the absence of efficient mechanisms that can

utilize oxygen as the ultimate electron acceptor and produce ATP on demand. Rather, a greater capacity for responsive ATP production would favour improved oxygen delivery to critical tissues (striated muscles and CNS). Rapid oxygen delivery would be facilitated by the emergence of four-chamber hearts in mammals, birds and crocodilians, through-flow lungs in birds and theropods (104), blood sinuses on the top of the head of earless lizards rapidly warming the whole body, and superior capillary systems allowing efficient oxygen transport. E1 is most expressed in the left heart ventricle (Figure S8). OGDHC generates a substrate (succinyl-CoA) for synthesis of heme, a prosthetic group of hemeproteins involved in electron and oxygen transport, such as cytochrom c oxidase and hemo-/myo-globins. In yeast *Ogdh* mutants, expression of hypoxic/anaerobic genes was elevated while expression of oxidative genes was diminished, consistent with a heme signalling defect caused by inadequate levels of succinyl-CoA, the heme precursor (55). Ca^{2+} can induce a spectral shift in heme *a* and change in the midpoint redox potential (105,106). OGDHC is sensitive to oxidative stress and occupies a central position at the cross-roads of redox pathways ((107) and refs. therein). OGDHC is important for regulation of hypoxia-inducible factor HIF1α in aerobic conditions, a master regulator of genes involved in essential hypoxic responses that maintain cellular ATP levels (108). In addition, OGDHC is the main source of reactive oxygen species (ROS), which are strongly stimulated by Ca^{2+}, beating other TCA cycle enzymes, including pyruvate DH, both in cardiac and visceral tissues (109-111). OGDHC dominates ROS production regardless of whether pyruvate or succinate serves as the sole source of carbon (110,111).

Brown adipose tissue (BAT) is believed to have unique thermogenic abilities that are derived from properties of the uncoupling protein UCP1 ((112) and refs. therein) (see also Discussion S6). However, BAT has not evolved until the appearance of placentals and cannot explain more ancient examples of regional endothermy (79). Neither fish nor large mammals have BAT (64). Importantly, skeletal muscles and BAT have a common ancestor: for example, mitochondrial proteomic signatures show more similarities between muscles and BAT than between BAT and white adipose tissue ((79) and refs. therein).

The increase in the Ca^{2+} cycling across the mitochondrial membrane generates proton leak and heat. The contribution of proton leak to oxygen consumption in resting skeletal muscles was reported as high as 60% in vitro (113,114). However, this figure was estimated to be very low or close to zero in other systems or in vivo (115,116) or during intense muscle activity (117). Ca^{2+}-induced increase in state 4 respiration resulted from elevated protonmotive force and not from direct activation of proton leak (115).

Collectively, these studies show that responsive and Ca^{2+}-dependent ATP supply to striated muscles has been crucial for internal heat generation during evolution. OGDH role in Ca^{2+}-dependent NADH supply, coregulation of this reaction with and distant steps of ATP synthesis pathways (Figure 9B-D, S16), as well as the OGDHC role in glutamate (Discussion S4), heme, redox and fatty acid metabolisms make E1 a strong candidate for playing a potentially major role in shaping the aerobic scope and evolution of endothermy.

Discussion S3 OGDHC and Ca^{2+} in hypometabolic states

In torpor and hibernation, ATP supply and T_b are dramatically reduced in multiple tissues, particularly in skeletal muscles, and the TCA cycle enzymes such as pyruvate DH are repressed ((118) and refs. therein). For example, pyruvate carboxylation in intact mitochondria is decreased by 75% during hibernation and mitochondrial, but not cellular, ATP/ADP/AMP are also reduced (119). A recent comparative study of several hibernators showed that genes involved in mitochondrial oxidation and pyruvate metabolism in skeletal muscles were most significantly associated with hibernation phenotypes (120), but their alternative splicing was not studied. Moreover, Ogdh isoforms were among top-ranked proteins differentially expressed in the brainstem and skeletal muscle proteomes in summer active *versus* hibernating ground squirrels (121,122), but their exact identities and underlying mRNA isoforms were not determined. Among differentially expressed factors, including Ogdh, mitochondrial genes were highly enriched, particularly in striated muscles (122-124), but exon-level comparisons were not performed. Both Atp5c1 (Figure 9B-D) and Ogdh were identified among proteins differentially expressed in skeletal

muscles of ground squirrels, even when comparing March arousals with April activity (103), but alternatively spliced mRNA isoforms were not studied. Recent transcriptomics analysis in marsupial *Dromiciops gliroides* during hibernation revealed 566 transcripts that were significantly up-regulated during hibernation (369 in brain, 147 in liver and 50 in skeletal muscle) and 339 down-regulated transcripts (225 in brain, 79 in liver and 35 in muscle), including alterations of spliceosome components (125).

As compared to non-hibernating mammals, hibernators such as ground squirrels exhibit enhanced muscle contractility and remarkable ability to maintain stable Ca^{2+}_c levels at low temperatures (eg.,(126,127)), suggesting that they evolved mechanisms that prevent Ca^{2+}_c overload. Low temperatures reduce the rate of Ca^{2+} removal from cytosol although the relative contribution of plasma and organelle channels in hibernator tissues are poorly understood (126). Cardiac sarcoplasmic reticula from ground squirrels showed faster Ca^{2+} uptake than non-hibernators, but SERCA activities did not appear to be distinct ((126) and refs. therein). Brain mitochondria of ground squirrels were able to load significantly less Ca^{2+} during torpor than in spring animals (128). Interbout arousal squirrels displayed a striking increase in intracellular Ca^{2+} concentration (129).

Rapid increase in ATP supply during arousal periods (121,122) would require a control of the cyclical activation of TCA cycle enzymes, such as Ogdhc and its MXE regulators. For example, TIA-1 (Figure 1F) showed up to a sevenfold increase in relative protein levels in the nucleus during hibernation (130), which would repress Ca^{2+}-sensitive isoform 4b+. When exposed to low temperatures, distal muscles trigger formation of TIA-1-containing stress granules (131).

Collectively, these studies support a profound role of Ca^{2+}_c and energy metabolism in hypometabolic states, and some directly point to Ca^{2+}-activated TCA cycle enzymes. As hypometabolic states in birds and mammals may share the same ancestral origin with reptiles (132) and *Ogdh* intron 4a in reptiles reached the same size as in endotherms, regulation of the MXE pairs could provide valuable clues about their evolution. Expanding the number of available hibernator genomes/transcriptomes coupled with exon-level transcriptomic data should facilitate future insights into molecular mechanisms underlying evolution of these phenotypes.

Discussion S4 Dichotomy of *OGDH* splicing in neurons and astrocytes
A raison d'être?

Presynaptic Ca^{2+} is the principal regulator of neurotransmitter release and synaptic plasticity ((133) and refs. therein). Presynaptic terminals are densely packed with mitochondria to support high demand for energy at the synapse. The absence of mitochondria in the terminals of *dmiro* (a GTPase that binds Ca^{2+}) fly mutants leads to locomotory defects (133,134). MCU is very sensitive to pre-synaptic Ca^{2+} levels (135), but unlike in striated muscles, the MCU deletion in brain synaptic or non-synaptic mitochondria does not lead to a complete block of Ca^{2+} uptake (136).

In eukaryotes, Ca^{2+}_c elevations come from intracellular and extracellular sources. In striated muscles, the bulk originates from intracellular stores, largely from the sarcoplasmic reticulum, whereas the main source of Ca^{2+}_c spikes in neurons, but not in astrocytes, is extracellular Ca^{2+}. Elevations of Ca^{2+} in visceral organs come both from the outside and the inside ((137) and refs. therein).

Neurons and their precursors repress the Ca^{2+}-sensitive *OGDH* isoform, unlike non-excitable astrocytes or striated muscles (Figure S10). In response to stress, OGDHC activity fluctuates less in the brain than in striated muscles or in liver (56). Astrocytes respond to synaptic activity with Ca^{2+}_c elevations, which stimulate chemical transmitters, including glutamate (138). Astrocytes consume considerable energy to remove/release neurotransmitters, restore ion gradients and maintain other homeostatic processes ((139,140) and refs. therein). Excitatory neurotransmition mediated by astrocytes is a critical contributor to brain energy needs (141), consistent with their high requirement for Ca^{2+}-stimulated ATP supply and high exon 4b+/4a+ ratios (Figure S10).

Astrocyte size and number of their processes dramatically expanded during vertebrate evolution, with a single human cell employing up to 2 million synapses ((141) and refs therein). Ca^{2+} regulates mitochondrial mobility in astrocyte processes, and mitochondria in turn control Ca^{2+} signals (142).

Astrocytes are highly sensitive to physiological hypoxia, are capable of efficiently modulating vaso-constriction/-dilation and can determine the exercise capacity through adaptive respiratory responses in conditions of increased metabolic demand (143,144), acting essentially as functionally specialized oxygen sensors. They respond to low oxygen levels with increased exocytosis of ATP-containing vesicles (143). Unlike a massive depletion of ATP upon inhibition of mitochodrial respiration in neurons, astrocytes are able to limit the ATP decline (141).

Apart from providing global support for neural circuits, astrocytes also exert local control over individual synapses or a small group of synapses, acting as regulatory units for the astrocyte-neuron metabolic cooperation (139,140). They express a variety of neurotransmitter receptors including glutamate receptors, which induce transient increases of Ca^{2+}_c, particularly in localized microdomains (140). The microdomain Ca^{2+} transients colocalize with mitochondria; these organelles are highly abundant in astrocytic microdomains at a density comparable to nerve terminals, reflecting a high demand for ATP (139). Spatially restricted Ca^{2+} transients in astrocyte processes that are independent of the release from endoplasmic reticulum stores result from the Ca^{2+} efflux from mitochondria via transient openings of the permeability transition pore, generating microdomain Ca^{2+} signals (139). Enhanced neuronal firing in vivo, such as arousal during locomotion, increases the microdomain activity of astrocytes in the absence of signalling through a Ca^{2+} release channel (IP3R2/ITPR2), linking the microdomain Ca^{2+} transients to the metabolic rate (139). Astrocyte Ca^{2+}_c elevations may be as rapid as in neurons (145) and can propagate to astrocytic perivascular endfeet to regulate the vasomotion and microcirculation and to meet rapid energy demand in the areas of elevated neuronal activity (146). Recovery from the glutamate-induced rise of Ca^{2+}_c is more efficient in astrocytes than in neurons (147).

The ratio of *OGDH* exons 4a/4b in myelinating oligodendrocytes (MOs), but not in their precursors, is similar to neurons (Figure S10). MOs express many molecules that make them susceptible to excitotoxic cell death, including glutamate receptors (148). MOs express P2X7, which makes them prone to detrimental effects of sustained levels of extracellular ATP (148,149). ATP is a major excitatory neurotransmitter in the central nervous system, activating ionotropic (P2X) and metabotropic (P2Y) receptors. ATP-gated P2X channels are Ca^{2+}-permeable and participate in fast synaptic transmission and modulation; ATP signalling may trigger oligodendrocyte excitotoxicity via activation of Ca^{2+} permeable P2X7 receptors (149). Glial function started to evolve in invertebrates, but reactive astrocytes are still absent in zebrafish (150), which may not yet gained an optimal MXE control (Figure S6).

Further clues to our understanding of distinct *Ogdh* exon 4a+/4b+ ratios in neurons and glia (Figure S10) may lie in the malate-aspartate shuttle (MAS). In MAS, glutamate and malate enter the mitochondrial matrix in exchange for aspartate through Ca^{2+}-sensitive aspartate/glutamate carriers aralar (SLC25A12) and citrin (SLC25A13; for so<u>l</u>ute <u>c</u>arriers A12 and A13) while 2OG is transported out through the oxoglutarate carrier (SLC25A11). SLC25A12-deficient neurons showed decreased respiration levels and a failure to regulate respiration rates in response to Ca^{2+} (151). A lack of Ca^{2+} stimulation observed under workload in SLC25A12 knock-out neurons (152) indicates that SLC25A12 is a major contributor of Ca^{2+}-stimulated respiration in neurons by providing increased pyruvate supply to mitochondria. SLC25A12 is preferentially expressed in the brain and striated muscles. In the brain, the SLC25A12 expression is most abundant in neurons (24), and the protein is thought to be functional only in this cell type (reviewed in (153)). MAS reconstitution using isolated mitochondria showed that activation of OGDH by Ca^{2+}_m reduced efflux through SLC25A11, decreasing the MAS activity as a result of competition between MAS and TCA cycles for the shared metabolite 2OG (154). Aralar has a higher Ca^{2+} affinity than the MCU and even tiny Ca^{2+} signals can stimulate ATP (155). In plants, malate DH has a high flux control coefficient for respiration (52). A mutual exclusivity of MAS, the main NADH shuttle in the brain, and MCU-DH pathways in brain mitochondria under Ca^{2+}-stimulated conditions (155) might have facilitated *Ogdh* exon 4b repression in neurons. Under the sequential MAS and the MCU-DH activation model (154), the Ca^{2+} activated OGDHC may become the main NADH producing pathway when Ca^{2+}_m transients are high following the MCU activation. Finally, 2OG as an important source of glutamate and glutamine directly inhibits F_1F_o-ATP synthase by binding ATP5B, reducing ATP and

oxygen consumption and increasing the *C. elegans* lifespan (156). This link may expaned by the shared exon-level regulation of F_1F_o-ATP synthase and *OGDH* MXEs (Figure 9B-D).

Taken together, the important role of astrocytes in neuroenergetics was associated with derepression of *OGDH* exon 4b in this cell type (Figure S10). Prominent exon 4b activation in astrocyte Ca^{2+} microdomains would provide responsive ATP supply and superior synaptic and locomotory control *en route* to endothermy. In contrast, exon 4b repression in neurons and MOs (Figure S10) may have been required to prevent excitoxicity, in line with a strong pressure against energy dissipation in the brain (157). The key role of OGDHC in glutamate metabolism should provide additional clues for the dichotomy of this MXE pair in neurons and glia.

Regulation of OGDH exon 5 splicing in neurons

Neurons adopted an extra exon 5, which encodes additional 15 amino acids (Figure S8-S10) (4). It remains to be confirmed when exactly this event evolved, possibly before the split of cartilaginous and bony fish (4). The inclusion of exon 5 in mRNAs suggests that a simple on/off switch of MXEs to reduce or increase the Ca^{2+}-dependent OGDHC activation is not satisfactory in this cell type. Exon 5 inclusion is associated with exon 4a, albeit not exclusively (Figure S10), raising a possibility that it may fine-tune OGDHC inhibition. The *OGDH* 4a+5+ or 4b+5+ isoforms could modulate Ca^{2+}-sensitivity of OGDHC since both isoforms showed much attenuated response to Ca^{2+}; among tested E1, isoform 4a+5+ was associated with the lowest enzyme activation at high Ca^{2+} concentrations, nevertheless the loss of Ca^{2+} -sensitivity was accompanied only by modest decreases in sensitivity to inhibition by NADH and elevated ATP/ADP ratios (4).

The neuron-specific exon 5 activation can be explained by the presence of an activator and/or a lack of repressor in this cell type. One candidate for the repressive regulation is U2AF35 (17). U2AF35 isoforms are expressed at similar levels as U2AF65 in skeletal muscles, but U2AF35a mRNAs are low in the brain, less than half of the U2AF65 mRNA levels (158,159). U2AF35 but neither U2AF65 nor PUF60 knockdown activated exon 5 inclusion (Figures S6 in ref. (17)). The U2AF35a/U2AF35b ratio is also low in the brain and high in skeletal muscles (159). The two U2AF35 isoforms play an important role in regulated splicing (158,160-162), which could be involved in ensuring protection of neurons against Ca^{2+}-dependent ATP oversupply and neurotoxicity.

Discussion S5 Endothermy and body mass: clues from *Ogdh* MXEs?

Smaller organisms have a larger surface-to-volume ratio than larger ones and heat and cool faster, suggesting that selection for the body size was important in the acquisition of homeothermy and for evolution of hypometabolic states (163-167). Mitochondrial function in relation to body mass has been studied extensively both in endotherms and ectotherms. For example, small endotherms were proposed to have a lower mitochondrial efficiency than larger species and the production of ATP and reactive oxygen species have been correlated with body mass (168,169). Mitochondrial efficiency to generate ATP is reduced by proton leak, a dissipation of the protonmotive force through mitochondrial inner membranes, diverting energy away as heat (168,169). During vertebrate evolution, however, this reduction should promote selection processes that favour activity-dependent ATP supply pathways.

Selection routes shaping body or organ size are controlled by apoptotic cellular decisions. Such choices involve Ca^{2+} signalling both in extrinsic (receptor-mediated) and intrinsic (mitochondria-mediated) cell death pathways (170), including $Ca^{2+}{}_m$ spikes (171) and Ca^{2+} regulators in endoplasmic reticula (reviewed by (170)). Importantly, $Ca^{2+}{}_m$ overload acts as a proapoptotic signal that induce mitochondria swelling and the release of mitochondrial apoptotic factors into the cytosol (172). Local communication between organelles can propagate Ca^{2+}-mediated apoptotic signals over relatively large distances, which would ensure coordinated execution of apoptosis across large cells, particularly cells with high mitochondrial densities (171). Ca^{2+} is involved in multiple cell death modalities including autophagy, which can be triggered by very subtle changes in Ca^{2+} distribution within intracellular compartments (170).

In large marine species, the mass-specific metabolic rate decreases with increasing body mass, suggesting that active macropredation cannot be sustained once a given body size is reached and only less active strategies such as filter feeding are physiologically affordable above a certain threshold (173). Endothermic macropredators can attain larger body masses than their ectothermic counterparts and endothermy was even proposed to play a role in the evolution of gigantism in extinct macropredatory groups ((173) and refs. therein). Ocean sunfish (*M. mola*), the *Ogdh* intron 4a AGEZ outlier (Figures 6E and S13), is the world's largest bony fish weighing up to 2.3 tons and native only to tepid and tropical waters; prolonged periods spent in water at temperatures of 12 °C or lower lead to disorientation and death (174). The AGEZ of *M. mola Ogdh* intron 4a is violated, but the associated exon 4b skipping was not rescued by extending the AGEZ (Figure S13). Nevertheless, this result does not completely exclude physiological significance of the reduced AGEZ in endogenous expression. If this reduction does decrease Ca^{2+}-dependent OGDH activation by repressing authentic 3'ss of intron 4a, activity-related ATP supply by sunfish mitochondria may not be sufficient for muscle thermogenesis to cope with cold ambient temperatures, preventing predatory activities, selection for higher MMR and limiting the habitat.

Taken together, the proposed link between diversification of *Ogdh* isoforms and the acquisition of endothermy may extend beyond the Ca^{2+}_m -stimulated ATP supply. OGDHC occupies a critical position between carbohydrate, amino acid and fatty acid metabolism (50,56), which may provide clues for our understanding of the role of body mass and blubber insulation in the evolution of endothermy, both in water and terrestrial environments.

Discussion S6 Alternative pre-mRNA splicing and endothermy: a search for candidate isoforms

The division between endotherms and ectotherms is not absolute. Intermediate phenotypes range from mammals with lower T_b and intrinsic metabolic activities (monotremes, most marsupials) or hypometabolic states (torpor, hibernation; Discussion S3) to 'partially endothermic' reptiles, such as Burmese python or leatherback turtles (175,176), fish, such as lamnid sharks, tunas and opah (70,177), and extinct species, including dinosaurs (178). This large body of indirect evidence and a growing support for the aerobic capacity model (179-181) strongly indicate that these regional or partial endotherms (also termed 'mesotherms' (178)) already harboured heritable mechanisms that promoted selection for MMR.

The acquisition of endothermy as well as other complex traits cannot be explained by a single selection event, isoform or DNA variant (165,167). Nevertheless, it is conceivable that only a limited number of molecular 'drivers' on the energy supply side may have been necessary for orchestrating selection processes that led to this animal innovation. This concept is compatible with the independent emergence of many 'mesothermic' taxa that share the same metabolic features (132,165,167,182), apparently driven largely by Ca^{2+}-dependent muscle thermogenesis (Discussion S2). Although OGDHC activation by Ca^{2+} is a critical step in activity-driven NADH and ATP supply (Discussion S1), activation of a single enzyme may not always produce massive increases in flux in complex metabolic pathways (183). Effective control often involves multisite modulation involving many enzymes and their isoforms (183). The TCA cycle is no exception, yet only a subset of enzymes showed positive flux coefficients (52). The multisite modulation would require coregulation of these enzymatic reactions, including tissue-specific components of the respiratory chain (66). Regulation of distant steps can be conveniently accomplished at the exon level (Figure 9B-D) and RBPs that bind accessible PPTs seem to be well-suited for this task, providing a wealth of exon activating and inhibitory effects (Figure 1).

Alternatively spliced gene segments preferentially encode peptides that are intrinsically disordered and/or contain linear interaction motifs or posttranslational modification sites (184). The resulting protein isoforms can evolve distinct function in different tissues and organisms by rewiring interaction networks through the recruitment of distinct interaction partners, contributing to the emergence of new traits (184). Which mRNA isoforms are respectable candidates for their role in the evolution of warm-bloodedness?

Assuming the validity of aerobic capacity model (179), genes encoding components of mitochondrial Ca^{2+} signalling pathways, many displaying Ca^{2+}-regulated MXEs (Tables S6,S7) and tissue-specific isoform expression similar to *OGDH*, would appear to be strong suspects. Ca^{2+} activates

several other mitochondrial enzymes encoded by alternatively spliced genes, including mitochondrial isocitrate DH and F_1F_o-ATPase components (44,185). The isocitrate DH β subunit (*IDH3B*) also contains homologous MXEs (186). In the context of protein organization, cofactors, and the mechanism of action, OGDHC is similar to the pyruvate DH complex (52), which competes in mitochondria with OGDHC for NAD and CoA (187). MCU silencing increased pyruvate DH phosphorylation and decreased activity of the enzyme (85), consistent a compensatory TCA cycle responses, although the OGDHC activity was not investigated.

Second, ATP-generating pathways have been under strong selection pressure during evolution, ensuring the right balance between the rate and yield of ATP production (188). The high ATP yield of cellular respiration probably facilitated evolutionary transition from unicellular to early multicellular organisms (188). The F_1F_o-ATPase converts the protonmotive capacity into ATP and its γ-subunit is regulated by alternatively spliced *ATP5C1* exon 9 in the heart and skeletal muscles. This alternative splicing event is controlled by a muscle-specific exonic silencer (189), by Fox-1 (*A2BP1*) through GCAUG *cis*-elements (190), and by PUF60 (Figure 9B-D and Table S8). Fox-1 is expressed exclusively in brain and muscles (191). Examination of mitochondrial matrix proteomes in porcine hearts revealed many phosphorylation events associated with Ca^{2+} signalling, including sites in OGDHC and the γ-subunit of F_1F_o-ATPase (192).

Turning to Ca^{2+}_m transporters, a skeletal muscle- and vertebrate-specific microexon in *MICU1* confers high sensitivity of the mitochondrial Ca^{2+} uptake machinery and is required for sustained ATP production (193). Intronic sequences surrounding this microexon are highly conserved, including a putative UAA BP located 32 nts upstream, and the microexon is located close to alternative transcription initiation sites that produce shorter annotated transcripts (I.V., unpublished data). Impaired Ca^{2+} handling was also observed for knockouts of cardiac splicing factors, including SRSF2 and SRSF10 (194).

Fourth, one of the highest fractions of tissue-specific alternative splicing was found in skeletal muscles (62). Splicing of troponin transcripts, including Ca^{2+}-dependent troponin C, might explain isometric tension differences of flight muscles between insect species (195) and their variable thermogenic potential. Troponin T is a central player in the Ca^{2+} regulation of actin thin filament function and is essential for the contraction of striated muscles (98). The expression of slow-skeletal muscle gene (*TNNT1*) involves alternative splicing of exon 5, which is PUF60-dependent and is upregulated in cells lacking U2AF ((17) and J.K. and I.V., unpublished data). The hearts of dry land frogs utilize the slow-muscle *TNNT1* rather than canonical cardiac *TNNT2* ((98) and refs. therein). The Ca^{2+}-induced fast-to-slow myosin transition was associated with elevated citric synthase (196). Finally, ATP generation in response to high demand in muscles may be influenced by alternative RNA processing of transcripts encoding uncoupling proteins as well as other Ca^{2+}-sensitive mitochondrial enzymes, including those in the NADH glycerol-3-phosphate shuttle, a key link between lipid and carbohydrate metabolism (71).

Fifth, alternative pre-mRNA splicing is likely to play a pivotal role in the regulation of futile or substrate cycles that occur when two metabolic pathways run simultaneously in opposite directions, dissipating heat by ATP hydrolysis (157). A large number of futile cycles that consume ATP has been identified in both prokaryotes and humans (157,197), with the median length of predicted cycles in humans of ~35 and most cycles spanning several cellular compartments, often involving (de)phosphorylation (157). Selective pressure also acts against the uncontrolled dissipation of energy by avoiding the coexpression of enzymes belonging to the same substrate cycle; these selective forces are particularly strong against high-flux futile cycles (157). Futile cycles can be highly sensitive to very small changes in enzyme activities (198), which can be easily modified by alternative splicing.

In conclusion, although the candidate list is far from complete, this discussion may help identify RNA processing events important in selection processes that led to endothermy. Expanding transcriptomic and exon-level data from extant species should permit more detailed studies in the future, ultimately identifying key selection pathways *en route* to this highly successful animal strategy.

Discussion S7 Endothermy in monotremes and marsupials: TCA cycle clues?
Monotreme and marsupial T_b and metabolic rates are on average lower than those of placentals (199,200). Monotreme cell lines must be maintained at 32 °C, close to their average $T_{b,}$ because they do not survive mean T_b of placentals or birds (201). Splicing of endogenous *Ogdh* transcripts including brain-specific exon 5 in monotrems and marsupials is similar to other mammals (Figure S9), arguing against the involvement of MXEs in reduced metabolic rates and T_b in this group of mammals. Except for platypus, their DNA variability within the dBP cluster is similar to other homeotherms and cannot explain a unique mammalian thermoconformity of the naked mole rat either (Figure S1), supporting the role of other molecular drivers in this group of animals.

Unlike placental mammals and birds, however, monotremes and marsupials have functional malate synthase genes, a key enzyme of the glyoxylate cycle, a TCA cycle variation present in plants, fungi, protists and bacteria (202). The glyoxylate bypass skips two rate-limiting decarboxylation steps of the TCA cycle catalyzed by IDH and OGDHC and employs isocitrate lyase and malate synthase instead (203). Mice expressing the glyoxylate shunt had reduced total liver ATP and malonyl-CoA levels compared to lacZ-injected mice and develop resistance to diet-induced obesity (204). The ATP reduction was attributed to the shunt bypassing the oxidative portion of the TCA cycle and the possibility that the NADH produced by cytosolic malate dehydrogenase was oxidized in lactate rather than shuttling back into mitochondria. The reduced ATP might explain the observed AMP-activated protein kinase activation in the HepG2 cell line constitutively expressing *E. coli* glyoxylate shunt genes (204). The AMP-activated kinase responds to low energy states by enhancing ATP producing pathways and inhibiting energy consuming pathways such as fatty acid biosynthesis, suggesting that despite increased fatty acid oxidation, the shunt mice generates less energy per oxidized fatty acid than the wild-type mice.

This energy dissipation resembles the mechanism by which uncoupling proteins (UCPs) regulate the whole body energy balance. UCPs decrease metabolic efficiency by dissipating the proton gradient in mitochondria, causing energy created from metabolism to be released as heat. UCPs have been studied as candidate proteins for the transition from ecto- to endothermy, with the most prominent member UCP1 proposed to facilitate non-shivering thermogenesis in mammalian BAT (205). However, an avian UCP is not involved in uncoupling of respiration and metabolic heat (206), birds do not possess BAT and there is no evidence for BAT thermogenesis in marsupials and no evidence for BAT in monotremes ((199,206) and refs. therein). The activity of UCPs appears to modulate the efficiency of oxidative phosphorylation, presumably by catalyzing proton re-entry into the matrix (207). However, at least some of the physiological effects of UCPs might be explained by Ca^{2+} transport, with UCP2/3 facilitating Ca^{2+} flux across the inner mitochondrial membrane and not directly supporting thermogenic function (208). UCPs were proposed to be essential for Ca^{2+} uptake to mitochondria, but animal knockouts revealed little or no effect (209,210). Mice overexpressing UCPs in skeletal tissue resisted diet-induced obesity and the liver-specific UCP1 expression was associated with increased energy expenditure and decreased liver triglycerides ((204) and refs. therein).

Taken together, similar to UCPs, the glyoxylate shunt may alter the energy state of the cell in part by decreasing the energy produced from fatty acid oxidation. This TCA branch point could interfere with cellular energy needs in vertebrates that express relics of the shunt pathway. We speculate that the flux diversion via malate synthase and other activities could reduce ATP supply and modulate evolutionary pathways that led to typical homeothermy.

Discussion S8 A timeline of *OGDH* MXE regulation and evolution of endothermy in vertebrates
The DADLD motif is present in multiple ***fish*** species (4), in which tissue-specific MXE patterns and regulation do not appear to be fully developed (Figure S6, S9, S13) and may not be sufficient for optimal Ca^{2+}-dependent control of Ohdh in their water milieu. Fish have the widest range of *Ogdh* intron 4a length diversity, only a single primordial dBP and the largest bone fish, but not other fish, have a reduced AGEZ (Figure 1B, 6E, S13; Discussion S5). However, teleost fish already show correlated mass- and temperature-adjusted BMR and MMR (180). Fish with more active lifestyles have higher BMR when comparing various taxa living at similar temperatures. In pelagic teleost predators, BMR correlates with

protein-rich skeletal musculature and caudal fin aspect ratios, but not with the brain mass (180). Fish predators exhibit regional endothermy more often than the opposite end of their life-style continuum, such as sluggish, sit-and-wait cyprinids, which have low BMR and high tolerance to hypoxia (180). This continuum was linked to body mass, with a trend toward less active life style in bigger marine species (173). Partially endothermic fish such as tunas were found to have a higher proportion of slow-twitch muscles relative to fast-twitch counterparts than other teleosts ((211) and refs. therein; Discussion S2). Some sharks such as *Callorhinchus millii* lack the DADLD motif and would be expected to exhibit greatly attenuated activation of OGDHC (4,60). More generally, reduced capacity to activate OGDHC could manifest as intolerance to low ambient temperatures, selective stenothermal altitude and largely coastal habitats. In any case, this vertebrate class shows convincing signs of early selection for endothermy and must have already possessed a molecular 'gas-pedal' mechanism for sustained energy supply to muscles. Fish will therefore continue to provide an ideal taxonomic context for studying early events leading to endothermy (182).

With only one dominant and nonredundant dBP in long intron 4a, the regulatory *OGDH* MXE potential in ***amphibians*** may still be suboptimal (Figure 1B, S6, S12-S13). However, primary transcripts in *X. laevis* already show efficient muscle-specific promotion of exon 4b (Figure S9) and frog OGDHC is activated by Ca^{2+} even if addition of Ca^{2+} decreased the K_m value for 2OG to a lesser extent than in placentals (29) (Figure S15). Amphibians also show a brain-specific repression of isoforms 4b+ together with activation of exon 5 (Figure S9) (Discussion S4). The extra codon in exon 4a in fish and amphibians, which is absent in reptiles, birds and mammals (Figure S1), promotes the inclusion levels of this exon in mature transcripts (Figure 5), which could limit maximum Ca^{2+}-mediated OGDHC activation by isoform 4b+.

A narrow range of intron 4a size typical of mammals was first achieved in ***reptiles***, barring crocodilians (Figure 1C and S1). The length of crocodilian intron 4a is intermediate between endo- and ectotherms (Figure 1C), probably reflecting an exceptionally slow evolutionary rate of microdeletions (212). This rate was much slower than that of lizards, which have the intron 4a length typical of mammals. Nevertheless, the aerobic scope and tidal volume of alligators are close to values observed in endotherms although the total power generated by maximally active crocodiles is lower than in mammals of the same size (213,214). However, this reptile class has evolved piston lungs and four-chambered hearts, which prevent mixing of de- and oxygenated blood. This separation is typical of homeotherms and can deliver blood to tissues at higher pressure, facilitating adaptive tissue oxygen supply and oxidative phosphorylation. More generally, reptiles already exhibit many features of mammalian or avian endothermy, including the ability to enhance metabolic rate by muscular activities decoupled from locomotion, circadian T_b rhythms not associated with activity and a sophisticated peripheral vascular control, indicating that they already possess selection instruments for incipient facultative endothermy ((132,175,176) and refs. therein). Field factorial scopes, which may better reflect sustained aerobic performance than MMR, are quite similar for reptiles, mammals and birds (166). Some reptiles, such as brooding pythons, efficiently integrate shivering thermogenesis and basking, which is not dissimilar to torpidating marsupials, suggesting that reptiles rather than fish could be 'protoendotherms' (132). Stronger skeletal muscles in terrestrial reptiles would stimulate muscle thermogenesis (Discussion S2), which has been best studied in brooding pythons and leatherback turtles (132,175,176). For example, deep T_b of leatherback turtles, the largest living marine turtles, is higher than water temperature as a result of muscular activity, which increases as surrounding temperature is lowered (176,215). Leatherback turtles have a high energy intake and are highly migratory, up to 18,000 km between tropical and northern foraging grounds ((216) and refs. therein). The reptile dBP cluster resembles that of birds, however, their dBPs are upstream of megaPPTs with a lower U content (Figure S7). This might reduce the repertoire of U-bound regulators and lead to a less responsive regulation of MXE usage.

Retention of metabolic heat by feathers or fur would help conserve the energy and maintain higher T_b for longer ((166) and refs. therein). T_b of ***birds*** is on average significantly higher than mammalian T_b ((217) and refs. therein). Alternative pre-mRNA splicing in birds is already extensive although chicken genes still generate, on average, fewer transcripts per gene and have shorter introns than

humans or mice (218). The *Ogdh* endogenous splicing pattern in the brain and viscera of *C. japonica* is similar to the mammalian pattern (Fiure S9). The avian dBP cluster, however, largely employs efficient orthologues of low-usage human dBP+41 (Figure 3H and 6D), which has the optimal dBP motif UAA (Figure S1, S4). The dominant bird dBP is also more accessible than the human counterpart (Figures 3, 6-8) and seems to contribute to the excessive exon 4b+/4a+ ratios observed for exogenous chicken transcripts (Figure S13).

In contrast to birds, ***monotremes*** and most ***marsupials*** have lower T_b than placental mammals (Discussion S7). The length of platypus intron 4a is intermediate between aquatic and terrestrial species and the platypus dBP+31 ortologue is inefficient, but the endogenous splicing pattern of tested monotremes and marsupials was similar to placentals (Figure S9). Finally, the tentative role of OGDHC in hypometabolic states of ***mammalian hibernators*** is explained in Discussion S3.

Collectively, this timeline suggests that changes in the *Ogdh* MXE regulation influenced the capacity of animals to optimize Ca^{2+}-mediated responses to sustained energy demand and maximum locomotory endurance *en route* to endothermy.

REFERENCES TO SUPPLEMENTAL INFORMATION

1. Jurka, J., Kapitonov, V.V., Pavlicek, A., Klonowski, P., Kohany, O. and Walichiewicz, J. (2005) Repbase Update, a database of eukaryotic repetitive elements. *Cytogenet. Genome Res.*, **110**, 462-467.
2. Yeo, G. and Burge, C.B. (2004) Maximum entropy modeling of short sequence motifs with applications to RNA splicing signals. *J. Comput. Biol.*, **11**, 377-394.
3. Kielkopf, C.L., Lucke, S. and Green, M.R. (2004) U2AF homology motifs: protein recognition in the RRM world. *Genes Dev.*, **18**, 1513-1526.
4. Denton, R.M., Pullen, T.J., Armstrong, C.T., Heesom, K.J. and Rutter, G.A. (2016) Calcium-insensitive splice variants of mammalian E1 subunit of 2-oxoglutarate dehydrogenase complex with tissue-specific patterns of expression. *Biochem. J.*, **473**, 1165-1178.
5. Soom, M., Gessner, G., Heuer, H., Hoshi, T. and Heinemann, S.H. (2008) A mutually exclusive alternative exon of slo1 codes for a neuronal BK channel with altered function. *Channels*, **2**, 278-282.
6. Tseng-Crank, J., Foster, C.D., Krause, J.D., Mertz, R., Godinot, N., DiChiara, T.J. and Reinhart, P.H. (1994) Cloning, expression, and distribution of functionally distinct Ca(2+)-activated K+ channel isoforms from human brain. *Neuron*, **13**, 1315-1330.
7. Ramanathan, K., Michael, T.H., Jiang, G.J., Hiel, H. and Fuchs, P.A. (1999) A molecular mechanism for electrical tuning of cochlear hair cells. *Science*, **283**, 215-217.
8. Sorensen, J.B., Nagy, G., Varoqueaux, F., Nehring, R.B., Brose, N., Wilson, M.C. and Neher, E. (2003) Differential control of the releasable vesicle pools by SNAP-25 splice variants and SNAP-23. *Cell*, **114**, 75-86.
9. Johansson, J.U., Ericsson, J., Janson, J., Beraki, S., Stanic, D., Mandic, S.A., Wikstrom, M.A., Hokfelt, T., Ogren, S.O., Rozell, B. *et al.* (2008) An ancient duplication of exon 5 in the Snap25 gene is required for complex neuronal development/function. *PLoS Genet*, **4**, e1000278.
10. Hatje, K., Rahman, R.U., Vidal, R.O., Simm, D., Hammesfahr, B., Bansal, V., Rajput, A., Mickael, M.E., Sun, T., Bonn, S. *et al.* (2017) The landscape of human mutually exclusive splicing. *Mol Syst Biol*, **13**, 959.
11. Hastings, M.L., Allemand, E., Duelli, D.M., Myers, M.P. and Krainer, A.R. (2007) Control of pre-mRNA splicing by the general splicing factors PUF60 and U2AF. *PLoS ONE*, **2**, e538.
12. Page-McCaw, P.S., Amonlirdviman, K. and Sharp, P.A. (1999) PUF60: a novel U2AF65-related splicing activity. *RNA*, **5**, 1548-1560.
13. Zamore, P.D., Patton, J.G. and Green, M.R. (1992) Cloning and domain structure of the mammalian splicing factor U2AF. *Nature*, **355**, 609-614.
14. Singh, R., Valcarcel, J. and Green, M.R. (1995) Distinct binding specificities and functions of higher eukaryotic polypyrimidine tract-binding proteins. *Science*, **268**, 1173-1176.
15. Bouck, J., Litwin, S., Skalka, A.M. and Katz, R.A. (1998) In vivo selection for intronic splicing signals from a randomized pool. *Nucleic Acids Res.*, **26**, 4516-4523.
16. Bloom, K., Mohsen, A.W., Karunanidhi, A., El Demellawy, D., Reyes-Mugica, M., Wang, Y., Ghaloul-Gonzalez, L., Otsubo, C., Tobita, K., Muzumdar, R. *et al.* (2018) Investigating the link of ACAD10 deficiency to type 2 diabetes mellitus. *J. Inherit. Metab. Dis.*, **41**, 49-57.
17. Kralovicova, J., Sevcikova, I., Stejskalova, E., Obuca, M., Hiller, M., Stanek, D. and Vorechovsky, I. (2018) PUF60-activated exons uncover altered 3' splice-site selection by germline missense mutations in a single RRM. *Nucleic Acids Res.*, **46**, 6166-6187.
18. Ascher, D.B., Spiga, O., Sekelska, M., Pires, D.E.V., Bernini, A., Tiezzi, M., Kralovicova, J., Borovska, I., Soltysova, A., Olsson, B. *et al.* (2019) Homogentisate 1,2-dioxygenase (HGD) gene variants, their analysis and genotype-phenotype correlations in the largest cohort of patients with AKU. *Eur. J. Hum. Genet.*
19. Kralovicova, J., Haixin, L. and Vorechovsky, I. (2006) Phenotypic consequences of branchpoint substitutions. *Hum. Mutat.*, **27**, 803-813.
20. Caldwell, R.B., Kierzek, A.M., Arakawa, H., Bezzubov, Y., Zaim, J., Fiedler, P., Kutter, S., Blagodatski, A., Kostovska, D., Koter, M. *et al.* (2005) Full-length cDNAs from chicken bursal lymphocytes to facilitate gene function analysis. *Genome Biol*, **6**, R6.
21. Smith, C.W. and Nadal-Ginard, B. (1989) Mutually exclusive splicing of alpha-tropomyosin exons enforced by an unusual lariat branch point location: implications for constitutive splicing. *Cell*, **56**, 749-758.
22. Southby, J., Gooding, C. and Smith, C.W. (1999) Polypyrimidine tract binding protein functions as a repressor to regulate alternative splicing of alpha-actinin mutally exclusive exons. *Mol. Cell. Biol.*, **19**, 2699-2711.

23. Consortium, G.T. (2013) The Genotype-Tissue Expression (GTEx) project. *Nat. Genet.*, **45**, 580-585.
24. Zhang, Y., Chen, K., Sloan, S.A., Bennett, M.L., Scholze, A.R., O'Keeffe, S., Phatnani, H.P., Guarnieri, P., Caneda, C., Ruderisch, N. *et al.* (2014) An RNA-sequencing transcriptome and splicing database of glia, neurons, and vascular cells of the cerebral cortex. *J. Neurosci.*, **34**, 11929-11947.
25. Vogel, J., Hess, W.R. and Borner, T. (1997) Precise branch point mapping and quantification of splicing intermediates. *Nucleic Acids Res.*, **25**, 2030-2031.
26. Gao, K., Masuda, A., Matsuura, T. and Ohno, K. (2008) Human branch point consensus sequence is yUnAy. *Nucleic Acids Res.*, **36**, 2257-2267.
27. Mercer, T.R., Clark, M.B., Andersen, S.B., Brunck, M.E., Haerty, W., Crawford, J., Taft, R.J., Nielsen, L.K., Dinger, M.E. and Mattick, J.S. (2015) Genome-wide discovery of human splicing branchpoints. *Genome Res.*, **25**, 290-303.
28. Wang, J., Duncan, D., Shi, Z. and Zhang, B. (2013) WEB-based GEne SeT AnaLysis toolkit (WebGestalt): update 2013. *Nucleic Acids Resear*, **41**, W77 –W83.
29. McCormack, J.G. and Denton, R.M. (1981) A comparative study of the regulation of Ca2+ of the activities of the 2-oxoglutarate dehydrogenase complex and NAD+-isocitrate dehydrogenase from a variety of sources. *Biochem. J.*, **196**, 619-624.
30. Konig, T., Troder, S.E., Bakka, K., Korwitz, A., Richter-Dennerlein, R., Lampe, P.A., Patron, M., Muhlmeister, M., Guerrero-Castillo, S., Brandt, U. *et al.* (2016) The m-AAA Protease Associated with Neurodegeneration Limits MCU Activity in Mitochondria. *Mol. Cell*, **64**, 148-162.
31. Maltecca, F., Baseggio, E., Consolato, F., Mazza, D., Podini, P., Young, S.M., Jr., Drago, I., Bahr, B.A., Puliti, A., Codazzi, F. *et al.* (2015) Purkinje neuron Ca2+ influx reduction rescues ataxia in SCA28 model. *J. Clin. Invest.*, **125**, 263-274.
32. Liu, J.C., Liu, J., Holmstrom, K.M., Menazza, S., Parks, R.J., Fergusson, M.M., Yu, Z.X., Springer, D.A., Halsey, C., Liu, C. *et al.* (2016) MICU1 serves as a molecular gatekeeper to prevent in vivo mitochondrial calcium overload. *Cell Rep*, **16**, 1561-1573.
33. Marquez, J., Mates, J.M. and Campos-Sandoval, J.A. (2016) Glutaminases. *Adv. Neurobiol.*, **13**, 133-171.
34. Takeda, K., Ishida, A., Takahashi, K. and Ueda, T. (2012) Synaptic vesicles are capable of synthesizing the VGLUT substrate glutamate from alpha-ketoglutarate for vesicular loading. *J. Neurochem.*, **121**, 184-196.
35. Kam, K. and Nicoll, R. (2007) Excitatory synaptic transmission persists independently of the glutamate-glutamine cycle. *J. Neurosci.*, **27**, 9192-9200.
36. Hertz, L. and Zielke, H.R. (2004) Astrocytic control of glutamatergic activity: astrocytes as stars of the show. *Trends Neurosci.*, **27**, 735-743.
37. Ke, S., Shang, S., Kalachikov, S.M., Morozova, I., Yu, L., Russo, J.J., Ju, J. and Chasin, L.A. (2011) Quantitative evaluation of all hexamers as exonic splicing elements. *Genome Res.*, **21**, doi10.1101/gr.119628.119110.
38. Lu, C.H., Lin, Y.F., Lin, J.J. and Yu, C.S. (2012) Prediction of metal ion-binding sites in proteins using the fragment transformation method. *PLoS One*, **7**, e39252.
39. Caceres, E.F. and Hurst, L.D. (2013) The evolution, impact and properties of exonic splice enhancers. *Genome Biol*, **14**, R143.
40. Fairbrother, W.G., Yeh, R.F., Sharp, P.A. and Burge, C.B. (2002) Predictive identification of exonic splicing enhancers in human genes. *Science*, **297**, 1007-1013.
41. Fairbrother, W.G., Yeo, G.W., Yeh, R., Goldstein, P., Mawson, M., Sharp, P.A. and Burge, C.B. (2004) RESCUE-ESE identifies candidate exonic splicing enhancers in vertebrate exons. *Nucleic Acids Res.*, **32**, W187-190.
42. Zhang, X.H. and Chasin, L.A. (2004) Computational definition of sequence motifs governing constitutive exon splicing. *Genes Dev.*, **18**, 1241-1250.
43. Goren, A., Ram, O., Amit, M., Keren, H., Lev-Maor, G., Vig, I., Pupko, T. and Ast, G. (2006) Comparative analysis identifies exonic splicing regulatory sequences. The complex definition of enhancers and silencers. *Mol. Cell*, **22**, 769-781.
44. Denton, R.M. (2009) Regulation of mitochondrial dehydrogenases by calcium ions. *Biochim. Biophys. Acta*, **1787**, 1309-1316.
45. Jouaville, L.S., Pinton, P., Bastianutto, C., Rutter, G.A. and Rizzuto, R. (1999) Regulation of mitochondrial ATP synthesis by calcium: evidence for a long-term metabolic priming. *Proc. Natl. Acad. Sci. USA*, **96**, 13807-13812.
46. Cooney, G.J., Taegtmeyer, H. and Newsholme, E.A. (1981) Tricarboxylic acid cycle flux and enzyme activities in the isolated working rat heart. *Biochem. J.*, **200**, 701-703.

47. Moreno-Sanchez, R., Hogue, B.A. and Hansford, R.G. (1990) Influence of NAD-linked dehydrogenase activity on flux through oxidative phosphorylation. *Biochem. J.*, **268**, 421-428.
48. Tretter, L. and Adam-Vizi, V. (2000) Inhibition of Krebs cycle enzymes by hydrogen peroxide: A key role of [alpha]-ketoglutarate dehydrogenase in limiting NADH production under oxidative stress. *J. Neurosci.*, **20**, 8972-8979.
49. Araujo, W.L., Nunes-Nesi, A., Trenkamp, S., Bunik, V.I. and Fernie, A.R. (2008) Inhibition of 2-oxoglutarate dehydrogenase in potato tuber suggests the enzyme is limiting for respiration and confirms its importance in nitrogen assimilation. *Plant Physiol.*, **148**, 1782-1796.
50. Bunik, V.I. and Fernie, A.R. (2009) Metabolic control exerted by the 2-oxoglutarate dehydrogenase reaction: a cross-kingdom comparison of the crossroad between energy production and nitrogen assimilation. *Biochem. J.*, **422**, 405-421.
51. Read, G., Crabtree, B. and Smith, G.H. (1977) The activities of 2-oxoglutarate dehydrogenase and pyruvate dehydrogenase in hearts and mammary glands from ruminants and non-ruminants. *Biochem. J.*, **164**, 349-355.
52. Araujo, W.L., Nunes-Nesi, A., Nikoloski, Z., Sweetlove, L.J. and Fernie, A.R. (2012) Metabolic control and regulation of the tricarboxylic acid cycle in photosynthetic and heterotrophic plant tissues. *Plant Cell Environ.*, **35**, 1-21.
53. Cortassa, S., Aon, M.A., Marban, E., Winslow, R.L. and O'Rourke, B. (2003) An integrated model of cardiac mitochondrial energy metabolism and calcium dynamics. *Biophys. J.*, **84**, 2734-2755.
54. McCormack, J.G., Halestrap, A.P. and Denton, R.M. (1990) Role of calcium ions in regulation of mammalian intramitochondrial metabolism. *Physiol. Rev.*, **70**, 391-425.
55. McCammon, M.T., Epstein, C.B., Przybyla-Zawislak, B., McAlister-Henn, L. and Butow, R.A. (2003) Global transcription analysis of Krebs tricarboxylic acid cycle mutants reveals an alternating pattern of gene expression and effects on hypoxic and oxidative genes. *Mol. Biol. Cell*, **14**, 958-972.
56. Graf, A., Trofimova, L., Loshinskaja, A., Mkrtchyan, G., Strokina, A., Lovat, M., Tylicky, A., Strumilo, S., Bettendorff, L. and Bunik, V.I. (2013) Up-regulation of 2-oxoglutarate dehydrogenase as a stress response. *Int. J. Biochem. Cell Biol.*, **45**, 175-189.
57. Pan, X., Liu, J., Nguyen, T., Liu, C., Sun, J., Teng, Y., Fergusson, M.M., Rovira, II, Allen, M., Springer, D.A. *et al.* (2013) The physiological role of mitochondrial calcium revealed by mice lacking the mitochondrial calcium uniporter. *Nat. Cell. Biol.*, **15**, 1464-1472.
58. Rizzuto, R., De Stefani, D., Raffaello, A. and Mammucari, C. (2012) Mitochondria as sensors and regulators of calcium signalling. *Nat. Rev. Mol. Cell. Biol.*, **13**, 566-578.
59. Wu, Y., Rasmussen, T.P., Koval, O.M., Joiner, M.L., Hall, D.D., Chen, B., Luczak, E.D., Wang, Q., Rokita, A.G., Wehrens, X.H. *et al.* (2015) The mitochondrial uniporter controls fight or flight heart rate increases. *Nat. Commun.*, **6**, 6081.
60. Armstrong, C.T., Anderson, J.L. and Denton, R.M. (2014) Studies on the regulation of the human E1 subunit of the 2-oxoglutarate dehydrogenase complex, including the identification of a novel calcium-binding site. *Biochem. J.*, **459**, 369-381.
61. Lane, N. and Martin, W. (2010) The energetics of genome complexity. *Nature*, **467**, 929-934.
62. Castle, J.C., Zhang, C., Shah, J.K., Kulkarni, A.V., Kalsotra, A., Cooper, T.A. and Johnson, J.M. (2008) Expression of 24,426 human alternative splicing events and predicted cis regulation in 48 tissues and cell lines. *Nat. Genet.*, **40**, 1416-1425.
63. Barash, Y., Calarco, J.A., Gao, W., Pan, Q., Wang, X., Shai, O., Blencowe, B.J. and Frey, B.J. (2010) Deciphering the splicing code. *Nature*, **465**, 53-59.
64. Periasamy, M., Herrera, J.L. and Reis, F.C.G. (2017) Skeletal Muscle Thermogenesis and Its Role in Whole Body Energy Metabolism. *Diabetes Metab. J.*, **41**, 327-336.
65. Ingwall, J.S. (2004) Transgenesis and cardiac energetics: new insights into cardiac metabolism. *J. Mol. Cell. Cardiol.*, **37**, 613-623.
66. Benard, G., Faustin, B., Passerieux, E., Galinier, A., Rocher, C., Bellance, N., Delage, J.P., Casteilla, L., Letellier, T. and Rossignol, R. (2006) Physiological diversity of mitochondrial oxidative phosphorylation. *Am. J. Physiol. Cell Physiol.*, **291**, C1172-1182.
67. Chretien, D., Benit, P., Ha, H.H., Keipert, S., El-Khoury, R., Chang, Y.T., Jastroch, M., Jacobs, H.T., Rustin, P. and Rak, M. (2018) Mitochondria are physiologically maintained at close to 50 degrees C. *PLoS Biol.*, **16**, e2003992.

68. Shikata, S., Ozaki, K., Kawai, S., Ito, S. and Okamoto, K. (1988) Purification and characterization of NADP+-linked isocitrate dehydrogenase from an alkalophilic Bacillus. *Biochim. Biophys. Acta*, **952**, 282-289.
69. Maloney, A.P., Callan, S.M., Murray, P.G. and Tuohy, M.G. (2004) Mitochondrial malate dehydrogenase from the thermophilic, filamentous fungus Talaromyces emersonii. *Eur. J. Biochem.*, **271**, 3115-3126.
70. Block, B.A. (1994) Thermogenesis in muscle. *Annu. Rev. Physiol.*, **56**, 535-577.
71. Silva, J.E. (2006) Thermogenic mechanisms and their hormonal regulation. *Physiol. Rev.*, **86**, 435-464.
72. Diogo, R. and Molnar, J. (2014) Comparative anatomy, evolution, and homologies of tetrapod hindlimb muscles, comparison with forelimb muscles, and deconstruction of the forelimb-hindlimb serial homology hypothesis. *Anat. Rec.*, **297**, 1047-1075.
73. Higham, T.E., Korchari, P.G. and McBrayer, L.D. (2011) How muscles define maximum running performance in lizards: an analysis using swing- and stance-phase muscles. *J. Exp. Biol.*, **214**, 1685-1691.
74. Stevenson, E.J., Giresi, P.G., Koncarevic, A. and Kandarian, S.C. (2003) Global analysis of gene expression patterns during disuse atrophy in rat skeletal muscle. *J. Physiol.*, **551**, 33-48.
75. Xu, R., Andres-Mateos, E., Mejias, R., MacDonald, E.M., Leinwand, L.A., Merriman, D.K., Fink, R.H. and Cohn, R.D. (2013) Hibernating squirrel muscle activates the endurance exercise pathway despite prolonged immobilization. *Exp. Neurol.*, **247**, 392-401.
76. Lin, J., Wu, H., Tarr, P.T., Zhang, C.Y., Wu, Z., Boss, O., Michael, L.F., Puigserver, P., Isotani, E., Olson, E.N. *et al.* (2002) Transcriptional co-activator PGC-1 alpha drives the formation of slow-twitch muscle fibres. *Nature*, **418**, 797-801.
77. Puigserver, P. and Spiegelman, B.M. (2003) Peroxisome proliferator-activated receptor-gamma coactivator 1 alpha (PGC-1 alpha): transcriptional coactivator and metabolic regulator. *Endocr. Rev.*, **24**, 78-90.
78. Heinrich, B. (1993). The hot-blooded insects. Springer-Verlag, Berlin, pp. 601.
79. Rowland, L.A., Bal, N.C. and Periasamy, M. (2015) The role of skeletal-muscle-based thermogenic mechanisms in vertebrate endothermy. *Biol. Rev. Camb. Philos. Soc.*, **90**, 1279-1297.
80. Jingting, S., Qin, X., Yanju, S., Ming, Z., Yunjie, T., Gaige, J., Zhongwei, S. and Jianmin, Z. (2017) Oxidative and glycolytic skeletal muscles show marked differences in gene expression profile in Chinese Qingyuan partridge chickens. *PLoS One*, **12**, e0183118.
81. Seebacher, F. and Walter, I. (2012) Differences in locomotor performance between individuals: importance of parvalbumin, calcium handling and metabolism. *J. Exp. Biol.*, **215**, 663-670.
82. MacLennan, D.H. and Phillips, M.S. (1992) Malignant hyperthermia. *Science*, **256**, 789-794.
83. Dias, J.M., Szegedi, C., Jona, I. and Vogel, P.D. (2006) Insights into the regulation of the ryanodine receptor: differential effects of Mg^{2+} and Ca^{2+} on ATP binding. *Biochemistry*, **45**, 9408-9415.
84. Moraru, A., Cakan-Akdogan, G., Strassburger, K., Males, M., Mueller, S., Jabs, M., Muelleder, M., Frejno, M., Braeckman, B.P., Ralser, M. *et al.* (2017) THADA regulates the organismal balance between energy storage and heat production. *Dev. Cell*, **41**, 72-81 e76.
85. Mammucari, C., Gherardi, G., Zamparo, I., Raffaello, A., Boncompagni, S., Chemello, F., Cagnin, S., Braga, A., Zanin, S., Pallafacchina, G. *et al.* (2015) The mitochondrial calcium uniporter controls skeletal muscle trophism in vivo. *Cell Rep.*, **10**, 1269-1279.
86. Logan, C.V., Szabadkai, G., Sharpe, J.A., Parry, D.A., Torelli, S., Childs, A.M., Kriek, M., Phadke, R., Johnson, C.A., Roberts, N.Y. *et al.* (2014) Loss-of-function mutations in MICU1 cause a brain and muscle disorder linked to primary alterations in mitochondrial calcium signaling. *Nat. Genet.*, **46**, 188-193.
87. Hackman, P., Sarparanta, J., Lehtinen, S., Vihola, A., Evila, A., Jonson, P.H., Luque, H., Kere, J., Screen, M., Chinnery, P.F. *et al.* (2013) Welander distal myopathy is caused by a mutation in the RNA-binding protein TIA1. *Ann. Neurol.*, **73**, 500-509.
88. Lee, Y., Jonson, P.H., Sarparanta, J., Palmio, J., Sarkar, M., Vihola, A., Evila, A., Suominen, T., Penttila, S., Savarese, M. *et al.* (2018) TIA1 variant drives myodegeneration in multisystem proteinopathy with SQSTM1 mutations. *J. Clin. Invest.*, **128**, 1164-1177.
89. Ruben, J.A. and Bennett, A.F. (1981) Intense exercise, bone structure and blood calcium levels in vertebrates. *Nature*, **291**, 411-413.
90. Weibel, E.R. and Hoppeler, H. (2005) Exercise-induced maximal metabolic rate scales with muscle aerobic capacity. *J. Exp. Biol.*, **208**, 1635-1644.
91. Territo, P.R., French, S.A., Dunleavy, M.C., Evans, F.J. and Balaban, R.S. (2001) Calcium activation of heart mitochondrial oxidative phosphorylation: rapid kinetics of mVO2, NADH, and light scattering. *J. Biol. Chem.*, **276**, 2586-2599.

92. Bell, C.J., Bright, N.A., Rutter, G.A. and Griffiths, E.J. (2006) ATP regulation in adult rat cardiomyocytes: time-resolved decoding of rapid mitochondrial calcium spiking imaged with targeted photoproteins. *J. Biol. Chem.*, **281**, 28058-28067.
93. Griffiths, E.J. and Rutter, G.A. (2009) Mitochondrial calcium as a key regulator of mitochondrial ATP production in mammalian cells. *Biochim. Biophys. Acta*, **1787**, 1324-1333.
94. Rudolf, R., Mongillo, M., Magalhaes, P.J. and Pozzan, T. (2004) In vivo monitoring of Ca(2+) uptake into mitochondria of mouse skeletal muscle during contraction. *J. Cell Biol.*, **166**, 527-536.
95. Glancy, B., Hartnell, L.M., Malide, D., Yu, Z.X., Combs, C.A., Connelly, P.S., Subramaniam, S. and Balaban, R.S. (2015) Mitochondrial reticulum for cellular energy distribution in muscle. *Nature*, **523**, 617-620.
96. Kavanagh, N.I., Ainscow, E.K. and Brand, M.D. (2000) Calcium regulation of oxidative phosphorylation in rat skeletal muscle mitochondria. *Biochim. Biophys. Acta*, **1457**, 57-70.
97. Waites, G.T., Graham, I.R., Jackson, P., Millake, D.B., Patel, B., Blanchard, A.D., Weller, P.A., Eperon, I.C. and Critchley, D.R. (1992) Mutually exclusive splicing of calcium-binding domain exons in chick alpha-actinin. *J. Biol. Chem.*, **267**, 6263-6271.
98. Wei, B. and Jin, J.P. (2016) TNNT1, TNNT2, and TNNT3: Isoform genes, regulation, and structure-function relationships. *Gene*, **582**, 1-13.
99. Periasamy, M., Strehler, E.E., Garfinkel, L.I., Gubits, R.M., Ruiz-Opazo, N. and Nadal-Ginard, B. (1984) Fast skeletal muscle myosin light chains 1 and 3 are produced from a single gene by a combined process of differential RNA transcription and splicing. *J. Biol. Chem.*, **259**, 13595-13604.
100. Mills, M., Yang, N., Weinberger, R., Vander Woude, D.L., Beggs, A.H., Easteal, S. and North, K. (2001) Differential expression of the actin-binding proteins, alpha-actinin-2 and -3, in different species: implications for the evolution of functional redundancy. *Hum. Mol. Genet.*, **10**, 1335-1346.
101. Head, S.I., Chan, S., Houweling, P.J., Quinlan, K.G., Murphy, R., Wagner, S., Friedrich, O. and North, K.N. (2015) Altered Ca2+ kinetics associated with alpha-actinin-3 deficiency may explain positive selection for ACTN3 null allele in human evolution. *PLoS Genet.*, **11**, e1004862.
102. Ivarsson, N. and Westerblad, H. (2015) alpha-Actinin-3: why gene loss is an evolutionary gain. *PLoS Genet.*, **11**, e1004908.
103. Anderson, K.J., Vermillion, K.L., Jagtap, P., Johnson, J.E., Griffin, T.J. and Andrews, M.T. (2016) Proteogenomic analysis of a hibernating mammal indicates contribution of skeletal muscle physiology to the hibernation phenotype. *J. Proteome Res.*, **15**, 1253-1261.
104. O'Connor, P.M. and Claessens, L.P. (2005) Basic avian pulmonary design and flow-through ventilation in non-avian theropod dinosaurs. *Nature*, **436**, 253-256.
105. Wikstrom, M. and Saari, H. (1975) A spectral shift in cytochrome a induced by calcium ions. *Biochim. Biophys. Acta*, **408**, 170-179.
106. Kirichenko, A., Vygodina, T., Mkrtchyan, H.M. and Konstantinov, A. (1998) Specific cation binding site in mammalian cytochrome oxidase. *FEBS Lett.*, **423**, 329-333.
107. McLain, A.L., Szweda, P.A. and Szweda, L.I. (2011) alpha-ketoglutarate dehydrogenase: a mitochondrial redox sensor. *Free Radic. Res.*, **45**, 29-36.
108. Burr, S.P., Costa, A.S., Grice, G.L., Timms, R.T., Lobb, I.T., Freisinger, P., Dodd, R.B., Dougan, G., Lehner, P.J., Frezza, C. *et al.* (2016) Mitochondrial Protein Lipoylation and the 2-oxoglutarate dehydrogenase complex controls HIF1alpha stability in aerobic conditions. *Cell Metab.*, **24**, 740-752.
109. Nemeria, N.S., Ambrus, A., Patel, H., Gerfen, G., Adam-Vizi, V., Tretter, L., Zhou, J., Wang, J. and Jordan, F. (2014) Human 2-oxoglutarate dehydrogenase complex E1 component forms a thiamin-derived radical by aerobic oxidation of the enamine intermediate. *J. Biol. Chem.*, **289**, 29859-29873.
110. Quinlan, C.L., Goncalves, R.L., Hey-Mogensen, M., Yadava, N., Bunik, V.I. and Brand, M.D. (2014) The 2-oxoacid dehydrogenase complexes in mitochondria can produce superoxide/hydrogen peroxide at much higher rates than complex I. *J. Biol. Chem.*, **289**, 8312-8325.
111. Mailloux, R.J., Gardiner, D. and O'Brien, M. (2016) 2-Oxoglutarate dehydrogenase is a more significant source of O2(.-)/H2O2 than pyruvate dehydrogenase in cardiac and liver tissue. *Free Radic. Biol. Med.*, **97**, 501-512.
112. Nedergaard, J. and Cannon, B. (2013) How brown is brown fat? It depends where you look. *Nat. Med.*, **19**, 540-541.
113. Rolfe, D.F.S. and Brand, M.D. (1996) Proton leak and control of oxidative phosphorylation in perfused, resting rat skeletal muscle. *Biochim. Biophys. Acta. (Bioenergetics)*, **1276**, 45-50.

114. Brown, G.C. and Brand, M.D. (1991) On the nature of the mitochondrial proton leak. *Biochim. Biophys. Acta*, **1059**, 55-62.
115. Glancy, B., Willis, W.T., Chess, D.J. and Balaban, R.S. (2013) Effect of calcium on the oxidative phosphorylation cascade in skeletal muscle mitochondria. *Biochemistry*, **52**, 2793-2809.
116. Marcinek, D.J., Schenkman, K.A., Ciesielski, W.A. and Conley, K.E. (2004) Mitochondrial coupling in vivo in mouse skeletal muscle. *Am. J. Physiol.*, **286**, C457-C463.
117. Korzeniewski, B. (2017) Contribution of proton leak to oxygen consumption in skeletal muscle during intense exercise is very low despite large contribution at rest. *PLoS One*, **12**, e0185991.
118. Storey, K.B. (1997) Metabolic regulation in mammalian hibernation: enzyme and protein adaptations. *Comp. Biochem. Physiol. A. Physiol.*, **118**, 1115-1124.
119. Gehnrich, S.C. and Aprille, J.R. (1988) Hepatic gluconeogenesis and mitochondrial function during hibernation. *Comp. Biochem. Physiol. B.*, **91**, 11-16.
120. Villanueva-Canas, J.L., Faherty, S.L., Yoder, A.D. and Alba, M.M. (2014) Comparative genomics of mammalian hibernators using gene networks. *Integr. Comp. Biol*, **54**, 452-462.
121. Epperson, L.E., Rose, J.C., Russell, R.L., Nikrad, M.P., Carey, H.V. and Martin, S.L. (2010) Seasonal protein changes support rapid energy production in hibernator brainstem. *J. Comp. Physiol. B*, **180**, 599-617.
122. Hindle, A.G., Karimpour-Fard, A., Epperson, L.E., Hunter, L.E. and Martin, S.L. (2011) Skeletal muscle proteomics: carbohydrate metabolism oscillates with seasonal and torpor-arousal physiology of hibernation. *Am. J. Physiol. Regul. Integr. Comp. Physiol.*, **301**, R1440-1452.
123. Bogren, L.K., Grabek, K.R., Barsh, G.S. and Martin, S.L. (2017) Comparative tissue transcriptomics highlights dynamic differences among tissues but conserved metabolic transcript prioritization in preparation for arousal from torpor. *J. Comp. Physiol. B*, **187**, 735-748.
124. Hampton, M., Melvin, R.G., Kendall, A.H., Kirkpatrick, B.R., Peterson, N. and Andrews, M.T. (2011) Deep sequencing the transcriptome reveals seasonal adaptive mechanisms in a hibernating mammal. *PLoS One*, **6**, e27021.
125. Nespolo, R.F., Gaitan-Espitia, J.D., Quintero-Galvis, J.F., Fernandez, F.V., Silva, A.X., Molina, C., Storey, K.B. and Bozinovic, F. (2018) A functional transcriptomic analysis in the relict marsupial Dromiciops gliroides reveals adaptive regulation of protective functions during hibernation. *Mol. Ecol.*, **27**, 4489-4500.
126. Wang, S.Q., Lakatta, E.G., Cheng, H. and Zhou, Z.Q. (2002) Adaptive mechanisms of intracellular calcium homeostasis in mammalian hibernators. *J. Exp. Biol.*, **205**, 2957-2962.
127. Li, X.C., Wei, L., Zhang, G.Q., Bai, Z.L., Hu, Y.Y., Zhou, P., Bai, S.H., Chai, Z., Lakatta, E.G., Hao, X.M. *et al.* (2011) Ca^{2+} cycling in heart cells from ground squirrels: adaptive strategies for intracellular Ca^{2+} homeostasis. *PLoS ONE*, **6**, e24787.
128. Ballinger, M.A., Schwartz, C. and Andrews, M.T. (2017) Enhanced oxidative capacity of ground squirrel brain mitochondria during hibernation. *Am. J. Physiol. Regul. Integr. Comp. Physiol.*, **312**, R301-R310.
129. Fu, W., Hu, H., Dang, K., Chang, H., Du, B., Wu, X. and Gao, Y. (2016) Remarkable preservation of Ca^{2+} homeostasis and inhibition of apoptosis contribute to anti-muscle atrophy effect in hibernating Daurian ground squirrels. *Sci. Rep.*, **6**, 27020.
130. Tessier, S.N., Audas, T.E., Wu, C.W., Lee, S. and Storey, K.B. (2014) The involvement of mRNA processing factors TIA-1, TIAR, and PABP-1 during mammalian hibernation. *Cell. Stress. Chaperones*, **19**, 813-825.
131. Hofmann, S., Cherkasova, V., Bankhead, P., Bukau, B. and Stoecklin, G. (2012) Translation suppression promotes stress granule formation and cell survival in response to cold shock. *Mol. Biol. Cell*, **23**, 3786-3800.
132. Grigg, G.C., Beard, L.A. and Augee, M.L. (2004) The evolution of endothermy and its diversity in mammals and birds. *Physiol. Biochem. Zool.*, **77**, 982-997.
133. Hollenbeck, P.J. (2005) Mitochondria and neurotransmission: evacuating the synapse. *Neuron*, **47**, 331-333.
134. Guo, X., Macleod, G.T., Wellington, A., Hu, F., Panchumarthi, S., Schoenfield, M., Marin, L., Charlton, M.P., Atwood, H.L. and Zinsmaier, K.E. (2005) The GTPase dMiro is required for axonal transport of mitochondria to Drosophila synapses. *Neuron*, **47**, 379-393.
135. Shutov, L.P., Kim, M.S., Houlihan, P.R., Medvedeva, Y.V. and Usachev, Y.M. (2013) Mitochondria and plasma membrane Ca2+-ATPase control presynaptic Ca2+ clearance in capsaicin-sensitive rat sensory neurons. *J. Physiol.*, **591**, 2443-2462.

136. Hamilton, J., Brustovetsky, T., Rysted, J.E., Lin, Z., Usachev, Y.M. and Brustovetsky, N. (2018) Deletion of mitochondrial calcium uniporter incompletely inhibits calcium uptake and induction of the permeability transition pore in brain mitochondria. *J. Biol. Chem.*, **293**, 15652-15663.
137. Campbell, A.K. (2015) *Intracellular calcium*. John Wiley and Sons., Chichester, United Kingdom.
138. Santello, M. and Volterra, A. (2009) Synaptic modulation by astrocytes via Ca^{2+}-dependent glutamate release. *Neuroscience*, **158**, 253-259.
139. Agarwal, A., Wu, P.H., Hughes, E.G., Fukaya, M., Tischfield, M.A., Langseth, A.J., Wirtz, D. and Bergles, D.E. (2017) Transient opening of the mitochondrial permeability transition pore induces microdomain calcium transients in astrocyte processes. *Neuron*, **93**, 587-605.
140. Oheim, M., Schmidt, E. and Hirrlinger, J. (2018) Local energy on demand: Are 'spontaneous' astrocytic Ca^{2+}-microdomains the regulatory unit for astrocyte-neuron metabolic cooperation? *Brain Res. Bull.*, **136**, 54-64.
141. Belanger, M., Allaman, I. and Magistretti, P.J. (2011) Brain energy metabolism: focus on astrocyte-neuron metabolic cooperation. *Cell Metab.*, **14**, 724-738.
142. Jackson, J.G. and Robinson, M.B. (2015) Reciprocal regulation of mitochondrial dynamics and calcium signaling in astrocyte processes. *J. Neurosci.*, **35**, 15199-15213.
143. Angelova, P.R., Kasymov, V., Christie, I., Sheikhbahaei, S., Turovsky, E., Marina, N., Korsak, A., Zwicker, J., Teschemacher, A.G., Ackland, G.L. *et al.* (2015) Functional oxygen sensitivity of astrocytes. *J. Neurosci.*, **35**, 10460-10473.
144. Sheikhbahaei, S., Turovsky, E.A., Hosford, P.S., Hadjihambi, A., Theparambil, S.M., Liu, B., Marina, N., Teschemacher, A.G., Kasparov, S., Smith, J.C. *et al.* (2018) Astrocytes modulate brainstem respiratory rhythm-generating circuits and determine exercise capacity. *Nat. Commun.*, **9**, 370.
145. Winship, I.R., Plaa, N. and Murphy, T.H. (2007) Rapid astrocyte calcium signals correlate with neuronal activity and onset of the hemodynamic response in vivo. *J. Neurosci.*, **27**, 6268-6272.
146. Haydon, P.G. and Carmignoto, G. (2006) Astrocyte control of synaptic transmission and neurovascular coupling. *Physiol. Rev.*, **86**, 1009-1031.
147. Kahlert, S., Zundorf, G. and Reiser, G. (2005) Glutamate-mediated influx of extracellular Ca2+ is coupled with reactive oxygen species generation in cultured hippocampal neurons but not in astrocytes. *J. Neurosci. Res.*, **79**, 262-271.
148. Bradl, M. and Lassmann, H. (2010) Oligodendrocytes: biology and pathology. *Acta Neuropathol.*, **119**, 37-53.
149. Matute, C., Torre, I., Perez-Cerda, F., Perez-Samartin, A., Alberdi, E., Etxebarria, E., Arranz, A.M., Ravid, R., Rodriguez-Antiguedad, A., Sanchez-Gomez, M. *et al.* (2007) P2X(7) receptor blockade prevents ATP excitotoxicity in oligodendrocytes and ameliorates experimental autoimmune encephalomyelitis. *J. Neurosci.*, **27**, 9525-9533.
150. Freeman, M.R. and Rowitch, D.H. (2013) Evolving concepts of gliogenesis: a look way back and ahead to the next 25 years. *Neuron*, **80**, 613-623.
151. Llorente-Folch, I., Rueda, C.B., Amigo, I., del Arco, A., Saheki, T., Pardo, B. and Satrustegui, J. (2013) Calcium-regulation of mitochondrial respiration maintains ATP homeostasis and requires ARALAR/AGC1-malate aspartate shuttle in intact cortical neurons. *J. Neurosci.*, **33**, 13957-13971, 13971a.
152. Rueda, C.B., Llorente-Folch, I., Amigo, I., Contreras, L., Gonzalez-Sanchez, P., Martinez-Valero, P., Juaristi, I., Pardo, B., del Arco, A. and Satrustegui, J. (2014) Ca^{2+} regulation of mitochondrial function in neurons. *Biochim. Biophys. Acta*, **1837**, 1617-1624.
153. Contreras, L. (2015) Role of AGC1/aralar in the metabolic synergies between neuron and glia. *Neurochem. Int.*, **88**, 38-46.
154. Contreras, L. and Satrustegui, J. (2009) Calcium signaling in brain mitochondria: interplay of malate aspartate NADH shuttle and calcium uniporter/mitochondrial dehydrogenase pathways. *J. Biol. Chem.*, **284**, 7091-7099.
155. Pardo, B., Contreras, L., Serrano, A., Ramos, M., Kobayashi, K., Iijima, M., Saheki, T. and Satrustegui, J. (2006) Essential role of aralar in the transduction of small Ca2+ signals to neuronal mitochondria. *J. Biol. Chem.*, **281**, 1039-1047.
156. Chin, R.M., Fu, X., Pai, M.Y., Vergnes, L., Hwang, H., Deng, G., Diep, S., Lomenick, B., Meli, V.S., Monsalve, G.C. *et al.* (2014) The metabolite alpha-ketoglutarate extends lifespan by inhibiting ATP synthase and TOR. *Nature*, **510**, 397-401.

157. Gebauer, J., Schuster, S., de Figueiredo, L.F. and Kaleta, C. (2012) Detecting and investigating substrate cycles in a genome-scale human metabolic network. *FEBS J.*, **279**, 3192-3202.
158. Shepard, J., Reick, M., Olson, S. and Graveley, B.R. (2002) Characterization of U2AF(26), a splicing factor related to U2AF(35). *Mol. Cell. Biol.*, **22**, 221-230.
159. Pacheco, T.R., Gomes, A.Q., Barbosa-Morais, N.L., Benes, V., Ansorge, W., Wollerton, M., Smith, C.W., Valcarcel, J. and Carmo-Fonseca, M. (2004) Diversity of vertebrate splicing factor U2AF35: identification of alternatively spliced U2AF1 mRNAS. *J. Biol. Chem.*, **279**, 27039-27049.
160. Wu, J.Y. and Maniatis, T. (1993) Specific interactions between proteins implicated in splice site selection and regulated alternative splicing. *Cell*, **75**, 1061-1070.
161. Kralovicova, J., Knut, M., Cross, N.C. and Vorechovsky, I. (2015) Identification of U2AF(35)-dependent exons by RNA-Seq reveals a link between 3' splice-site organization and activity of U2AF-related proteins. *Nucleic Acids Res.*, **43**, 3747-3763.
162. Chang, J.W., Yeh, H.S., Park, M., Erber, L., Sun, J., Cheng, S., Bui, A.M., Fahmi, N.A., Nasti, R., Kuang, R. *et al.* (2019) mTOR-regulated U2af1 tandem exon splicing specifies transcriptome features for translational control. *Nucleic Acids Res.*, **47**, 10373-10387.
163. McNab, B.K. (1978) The evolution of endothermy in the phylogeny of mammals. *Am. Nat.*, **112**, 1-21.
164. Geiser, F. (1998) Evolution of daily torpor and hibernation in birds and mammals: importance of body size. *Clin. Exp. Pharmacol. Physiol.*, **25**, 736-739.
165. Kemp, T.S. (2006) The origin of mammalian endothermy: a paradigm for the evolution of complex biological structure. *Zool. J. Linnean Soc.*, **147**, 473–488.
166. Clarke, A. and Portner, H.O. (2010) Temperature, metabolic power and the evolution of endothermy. *Biol. Rev. Camb. Philos. Soc.*, **85**, 703-727.
167. Lovegrove, B.G. (2017) A phenology of the evolution of endothermy in birds and mammals. *Biol. Rev. Camb. Philos. Soc.*, **92**, 1213-1240.
168. Roussel, D., Salin, K., Dumet, A., Romestaing, C., Rey, B. and Voituron, Y. (2015) Oxidative phosphorylation efficiency, proton conductance and reactive oxygen species production of liver mitochondria correlates with body mass in frogs. *J. Exp. Biol.*, **218**, 3222-3228.
169. Brand, M.D., Turner, N., Ocloo, A., Else, P.L. and Hulbert, A.J. (2003) Proton conductance and fatty acyl composition of liver mitochondria correlates with body mass in birds. *Biochem. J.*, **376**, 741-748.
170. Zhivotovsky, B. and Orrenius, S. (2011) Calcium and cell death mechanisms: a perspective from the cell death community. *Cell Calcium*, **50**, 211-221.
171. Pacher, P. and Hajnoczky, G. (2001) Propagation of the apoptotic signal by mitochondrial waves. *EMBO J.*, **20**, 4107-4121.
172. Giorgi, C., Baldassari, F., Bononi, A., Bonora, M., De Marchi, E., Marchi, S., Missiroli, S., Patergnani, S., Rimessi, A., Suski, J.M. *et al.* (2012) Mitochondrial Ca^{2+} and apoptosis. *Cell Calcium*, **52**, 36-43.
173. Ferron, H.G. (2017) Regional endothermy as a trigger for gigantism in some extinct macropredatory sharks. *PLoS One*, **12**, e0185185.
174. Pan, H., Yu, H., Ravi, V., Li, C., Lee, A.P., Lian, M.M., Tay, B.H., Brenner, S., Wang, J., Yang, H. *et al.* (2016) The genome of the largest bony fish, ocean sunfish (*Mola mola*), provides insights into its fast growth rate. *Gigascience*, **5**, 36.
175. Hutchison, V.H., Dowling, H.G. and Vinegar, A. (1966) Thermoregulation in a brooding female Indian python, *Python molurus bivittatus*. *Science*, **151**, 694-696.
176. Frair, W., Ackman, R.G. and Mrosovsky, N. (1972) Body temperature of *Dermochelys coriacea*: warm turtle from cold water. *Science*, **177**, 791-793.
177. Runcie, R.M., Dewar, H., Hawn, D.R., Frank, L.R. and Dickson, K.A. (2009) Evidence for cranial endothermy in the opah (*Lampris guttatus*). *J. Exp. Biol.*, **212**, 461-470.
178. Grady, J.M., Enquist, B.J., Dettweiler-Robinson, E., Wright, N.A. and Smith, F.A. (2014) Dinosaur physiology. Evidence for mesothermy in dinosaurs. *Science*, **344**, 1268-1272.
179. Bennett, A.F. and Ruben, J.A. (1979) Endothermy and activity in vertebrates. *Science*, **206**, 649-654.
180. Killen, S.S., Glazier, D.S., Rezende, E.L., Clark, T.D., Atkinson, D., Willener, A.S. and Halsey, L.G. (2016) Ecological influences and morphological correlates of resting and maximal metabolic rates across teleost fish species. *Am. Nat.*, **187**, 592-606.
181. Nespolo, R.F., Solano-Iguaran, J.J. and Bozinovic, F. (2017) Phylogenetic analysis supports the aerobic-capacity model for the evolution of endothermy. *Am. Nat.*, **189**, 13-27.
182. Block, B.A., Finnerty, J.R., Stewart, A.F. and Kidd, J. (1993) Evolution of endothermy in fish: mapping physiological traits on a molecular phylogeny. *Science*, **260**, 210-214.

183. Fell, D.A. and Thomas, S. (1995) Physiological control of metabolic flux: the requirement for multisite modulation. *Biochem. J.*, **311** (Pt 1), 35-39.
184. Buljan, M., Chalancon, G., Dunker, A.K., Bateman, A., Balaji, S., Fuxreiter, M. and Babu, M.M. (2013) Alternative splicing of intrinsically disordered regions and rewiring of protein interactions. *Curr. Opin. Struct. Biol.*, **23**, 443-450.
185. Territo, P.R., Mootha, V.K., French, S.A. and Balaban, R.S. (2000) Ca^{2+} activation of heart mitochondrial oxidative phosphorylation: role of the F_0/F_1-ATPase. *Am. J. Physiol. Cell Physiol.*, **278**, C423-435.
186. Abascal, F., Tress, M.L. and Valencia, A. (2015) The evolutionary fate of alternatively spliced homologous exons after gene duplication. *Genome Biol. Evol.*, **7**, 1392-1403.
187. Dry, I.B. and Wiskich, J.T. (1987) 2-Oxoglutarate dehydrogenase and pyruvate dehydrogenase activities in plant mitochondria: interaction via a common coenzyme a pool. *Arch. Biochem. Biophys.*, **257**, 92-99.
188. Pfeiffer, T., Schuster, S. and Bonhoeffer, S. (2001) Cooperation and competition in the evolution of ATP-producing pathways. *Science*, **292**, 504-507.
189. Hayakawa, M., Sakashita, E., Ueno, E., Tominaga, S., Hamamoto, T., Kagawa, Y. and Endo, H. (2002) Muscle-specific exonic splicing silencer for exon exclusion in human ATP synthase gamma-subunit pre-mRNA. *J. Biol. Chem.*, **277**, 6974-6984.
190. Jin, Y., Suzuki, H., Maegawa, S., Endo, H., Sugano, S., Hashimoto, K., Yasuda, K. and Inoue, K. (2003) A vertebrate RNA-binding protein Fox-1 regulates tissue-specific splicing via the pentanucleotide GCAUG. *EMBO J.*, **22**, 905-912.
191. Kuroyanagi, H. (2009) Fox-1 family of RNA-binding proteins. *Cell. Mol. Life Sci.*, **66**, 3895-3907.
192. Hopper, R.K., Carroll, S., Aponte, A.M., Johnson, D.T., French, S., Shen, R.F., Witzmann, F.A., Harris, R.A. and Balaban, R.S. (2006) Mitochondrial matrix phosphoproteome: effect of extra mitochondrial calcium. *Biochemistry*, **45**, 2524-2536.
193. Vecellio Reane, D., Vallese, F., Checchetto, V., Acquasaliente, L., Butera, G., De Filippis, V., Szabo, I., Zanotti, G., Rizzuto, R. and Raffaello, A. (2016) A MICU1 splice variant confers high sensitivity to the mitochondrial Ca^{2+} uptake machinery of skeletal muscle. *Mol. Cell*, **64**, 760-773.
194. Ding, J.H., Xu, X., Yang, D., Chu, P.H., Dalton, N.D., Ye, Z., Yeakley, J.M., Cheng, H., Xiao, R.P., Ross, J. *et al.* (2004) Dilated cardiomyopathy caused by tissue-specific ablation of SC35 in the heart. *EMBO J.*, **23**, 885-896.
195. Eldred, C.C., Katzemich, A., Patel, M., Bullard, B. and Swank, D.M. (2014) The roles of troponin C isoforms in the mechanical function of Drosophila indirect flight muscle. *J. Muscle Res. Cell Motil.*, **35**, 211-223.
196. Kubis, H.P., Haller, E.A., Wetzel, P. and Gros, G. (1997) Adult fast myosin pattern and Ca^{2+}-induced slow myosin pattern in primary skeletal muscle culture. *Proc. Natl. Acad. Sci. USA*, **94**, 4205-4210.
197. Teusink, B., Wiersma, A., Molenaar, D., Francke, C., de Vos, W.M., Siezen, R.J. and Smid, E.J. (2006) Analysis of growth of *Lactobacillus plantarum* WCFS1 on a complex medium using a genome-scale metabolic model. *J. Biol. Chem.*, **281**, 40041-40048.
198. Samoilov, M., Plyasunov, S. and Arkin, A.P. (2005) Stochastic amplification and signaling in enzymatic futile cycles through noise-induced bistability with oscillations. *Proc. Natl. Acad. Sci. USA*, **102**, 2310-2315.
199. Nicol, S.C. (2017) Energy Homeostasis in Monotremes. *Front.Neurosci.*, **11**, 195.
200. Dawson, T.R., Grant, T.R. and Fanning, D. (1979) Standard metabolism of monotremes and the evolution of homeothermy. *Aust. J. Zool.*, **27**, 511-515.
201. Wrigley, J.M. and Graves, J.A. (1984) Two monotreme cell lines, derived from female platypuses (*Ornithorhynchus anatinus*; Monotremata, Mammalia). *In Vitro*, **20**, 321-328.
202. Kondrashov, F.A., Koonin, E.V., Morgunov, I.G., Finogenova, T.V. and Kondrashova, M.N. (2006) Evolution of glyoxylate cycle enzymes in Metazoa: evidence of multiple horizontal transfer events and pseudogene formation. *Biol. Direct*, **1**, 31.
203. Dolan, S.K. and Welch, M. (2018) The glyoxylate shunt, 60 Years On. *Annu. Rev. Microbiol.*, **72**, 309-330.
204. Dean, J.T., Tran, L., Beaven, S., Tontonoz, P., Reue, K., Dipple, K.M. and Liao, J.C. (2009) Resistance to diet-induced obesity in mice with synthetic glyoxylate shunt. *Cell Metab.*, **9**, 525-536.
205. Cannon, B. and Nedergaard, J. (2004) Brown adipose tissue: function and physiological significance. *Physiol. Rev.*, **84**, 277-359.
206. Walter, I. and Seebacher, F. (2009) Endothermy in birds: underlying molecular mechanisms. *J. Exp. Biol.*, **212**, 2328-2336.

207. Ledesma, A., de Lacoba, M.G. and Rial, E. (2002) The mitochondrial uncoupling proteins. *Genome Biol*, **3**, REVIEWS3015.
208. Graier, W.F., Frieden, M. and Malli, R. (2007) Mitochondria and Ca^{2+} signaling: old guests, new functions. *Pflugers Arch.*, **455**, 375-396.
209. Trenker, M., Malli, R., Fertschai, I., Levak-Frank, S. and Graier, W.F. (2007) Uncoupling proteins 2 and 3 are fundamental for mitochondrial Ca^{2+} uniport. *Nat. Cell. Biol.*, **9**, 445-452.
210. Brookes, P.S., Parker, N., Buckingham, J.A., Vidal-Puig, A., Halestrap, A.P., Gunter, T.E., Nicholls, D.G., Bernardi, P., Lemasters, J.J. and Brand, M.D. (2008) UCPs - unlikely calcium porters. *Nat. Cell. Biol.*, **10**, 1235-1237; author reply 1237-1240.
211. Altringham, J.D. and Block, B.A. (1997) Why do tuna maintain elevated slow muscle temperatures? Power output of muscle isolated from endothermic and ectothermic fish. *J. Exp. Biol.*, **200**, 2617-2627.
212. Green, R.E., Braun, E.L., Armstrong, J., Earl, D., Nguyen, N., Hickey, G., Vandewege, M.W., St. John, J.A., Capella-Gutierrez, S., Castoe, T.A. *et al.* (2014) Three crocodilian genomes reveal ancestral patterns of evolution among archosaurs. *Science*, **346**, 1254449.
213. Farmer, C.G. and Carrier, D.R. (2000) Ventilation and gas exchange during treadmill locomotion in the American alligator (*Alligator mississippiensis*). *J. Exp. Biol.*, **203**, 1671-1678.
214. Seymour, R.S. (2013) Maximal aerobic and anaerobic power generation in large crocodiles versus mammals: implications for dinosaur gigantothermy. *PLoS One*, **8**, e69361.
215. Bostrom, B.L., Jones, T.T., Hastings, M. and Jones, D.R. (2010) Behaviour and physiology: the thermal strategy of leatherback turtles. *PLoS One*, **5**, e13925.
216. Heaslip, S.G., Iverson, S.J., Bowen, W.D. and James, M.C. (2012) Jellyfish support high energy intake of leatherback sea turtles (*Dermochelys coriacea*): video evidence from animal-borne cameras. *PLoS One*, **7**, e33259.
217. Clarke, A. and Rothery, P. (2008) Scaling of body temperature in mammals and birds. *Functional Ecol.* **22**, 58–67.
218. Chacko, E. and Ranganathan, S. (2009) Comprehensive splicing graph analysis of alternative splicing patterns in chicken, compared to human and mouse. *BMC Genomics*, **10** Suppl 1, S5.
